# Supplementary material for: Evaluation of a community health worker home visit intervention to improve child development in South Africa: A cluster-randomized controlled trial
Source: PLoS Med. 2023 Apr 14;20(4):e1004222. doi: 10.1371/journal.pmed.1004222 (PMC10146459; doi:10.1371/journal.pmed.1004222)
Supplement: S1 Text — (DOCX) [file pmed.1004222.s002.docx]

**S1 Text. Study protocol**

# Executive summary

**The goal of this project is to evaluate the impact and scalability of an innovative home visit intervention designed to improve early childhood development. The intervention will be integrated into an existing community health worker program in South Africa and evaluated through a cluster-randomized trial.**

Several recent studies have highlighted the potential of home-based interventions to positively impact early childhood outcomes: Integrated community case management (iCCM) has been shown to reduce child illness [1]; parental training in infant and young child feeding (IYCF) practices has been shown to improve nutrition and physical development [2]; home-based stimulation has been shown to increase child cognitive and socioemotional development [3-5]; and basic cognitive behavioral therapy (CBT) has been shown to reduce maternal depression as one of the best predictors of early childhood development (ECD) [6,7]. As part of the *Reengineering Primary Health Care* initiative, the South African National Department of Health (NDoH) has committed to expanding access to home-based care provided by community health workers (CHWs). However, the protocols for home visits made by CHWs in the country are limited and, with the exception of some iCCM strategies, do not include the interventions described above. In this project, we will enrich the current CHW curriculum with an additional package of interventions, which we believe has great potential to improve early childhood health and development in South Africa. To assess the feasibility and impact of this enriched curriculum, we will conduct a cluster-randomized trial in Mopani District, Limpopo Province with CHWs and caregiver-child pairs.

To ensure a successful project, the study team combines several complimentary skills and expertise, including: local research capacity at the Health Economics and Epidemiology Research Office (HE^2^RO) at the Wits Health Consortium, University of the Witwatersrand; local health system management and implementation capacity at the Anova Health Institute; and international expertise in early childhood health and development at Boston University and Harvard University. HE^2^RO and Anova have strong relationships with key stakeholders in the South African health sector, including at the NDoH. Furthermore, the Limpopo Province and Mopani District Departments of Health will advise on this project. If the study intervention package is found to have a strong positive impact on early childhood outcomes with minimal disruption of CHW operations, we anticipate strong interest in larger-scale versions of this program from all stakeholders.

# Background

South Africa is home to more than 5.7 million children under the age of five [8]. While the country is classified as upper-middle income, economic inequality remains high. More than half of children are living in poverty [9], and 24% of children are stunted, a rate very similar to those found in nearby low-income countries [10]. Research conducted in South Africa and elsewhere shows that childhood stunting strongly predicts later cognitive and schooling outcomes [11,12], contributing to low levels of literacy and educational attainment in the country [13,14].

More generally, there is increasing recognition that health and nutrition during the earliest years of life are key determinants of later life outcomes, including success in school and job performance [15]. Chronic infections that go untreated, poor nutrition, and lack of stimulating activity during childhood have all been shown to contribute to deficits in ECD, including deficits in fine motor, verbal, and math skills. One of the most important risk factors for ECD is maternal depression, which is highly common among poor mothers in South Africa [16]. Depressed mothers are often unable to provide the resources and emotional support that developing children require; depression has been shown to be associated with developmental deficits in children in several studies, including some conducted in South Africa [7,17,18].

A growing body of evidence shows that home visits by CHWs focusing on childhood health and nutrition have the potential to achieve substantial improvements in ECD outcomes [19]. Integrated community case management (iCCM) services delivered at the home by health workers have been shown to reduce child illness [1]. Parental training in infant and young child feeding (IYCF) practices has been shown to improve nutrition and physical development [2]. Support for home-based child stimulation activities has been shown to increase cognitive and socioemotional development [3-5]. Finally, basic cognitive behavioral therapy (CBT) delivered at home by healthcare workers has been shown to reduce maternal depression [6], a key determinant of early childhood development [7].

In terms of the local political context, many of the current efforts to address ECD in South Africa are delivered through the education sector, although the health and social development sectors also play key roles [20,21]. The Department of Basic Education (DoBE) has taken a leadership role on the issue and has developed a curriculum devoted to ECD [22]. Furthermore, many ECD programs run by NGOs in South Africa use a center-based model integrated with the public education system [23]. These programs are very important; quality early education and other center-based programs have been shown to be quite effective at improving ECD outcomes [24]. However, one key limitation of center-based models of service delivery is that they require active attendance by parents and children, and thus are subject to access barriers that likely disproportionately affect the poorest members of society. Furthermore, center-based ECD services are often targeted at older children, and may miss children in the critical first 1,000 days of life. Indeed, there are important challenges to providing center-based ECD services to infants, as these children require more intensive monitoring and may have difficulty being apart from their caregivers.

As part of the *Reengineering Primary Health Care* initiative, the South African NDoH has committed to expanding access to home-based care provided by CHWs. However, the protocols for home visits made by CHWs in the country are limited and, with the exception of some iCCM strategies, do not include interventions focused on ECD. We believe that there is great potential to deliver home-based ECD services and positively impact early childhood health and development in South Africa without overburdening CHWs. We will spend time at the start of the project period developing and piloting a package of ECD interventions that can be integrated into current CHW protocols in South Africa. To maximize the feasibility of the intervention package, curriculums will be adapted from similar programs currently operating in other settings. Two versions of the curriculum will be developed: the first version will emphasize caregiver-directed interactions focused on developing and reinforcing skills within the child’s zone of proximal development; the second version will encourage caregiver-child interactions focused on strengthening responsiveness. After the intervention packages are finalized, we will conduct a cluster-randomized trial with CHWs and caregiver-child pairs in two sub-districts within Mopani District, Limpopo Province: Greater Tzaneen and Greater Giyani.

A random subsample of caregiver-child pairs from Greater Tzaneen sub-district enrolled in the intervention trial will be invited to participate in repeated assessments of neural function. The selected pairs will be transported by the study team to a centrally located lab in Tzaneen, where children will be measured using electroencephalogram (EEG) and eye-tracking (ET) technologies. These methods provide high-resolution data on important neural processes and are a novel approach to assessing child development that has not previously been widely applied in South Africa but have the potential to be developed for general use.

# Scope and Approach

***Overview of approach***

In this project, we will conduct a cluster-randomized trial to evaluate the impact of an innovative early childhood intervention delivered by CHWs during routine home-based maternal and child health visits. We will spend the first part of the project period developing and piloting a package of interventions. We anticipate including a set of interventions found to be effective in settings similar to South Africa: reinforced support for a set of iCCM protocols to address child illness [1]; parental training in IYCF practices to improve early-life nutrition [2]; support for cognitive stimulation methods to improve cognitive and socioemotional development [3-5]; and CBT to address maternal depression [6]. To maximize the feasibility of package uptake by CHWs, we plan to adapt existing curricula used for similar interventions in other settings.

The proposed study will test two main hypotheses:

H1: **Feasibility:** A comprehensive package of early childhood interventions can be effectively integrated into existing CHW protocols in South Africa; and

H2: **Impact:** The intervention can measurably improve early childhood development.

***Objectives***

Table 1 outlines the main objectives of the project within a ‘theory of change’ framework that identifies relevant outputs for each objective.

**Table 1.** Theory of Change framework

| **Objectives** | **Outputs** | **Intermediate outcome** | **Primary outcome** |
| --- | --- | --- | --- |
| 1. Study pilot | 1. Focus group discussions (FGDs) with CHW 2. FGDs with caregivers 3. Final intervention package 4. CHW household visit protocols 5. Data collection instruments and data management processes | 1. Delivery of home visit intervention 2. Implementation of high-quality research protocols | 1. Height-for-age z-score (HAZ) and stunting 2. Child development measured using the BSID-III 3. Absolute EEG gamma power 4. Saccadic reaction time (SRT) |
| 1. CHW selection | 1. Enrollment CHWs |  |  |
| 1. Household selection and baseline assessment | 1. Enrollment of households 2. Baseline data for each enrolled household |  |  |
| 1. Implementation of intervention | 1. Random assignment of CHWs to intervention or control groups 2. Random assignment of intervention package variant within treatment group 3. Intervention CHWs trained to provide package of early childhood services |  |  |
| 1. Endline assessment of households | 1. Endline data for each enrolled household |  |  |
| 1. Data analysis and reporting | 1. Peer-reviewed publication 2. Report of study evidence for key stakeholders 3. Dissemination activities |  |  |

The project has six main objectives:

1. *Conduct an initial pilot phase of research to establish CHW curriculum.* Key outputs for this objective include: FGDs with CHWs and caregivers; determination of topics to be included in the final study package; development of child-age-specific household visit protocols integrating package interventions into existing CHW standards; and refinement of data collection instruments and data management processes.
2. *Select a random sample of CHWs to include in the study.* Key outputs for this objective include: selection of CHWs. A short questionnaire will be used to collect information from all CHWs in Greater Tzaneen and Greater Giyani. This information will be used for the randomization procedure (see details below).
3. *Select and enroll caregiver-child pairs that meet eligibility criteria and conduct a baseline assessment of demographics.* Key outputs for this objective include: completed enrollment of caregiver-child pairs; and completed baseline surveys for each enrolled pair. Caregiver-child pairs will be recruited soon after birth. Key indicators to be measured include: household demographics and details of the child’s birth, including gestational age at birth and birthweight.
4. *Implement a home-based intervention among a randomly selected group of study CHWs.* Key outputs for this objective include: i) randomization at the WBOT-level to either the intervention or control group; ii) random assignment of intervention package variant within treatment group; and iii) training of selected CHWs to deliver the intervention package as part of their standard protocols.
5. *Conduct an endline assessment of the sample of caregiver-child pairs assessed at baseline.* The primary outcomes for this study are child development and neural function. Child length will be measured at endline and converted to HAZ for analysis; stunting status (HAZ < -2) will also be calculated for analysis. Skill development indicators and neural function measures will be collected at endline (we provide a summary of these measures below). We will aim to minimize loss to follow-up during the study period.
6. *Analyze study data and report findings to key stakeholders.* Reporting activities will include specific efforts to engage with NDoH representatives in an attempt to disseminate key findings and mobilize support for scale-up should the intervention package prove effective.

Key intermediate outcomes for the proposed study are: i) delivery of a home-based early childhood intervention; and ii) implementation of high-quality research protocols.

***Setting***

The study will be conducted in Greater Tzaneen and Greater Giyani, Mopani District, Limpopo Province. Over the past few years, as part of the *Reengineering of Primary Health Care* initiative, CHWs in Mopani District (as elsewhere in South Africa) have been reorganized and deployed within WBOTs. In Mopani District the Anova Health Institute, a key partner for the proposed study, supports the Department of Health to manage CHW and WBOT operations.

Mopani District is located on the eastern side of Limpopo Province and has a total population of 1,092,507, including more than 56,000 children under the age of two. Within Greater Tzaneen, there are 282 CHWs operating in 38 WBOTs. Within Greater Giyani, there are 268 CHWs operating in 30 WBOTs. Figure 1 is a map of Mopani District with ward demarcations.

**Figure 1.** Map of Mopani District with ward demarcations

[Figure removed for

rights reasons]

*Notes*: Map outlining Mopani District and wards in eastern Limpopo Province

***Study design***

The study will employ a cluster-randomized trial design. After an initial phase during which the home-based intervention package will be developed and piloted, and data collection procedures finalized, all CHWs operating in Greater Tzaneen and Greater Giyani will be recruited to participate in the study. We will then randomize CHWs with equal probability at the WBOT-level to either the intervention or control group. Within the treatment arm, WBOTs will be randomized to one of the two variants of the intervention package. A covariate constrained randomization procedure will be used to balance treatment groups on key descriptive variables (e.g., population demographics, indicators of disease burden) and increase statistical power. CHWs randomized to the intervention group will be trained to provide the ECD program package.

Children born to parents residing within each study CHW’s catchment area will be enrolled in the study. Caregiver-child pairs will be recruited at the home soon after birth. CHWs in the study area track all pregnancies and deliveries and their logs will be used to identify eligible households. CHW logs will be checked with local community members for missing births during baseline household visits. Eligible pairs, i.e., those born after the start of the enrollment period.

***Intervention***

Two variations on the intervention package will be developed and piloted during the first year of the study period. Both intervention arms will include components related to iCCM to address child infection, IYCF to address child nutrition, and CBT to address maternal mental health. The two arms will differ only with respect to the suggested play-based activities and the approach to how caregivers interacted with their child during play. The first version will emphasize caregiver-directed interactions focused on developing and reinforcing skills within the child’s zone of proximal development. The second version will encourage caregiver-child interactions focused on strengthening responsiveness.

Intervention activities will be added to current CHW protocols for home-based health visits. The overall structure of intervention visits will be informed by similar research in other settings [19,25,26], and we will also use the Care for Child Development (CCD) Package developed by UNICEF and WHO as a resource [27]. We will work with partners in the Mopani District Department of Health and the Limpopo Province Department of Health in designing the intervention package, to ensure maximum feasibility.

***Recruitment and Enrollment***

All CHWs operating in Greater Tzaneen and Greater Giyani, Mopani District, will be invited to participate in the study. The primary eligibility criterion for CHWs is that they must be currently active at the time of the study and they must be conducting home-based child health visits as part of their routine activities. Nearly all CHWs in the District meet these criteria.

After CHWs are enrolled, eligible caregiver-child pairs residing within the wards served by study CHWs (both intervention and control) will be sampled and recruited to the study. Recruitment and enrollment of caregiver-child pairs will occur as part of baseline data collection activities. To ensure community support for study activities, the project will be introduced to local authorities and community leaders. After making sure that the study’s goals and procedures are acceptable to the community, eligible caregiver-child pairs will be identified by reviewing on a rolling basis the standard birth registration records that CHWs maintain. Eligibility criteria for caregiver-child pairs are provided below. Eligible households will be visited, information about the scope and content of the study will be provided to caregivers, and if they are willing to provide consent to participate in the study, the caregiver and child will be enrolled.

Inclusion criteria for caregiver-child pairs

1. Households within study CHW catchment areas with a child recruited soon after birth
2. Mother or primary care giver must be 18 years or older

A random sub-sample of pairs from Greater Tzaneen will be recruited to participate in repeated assessments of neural function. Children enrolled in the trial from Greater Giyani will not be recruited for these assessments because the long distances they would need to travel to the lab would make this infeasible. Pairs invited to participate in these assessments will be invited to come to a lab which will be centrally located in Tzaneen. A study vehicle will pick up caregivers and children at their homes and transport them to the lab for measurement, and then transport them back to their homes.

Inclusion criteria:

- Children enrolled in the intervention trial residing in Greater Tzaneen

Exclusion criteria:

- Children born with low birth weight (<2500g);
- Children with a diagnosed developmental disorder.

***Data collection***

The impact of the intervention package on child development indicators will be assessed with data collected at households and at the central lab. Each of the caregiver-child pairs enrolled in the study will be administered a survey at their household at baseline and endline that will include questions related to: household demographics; parent’s knowledge and perceptions of child health and nutrition; and assessment of maternal depression. At baseline, we will collect information on birthweight and gestational age at birth. At endline, study children will be assessed for anthropometrics (e.g., height, weight, mid-upper arm circumference) as measures of physical development. At endline, study children will be assessed for development using the Bayley Scale for Infant and Toddler Development (BSID-III).

EEG: When conducting EEG assessments, assessors will first measure the child’s head circumference using a tape measure, to determine what size of sensor net will fit the child’s head. The sensor net is a stretchy cap made of soft sponges attached to electrodes, which each record brain activity from a different area of the child’s scalp. Each sensor is attached to a wire to record the electrical activity. After determining head circumference, the sensor net will be soaked for 10 minutes in a warm potassium chloride solution to facilitate electrical conductance with the scalp. Caregivers will be seated on a chair on the assessment side of the partition and children will be seated on their caregiver’s lap. The assessor will entertain the child and provided them with toys while the EEG sensor net is placed on the child’s head and secured with a chin strap. The assessor will make sure each sensor had a good connection with the scalp, and, when necessary, apply more potassium chloride solution with an eyedropper and carefully move hair out of the way of the sensors. Caregivers will be asked to remove all braids in the children’s hair prior to attending the lab visit to ensure a good connection can be obtained. Once impedances below 50 ohms are established for all sensors, the EEG will record from all channels with reference to the vertex while the child sits on the caregiver’s lap with the lights dimmed. The assessor and caregiver will not speak during the recording. When necessary to keep the child calm during the recording, the assessor will show the child a toy. After recording is complete, the sensor net will be removed, rinsed, and disinfected.

[Figure removed for

rights reasons]

**Figure 2: Infant wearing EEG sensor cap**

Eye-tracking: To record infants’ eye movements, we will use a commercially available, screen-based eye tracker (Tobii X3-120) that meets all necessary safety requirements. Similar technology has been used extensively in prior studies with infants and young children. The eye tracker uses infrared light reflections and special cameras to record information about the position of the infant’s pupil, the reflection of a point-light that is projected from the eye-tracker on the infant’s eyeball, and the position of the infant’s head in 3D space. When combined, these three sources of information can be used to estimate where the infant is looking and to track infants’ eye movements at high temporal and spatial resolution.

The caregiver remains seated and holding the child on their lap (Figure 3). The caregiver will be positioned so that the child’s eyes are facing forward and at approximately 60cm viewing distance from the eye tracker and computer monitor. During the assessment, the child will be presented with short, alternating blocks of visual stimuli on the monitor that are designed to calibrate the eye tracking system, measure SRTs and fixations to social scenes.

**Figure 3. Eye-Tracking**

**

[Figure removed for

rights reasons]

*Notes: A) An illustration of the setup for recording eye movement from infants. B) Infants are shown high-contrast stimuli against an isoluminous background on computer screen. The stimuli move unexpectedly from one location (e.g., center) to another location (e.g., upper left corner). C-D) Given infants’ preference for novel stimuli and abrupt onsets, infants will typically shift attention from the first to the second stimulus (i.e., perform a saccadic eye movement), as illustrated by a step -like change in the x-coordinates in the eye tracking trace. The latency and frequency of these eye movements can be used to measure various visual and cognitive function in infants, as explained in the text.*

***Measurement of Outcomes***

Measures of child development to be investigated in this study are:

- *Mean HAZ (and stunting, defined as HAZ < -2):* Heights of study children will be measured at baseline and endline; HAZ will be computed according to WHO child growth standards.
- *BSID-III composite scores*: Study children will be assessed at endline using the BSID-III, which includes five domains of neurocognitive development: cognition; language; motor; adaptive behavior; and social-emotional.

The primary outcome for the EEG assessment is:

- *Absolute EEG Gamma Power:* high frequency oscillations that index synchronization of neuronal firing. Existing evidence has linked gamma power to cognition and language in early childhood.

The primary outcome for the eye-tracking assessment is:

- *Mean saccadic reaction time*: speed of visual orienting.

# Procedures and Risk Mitigation

CHWs eligible to participate in the study will be provided with information about the study prior to agreeing to participate. All CHWs agreeing to participate in the study will be asked to sign an informed consent document. All caregivers agreeing to participate in the study will also be asked to sign an informed consent document.

We will hire a team of qualified interviewers to implement all study procedures. These interviewers will undergo a rigorous training prior to beginning fieldwork. They will be trained to implement the procedures and also to protect the rights of the participants, including by maintaining strict confidentiality in all data collected.

We expect only minimal risks for children and their caregivers. All project protocols will be approved by Department of Health officials prior to implementation. The main purpose of intervention activities will be to ensure that children are receiving services that can help them thrive developmentally, and all of the potential services being considered have been tested previously in various environments and shown to be beneficial. In accordance with standard CHW protocols in South Africa, children identified as being in need of services that CHWs cannot provide will be referred to the appropriate primary health care clinic. With Department of Health oversight, all project CHWs will receive formal training in the administration of the final intervention package.

We consider risks for breach of confidentiality to be minimal. Baseline and endline survey activities at study households will be conducted by interviewers trained in the protection of human research participants. In addition, all intervention activities will be integrated into standard CHW household visit protocols, which already employ confidentiality protocols. To ensure that risks are minimized, we will employ the following efforts:

- During CHW training on the study intervention at the start of the project, we will provide a refresher training on the protection of human research participants; and
- All efforts will be made to maintain confidentiality by securely storing data from baseline and endline surveys in a safe, locked location.

Prior to the initiation of any project activities, we will seek a full review by the appropriate ethics oversight committee at the University of the Witwatersrand. In addition, all proposed activities will be reviewed by the appropriate Department of Health authorities.

**References**

1. Nsona H, Mtimuni A, Daelmans B, Callaghan-Koru JA, Gilroy K, Mgalula L, and Kachule T. (2012). Scaling up integrated community case management of childhood illness: update from Malawi. *American Journal of Tropical Medicine and Hygiene*, 87:54-60.
2. Bhutta ZA, Ahmed T, Black RE, Cousens S, Dewey K, Giugliani E, Haider BA, Kirkwood B, Morris SS, Sachdev HP, Shekar M, (2008). What works? Interventions for maternal and child undernutrition and survival. *Lancet*, 371:417-40.
3. Cooper P, Tomlinson M, Swartz L, Landman M, Molteno C, Stein A, and Murray L. (2009). Improving quality of mother-infant relationship and infant attachment in socioeconomically deprived community in South Africa: randomised controlled trial. *BMJ*, 338.
4. Walker SP, Chang SM, Powell CA, and Grantham-McGregor SM. (2005). Effects of early childhood psychosocial stimulation and nutritional supplementation on cognition and education in growth-stunted Jamaican children: prospective cohort study. *Lancet,* 366:1804-7.
5. Grantham-McGregor SM, Fernald LC, Kagawa RM, and Walker SP. (2014). Effects of integrated child development and nutrition interventions on child development and nutritional status. *Annals of the New York Academy of Sciences*, 1308:11-32.
6. Rahman A, Malik A, Sikander S, Roberts C, and Creed F. (2008). Cognitive behaviour therapy-based intervention by community health workers for mothers with depression and their infants in rural Pakistan: a cluster-randomised controlled trial. *Lancet*, 372:902-9.
7. Surkan PJ, Kennedy CE, Hurley KM, and Black MM. (2011). Maternal depression and early childhood growth in developing countries: systematic review and meta-analysis. *Bulletin of the World Health Organization*, 89:607-15.
8. Statistics South Africa. Population Census, 2011. (2012). Pretoria: Statistics South Africa.
9. World Bank. (2015). World Development Indicators Database. Washington DC: World Bank.
10. UNICEF. (2015). State of the World’s Children.
11. Richter LM, Yach D, Cameron N, Griesel RD, and De Wet T. (1995). Enrolment into Birth to Ten (BTT): population and sample characteristics. Paediatr Perinat Epidemiol, 9:109–120.
12. Grantham-McGregor S, Cheung YB, Cueto S, Glewwe P, Richter L, Strupp B, and International Child Development Steering Group. (2007). Developmental potential in the first 5 years for children in developing countries. *Lancet*, 369:60-70.
13. Department of Basic Education. (2011), Report on the Annual National Assessments of 2011, Pretoria: Department of Basic Education, South Africa
14. Cooper P. (2012) Promoting early cognitive development in South Africa. In: Public Service Review: UK Science and Technology.
15. Walker SP, Wachs TD, Grantham-McGregor S, Black MM, Nelson CA, Huffman SL, and Richter L. (2011). Inequality in early childhood: risk and protective factors for early child development. *Lancet*, 387:1325-38.
16. Cooper P, Tomlinson M, Swartz L, Woolgar M, Murray L, and Molteno C. (1999). Post-partum depression and the mother-infant relationship in a South African peri-urban settlement. *British Journal of Psychiatry*, 175:554-8.
17. Avan B, Richter LM, Ramchandani PG et al. (2010). Maternal postnatal depression and children’s growth and behaviour during the early years of life: exploring the interaction between physical and mental health. *Arch Dis Child*, 95:690–5.
18. Parsons CE, Young KS, Rochat TJ, Kringelbach ML, and Stein A. (2012). Postnatal depression and its effects on child development: a review of evidence from low-and middle-income countries. *British Medical* ***b****ulletin*, 101:57.
19. Engle PL, Fernald LC, Alderman H, Behrman J, O'Gara C, Yousafzai A, and Global Child Development Steering Group. (2011). Strategies for reducing inequalities and improving developmental outcomes for young children in low-income and middle-income countries. *Lancet*, 378:1339-1353.
20. Atmore, E. (2013). Early childhood development in South Africa–progress since the end of apartheid. *International Journal of Early Years Education*, 21:152-162.
21. Storbeck C, and Moodley S. (2011). ECD policies in South Africa–What about children with disabilities. *Journal of African Studies and Development*, 3:1-8.
22. Department of Basic Education. (2015). *The South African National Curriculum Framework for children from Birth to Four.* Pretoria: Department of Basic Education.
23. Neudorf K, Thurman TR, and Taylor TM. (2011). [*A Case Study: Interventions Promoting Early Childhood Development in South Africa*](http://hvc-tulane.org/downloads/ECD-case-study-low-res.pdf). New Orleans, LA: Tulane University School of Public Health and Tropical Medicine, Department of International Health and Development.
24. Nores M, and Barnett WS. (2010). Benefits of early childhood interventions across the world:(Under) Investing in the very young. *Economics of Education Review*, 29:271-282.
25. Baker-Henningham H, Powell C, Walker S, and Grantham-McGregor S. (2005). The effect of early stimulation on maternal depression: a cluster randomised controlled trial. *Archives of Disease in Childhood*, 90:1230-4.
26. Yousefzai AK, Rasheed MA, Rizvi A, Armstrong R, and Bhutta ZA. (2014). Effect of integrated responsive stimulation and nutrition interventions in the Lady Health Worker programme in Pakistan on child development, growth, and health outcomes: a cluster-randomised factorial effectiveness trial. *Lancet*, 384:1282-93.
27. WHO and UNICEF. (2012). Care for Child Development: Improving the care of young children (intervention package). Geneva: World Health Organization; and New York: UNICEF.
