# Supplementary material for: Evaluation of a community health worker home visit intervention to improve child development in South Africa: A cluster-randomized controlled trial
Source: PLoS Med. 2023 Apr 14;20(4):e1004222. doi: 10.1371/journal.pmed.1004222 (PMC10146459; doi:10.1371/journal.pmed.1004222)
Supplement: S2 Text — (DOCX) [file pmed.1004222.s003.docx]

**S2 Text. Additional detail on lab assessment procedures**

EEG and eye-tracking assessments were conducted at a lab that was established prior to the start of the study in the town of Tzaneen, which is centrally located within the study area. Lab facilities included two assessment rooms, one waiting room for study participants, one preparation room where supplies were stored and pre- and post-assessment procedures were implemented, and one office room where study staff worked when not conducting assessments. Each assessment room had one 32-channel portable Geodesic EEG System 400 (by EGI/Philips Neuro, Eugene, OR) and one Tobii Pro x3-120 120 Hz eye tracker (by Tobii Technology, Stockholm, Sweden). A standing partition was set up in each assessment room that cordoned off a small 5’ x 8’ assessment area in which the eye-tracking unit and a computer monitor were installed on a small desk. All other equipment, including the EEG amplifier and a laptop computer, were set up on a desk on the other side of the partition, where the assessors sat while monitoring assessments. During assessments, Hydrocel GSN 130 sensor nets were placed on the child’s head and attached to the EEG amplifier arm, which reached around the partition. Members of the investigator team with appropriate expertise in EEG (AT) and eye tracking (JL) set up the assessment rooms prior to the start of the study and extensively trained a team of permanent staff on the use of both technologies prior to the first assessment wave and again before each subsequent assessment wave. Assessors did not have previous medical training nor prior experience with the technologies. To ensure the quality of EEG data, the assessment rooms were confirmed to have low electrical noise using an electromagnetic meter. The average time to complete both assessments, including preparation activities and data collection, was around 45 minutes per child.

When conducting EEG assessments, the assessors first measured the child’s head circumference using a tape measure, to determine what size of sensor net would fit the child’s head. The sensor net is a stretchy cap made of soft sponges attached to electrodes, which each record brain activity from a different area of the child’s scalp. Each sensor is attached to a wire to record the electrical activity. After determining head circumference, the sensor net was soaked for 10 minutes in a warm potassium chloride solution to facilitate electrical conductance with the scalp. Caregivers were seated on a chair on the assessment side of the partition and children were seated on their caregiver’s lap. The assessor then entertained the child and provided them with toys while the EEG sensor net was placed on the child’s head and secured with a chin strap. The assessor made sure each sensor had a good connection with the scalp, and, when necessary, applied more potassium chloride solution with an eyedropper and carefully moved hair out of the way of the sensors. Caregivers were asked to remove all braids in the children’s hair prior to attending the lab visit to ensure a good connection could be obtained. Once impedances below 50 ohms were established for all sensors, the EEG recorded from all channels with reference to the vertex while the child sat on the caregiver’s lap with the lights dimmed. The assessor and caregiver did not speak during the recording. When necessary to keep the child calm during the recording, the assessor showed the child a toy. EEG data were recorded for 6 minutes at 250 or 1,000 Hz at the first assessment wave and at 500 Hz at the second and third waves, and all data was later resampled to 250 Hz. After recording was complete, the sensor net was removed, rinsed, and disinfected. Data were uploaded daily from lab computers to a secure file sharing platform. A member of the investigator team (AT) regularly checked data quality and provided feedback to the lab team.

Pupil-corneal reflection (P-CR) eye-tracking technologies have been used extensively in prior studies with infants and young children [1,2], including in LMIC contexts [3]. P-CR eye trackers are video-based methods that record information about gaze direction based on the coordinates of the pupil and the corneal reflection of an infrared light source in the camera image [4]. Previous studies have confirmed that the expected temporal (mean error ±4 msec) and empirically verified spatial (mean calibration error 0.7-1.1°) accuracy of eye-tracking with children is sufficient to reliably quantify SRT [5].

The eye-tracking assessment began just after the EEG assessment was complete. The caregiver remained seated and holding the child on their lap. The caregiver was positioned so that the child’s eyes were facing forward and at approximately 60cm viewing distance from the eye tracker and computer monitor. The caregiver was instructed to turn their head and eyes to the side (~90° from the screen) and to avoid looking at the screen during the assessment. During the assessment, the child was presented with short, alternating blocks of visual stimuli on the monitor that were designed to calibrate the eye tracking system, measure SRTs and fixations to social scenes. A detailed description of the eye tracking assessment is provided elsewhere [5]. Calibration targets were isoluminant white discs (1.3°×1.3°) presented in the corners of the screen. Calibration of the raw point-of-gaze estimates was performed using a similarity transformation estimator (https://github.com/axelpale/nudged) on samples that fell within a 9°×9° rectangle surrounding the white discs. Saccade targets were colorful cartoon animations of common objects (e.g., fish, bird, pig, rabbit, or a human face), subtending a 5.7° x 5.7° visual angle. Discernible colorful animations and a novel target for each presentation instead of more uniform visual stimuli were used based on children’s known proclivity for novelty and on evidence showing superior response rates to colorful, pictorial stimuli in young children [6]. The first target appeared in the center of the screen (0°,0°), and each subsequent target appeared in a new randomly chosen location on the screen 10° away from the previous target. After the initial stimulus in the center of the screen (not analyzed), the child saw a total of five animations in one block, and a total of 8 blocks to cumulate enough saccades to reliably estimate mean SRT. Videos of social scenes were presented in between SRT blocks.

The assessor monitored the eye-tracking assessment from the other side of the standing partition to ensure a controlled visual environment for the assessment. In instances when the system lost contact with the child’s eyes or the child became inattentive, the assessor paused the test and performed required adjustments or administered a break so that the caregiver could soothe the child. Eye-tracking assessments lasted approximately 10 minutes. Data were uploaded daily from lab computers to a secure file sharing platform. A member of the investigator team (JL) regularly checked data quality and provided feedback to the lab team.

**References**

1. Kulke L, Atkinson J, Braddick O. Automatic detection of attention shifts in infancy: eye tracking in the fixation shift paradigm. PLoS One. 2015;12*:* e0142505.
2. Jones PR, Kalwarowsky S, Atkinson J, Braddick OJ, Nardini M. Automated measurement of resolution acuity in infants using remote eye-tracking. Investigative Ophthalmology & Visual Science*.* 2014;12*:* 8102-10.
3. Forssman L, Ashorn P, Ashorn U, Maleta K, Matchado A, Kortekangas E, et al. Eye-tracking-based assessment of cognitive function in low-resource settings. Archives of Disease in Childhood. 2016;102: 301-2.
4. Holmqvist K, Örbom SL, Hooge IT, Niehorster DC, Alexander RG, Andersson R, et al. Eye tracking: empirical foundations for a minimal reporting guideline. Behavior Research Methods. 2022;1-53.
5. Leppänen JM, Butcher JW, Godbout C, Stephenson K, Hendrixson DT, Griswold S, et al. Assessing infant cognition in field settings using eye-tracking: a pilot cohort trial in Sierra Leone. BMJ Open. 2022;12: e049783.
6. Irving EL, González EG, Lillakas L, Wareham J, McCarthy T. Effect of stimulus type on the eye movements of children. Investigative Ophthalmology & Visual Science. 2011;2: 658-664.
