# Supplementary material for: Evaluation of a community health worker home visit intervention to improve child development in South Africa: A cluster-randomized controlled trial
Source: PLoS Med. 2023 Apr 14;20(4):e1004222. doi: 10.1371/journal.pmed.1004222 (PMC10146459; doi:10.1371/journal.pmed.1004222)
Supplement: S1 Table — (DOCX) [file pmed.1004222.s004.docx]

#### **S1 Table. Template for intervention description and replication (TIDieR) Checklist**

|  | **BRIEF NAME** |
| --- | --- |
| **1.** | CHW Home Visit Training and Job Aid for Early Childhood Development |
|  | **WHY** |
| **2.** | Regular home visits by trained staff who support and counsel caregivers on a diversity of topics, including child health, nutrition, and play-based stimulation, have been shown to be effective in several LMICs. |
|  | **WHAT** |
| **3.** | CHWs were provided with and trained on a job aid that included content on child health, nutrition, developmental milestones, and encouragement to engage in developmentally appropriate play-based activities. They were instructed to use the job aid to structure and guide the content of their regular monthly home visits with children under two years of age and their caregivers. The job aid included 24 pages, one for each month from birth to age two. The pages for the first six months of the child’s life included additional content focused on maternal well-being. During training, CHWs were instructed to flip to the page that matched the age of the child they were visiting. The job aid was designed so that the caregiver and child could view an outward facing page that included simple messages in three local languages (English, Sepedi, and Xitsonga) and corresponding illustrations of visit content. On the CHW-facing side of the job aid was a flowchart with the same content but with more detail that guided the CHW through the visit. The content of the job aid was also summarized in a small poster that was provided to each household for caregivers to keep and refer to throughout the course of the intervention period.  Two versions of the job aid were developed that differed only with respect to the suggested play-based activities and the approach to how caregivers interacted with their child during play. The first version emphasized caregiver-directed interactions focused on developing and reinforcing skills within the child’s zone of proximal development. For example, on the month-12 page of this version, the suggested activities included “take the child for walk and talk to the child about nature.” The second version encouraged caregiver-child interactions focused on strengthening responsiveness. On the month-12 page of this version, the suggested activities included “respond to child’s attempts to talk with a smile and a hug.” During home visits, CHWs encouraged mothers to try the activities described in the job aid and then provided feedback. |
| **4.** | The job aid was designed to be effort neutral by restructuring the already allotted time CHWs spent at homes. CHWs were trained on the job aid prior to the start of the intervention period and were provided with refresher trainings every 6 months thereafter. Each training also included an overview of key topics related to child development, a subject that was not part of standard CHW training in South Africa at the time of the study. |
|  | **WHO PROVIDED** |
| **5.** | The intervention was delivered by CHWs who were operating within Ward Based Outreach Teams (WBOTs) and paid by the government of South Africa. CHWs were trained on the job aid prior to the start of the intervention period and were provided with refresher trainings every 6 months thereafter. |
|  | **HOW** |
| **6.** | The intervention was delivered to caregivers face-to-face during monthly home visits that lasted around 30 minutes on average. |
|  | **WHERE** |
| **7.** | The intervention was delivered at the homes of recipients. |
|  | **WHEN and HOW MUCH** |
| **8.** | The intervention was designed to be delivered one per month over a 24-month intervention period, from the child’s birth until they turned two years old. Home visits were 30 minutes in duration. |
|  | **TAILORING** |
| **9.** | Two versions of the job aid were developed that differed only with respect to the suggested play-based activities and the approach to how caregivers interacted with their child during play. The first version emphasized caregiver-directed interactions focused on developing and reinforcing skills within the child’s zone of proximal development. For example, on the month-12 page of this version, the suggested activities included “take the child for walk and talk to the child about nature.” The second version encouraged caregiver-child interactions focused on strengthening responsiveness. On the month-12 page of this version, the suggested activities included “respond to child’s attempts to talk with a smile and a hug.” |
|  | **MODIFICATIONS** |
| **10.** | The intervention was not modified during the study. |
|  | **HOW WELL** |
| **11.** | We assessed CHW adherence to the intervention using data collected during a set of phone-based interviews we conducted with one randomly selected caregiver served by each CHW after the intervention had been running for one year. We intended to capture additional evidence of adherence at endline based on caregiver reporting, but because of the pause in the study due to COVID-19, the intervention had been completed for one year when we eventually conducted endline, making the recall period too long to generate quality data. We did not intervene to improve adherence based on collected data. |
| **12.** | Based on phone-based surveillance data collected after the first year of the intervention period, 43% of CHWs were determined to be compliant with monthly home visits, while 64% had made a home visit in the last 3 months. |
