# Supplementary material for: Evaluation of a community health worker home visit intervention to improve child development in South Africa: A cluster-randomized controlled trial
Source: PLoS Med. 2023 Apr 14;20(4):e1004222. doi: 10.1371/journal.pmed.1004222 (PMC10146459; doi:10.1371/journal.pmed.1004222)
Supplement: S3 Table — (DOCX) [file pmed.1004222.s006.docx]

**S3 Table. Characteristics of EEG and SRT observations**

|  | **EEG power** | |  | **SRT** | |
| --- | --- | --- | --- | --- | --- |
|  | **Included**  **(n=837)** | **Excluded**  **(n=79)** |  | **Included**  **(n=711)** | **Excluded**  **(n=205)** |
| Child older siblings | 1.30  (1.17) | 1.43  (1.15) |  | 1.33  (1.18) | 1.25  (1.13) |
| Caregiver age at birth (years) | 31.69  (9.81) | 33.40  (12.38) |  | 32.09  (10.03) | 30.95  (10.13) |
| Caregiver education (years) | 10.35  (2.37) | 10.03  (2.78) |  | 10.33  (2.41) | 10.27  (2.42) |
| Household wealth (z-score) | 0.20  (1.00) | 0.18  (1.01) |  | 0.21  (1.02) | 0.13  (0.93) |
| Household receives  Child Support Grant | 0.31  (0.46) | 0.27  (0.44) |  | 0.30  (0.46) | 0.33  (0.47) |

*Notes:* all values are mean (SD)
