# Supplementary material for: Evaluation of a community health worker home visit intervention to improve child development in South Africa: A cluster-randomized controlled trial
Source: PLoS Med. 2023 Apr 14;20(4):e1004222. doi: 10.1371/journal.pmed.1004222 (PMC10146459; doi:10.1371/journal.pmed.1004222)
Supplement: S4 Table — (DOCX) [file pmed.1004222.s007.docx]

**S4 Table. Impact on MDAT z-scores in lab subsample**

|  | **Adjusted^a^** | |
| --- | --- | --- |
|  | **β (95% CI)** | **p value** |
| MDAT z-scores^b^ |  |  |
| Gross motor | 0.04 (-0.23, 0.31) | 0.791 |
| Fine motor | -0.06 (-0.28, 0.17) | 0.623 |
| Language | -0.06 (-0.32, 0.20) | 0.668 |
| Social-emotional | 0.07 (-0.23, 0.37) | 0.633 |

^a^Includes covariates included in the randomization procedure and the following controls: child age, sex and number of siblings; household wealth and receipt of Child Support Grant; and caregiver age and education.

^b^Models include additional covariates indicating location of MDAT assessment (home or lab) and assessor fixed effects
