# Supplementary material for: Evaluation of a community health worker home visit intervention to improve child development in South Africa: A cluster-randomized controlled trial
Source: PLoS Med. 2023 Apr 14;20(4):e1004222. doi: 10.1371/journal.pmed.1004222 (PMC10146459; doi:10.1371/journal.pmed.1004222)
Supplement: S1 Fig — (PDF) [file pmed.1004222.s008.pdf]

S
E
T

Child Activity

- Bolela le go myemyela le lesea
- Dira go kgomana ga letlalo lago le la ngwana
- Phopola le go swara ngwana

- Talk to and smile at baby
- Practice skin-to-skin with baby
- Soothe, stroke, and hold baby

- Valavula ni ku nwayitela eka n'wana
- Endla ku khumbhana na n'wana
- Bambatela, ni ku vukarha n'wana

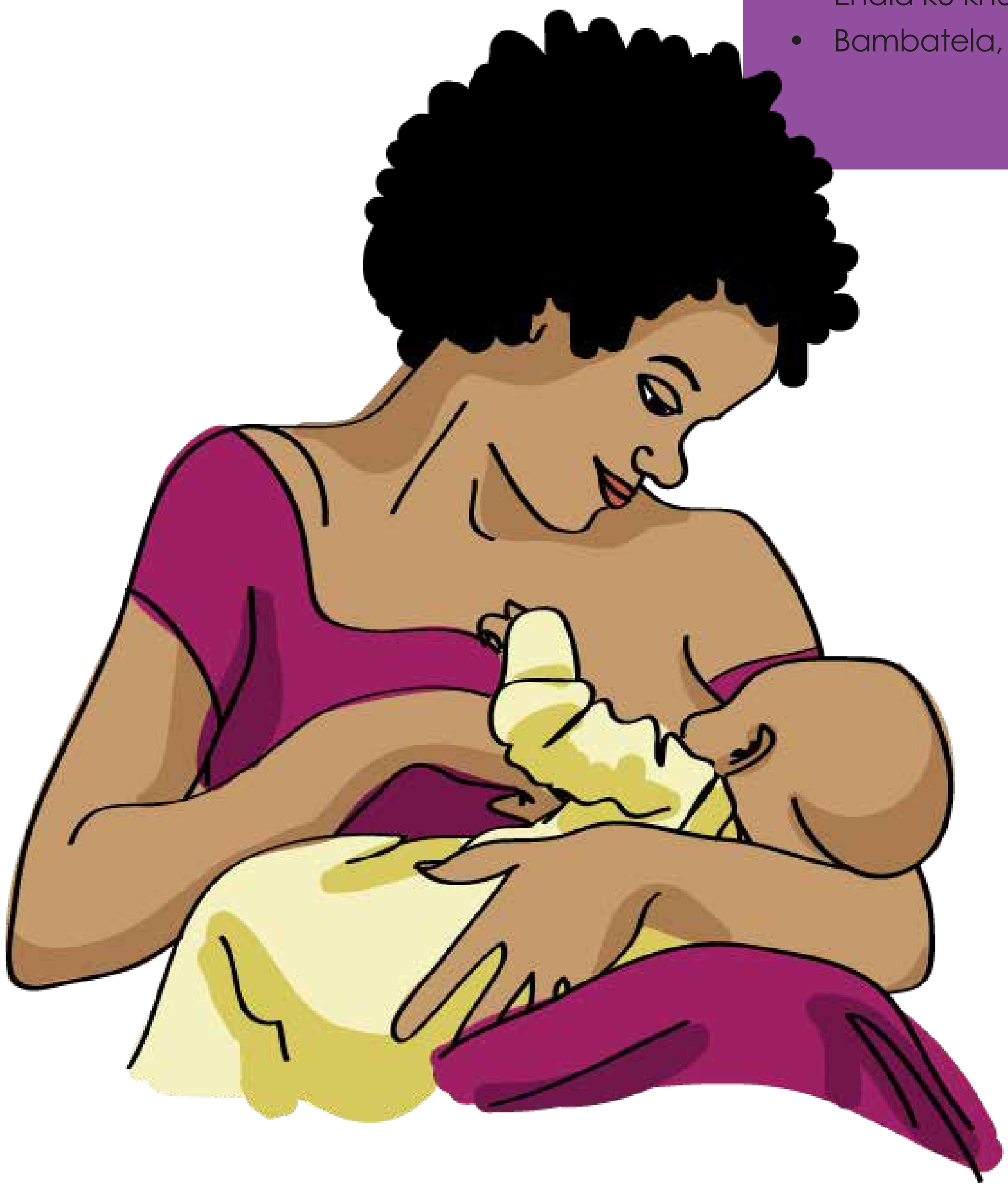

Child Health

- Ditšhwaana tša thibela malwetši di šireletša ngwana
- Nyaka thušo ka potlako ge lesea le lwala

- Vaccines protect baby
- Seek care immediately if baby is sick

- Misawutiso yi sirhelela n'wana
- Lava ku pfuniwa hi xihaltla loko n'wana a vabya

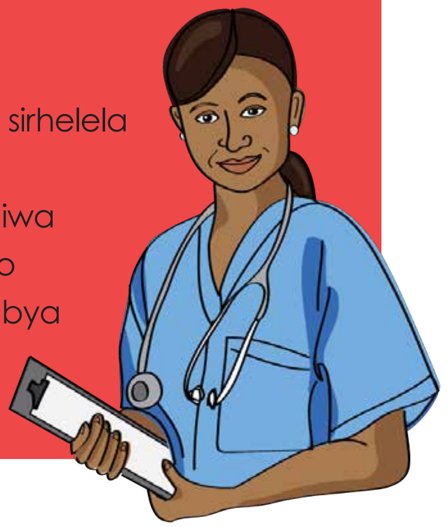

Child Nutrition

- Go mamisa go tshireletsa ngwana malwetsi

- Breastfeeding protects baby against disease

- Ku mamisa vele swi sirhelela n'wana eka mavabyi

Child Milestones

- Lesea le swanetše go hwetša dijo go mma

- Baby should feed from mom

- N'wana u fanele ku mama eka manana

Maternal Wellbeing

- Gantši bomma ba bafsa ba na le go ikwa ba le fasenyana

- New moms often feel 'blue'

- Mikarhi yin'wana vamhani lavantshwa va titwa va 'tsanile'

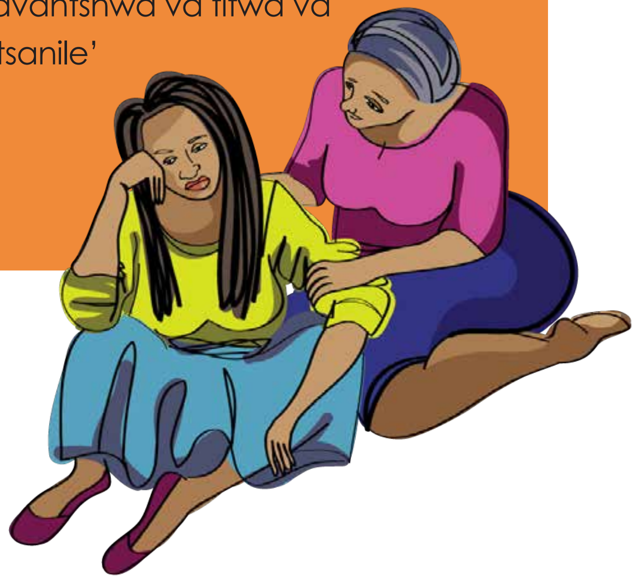

Start

Greet parent and explain purpose of visit.

Ask how parent and child are doing?

Ask mother/family if she has any concerns (e.g. child is sick, appears slower to develop)?

Maternal Wellbeing

- It is normal for new moms to feel 'blue' the first month or two after birth. This is partly due to changes with her body and will likely go away soon. If mom is feeling very 'blue' explain local resources that might help and encourage her to use them.

Child Health

- At birth, babies should receive BCG and OPV0. Confirm that the baby has received these vaccines. If not yet received, encourage mom to bring baby to the clinic for these vaccines immediately.
- Postnatal care in first days after birth is important to make sure baby is gaining weight and umbilical cord isn't infected.

Child Nutrition

- Assist mother with learning breastfeeding and encourage exclusive breastfeeding.

Child Milestones

- It is very normal for baby to sleep most of day, waking only to feed. Mom should observe normal breathing

Child Activity

- Baby can hear mom talking from birth. Talking to baby from first day will help develop connection with mom, and stimulate development. Encourage mom to talk to baby every day.
- Skin-to-skin helps to develop connection between baby and mom. Demonstrate for mom how to lay baby on her chest and practice skin-to-skin. Encourage mom to do skin-to-skin often.
- Baby needs mom's comfort to deal with new experiences in the world. Demonstrate for mom how to hold and soothe baby when she is upset.

Observe parent and child trying activity. If needed demonstrate again to help caregiver

Ask if the caregiver has any questions or concerns?
Arrange next visit date

Thank mother and family. Encourage mother to try implement the advice and activities.

Note any questions asked, action needed, referral required, or things to discuss with supervisor/ team leader

End

Maternal Wellbeing

- Gantši bomma ba bafsa ba na le go ikwa ba le fasenyana

- New moms often feel ‘blue’

- Mikarhi yin’wana vamhani lavant-shwa va titwa va ‘tsanile’

Child Milestones

- Lesea le ka dula le phafogile leb-akanyana le letelele
- Lesea le ka myemyela

- Baby might be awake for longer periods
- Baby might smile

- N’wana a nge he etleli mikarhi yo leha
- N’wana a nga n’wayitela

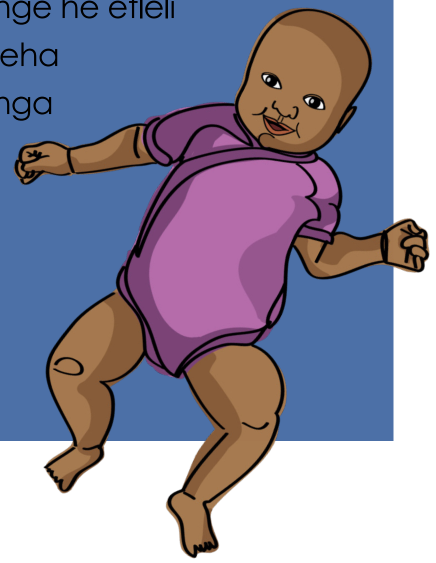

Child Health

- Hlapa diatla pele o swara lesea
- Nyaka thušo ka potlako ge lesea le lwala

- Wash hands before holding baby
- Seek care immediately if baby is sick

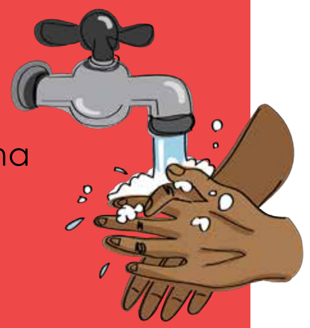

- Hlamba swandla u nga si khoma n’wana
- Lava ku pfuniwa hi xihatla loko n’wana a vabya

Child Nutrition

- Maswi a mma a na le tšohle tše di hlokwago ke lesea
- Meetse le dijo di ka ba kotsi go lesea

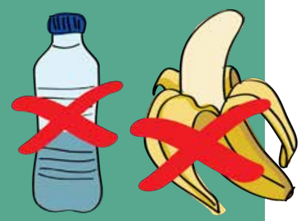

- Mom’s milk has all baby needs
- Water and foods can be dangerous for baby

- Mafi ya manana ya na hinkwaswo leswi n’wana a swi lavaka
- Mati na swakudya swi nga va na khombo eka n’wana

Child Activity

- Iebelela ka matlhong ya ngwa-na o myemyele
- Dira difatlhego tše segišago go dira ngwana gore a sege
- Binela ngwana dikoša tšatši ka tšatši

- Look into baby’s eyes and smile
- Make funny faces to make baby laugh
- Sing songs to baby every day

- Languta matihlo ya n’wana u n’wayitela
- Endla xikandza xi hlekisa ku endlela nwana a hleka
- Yimbelela n’wana tinsimu masiku hinkwawo

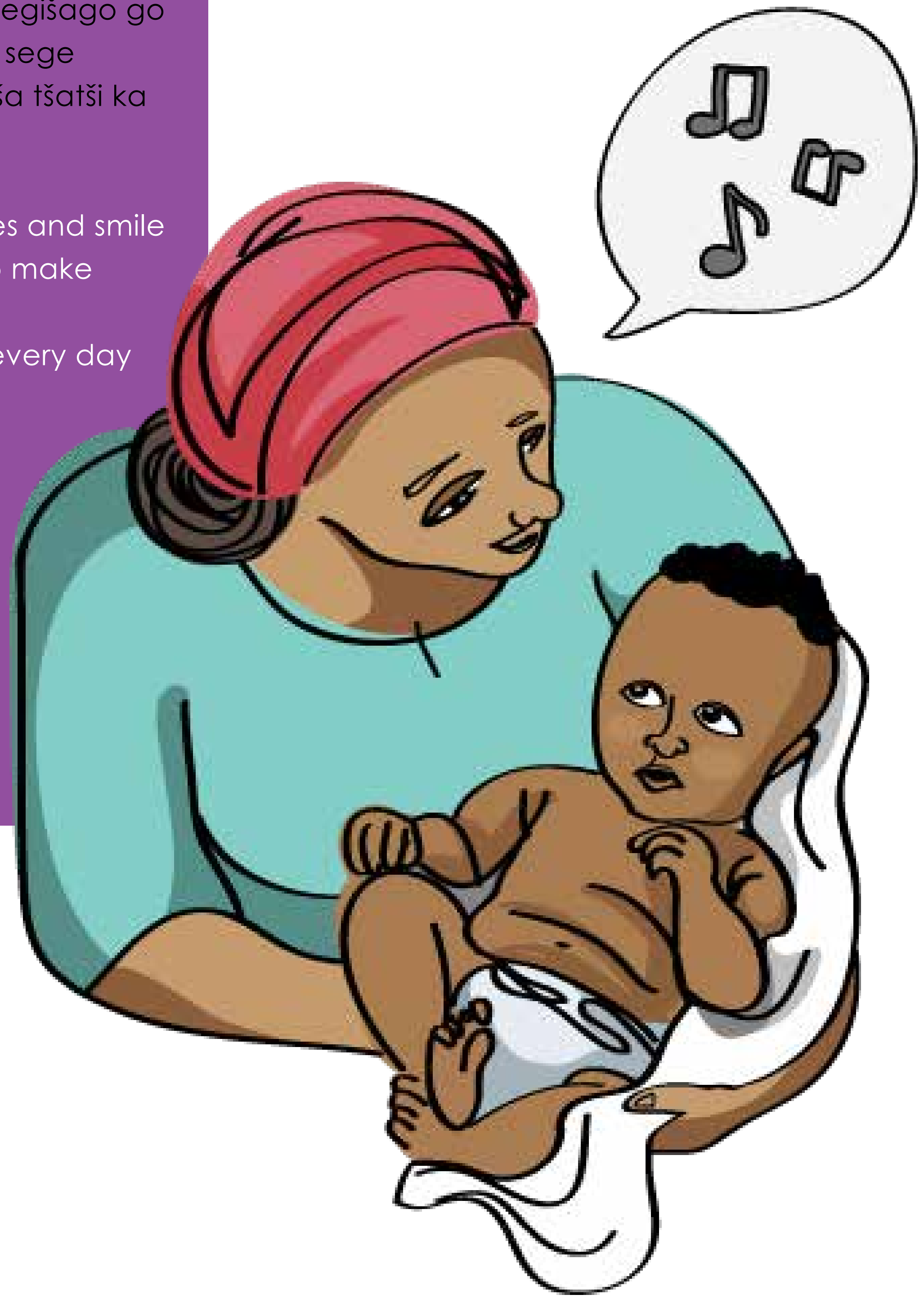

S E T

Start

Greet parent and explain purpose of visit.

Ask how parent and child are doing?

Ask caregiver if they have been trying any of the activities since last visit?

Ask if parent can show one of the activities?

Ask if mother/family has any difficulties/challenges in implementing the advice or activities?

Ask if mother/family observed any benefits in implementing the advice or activities?

Ask mother/family if she has any concerns (e.g. child is sick, appears slower to develop)?

Maternal Wellbeing

- It is normal for new moms to feel 'blue' the first month or two after birth. This is partly due to changes with her body and will likely go away soon. If mom is feeling very 'blue' explain local resources that might help and encourage her to use them.

Child Health

- Good hygiene by parents is important for baby. Encourage all caregivers in household to wash hands before touching baby, particularly after using toilet or changing baby.
- If baby has fever, cough, or is not feeding, they should be brought to the clinic immediately. Describe how to check for symptoms. Encourage immediate care seeking if symptoms found.

Child Nutrition

- Continue to encourage exclusive breastfeeding. Describe to mom how it has all of the nutrition that the baby needs at this age.
- Water and other foods can be dangerous for baby because they might have bacteria that cause illness and baby's digestive system is not developed.

Child Milestones

- As baby grows, she will start to be awake for longer periods of time.
- Baby might start smiling at this age.

Child Activity

- Baby is learning to recognize mom's face and how to interpret facial expressions. Encourage mom to look into baby's eyes and smile at baby as much as possible.
- Encourage mom to make funny faces for baby to help build a positive relationship
- Songs will help to soothe and calm baby. Encourage mom to sing songs to baby every day.

Observe parent and child trying activity. If needed demonstrate again to help caregiver

Ask if the caregiver has any questions or concerns?
Arrange next visit date

Thank mother and family. Encourage mother to try implement the advice and activities.

Note any questions asked, action needed, referral required, or things to discuss with supervisor/team leader

End

S
E
T

Child Activity

- Swara ngwana omo dumelele go go swara sefatlhego
- Kopisa modumo wa ngwana
- Binela ngwana tšatši ka tšatši

- Hold baby and let baby touch your face
- Copy baby's noises
- Sing songs to baby every day

- Khoma nhloko ya n'wana u endla n'wana akhoma xikandza xa wena
- Kopela ku rhasa ka n'wana
- Yimbelela n'wana tinsimu masiku hinkwawo

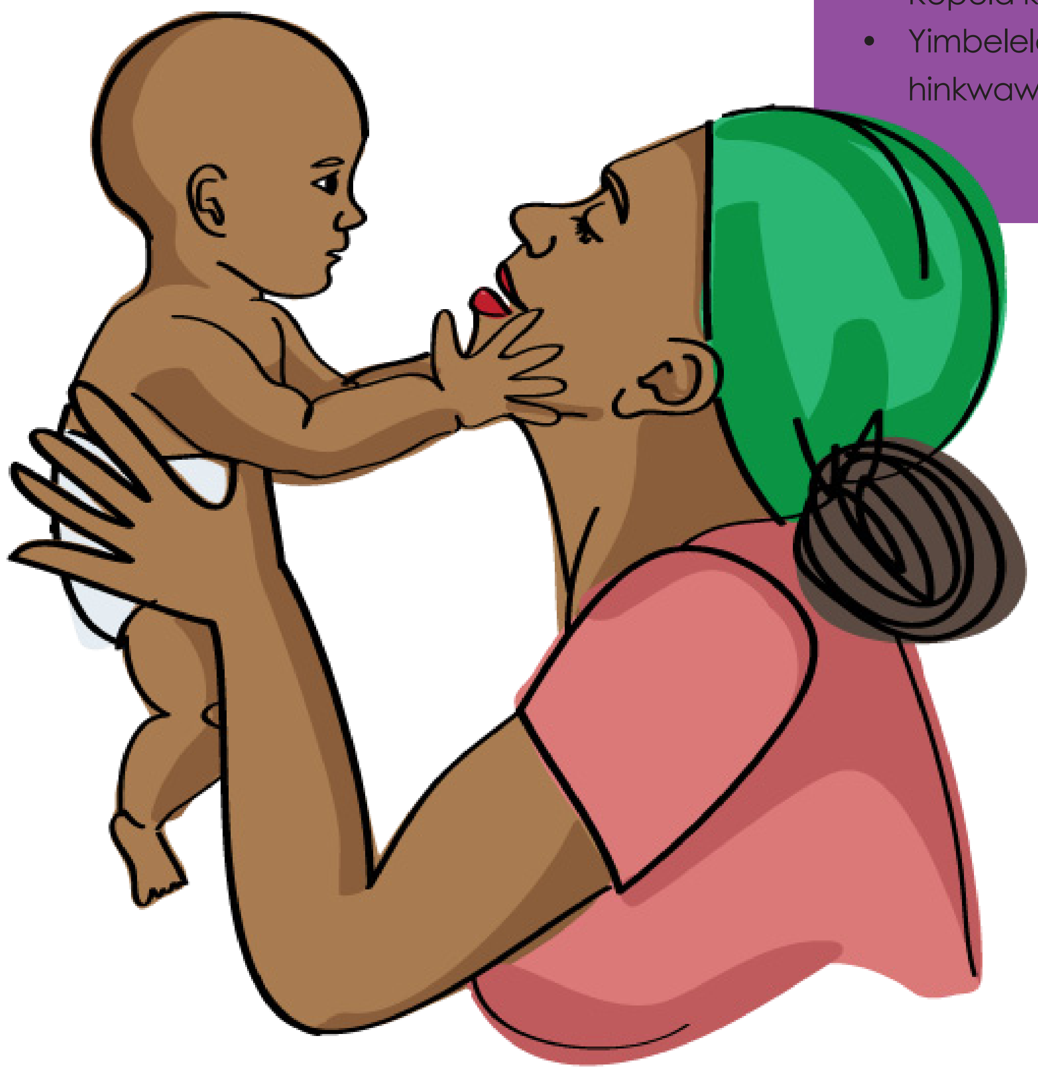

Child Milestones

- Lesea le ka dira medumo ya seb-jana
- Lesea le ka retola hlogo go ya ka modumo

- Baby might coo and make gur-gling sounds
- Baby might turn head toward sounds

- N'wana a nga endla mipfumawulo yo vulavula
- N'wana a nga hundzuluxa nhloko a languta laha mpfumawulo wu twakalaka kona

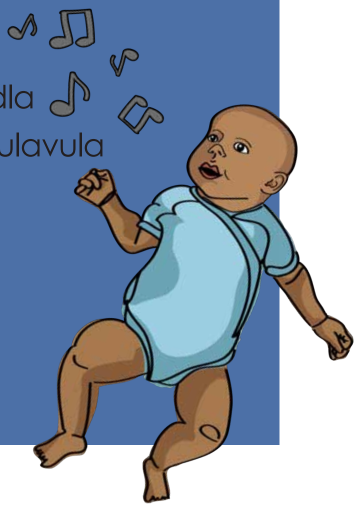

Maternal Wellbeing

- Thibela-pelegi e bohlokwa go tša maphelo a mma

- Family planning is important for mom's health

- Ku kunguhata mbeleko swi na nkoka eka rihanyu ra mhani

Child Health

- Ditšhwaana tša thibela malwetši di šireletša ngwana
- Nyaka thušo ka potlako ge lesea le lwala

- Vaccines protect baby
- Seek care immediately if baby is sick

- Misawutiso yi sirhelela n'wana
- Lava ku pfuniwa hi xihaltla loko n'wana a vabya

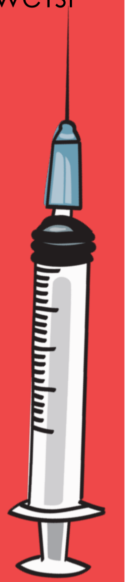

Child Nutrition

- Maswi a mma a na le tšohle tše di hlokwago ke lesea
- Meetse le dijo di ka ba kotsi go lesea

- Mom's milk has all baby needs
- Water and foods can be dangerous for baby

- Mafi ya manana ya na hinkwaswo leswi n'wana a swi lavaka
- Mati na swakudya swi nga va na khombo eka n'wana

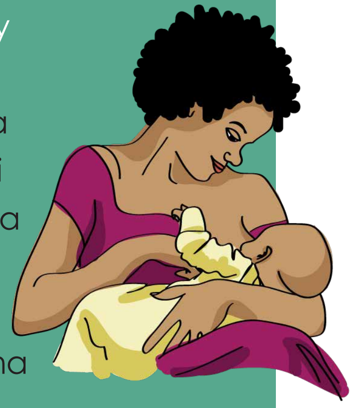

MONTH 2

Start

Greet parent and explain purpose of visit.

Ask how parent and child are doing?

Ask caregiver if they have been trying any of the activities since last visit?

Ask if parent can show one of the activities?

Ask if mother/family has any difficulties/challenges in implementing the advice or activities?

Ask if mother/family observed any benefits in implementing the advice or activities?

Ask mother/family if she has any concerns (e.g. child is sick, appears slower to develop)?

Maternal Wellbeing

- Explain to mom the family planning options available and encourage her to visit the clinic soon for a consultation.

Child Health

- At 6 weeks old, babies should receive OPV1, RV1, DTaP-IPV/Hib1, HBV1, and PCV1. Confirm that the baby has received these vaccines. If not yet received, encourage mom to bring baby to the clinic for these vaccines immediately.
- If baby has fever, cough, or is not feeding, they should be brought to the clinic immediately. Describe how to check for symptoms. Encourage immediate care seeking if symptoms found.

Child Nutrition

- Continue to encourage exclusive breastfeeding. Describe to mom how it has all of the nutrition that the baby needs at this age.
- Water and other foods can be dangerous for baby because they might have bacteria that cause illness and baby's digestive system is not developed.

Child Milestones

- Baby might coo and make gurgling sounds. Demonstrate sounds mom can listen for.
- Baby might turn head toward sounds. Demonstrate how mom can look for this.

Child Activity

- Demonstrate how mom can hold baby in front of her face so that baby can see her facial expressions clearly. Encourage mom to have baby touch her face.
- As baby starts to make noises, mom can copy noises to start to build a "conversation" with baby. This type of "serve and return" teaches baby how to interact with the world.
- Songs will help to soothe and calm baby. Encourage mom to sing songs to baby every day.

Observe parent and child trying activity. If needed demonstrate again to help caregiver

Ask if the caregiver has any questions or concerns?
Arrange next visit date

Thank mother and family. Encourage mother to try implement the advice and activities.

Note any questions asked, action needed, referral required, or things to discuss with supervisor/team leader

End

S
E
T

Maternal Wellbeing

- Go amuša ga go thibele go ima

- Breastfeeding does not protect against pregnancy

- Ku mamisa vele a swi siveli vuyimani

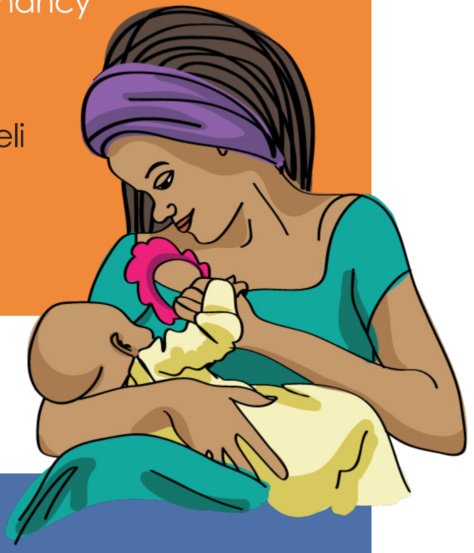

Child Milestones

- Lesea le ka latella dilo ka mahlo
- Lesea le ka emiša hlogo

- Baby might follow objects with eyes
- Baby might lift head

- N'wana a nga landzelela swilo hi mahlo
- N'wana a nga tlakusa nhloko

Child Health

- Ditšhwaana tša thibela malwetši di šireletša ngwana
- Nyaka thušo ka potlako ge lesea le lwala

- Vaccines protect baby
- Seek care immediately if baby is sick

- Misawutiso yi sirhelela n'wana
- Lava ku pfuniwa hi xihatla loko n'wana a vabya

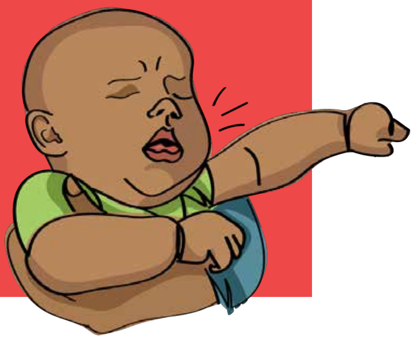

Child Nutrition

- Maswi a mma a na le tšohle tše di hlokwago ke lesea
- Meetse le dijo di ka ba kotsi go lesea

- Mom's milk has all baby needs
- Water and foods can be dangerous for baby

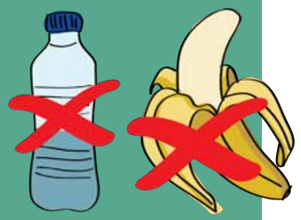

- Mafi ya manana ya na hinkwaswo leswi n'wana a swi lavaka
- Mati na swakudya swi nga va na khombo eka n'wana

Child Activity

- Kuka ngwana ge a lla o bolele le yena
- Dumelela papa go kuka ngwana le go bina
- Botja ngwana gore wa mo rata pele ga ge a robala

- Hold baby when baby is crying and talk to him/her
- Have dad hold baby and sing songs
- Tell baby you love him/her before going to sleep

- Khoma n'wana loko a rila na ku vulavula na yena
- Endla papa a khoma n'wana ni ku n'wu yimelela
- Byela n'wana ku ri wa n'wu rhandza u nga se ya tlela

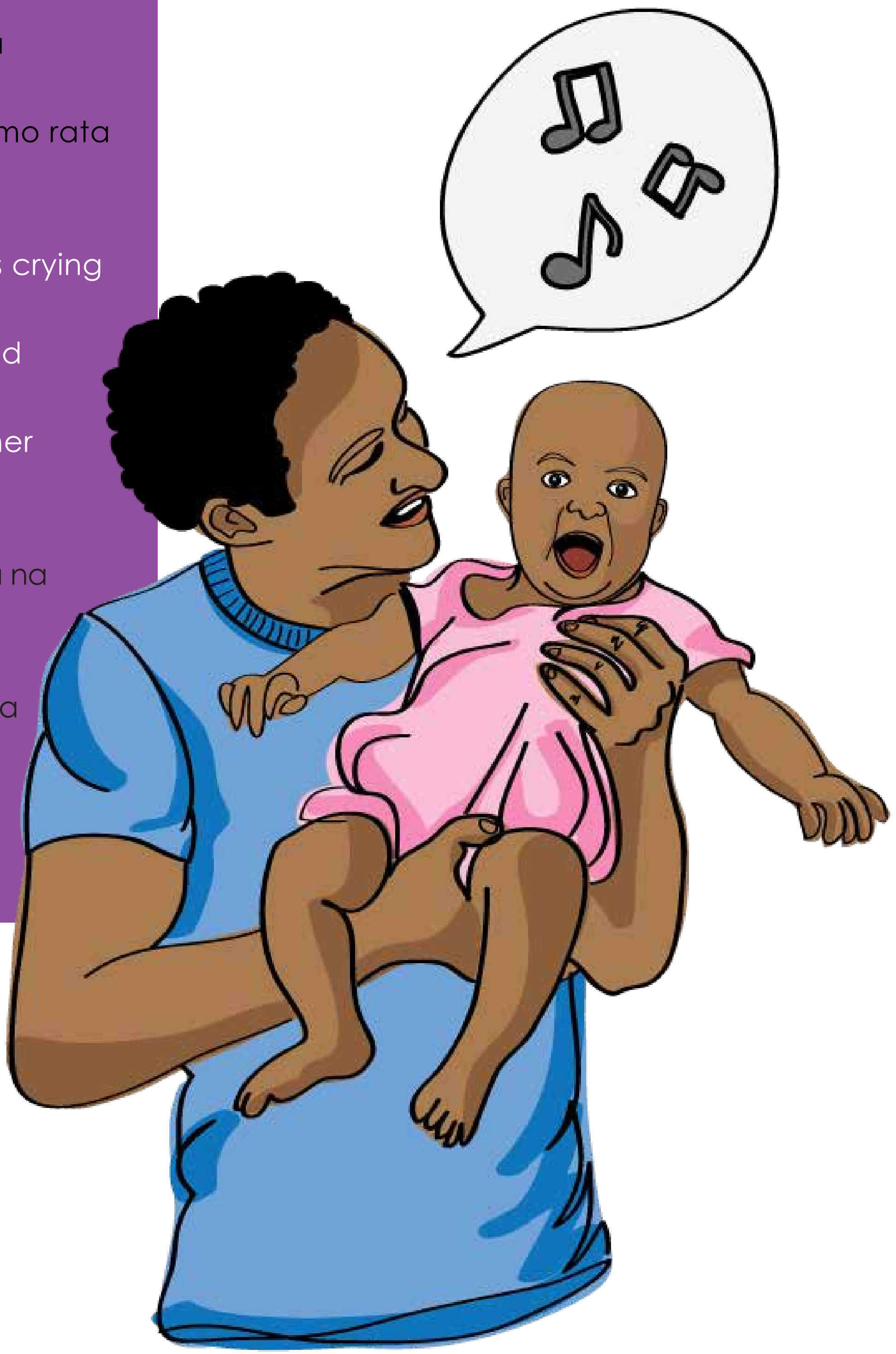

Start

Greet parent and explain purpose of visit.

Ask how parent and child are doing?

Ask caregiver if they have been trying any of the activities since last visit?

Ask if parent can show one of the activities?

Ask if mother/family has any difficulties/challenges in implementing the advice or activities?

Ask if mother/family observed any benefits in implementing the advice or activities?

Ask mother/family if she has any concerns (e.g. child is sick, appears slower to develop)?

Maternal Wellbeing

- Explain to mom the family planning options available and encourage her to visit the clinic soon for a consultation.

Child Health

- At 10 weeks old, babies should receive DTaP-IPV/Hib2, and HBV2. Confirm that the baby has received these vaccines. If not yet received, encourage mom to bring baby to the clinic for these vaccines immediately.
- If baby has fever, cough, or is not feeding, they should be brought to the clinic immediately. Describe how to check for symptoms. Encourage immediate care seeking if symptoms found.

Child Nutrition

- Continue to encourage exclusive breastfeeding. Describe to mom how it has all of the nutrition that the baby needs at this age.
- Water and other foods can be dangerous for baby because might have bacteria that cause illness and baby's digestive system is not developed.

Child Milestones

- Baby might follow objects with eyes. Demonstrate how mom can look for this.
- Baby might lift head when being held against mom's shoulder. Demonstrate how mom can look for this.

Child Activity

- Demonstrate how mom can hold baby in front of her face so that baby can see her facial expressions clearly. Encourage mom to talk to baby while holding her like this.
- Explain to mom and dad how important it is that dad be involved and interact with the child every day. Encourage dad to hold baby and sing her songs.
- Encourage mom to Tell baby you love him/her" before going to sleep every day.

Observe parent and child trying activity. If needed demonstrate again to help caregiver

Ask if the caregiver has any questions or concerns?
Arrange next visit date

Thank mother and family. Encourage mother to try implement the advice and activities.

Note any questions asked, action needed, referral required, or things to discuss with supervisor/team leader

End

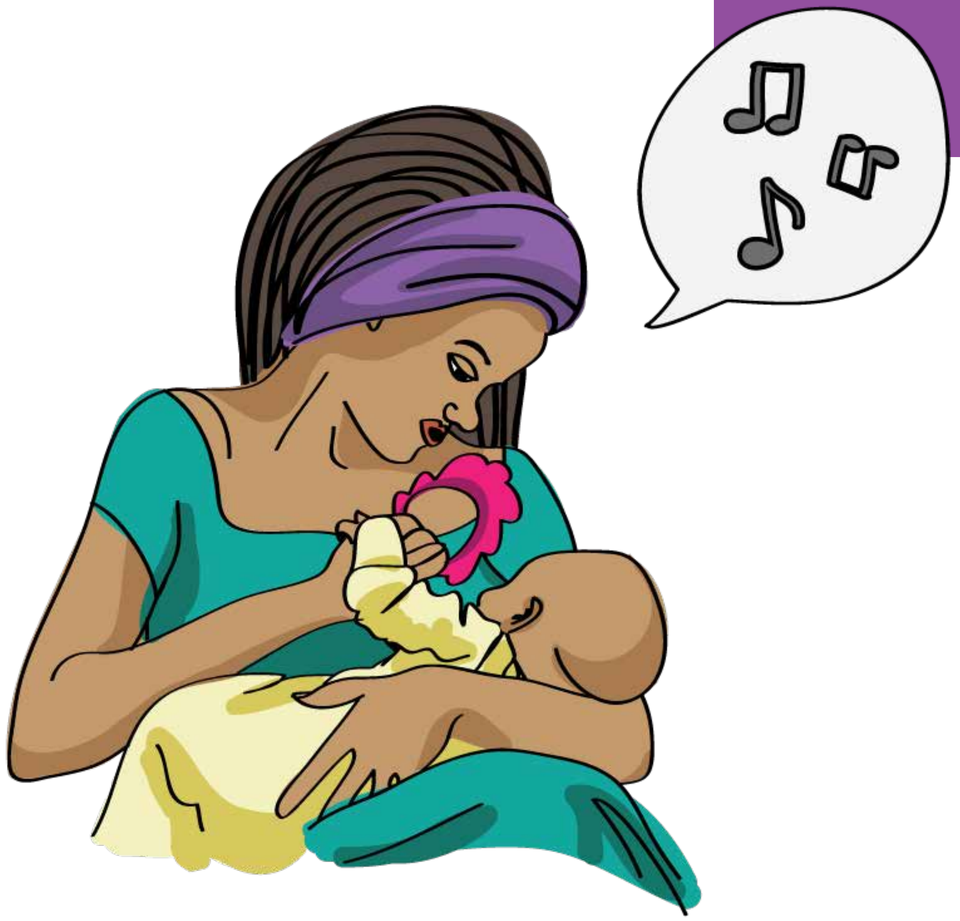

Child Activity

- Bolela le ngwana polelo ya bjana
- Akara ngwana o fetole seo ngwana a se dirang
- Opelela ngwana dikoša tšatši ka tšatši

- Talk baby language to baby
- Hug baby and respond to what baby does
- Sing songs to baby every day

- Valavula ma valavulelo ya n'wana eka n'wana
- Vukarha n'wana ni ku hlamula eka leswi n'wana a swi ndlaku
- Yimbelela n'wana tinsimu masiku hinkwawo

Child Milestones

- Lesea le ka swara dilo ka diatla
- Lesea le ka iphetola

- Baby might hold objects in hands
- Baby might roll over

- N'wana a nga khoma swilo hi mavoko
- N'wana a nga vumbuluka

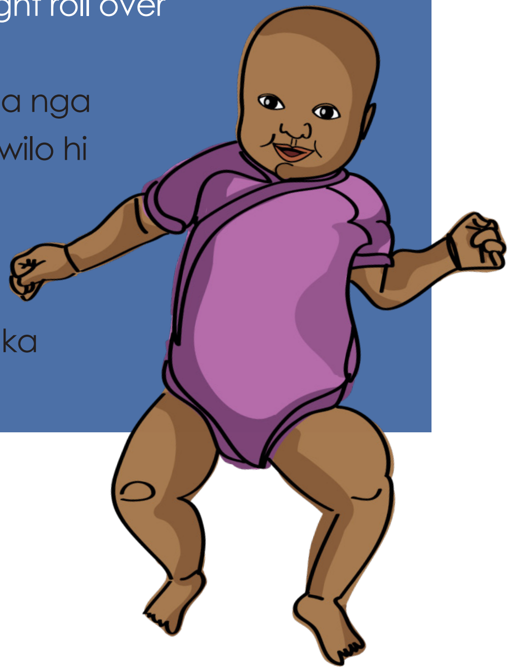

Maternal Wellbeing

- Thibela-pelegi e bohlokwa go tša maphelo a mma

- Family planning is important for mom's health

- Ku kunguhata mbeleko swi na nkoka eka rihanyu ra mhani

Child Health

- Ditšhwaana tša thibela malwetši di šireletša ngwana
- Nyaka thušo ka potlako ge lesea le lwala

- Vaccines protect baby
- Seek care immediately if baby is sick

- Misawutiso yi sirhelela n'wana
- Lava ku pfuniwa hi xihaltla loko n'wana a vabya

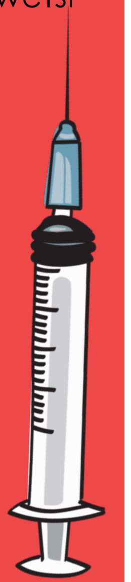

Child Nutrition

- Maswi a mma a na le tšohle tše di hlokwago ke lesea
- Meetse le dijo di ka ba kotsi go lesea

- Mom's milk has all baby needs
- Water and foods can be dangerous for baby

- Mafi ya manana ya na hinkwaswo leswi n'wana a swi lavaka
- Mati na swakudya swi nga va na khombo eka n'wana

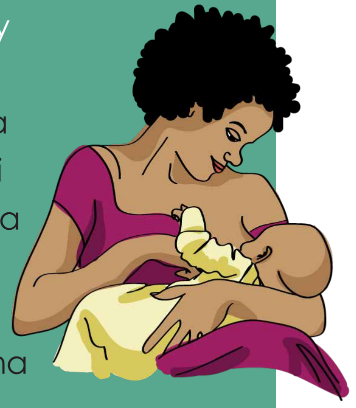

Start

Greet parent and explain purpose of visit.

Ask how parent and child are doing?

Ask caregiver if they have been trying any of the activities since last visit?

Ask if parent can show one of the activities?

Ask if mother/family has any difficulties/challenges in implementing the advice or activities?

Ask if mother/family observed any benefits in implementing the advice or activities?

Ask mother/family if she has any concerns (e.g. child is sick, appears slower to develop)?

Maternal Wellbeing

- Explain to mom the family planning options available and encourage her to visit the clinic soon for a consultation.

Child Health

- At 14 weeks old, babies should receive RV2, DTaP-IPV/Hib3, HBV3, and PCV2. Confirm that the baby has received these vaccines. If not yet received, encourage mom to bring baby to the clinic for these vaccines immediately.
- If baby has fever, cough, or is not feeding, they should be brought to the clinic immediately. Describe how to check for symptoms. Encourage immediate care seeking if symptoms found.

Child Nutrition

- Continue to encourage exclusive breastfeeding. Describe to mom how it has all of the nutrition that the baby needs at this age.
- Water and other foods can be dangerous for baby because they might have bacteria that cause illness and baby's digestive system is not developed.

Child Milestones

- Baby might hold objects in hands. Demonstrate how mom can look for this.
- Baby might roll over. Demonstrate how mom can look for this.

Child Activity

- Explain to mom that although baby is too young to understand meaning of words, it is still important to have "conversations" with baby. Encourage mom to use "baby language" with baby.
- Showing baby love with hugs and smiles will help baby deal with new challenges as they grow. Encourage mom to hug baby and give positive encourage when baby does new things.
- Songs will help to soothe and calm baby. Encourage mom to sing songs to baby every day.

Observe parent and child trying activity. If needed demonstrate again to help caregiver

Ask if the caregiver has any questions or concerns?
Arrange next visit date

Thank mother and family. Encourage mother to try implement the advice and activities.

Note any questions asked, action needed, referral required, or things to discuss with supervisor/team leader

End

S

E

T

Maternal Wellbeing

- Go hlola ga maphelo a mma
- Check-up on mom's health
- Nkambelo wa rihanyu ra manana

Child Health

- Nyaka thušo ka potlako ge lesea le lwala
- Tšwelapele ka go fepa gareng ga bolwetši
- Hlola kgolo ya lesea

- Seek care immediately if baby is sick
- Continue feeding during sickness
- Check baby's growth

- Lava ku pfuniwa hi xihatla loko n'wana a vabya
- Yana emahlweni na ku mamisa n'wana hambiloko a vabya
- Kamba ku kula ka n'wana

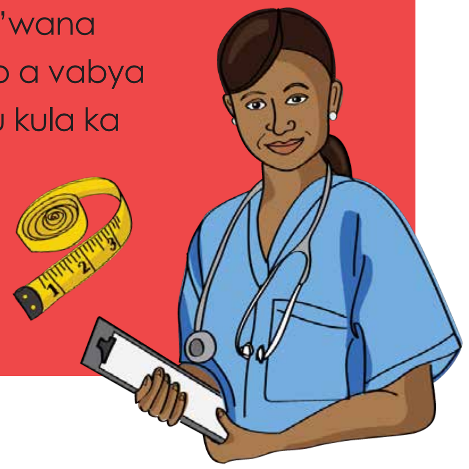

Child Milestones

- Lesea le ka fihlella dilo
- Lesea le ka lemoga ba leloko ba sa le kgojana

- Baby might reach for objects
- Baby might recognize family at a distance

- N'wana a nga fikelela swilo
- N'wana a nga tiva swirho swa ndyangu swi ri empfhukeni

Child Nutrition

- Ka go nanya thoma go fa lesea dijo tše nnyane tše bonolo
- Go tšwelela ka go amuša go loketše lesea

- Slowly introduce a few simple foods
- Continuing breastfeeding good for baby

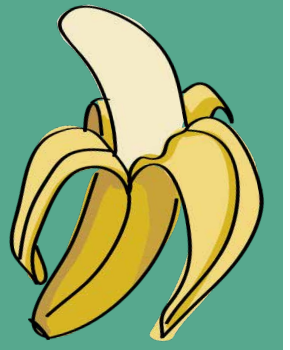

- Dyondzisa n'wana swakudya swintshwa hi switsongotsongo
- Yana emahlweni na ku mamisa hikuva swi kahle eka n'wana

Child Activity

- Leka go dira ngwana gore a meyemeyele ka matjatji.
- Reta ngwana ge a dira dilo tše diswa
- Opelela ngwana dikoša tšatši ka tšatši

- Try to make baby smile every day
- Praise baby when she does new things
- Sing songs to baby every day

- Ringeta ku endla n'wana a n'wayitela masiku hinkwawo
- Themendhela n'wana loko a endla swilo swi ntshwa
- Yimbelela n'wana tinsimu masiku hinkwawo

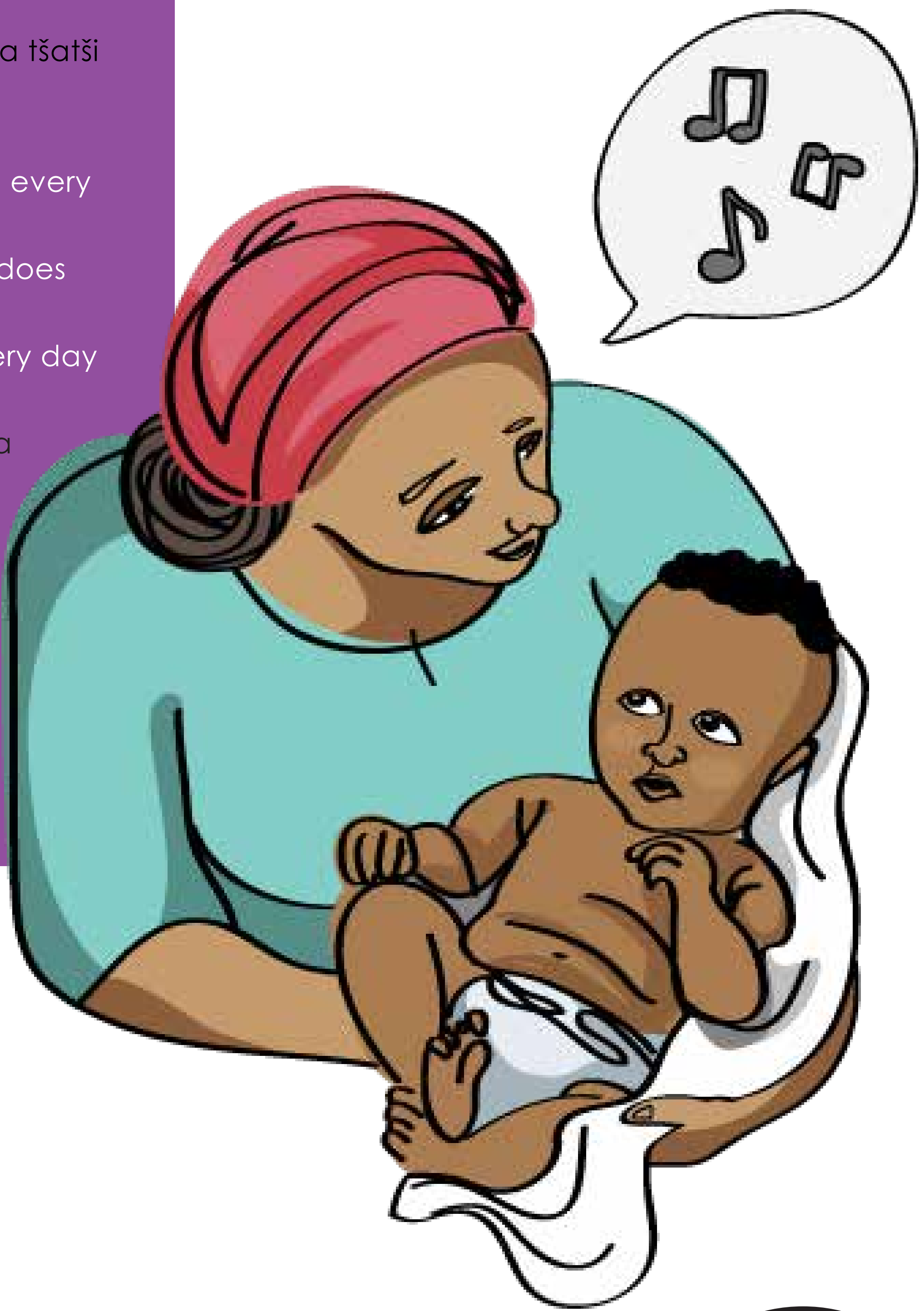

Start

Greet parent and explain purpose of visit.

Ask how parent and child are doing?

Ask caregiver if they have been trying any of the activities since last visit?

Ask if parent can show one of the activities?

Ask if mother/family has any difficulties/challenges in implementing the advice or activities?

Ask if mother/family observed any benefits in implementing the advice or activities?

Ask mother/family if she has any concerns (e.g. child is sick, appears slower to develop)?

Maternal Wellbeing

- Mom should get a general check-up at the clinic at least once in the six months after delivery. Check whether mom has been to the clinic for a check-up since delivery. If not, encourage her to visit clinic soon.

Child Health

- If baby has fever, cough, or is not feeding, they should be brought to the clinic immediately. Describe how to check for symptoms. Encourage immediate care seeking if symptoms found.
- Sometimes, sick babies might seem like they do not want to eat. However, it is very important for baby's health that she continue to be fed during sickness.
- Check whether baby has received weight or height measurement since birth. If not, encourage mom to take baby to clinic soon to be checked.

Child Nutrition

- At this age, mom can start to introduce simple new foods to baby. Encourage mom to give baby one new food each week, for one meal each day. Give examples of new foods.
- While new foods are introduced one meal each day, mom should continue to breastfeed for all other meals. Encourage mom to keep breastfeeding as long as possible, though her milk production may start to decline.

Child Milestones

- Baby might reach for objects. Demonstrate how mom can look for this.
- Baby might recognize family at a distance. Demonstrate how mom can check for this.

Child Activity

- Demonstrate how mom can make baby smile by tickling or making funny faces.
- Showing baby love with positive verbal praise (e.g., "good job!") will help baby deal with challenges as they grow. Encourage mom to give baby verbal praise when baby does new things.
- Songs will help to soothe and calm baby. Encourage mom to sing songs to baby every day.

Observe parent and child trying activity. If needed demonstrate again to help caregiver

Ask if the caregiver has any questions or concerns?
Arrange next visit date

Thank mother and family. Encourage mother to try implement the advice and activities.

Note any questions asked, action needed, referral required, or things to discuss with supervisor/team leader

End

S
E
T

Child Activity

- Bitša ngwana o mo tumiše
- Tumiša ngwana ge a dira dilo tše diswa
- Leka go dira ngwana gore a meyemeyele ka matjatji

- Call baby by name and applaud
- Praise baby when she does new things
- Try to make baby smile every day

- Vulavula na xihlangi hi swilo leswi xi swi langutaka
- Tlanga ntlangu wa '[xilo] xi le kwini?'
- Ringeta ku endla n'wana a n'wayitela masiku hinkwawo

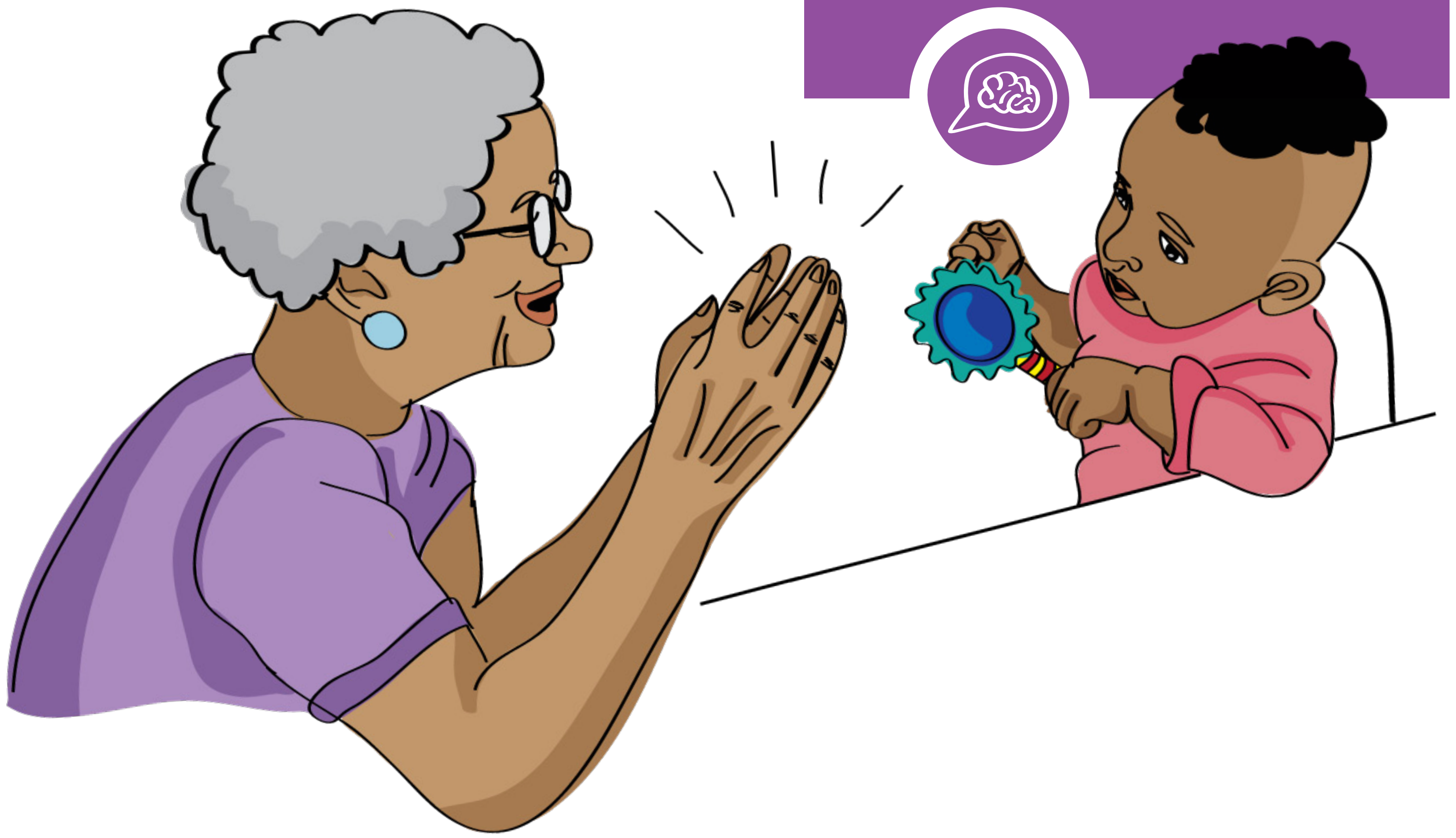

Child Milestones

- Lesea le ka dula ntle le go thekgwa
- Lesea le ka moma dilo ka molomong

- Baby might sit without support
- Baby might put things in mouth

- Xihlangi xi nga tshama handle ka ku seketeriwa
- Xihlangi xi nga hoxa swilo enon'weni

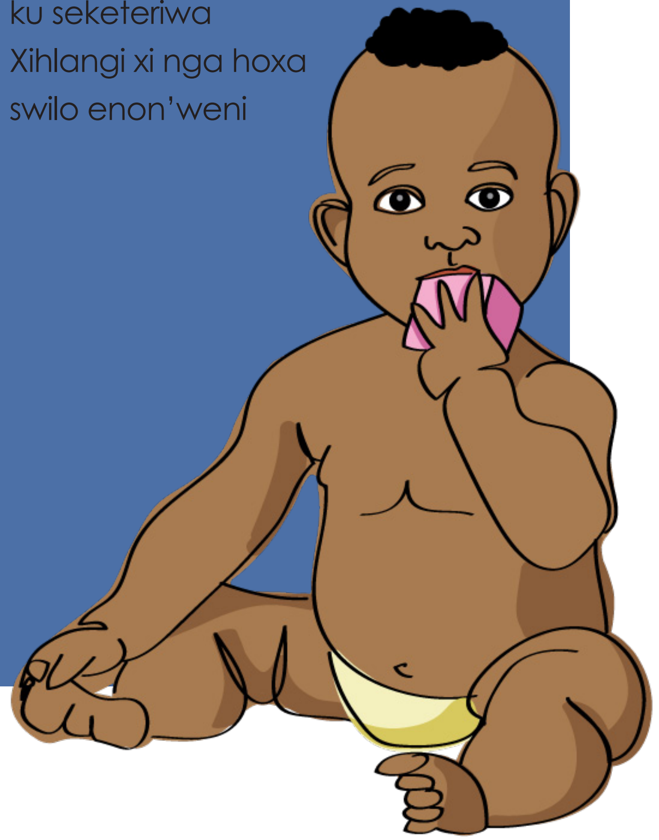

Child Health

- E ba le tsebo ka ga letšhollo
- Šomiša motswako wa go thibela letšhollo (ORS)
- Lesea le hloka maphelo a mabotse

- Look for diarrhea
- Use oral rehydration salt (ORS) solution
- Baby needs good hygiene

- Languta nchuluko
- Tirhisa diripi (oral rehydration salt [ORS])
- Swihlangi swi lava nsivelamavabyi wa kahle

Child Nutrition

- Go nyantšha letswele + go fa dijo makga a 2
- Mahwana a teye a 2-3 a proteine (dinawa goba mae)

- Breastfeeding + 2 meals
- 2-3 teaspoons protein (beans or eggs)

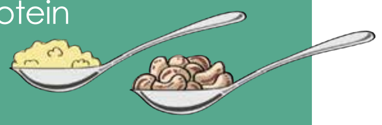

- Vitana n'wana hi vito unwibela mavoko.
- Ndhundhuzela n'wana loko a endla swilo leswi ntshwa.

Start

Greet parent and explain purpose of visit.

Ask how parent and child are doing?

Ask caregiver if they have been trying any of the activities since last visit?

Ask if parent can show one of the activities?

Ask if mother/family has any difficulties/challenges in implementing the advice or activities?

Ask if mother/family observed any benefits in implementing the advice or activities?

Ask mother/family if she has any concerns (e.g. child is sick, appears slower to develop)?

Child Health

- As baby starts to eat more and more new foods, the risks for diarrhea may increase. Encourage mom to pay close attention to whether baby has diarrhea, particularly if baby is showing signs of dehydration or malnutrition due to diarrhea.
- Encourage mom to give baby ORS when baby has diarrhea to avoid dehydration. Demonstrate for mom how to make ORS or discuss where she can buy ORS packets nearby.
- Baby is getting older and starting to move around and touch more things. This means baby is being exposed to more bacteria around the house. Encourage mom to clean baby regularly, every day if possible.

Child Nutrition

- At this age, baby's daily diet should include 2 meals of food and breastfeeding for all other meals. Discuss with mom a plan for the 2 food meals and continued breastfeeding.
- Baby needs protein to grow and develop properly. Breastmilk has lots of good protein. For the food meals, mom should give baby 2 – 3 teaspoons of protein. Discuss local options for protein for baby, for example beans or eggs.

Child Milestones

- Baby might sit without support. Demonstrate how mom can look for this.
- Baby might put things in mouth. Demonstrate how mom can look for this.

Child Activity

- Baby is starting to learn to respond to own name. Demonstrate how mom can call baby by name and applaud (e.g., "Good job!") when baby responds
- Showing baby love with positive verbal praise (e.g., "good job!") will help baby deal with challenges as they grow. Encourage mom to give baby verbal praise when baby does new things.
- Demonstrate how mom can make baby smile by tickling or making funny faces.

Observe parent and child trying activity. If needed demonstrate again to help caregiver

Ask if the caregiver has any questions or concerns?
Arrange next visit date

Thank mother and family. Encourage mother to try implement the advice and activities.

Note any questions asked, action needed, referral required, or things to discuss with supervisor/team leader

End

S
E
T

Child Health

- E ba le tsebo ka ga letadi
- Hwetša kalafo ka potlako ge lesea le na le letadi
- Lesea le swanetše go khupetšwa ka nete ge le robetše

- Look for fever
- Seek care immediately if baby has fever
- Baby should sleep under net

- Kamba ku hisa ka miri
- Lava vutshunguri hi xihatla loko xihlangi xi hisa miri
- Xihlangi xi fanele ku etlerisiwa endzeni ka nete

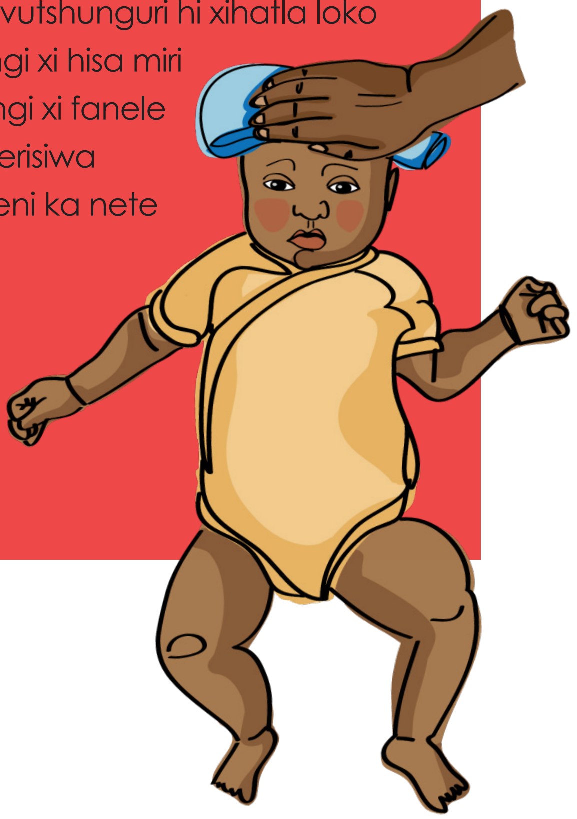

Child Milestones

- Lesea le ka araba ge leina le bitšwa
- Lesea le ka thoma go abula

- Baby might respond to name
- Baby might start to crawl

- Xihlangi xi nga tiva vito ra xona loko ri vitaniwa
- Xihlangi xi nga sungula ku kasa

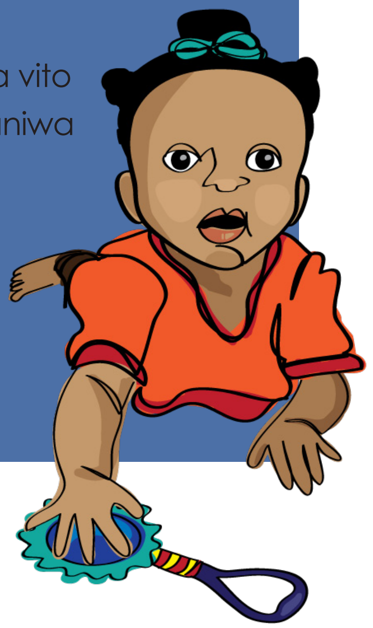

Child Nutrition

- Go nyantšha letswele + go fa dijo makga a 2
- Masea a rata dijo tše ntši

- Breastfeeding + 2 meals
- Baby likes many foods

- Ku mamisa vele + miphamelo yi mbirhi
- Xihlangi xi tsakela swakudya swo hambanahambana

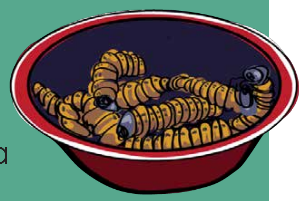

Child Activity

- Thuša ngwana go dula le go shuta go opelela
- Phuphuta matsogo le ngwana
- Akara le go suna ngwana ge a nyamile

- Help baby sit and move to singing
- Clap hands with baby
- Hug and kiss baby when she is upset

- Pfuna nwana a tshama ni ku fambisas a kha yimbelela
- Phokotela mavoko na nwana
- Vukarha nwana ni ku nwi ntswontswa loko a kwatili

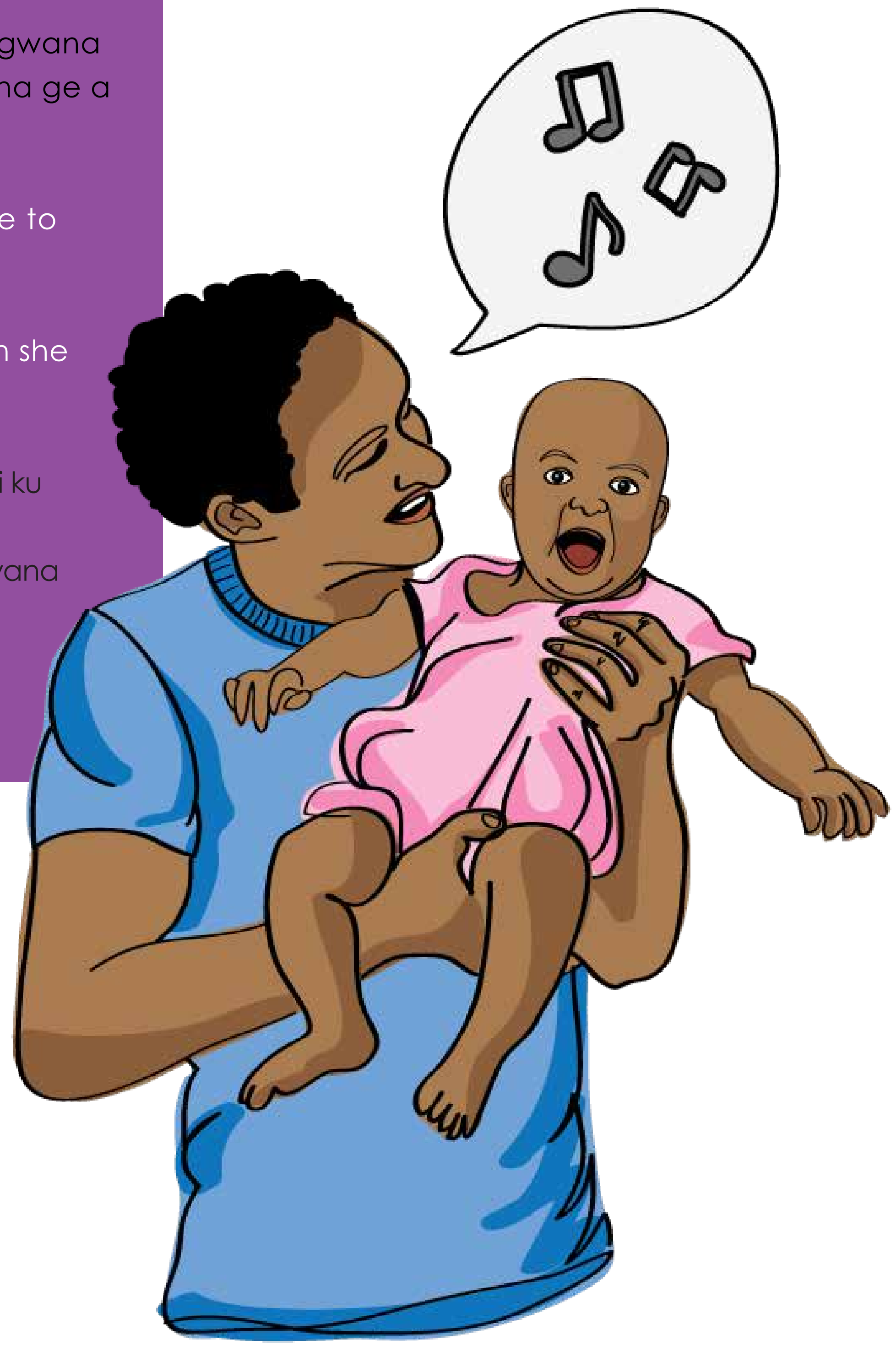

Start

Greet parent and explain purpose of visit.

Ask how parent and child are doing?

Ask caregiver if they have been trying any of the activities since last visit?

Ask if parent can show one of the activities?

Ask if mother/family has any difficulties/challenges in implementing the advice or activities?

Ask if mother/family observed any benefits in implementing the advice or activities?

Ask mother/family if she has any concerns (e.g. child is sick, appears slower to develop)?

Child Health

- Check baby for signs of fever and demonstrate for mom how she can also check. Encourage mom to pay close attention to baby for fever symptoms.
- Encourage mom to seek care immediately if baby has signs of fever. Explain that fever might be a sign of malaria or other infection and that the clinic has medicines that can help.
- Encourage mom to place baby under an insecticide treated bednet at night to protect baby against mosquitos that might cause malaria.

Child Nutrition

- At this age, baby's daily diet should include 2 meals of food and breastfeeding for all other meals. Discuss with mom a plan for the 2 food meals and continued breastfeeding.
- Baby needs a diverse diet with protein and other micronutrients to grow and develop properly. Breastmilk has lots of good protein and nutrients. For the food meals, mom should give baby a variety of other foods. Discuss adding at least one new food to increase diversity and add protein or micronutrients.

Child Milestones

- Baby might respond to name. Demonstrate how mom can look for this.
- Baby might start to crawl. Demonstrate how mom can look for this

Child Activity

- As baby learns to control body better, mom can start to "dance" with baby while singing. Demonstrate how mom can "dance" by having baby sit and move as mom sings to baby.
- Demonstrate how mom can sit with baby and hold baby's hands and have baby clap with mom as she sings a song or says a rhyme to baby.
- Comforting baby when she is upset will teach her to deal with challenges in a positive way as she grows. Encourage mom to given baby hugs and kisses when she is upset.

Observe parent and child trying activity. If needed demonstrate again to help caregiver

Ask if the caregiver has any questions or concerns?
Arrange next visit date

Thank mother and family. Encourage mother to try implement the advice and activities.

Note any questions asked, action needed, referral required, or things to discuss with supervisor/team leader

End

S
E
T

Child Activity

- Dira ngwana gore a kopiše mediro ya gago o mo tumishe/ rete
- Raloka makhutelana le ngwana
- Kuka ngwana o bolele le yena ge a nyamile

- Have baby copy your actions and applaud
- Play Peekaboo with the baby
- Hold baby and talk to baby when upset

- Endla n'wana a kopela swiendlo swa wena I vi I n'wu bela mavoko
- Tlanga ntlangi wa xitumbelelani na n'wana
- Tlakula n'wana ni ku valavula na yena loko a kwatili

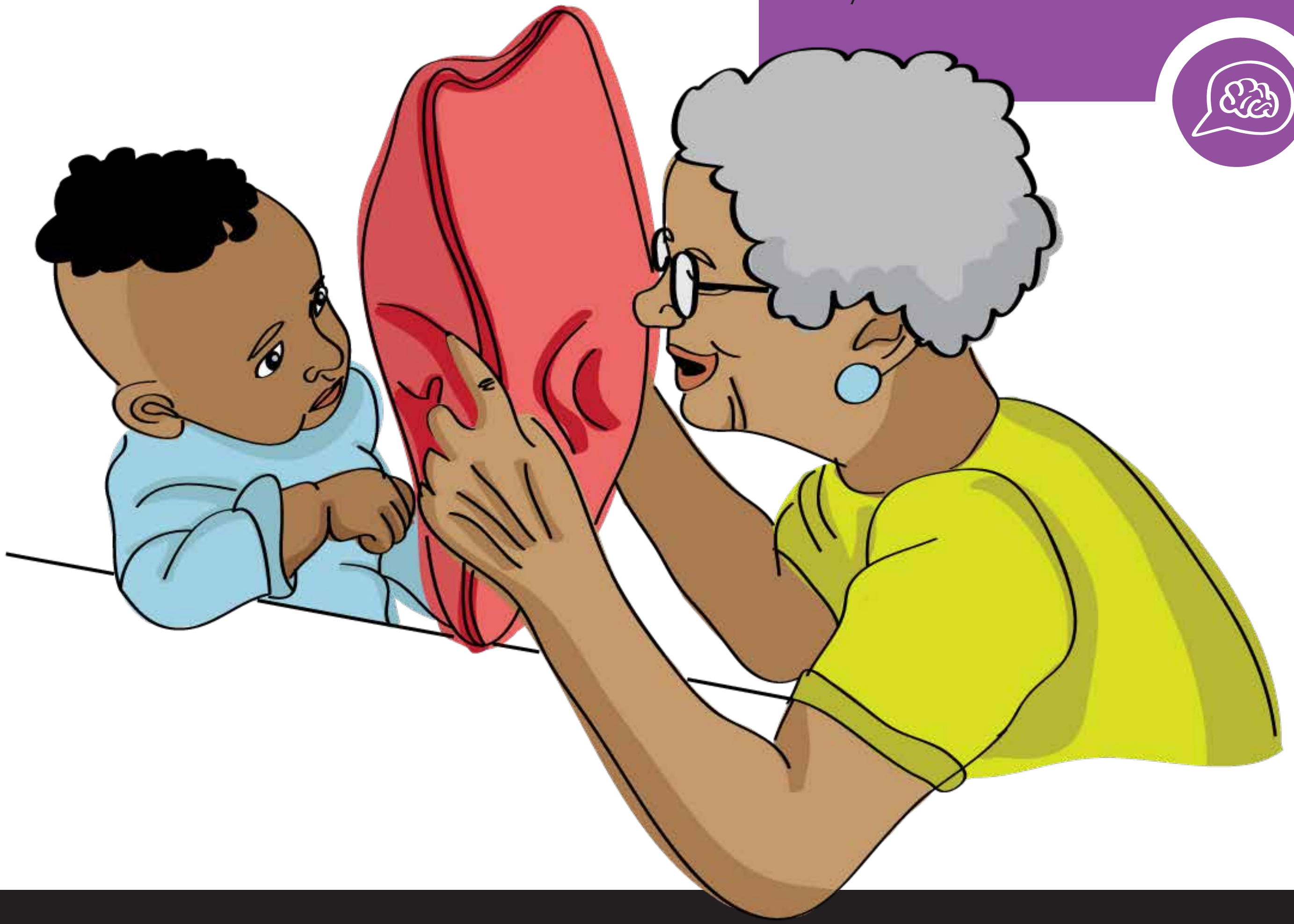

Child Milestones

- Lesea le ka namela dilo goba go emelela
- Lesea le ka tseba selo le sa se bone

- Baby might pull up or stand
- Baby might know object without seeing

- Xihlangi xi nga ringeta ku yima
- Xihlangi xi nga tiva swilo handle ka ku swi vona

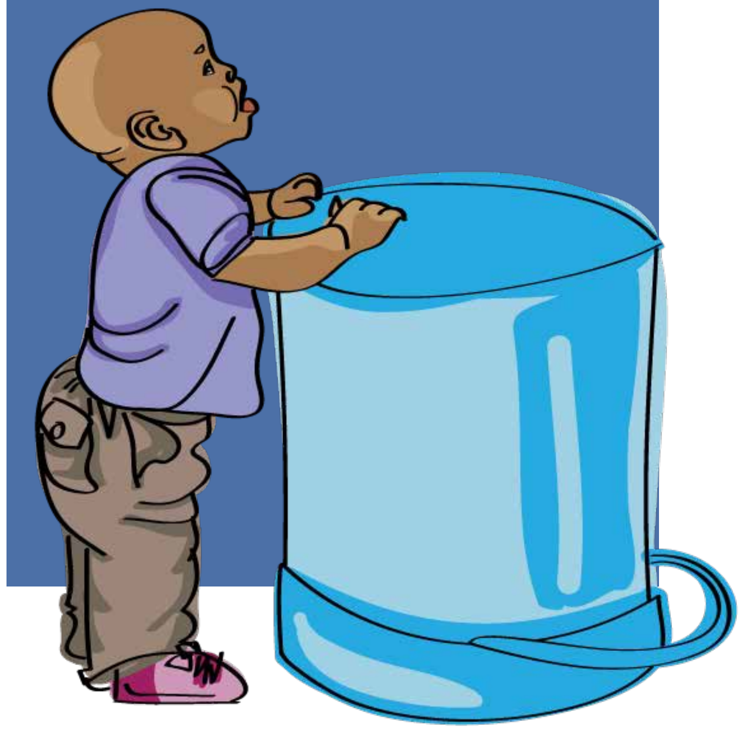

Child Health

- E ba le tsebo ka ga go gohlola le bothata bja go hema
- Hwetša kalafo ka potlako ge lesea le na le bothata bja go hema

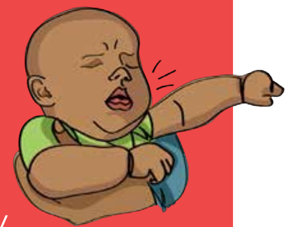

- Look for cough and difficulty breathing
- Seek care immediately if baby has difficulty breathing

- Kamba ku khohlola kumbe ku tik- eriwa ka ku hefemula
- Lava ku pfuniwa hi xihatla loko n'wana a tikeriwa ku hefemula

Child Nutrition

- Go nyantšha letswele + go fa dijo makga a 3
- Kgonthiša gore meetse a go nwa a hlwekile

- Breastfeeding + 3 meals
- Make sure drinking water is clean

- Ku mamisa vele + miphamelo yi 3
- Tiyisisa leswaku mati yo nwa ya basile

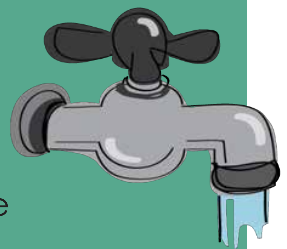

Start

Greet parent and explain purpose of visit.

Ask how parent and child are doing?

Ask caregiver if they have been trying any of the activities since last visit?

Ask if parent can show one of the activities?

Ask if mother/family has any difficulties/challenges in implementing the advice or activities?

Ask if mother/family observed any benefits in implementing the advice or activities?

Ask mother/family if she has any concerns (e.g. child is sick, appears slower to develop)?

Child Health

- Check baby for signs of cough and respiratory infection and demonstrate for mom how she can also check. Encourage mom to pay close attention to baby for respiratory symptoms.
- Encourage mom to seek care immediately if baby has danger signs of respiratory infection, including fast breathing and wheezing sounds. Explain that symptoms may be a sign of pneumonia or other infection and that the clinic has medicines that can help.

Child Nutrition

- At this age, baby's daily diet should include 3 meals of food and breastfeeding for all other meals. Discuss with mom a plan for the 3 food meals and continued breastfeeding.
- Explain to mom that dirty drinking water can make baby sick. Ask mom about current water source and discuss best ways to keep water clean.

Child Milestones

- Baby might pull herself up on household furniture or start to stand. Demonstrate how mom can look for this.
- Baby might know where objects are without directly looking at them. Demonstrate how mom can look for this.

Child Activity

- Mom can have a "conversation" with baby without speaking. Demonstrate how mom can have baby copy her actions. Encourage mom to do this and give praise when baby copies actions.
- Demonstrate how mom can play Peekaboo with the child to make child laugh.
- Hold baby and talk to baby when upset, try to explain things to baby.

Observe parent and child trying activity. If needed demonstrate again to help caregiver

Ask if the caregiver has any questions or concerns?
Arrange next visit date

Thank mother and family. Encourage mother to try implement the advice and activities.

Note any questions asked, action needed, referral required, or things to discuss with supervisor/team leader

End

S
E
T

Child Health

- Ditšhwaana tša thibela malwetši di šireletša ngwana
- Maphelo a ama kgodišo ya lesea

- Vaccines protect baby
- Health affects baby's development
-

- Misawutiso yi sirhelela n'wana
- Rihanyu ri khumba makulele ya xihlangi

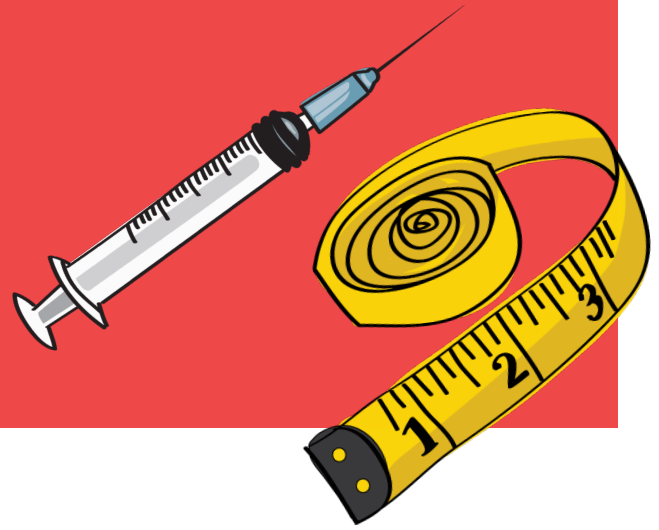

Child Milestones

- Lesea le ka sepela ka go phakiša
- Lesea le ka leka go bolela

- Baby might move faster
- Baby might to try to talk

- Xihlangi xi nga famba hi ku hatlisa
- Xihlangi xi nga ringeta ku vulavula

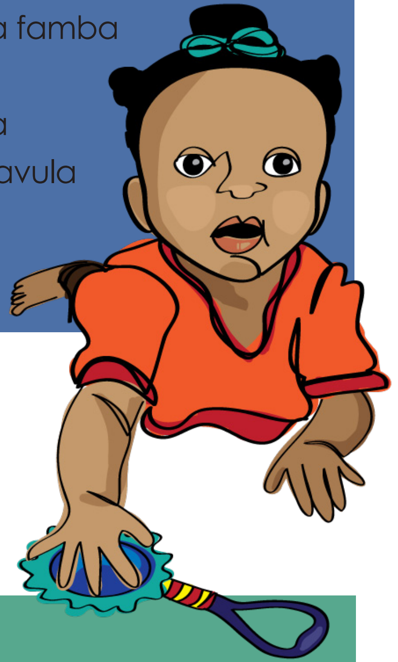

Child Nutrition

- Phepo e na le seabe go kgolo ya lesea
- Naa lesea le swanetše go ba lesea nako e kaakang?

- Nutrition affects baby's development
- How long should baby be?

- Tinyutiriyente ti khumba makulele ya xihlangi
- Xana xihlangi xi fanele ku va na vulehi byo tani hi kwihi?

Child Activity

- Rutiša lesea maina a batho
- Rutiša lesea 'dumela" le 'sala gabotse"
- Botšiša lesea gore o kae mma /papa le go akara

- Teach baby names of people
- Practice 'hello' and 'bye-bye'
- Ask baby "where is mama/ papa" and give hugs

- Dyondzisa n'wana mavito ya vanhu
- Endlani 'ahe' na 'tata'
- Vutisa n'wana 'va kwini manana /papa' ni ku nwi vukarha

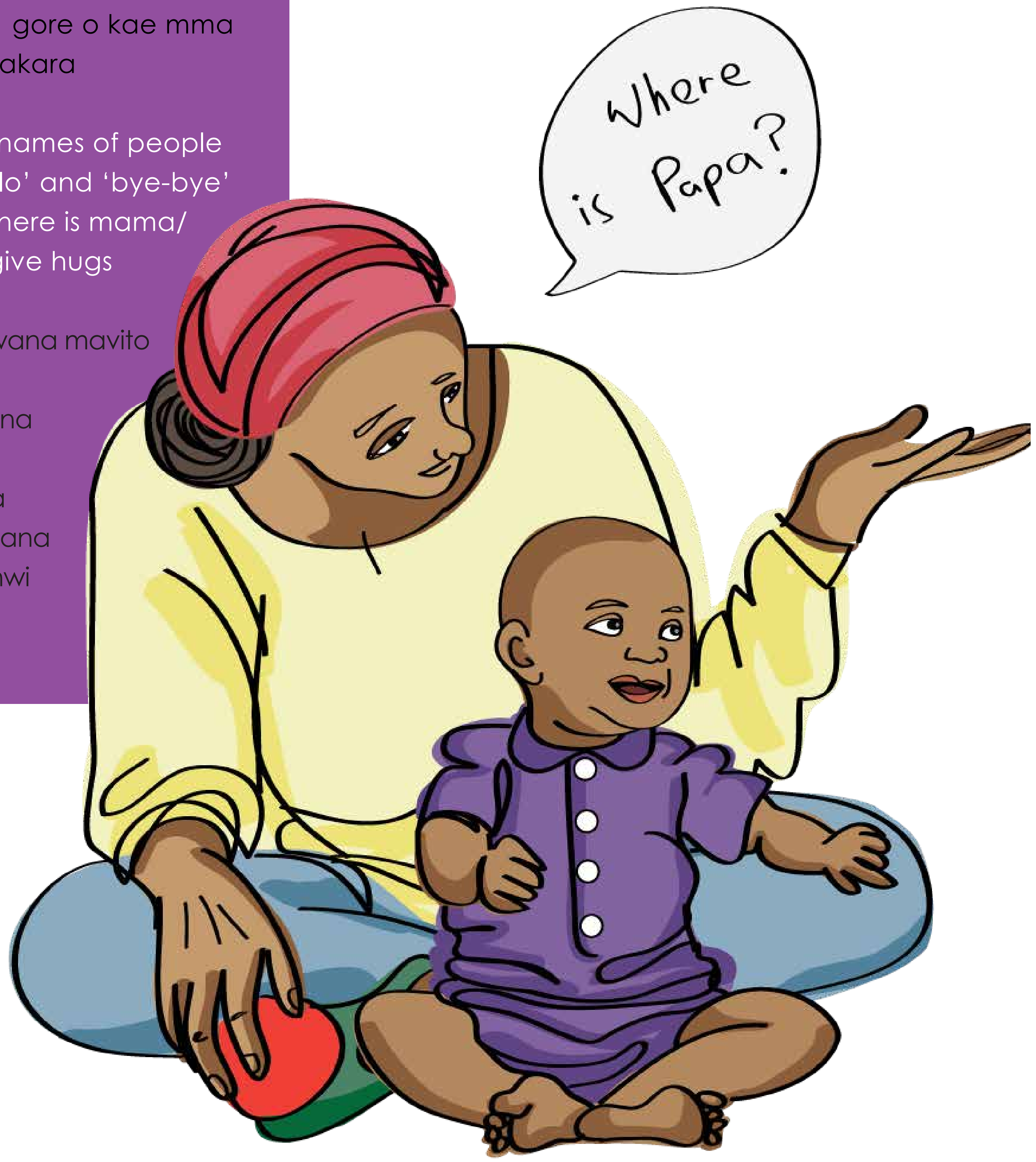

Start

Greet parent and explain purpose of visit.

Ask how parent and child are doing?

Ask caregiver if they have been trying any of the activities since last visit?

Ask if parent can show one of the activities?

Ask if mother/family has any difficulties/challenges in implementing the advice or activities?

Ask if mother/family observed any benefits in implementing the advice or activities?

Ask mother/family if she has any concerns (e.g. child is sick, appears slower to develop)?

Child Health

- At 9 months old, babies should receive measles1 and PCV3. Confirm that the baby has received these vaccines. If not yet received, encourage mom to bring baby to the clinic for these vaccines immediately.
- Baby's health affects physical growth and development. It is important that mom seeks care for baby when baby has symptoms of illness. Even if symptoms do not seem to serious, untreated infections may slow the baby's development over time. Mom should take baby to the clinic for a general checkup every three months or so. Ask mom if baby has been checked recently, and if not encourage mom to take baby to clinic for a checkup.

Child Nutrition

- Baby's nutrition affects physical growth and development. It is important that baby has enough protein and micronutrients in their diet. If they do not, their growth may be slowed.
- Baby's length is an important sign of their development, and is related to things like how the baby will perform in school later. Ask mom if baby's length has been measured recently (check Road to Health Booklet) and if baby is stunted. Talk with mom about how baby's nutrition might be improved to ensure better growth.

Child Milestones

- Baby might move faster and start to explore more. Demonstrate how mom can look for this.
- Baby might try to talk but will likely not be able to form clear words. Demonstrate how mom can look for this.

Child Activity

- Baby is starting to remember the faces of family members she sees often. Encourage mom to talk to baby and tell her the names of the people she sees.
- Baby is making noises but not yet talking. Demonstrate how mom can practice saying 'hello' and 'bye-bye' with baby.
- Demonstrate how mom and dad can play the game with baby of asking "where is mama" and "where is papa". When baby finds mama and papa they should give baby hugs and kisses.

Observe parent and child trying activity. If needed demonstrate again to help caregiver

Ask if the caregiver has any questions or concerns?
Arrange next visit date

Thank mother and family. Encourage mother to try implement the advice and activities.

Note any questions asked, action needed, referral required, or things to discuss with supervisor/team leader

End

S
E
T

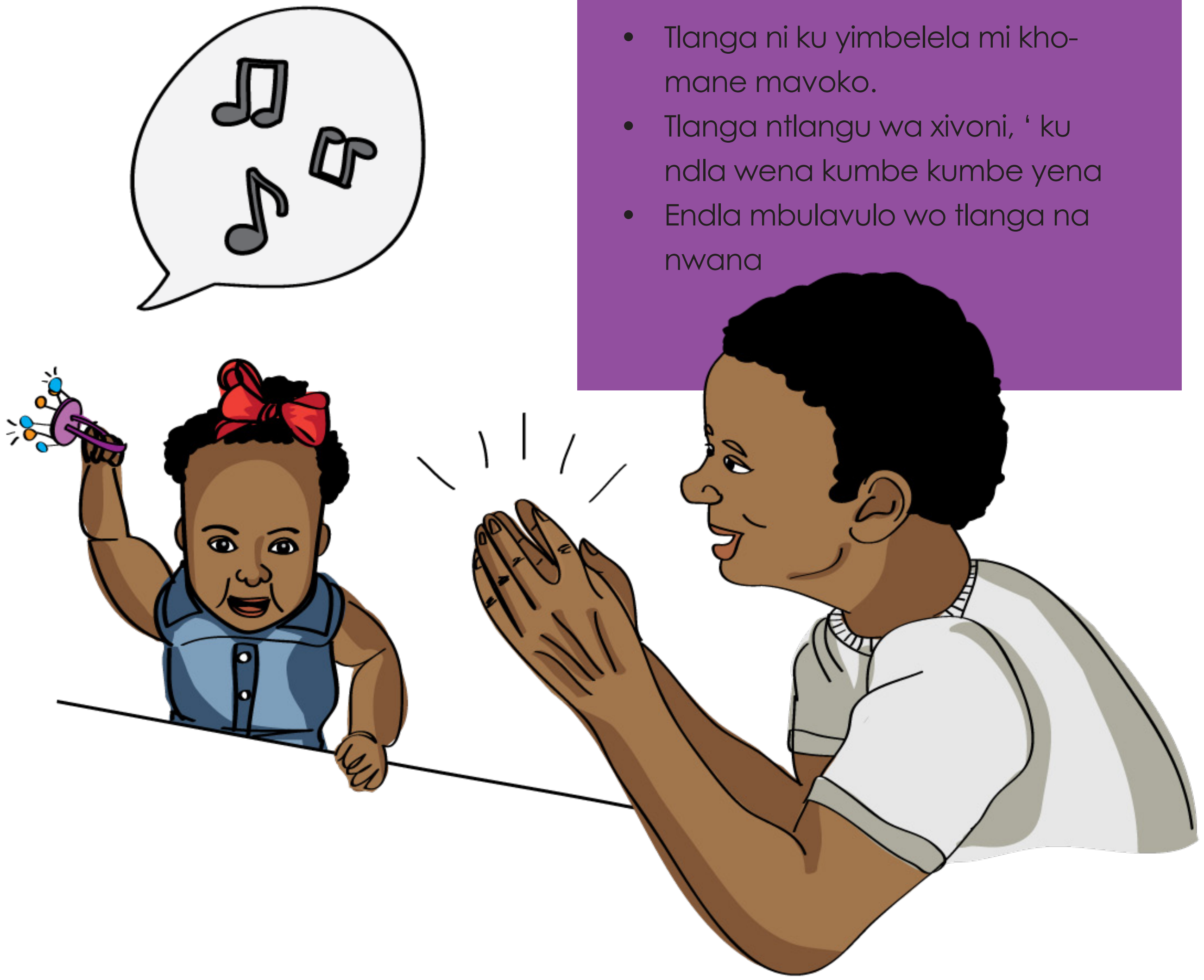

Child Activity

- Raloka le go opelela ka mat-sogo le mekgwa ya go ema
- Raloka thaloko ya sepili 'nako yaka......nako ya go"
- E ba le boledisano ya go itri-rela le ngwana

- Play and sing with hand ges-tures
- Play 'mirror' game, 'my turn . . . your turn'
- Have 'pretend conversations' with child

- Tlanga ni ku yimbelela mi kho-mane mavoko.
- Tlanga ntlangu wa xivoni, ' ku ndla wena kumbe kumbe yena
- Endla mbulavulo wo tlanga na nwana

Child Nutrition

- Go nyantšha letswele + go fa dijo makgа a 4
- Leka motswako o moswa

- Breastfeeding + 4 meals
- Try a new recipe

- Ku mamisa vele + miphamelo ya Mune.
- Ringeta rhesipi yintswa

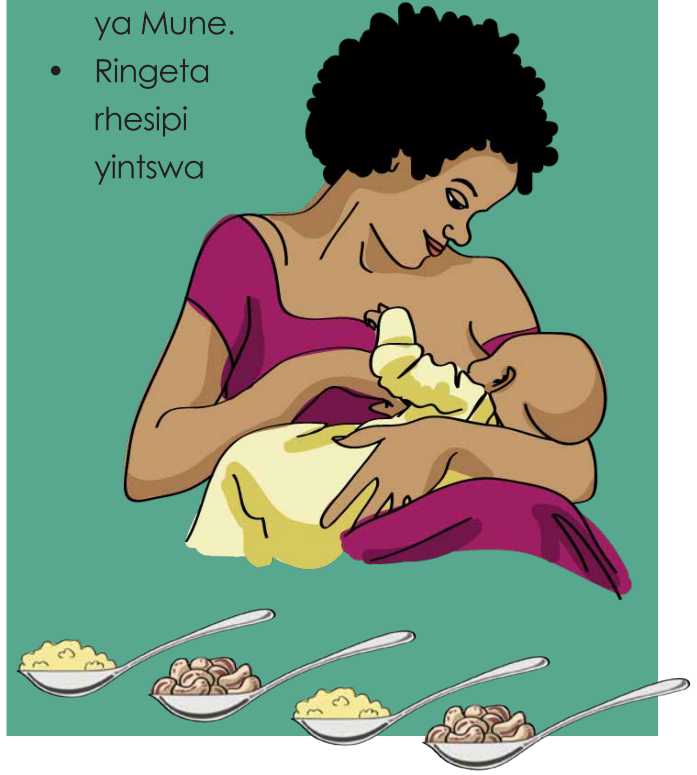

Child Health

- Kgonthiša gore ngwana o bolokegile ka ntlong

- Make house safe for child

- Endla leswaku yindlu yi va yo hlayiseka eka xihlangi

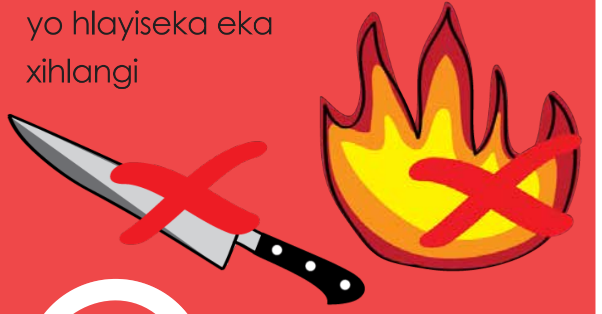

Child Milestones

- Ngwana a ka šupa dilo tše a di nyakago
- Ngwana a ka latela ditaelo tše bonolo go swana le 'go emelela' goba 'go ema'

- Child might point at objects they want
- Child might follow simple com-mands like 'stand up' or 'stop'

- Xihlangi xi nga kombetela swilo leswi xi swi lavaka
- Xihlangi xi nga landzelela swileriso swo olova swo tanihi 'tlakuka' kumbe 'yima'

Start

Greet parent and explain purpose of visit.

Ask how parent and child are doing?

Ask caregiver if they have been trying any of the activities since last visit?

Ask if parent can show one of the activities?

Ask if mother/family has any difficulties/challenges in implementing the advice or activities?

Ask if mother/family observed any benefits in implementing the advice or activities?

Ask mother/family if she has any concerns (e.g. child is sick, appears slower to develop)?

Child Health

- The child is becoming increasingly mobile and is exploring the house more and more. Walk around the house with mom and discuss ways in which the child might face danger or have an accident, and talk with mom about how she can make the house safer for the child.

Child Nutrition

- At this age, child's daily diet should include 4 meals of food and breastfeeding for all other meals. Discuss with mom a plan for the 4 food meals and continued breastfeeding.
- Discuss a new meal that mom can cook for child. Ask about child's preferences for food and make tailored suggestions.

Child Milestones

- Child might point at objects that they want. Demonstrate how mom can look for this.
- Child might follow simple commands like 'stand up' or 'stop'. Demonstrate how mom can check this.

Child Activity

- Demonstrate for mom how to incorporate hand gestures into the songs she sings with the child. Encourage mom to sing with the child each day and use hand gestures in the songs.
- Teach mom the 'mirror' game, where mom does a gesture or activity first and then the child 'mirrors' the gesture or activity. Encourage mom to play the 'mirror' game with the child often.
- The child can make some words but can't fully talk yet. Encourage mom to engage with child as they try to talk by having 'pretend conversations' where mom pretends like child is talking like an grown up.

Observe parent and child trying activity. If needed demonstrate again to help caregiver

Ask if the caregiver has any questions or concerns?
Arrange next visit date

Thank mother and family. Encourage mother to try implement the advice and activities.

Note any questions asked, action needed, referral required, or things to discuss with supervisor/team leader

End

Child Health

- Ngwana o hloka maphelo a mabotse
- Dira gore go tsene moya wo o hlwekilego ka gae

- Child needs good hygiene
- Ventilate home to keep air clean

- Xihlangi xi lava nsivelamavabyi wa kahle
- Vona leswaku yindlu yi nghena moya wo tenga na ku vona leswaku moya wu tshama wu tengile

Child Nutrition

- Go nyantšha letswele + go fa dijo makga a 5
- Leka motswako o moswa

- Breastfeeding + 5 meals
- Try a new recipe

- Ku mamisa vele + miphamelo ya nthlanu
- Ringeta rhesipi yintswa

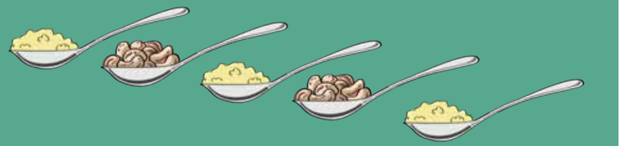

Child Milestones

- Ngwana a ka ganetša ka go šikinya hlogo
- Ngwana a ka sepela a thekgilwe

- Child might shake head ‘no’
- Child might walk with support

- Xihlangi xi nga kombisa ku ‘ala’ hi ku dzungudza nhloko
- Xihlangi xi nga deyisiwa

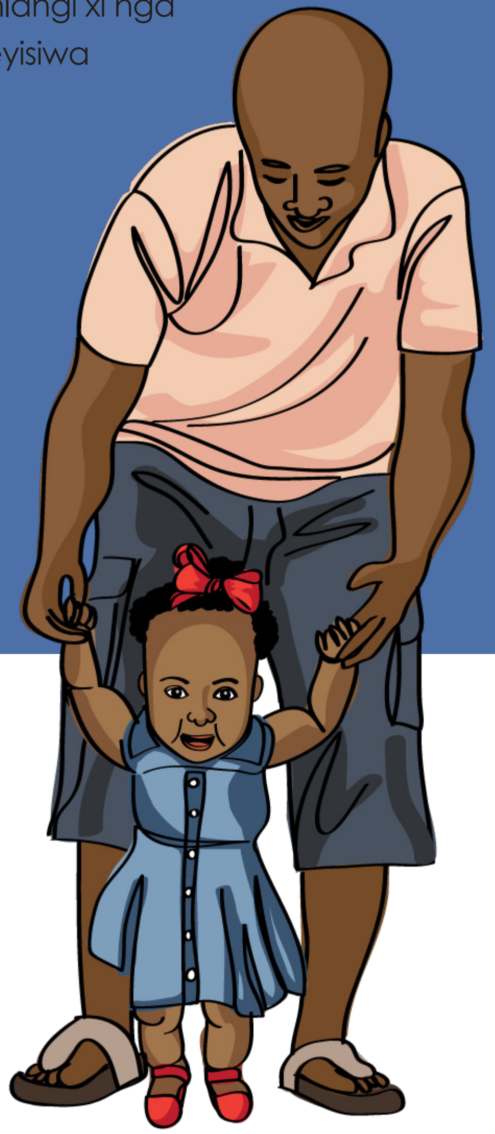

Child Activity

- Botšiša ngwana dipotšišo tše bonolo le go mo akara
- Dira papa gore a botšiše dipotšišo le go akara ngwana
- Go nyane rutiša ngwana melao

- Ask child simple questions and give hugs
- Have father ask questions and give hugs
- Gently teach child rules

- Vutisa n’wana swivutiso swo olova ni ku n’wu vukarha
- Endla papa a vutisa n’wana ni ku n’wu vukarha
- Hi ku olova dyondzisa n’wana milawu

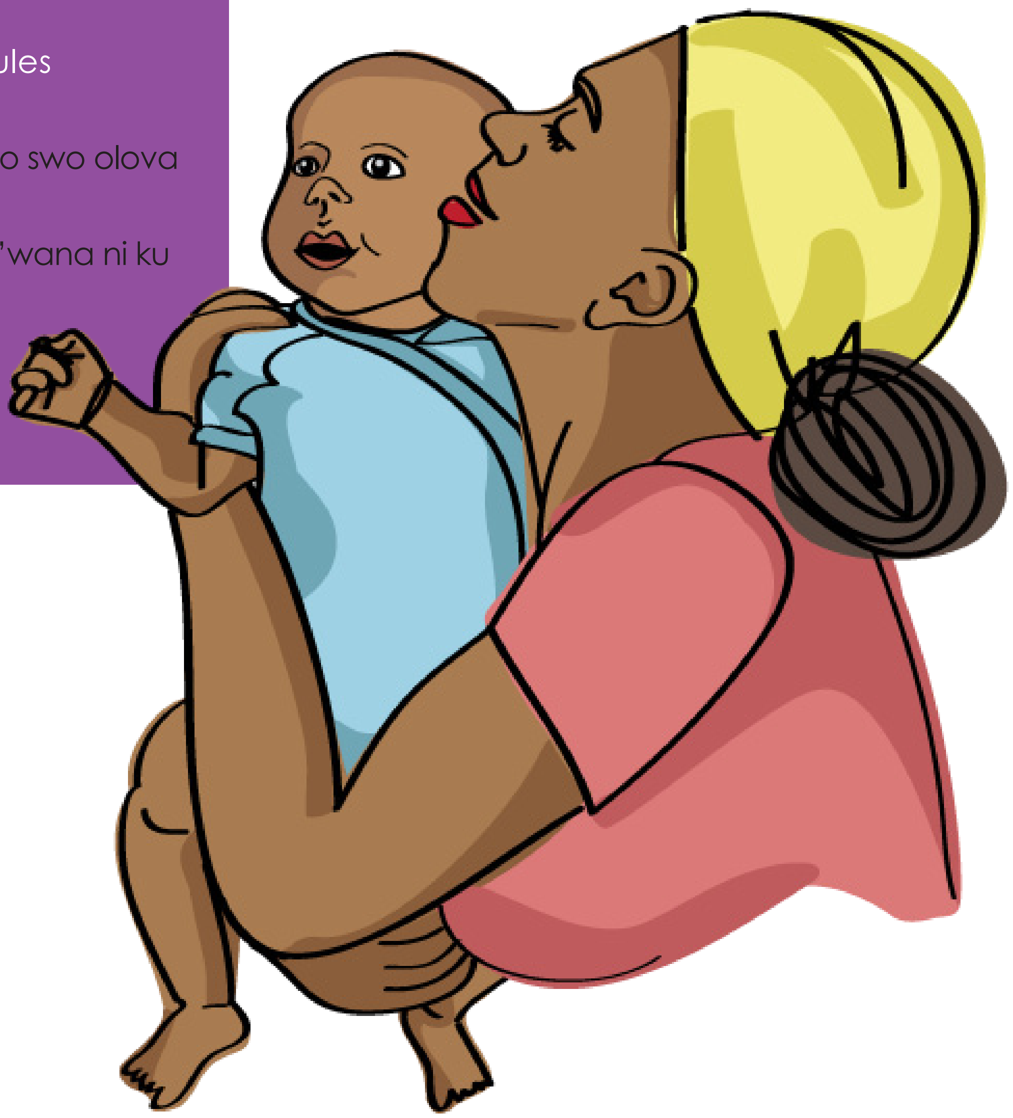

Start

Greet parent and explain purpose of visit.

Ask how parent and child are doing?

Ask caregiver if they have been trying any of the activities since last visit?

Ask if parent can show one of the activities?

Ask if mother/family has any difficulties/challenges in implementing the advice or activities?

Ask if mother/family observed any benefits in implementing the advice or activities?

Ask mother/family if she has any concerns (e.g. child is sick, appears slower to develop)?

Child Health

- Child may now handle their bathroom visits themselves. The child needs to practice good hygiene to avoid infections. Talk with mom and child about adopting good habits.
- Without proper ventilation, air in the home can contribute to illness. This is especially true if cooking is done in the home. Particles from cook stoves can make child sick over time. Ask mom to explain home ventilation and encourage better practices for ventilation if appropriate.

Child Nutrition

- At this age, child's daily diet should include 5 meals of food and breastfeeding for all other meals. Discuss with mom a plan for the 5 food meals and continued breastfeeding.
- Discuss a new meal that mom can cook for child. Ask about child's preferences for food and make tailored suggestions.

Child Milestones

- Child might shake head 'no'. Demonstrate how mom can check for this.
- Child might walk with or without support. Demonstrate how mom can check this.

Child Activity

- The child can give basic responses to mom's questions. Encourage mom to ask child questions and when child responds to give child hugs and kisses.
- Encourage dad to also ask child questions and give hugs when child responds.
- The child is still learning rules and figuring out how to deal with challenges from day to day. When the child gets frustrated, encourage mom to explain to child why there is rules.

Observe parent and child trying activity. If needed demonstrate again to help caregiver

Ask if the caregiver has any questions or concerns?
Arrange next visit date

Thank mother and family. Encourage mother to try implement the advice and activities.

Note any questions asked, action needed, referral required, or things to discuss with supervisor/team leader

End

S
E
T

Child Activity

- Kgopela ngwana gore a go gokaretje a be a go sune
- Fetola polelo ya ngwana ka go myemeyela le go go mo akara
- Rutisa ngwana go ba bonolo ga bana ba bangwe

- Ask child to give you a hug or a kiss
- Respond to child's talk with smile and hug
- Teach child to be gentle with other children.

- Kombela n'wana a ku vukarha kumbe ku ku tswontswa
- Hlamula n'wana hi ku valavula na yena hi ku n'wayitela niku n'wu vukarha
- Dyondzisa n'wana ku va kahle ka vana van'wani

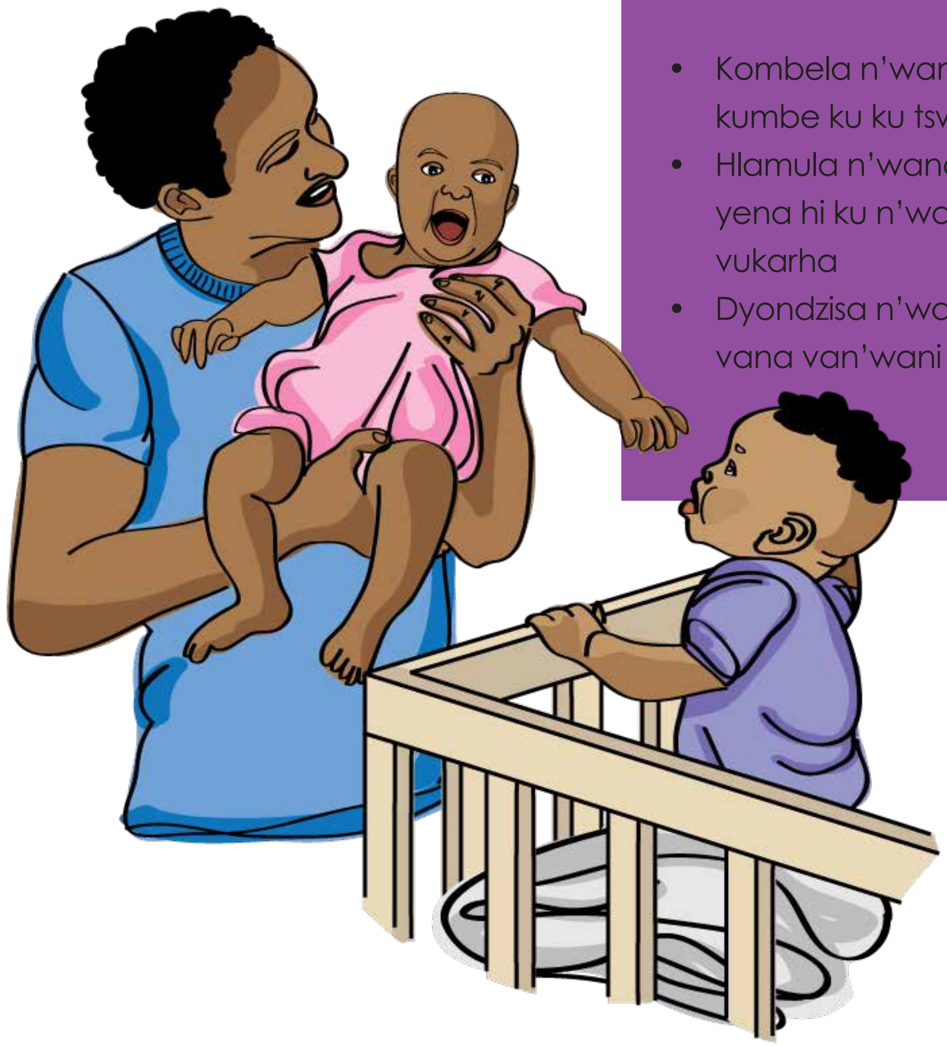

Child Nutrition

- Phepo e ama go gola ga ngwana
- Ngwana o hloka protheine
- Ngwana o swanetše go ja dinawa le mae

- Nutrition affects child's development
- Child needs protein
- Child should eat beans and eggs

- Tinyutiriyente ti khumba makulele ya n'wana
- N'wana u lava phurotheyini
- N'wana u fanele ku dya tinyawa na matandza

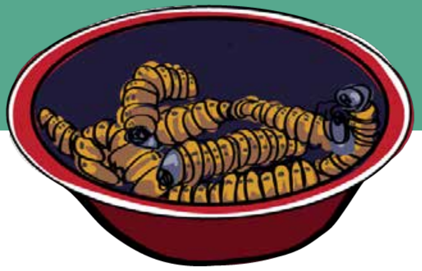

Child Health

- Maphelo a ama kgodišo ya ngwana

- Health affects child's development

- Rihanyu ri khumba makulele ya n'wana

Child Milestones

- Go lakaletša letšatši la matswalo!
- Ngwana a ka sepela a se a thekgwa

- Happy birthday!
- Child might walk without support

- Siku ra kahle ro velekiwa!
- Xihlangi xi nga kota ku famba handle ka nseketelo

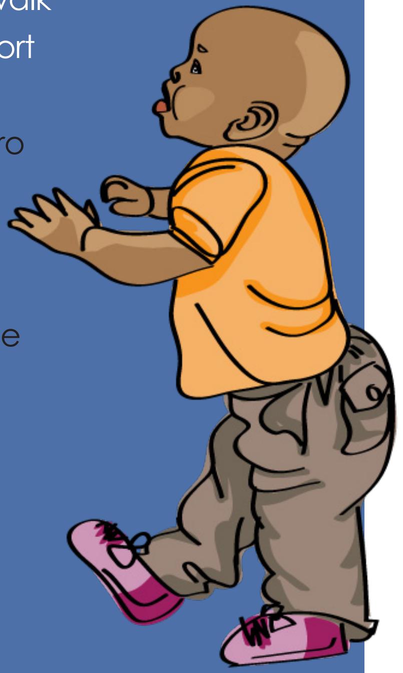

Start

Greet parent and explain purpose of visit.

Ask how parent and child are doing?

Ask caregiver if they have been trying any of the activities since last visit?

Ask if parent can show one of the activities?

Ask if mother/family has any difficulties/challenges in implementing the advice or activities?

Ask if mother/family observed any benefits in implementing the advice or activities?

Ask mother/family if she has any concerns (e.g. child is sick, appears slower to develop)?

Child Health

- Child's health affects physical growth and development. It is important that mom seeks care for child when child has symptoms of illness. Even if symptoms do not seem too serious, untreated infections may slow the child's development over time. Mom should take child to the clinic for a general checkup every three months or so. Ask mom if child has been checked recently, and if not encourage mom to take child to clinic for a checkup.

Child Nutrition

- Child's nutrition affects physical growth and development. It is important that child has enough protein and micronutrients in their diet. If they do not, their growth may be slowed.
- The child needs to consume a good amount of protein through their diet in order for their body to develop properly. Protein will help them grow taller and stronger.
- A few good sources of protein are beans and eggs. Encourage mom to incorporate beans or eggs into the child's diet as much as possible.

Child Milestones

- Child is now one-year-old and is likely twice as large as she was when born.
- Child might walk without support now. Demonstrate how mom can check this.

Child Activity

- As the child grows they can start to understand more questions. Encourage mom to ask child questions as the child gets older.
- Positive praise will reinforce child's desire to learn more words and talk more. Encourage mom to respond to child's talk with hugs and smiles.
- The child is starting to interact with other children more but is still learning the rules of how to interact. Encourage mom to show child to be "nice" to others.

Observe parent and child trying activity. If needed demonstrate again to help caregiver

Ask if the caregiver has any questions or concerns?
Arrange next visit date

Thank mother and family. Encourage mother to try implement the advice and activities.

Note any questions asked, action needed, referral required, or things to discuss with supervisor/team leader

End

Child Health

- Hlapiša ngwana diatla pele ga ge a eja
- Hlapiša ngwana le go fetoša diaparo letšatši le lengwe le le lengwe

- Wash child's hands before they eat
- Bath child and change clothes every day

- Hlambisa xihlangi swandlla xi nga si dya
- Hlambisa xihlangi miri na ku xi cinca swiambalo masiku hinkwawo

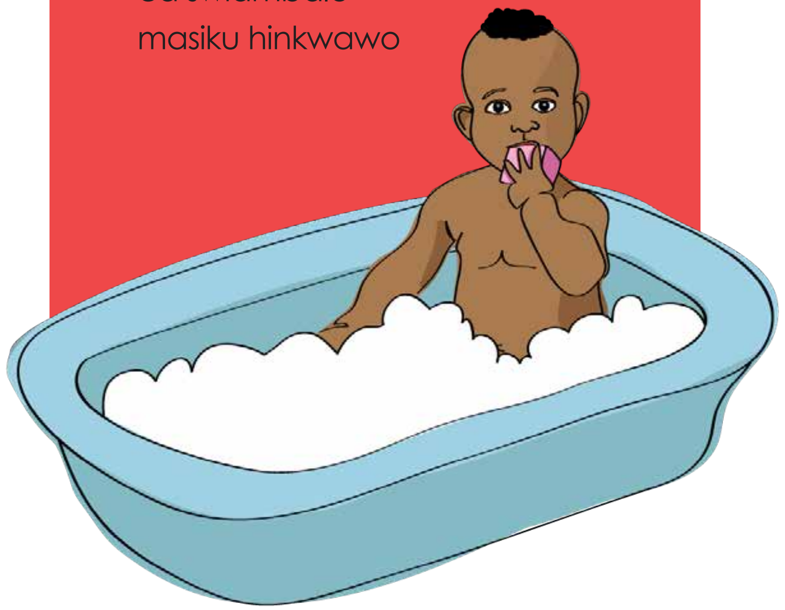

Child Milestones

- Ngwana a ka šomiša lehwana le komiki
- Ngwana a ka rata go thalathala

- Child might use spoon and cup
- Child might like to draw

- Xihlangi xi nga tirhisa lepula kumbe khapu
- Xihlangi xi nga tsakela ku dirowa

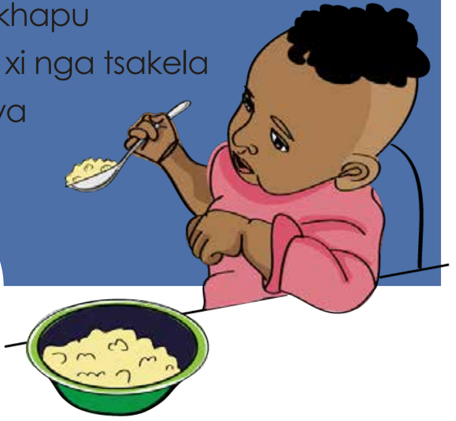

Child Nutrition

- Ngwana o hloka bitamine A
- Ngwana o swanetše go ja merogo ye metala ya mahlare

- Child needs vitamin A
- Child should eat leafy green vegetables

- N'wana u lava vhitamini A
- N'wana u fanele ku dya matsavu yo tsakama

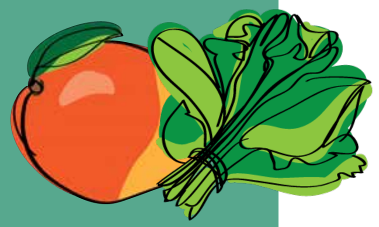

Child Activity

- Dira ngwana gore a tšee karolo go opelela le go bina
- Memela bana go tšea karolo ya go opelela le go bina
- Tšea ngwana go sepela le go ya maeto

- Have child participate in singing and dancing
- Invite siblings to join singing and dancing
- Take child with on walks or trips

- Endla n'wana a nghenelela ka ku yimbelela no cina
- Rhamba vamakwavo ku ta va cina no yimbelela
- Teka nwana mi famba famba swinwe

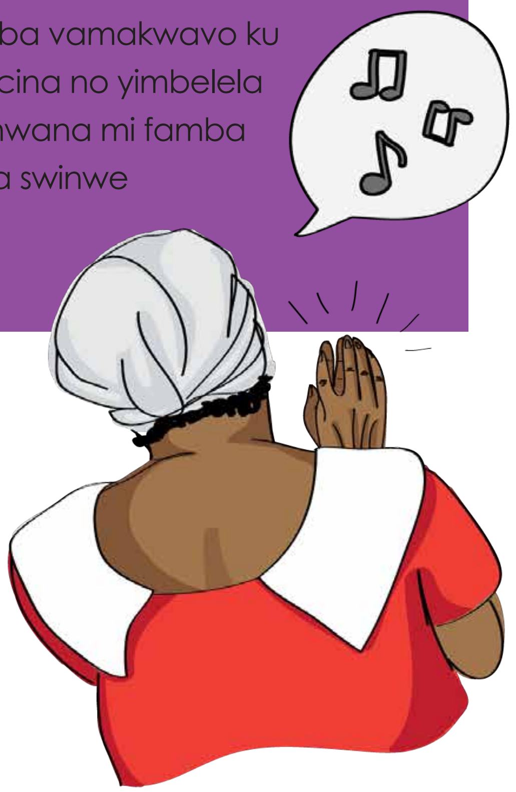

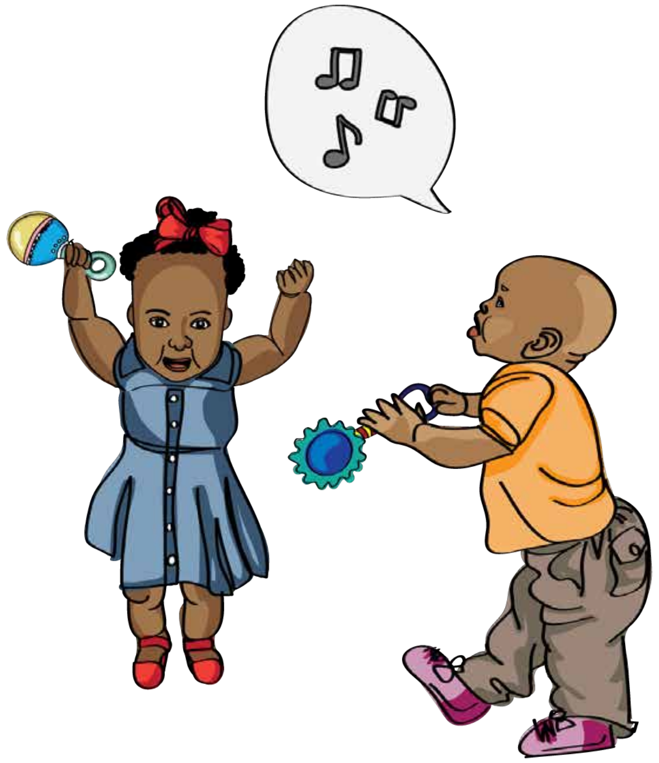

Start

Greet parent and explain purpose of visit.

Ask how parent and child are doing?

Ask caregiver if they have been trying any of the activities since last visit?

Ask if parent can show one of the activities?

Ask if mother/family has any difficulties/challenges in implementing the advice or activities?

Ask if mother/family observed any benefits in implementing the advice or activities?

Ask mother/family if she has any concerns (e.g. child is sick, appears slower to develop)?

Child Health

- Demonstrate for mom how they can wash the child's hands. Encourage mom to wash the child's hands before every meal. This will reduce the bacteria the child consumes that might make them sick.
- As the child moves and explores more, they will get dirty more quickly and will also be exposed to more bacteria. Explain to mom that the child needs to be cleaned more often to ensure good health.

Child Nutrition

- The child needs to consume a good amount of vitamin A through their diet in order for their body to develop correctly. In particular, vitamin A will help them fight infections.
- One good source of vitamin A is leafy green vegetables. Encourage mom to incorporate leafy green vegetables into the child's diet as much as possible.

Child Milestones

- Child might use a spoon and cup when eating and drinking. Demonstrate how mom can check for this.
- Child might like to draw with pencils or crayons. Demonstrate how mom can check this.

Child Activity

- Child developing more and more ability to participate in mom's singing and dancing. Encourage mom to sing and dance with the child each day and have child participate.
- Encourage mom to have child's siblings also join in singing and dancing.
- As child becomes more mobile, mom should bring child with on walks or trips to the market. Encourage mom to take child on trips when possible.

Observe parent and child trying activity. If needed demonstrate again to help caregiver

Ask if the caregiver has any questions or concerns?
Arrange next visit date

Thank mother and family. Encourage mother to try implement the advice and activities.

Note any questions asked, action needed, referral required, or things to discuss with supervisor/team leader

End

S
E
T

Child Activity

- Šomiša nako e ntshi o thtlheletša miaswaro a botse
- Akara ngwana go maiswaro a botse.
- Dumelela ngwana go rakola le bana bangwe ba megwaga ya go lekana le ya gwe

- Spend more time encouraging good behaviour
- Give child hugs for good behaviour
- Let child play with other children of similar age

- Tshamani nkarhi wo leha ku hlohletela mahanyelo lamanene
- Vukarha n'wana ka mahanyelo lamanene
- Pfumelela nwana a tlanga ni vana vo ringana na yena

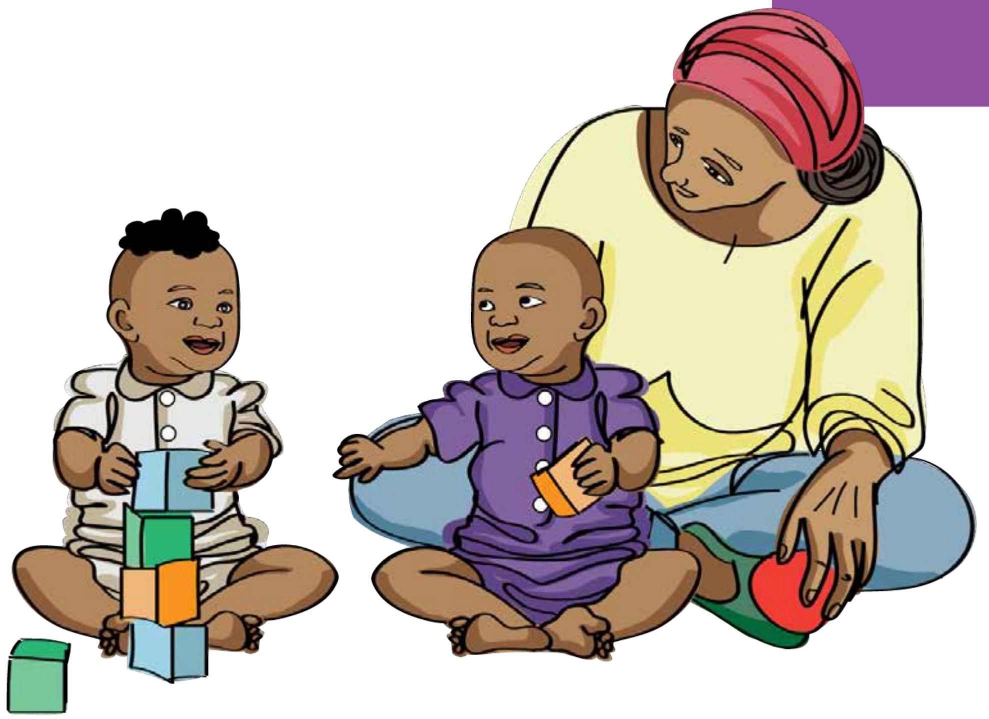

Child Milestones

- Ngwana a ka bolela mantšu a mmalwa
- Ngwana a ka foša goba a raga dilo bjalo ka kgwele

- Child might say a few words
- Child might throw or kick objects like a ball

- Xihlangi xi nga vula marito mo ka ma nga talangi
- Xihlangi xi nga hoxa kumbe ku raha swilo swo tanihi bolo

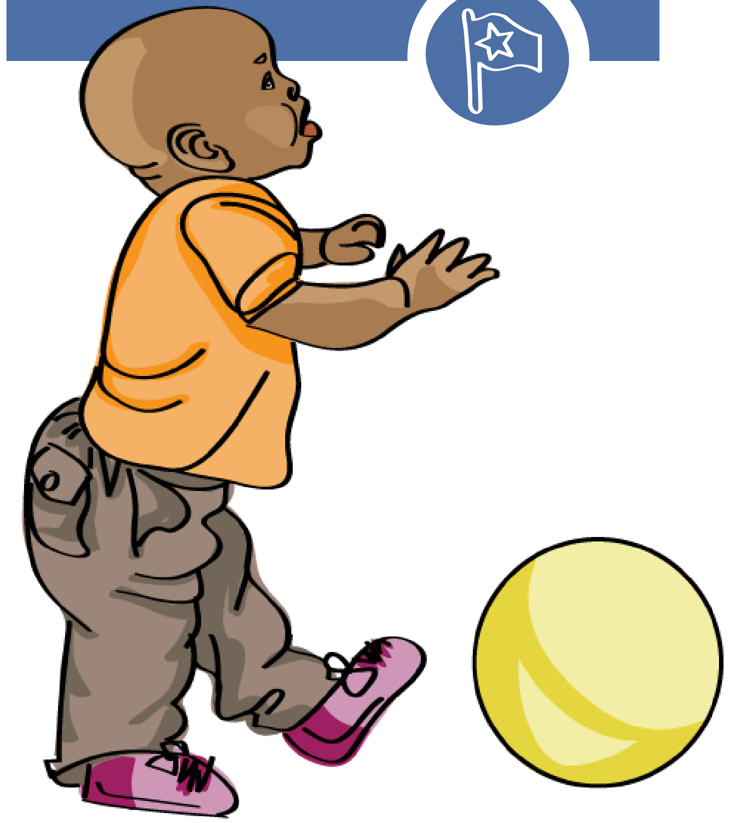

Child Health

- E ba le tsebo ka dika
- Hwetša kalafo ka potlako ya bolwetši

- Look for symptoms
- Seek care immediately for illness

- Languta swikombo
- Lava vutshunguri hi xihatla loko ku ri na mavabyi

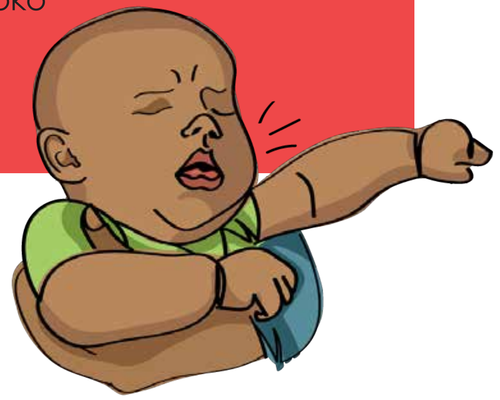

Child Nutrition

- Ngwana o hloka ayone
- Ngwana o swanetše go ja dinawa

- Child needs iron
- Child should eat beans

- N'wana u lava ayoni
- N'wana u fanele ku dya tinyawa

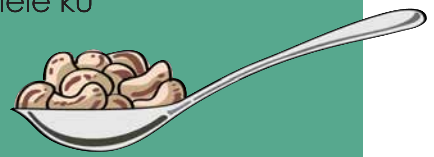

Start

Greet parent and explain purpose of visit.

Ask how parent and child are doing?

Ask caregiver if they have been trying any of the activities since last visit?

Ask if parent can show one of the activities?

Ask if mother/family has any difficulties/challenges in implementing the advice or activities?

Ask if mother/family observed any benefits in implementing the advice or activities?

Ask mother/family if she has any concerns (e.g. child is sick, appears slower to develop)?

Child Health

- Check child for symptoms and demonstrate for mom how she can also check. Encourage mom to pay close attention to child for symptoms.
- Encourage mom to seek care immediately if child has symptoms. Explain that even if symptoms seem non-severe, they may slow the child's development over time. Explain that the clinic has medicines that can help.

Child Nutrition

- The child needs to consume a good amount of iron through their diet in order for their body to develop correctly. In particular, iron will help their brain develop.
- One good source of iron is beans. Encourage mom to incorporate beans into the child's diet as much as possible.

Child Milestones

- Child might say a few words. Demonstrate how mom can check for this.
- Child might throw or kick objects like a ball. Demonstrate how mom can check for this.

Child Activity

- Child is learning more about how to behave appropriately outside the household. Demonstrate for mom how to encourage child's good behaviour.
- Encourage mom to give child hugs and kisses when child demonstrates good behaviour.
- Child is at the age now where they can start to play with other children in the community. Encourage mom to let child spend more time playing with other children of similar age.

Observe parent and child trying activity. If needed demonstrate again to help caregiver

Ask if the caregiver has any questions or concerns?
Arrange next visit date

Thank mother and family. Encourage mother to try implement the advice and activities.

Note any questions asked, action needed, referral required, or things to discuss with supervisor/team leader

End

Child Health

- E ba le tsebo ka ga letšhollo
- Šomiša motswako wa go thibela letšhollo (ORS)

- Look for diarrhea
- Use oral rehydration salt (ORS) solu-tion

- Languta nchuluko
- Tirhisa diripi (oral rehydration salt [ORS])

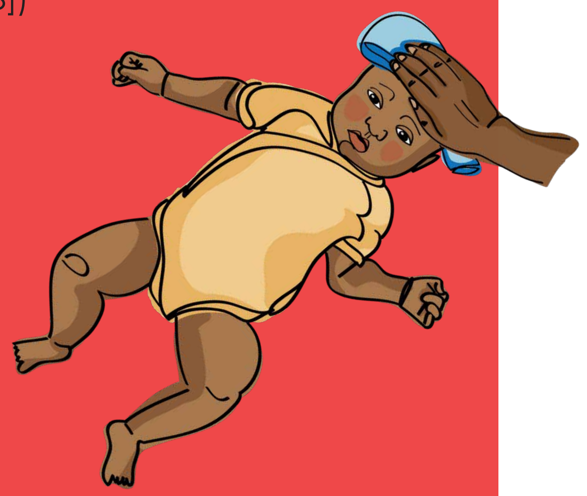

Child Milestones

- Ngwana a ka bolela mantšu a mantši
- Ngwana a ka nwa ka komiki

- Child might say more words
- Child might drink from a cup

- Xihlangi xi nga vula marito yo talanyana
- Xihlangi xi nga n'wa hi khapu

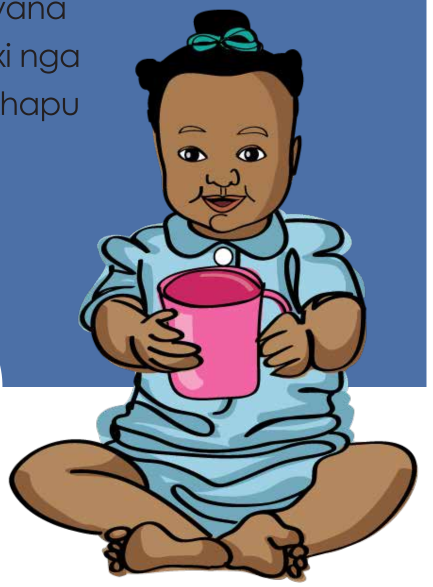

Child Nutrition

- Ngwana o hloka bitamine C
- Ngwana o swanetše go ja dienywa

- Child needs vitamin C
- Child should eat fruits

- N'wana u lava vhitamini C
- N'wana u fanele ku dya mihandzu

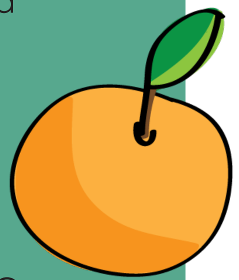

Child Activity

- Kgopela papa gore a raloke le ngwana
- Opelela dikoša le ngwana le papa
- Swara ngwana ge a gakanega

- Have father play with the child
- Sing songs with child and father
- Hold child when she gets frustrated

- Endla papa va tlanga na n'wana
- Yimbelela tinsimu na papa na n'wana
- Tlakula nwana loko a hela mbilu

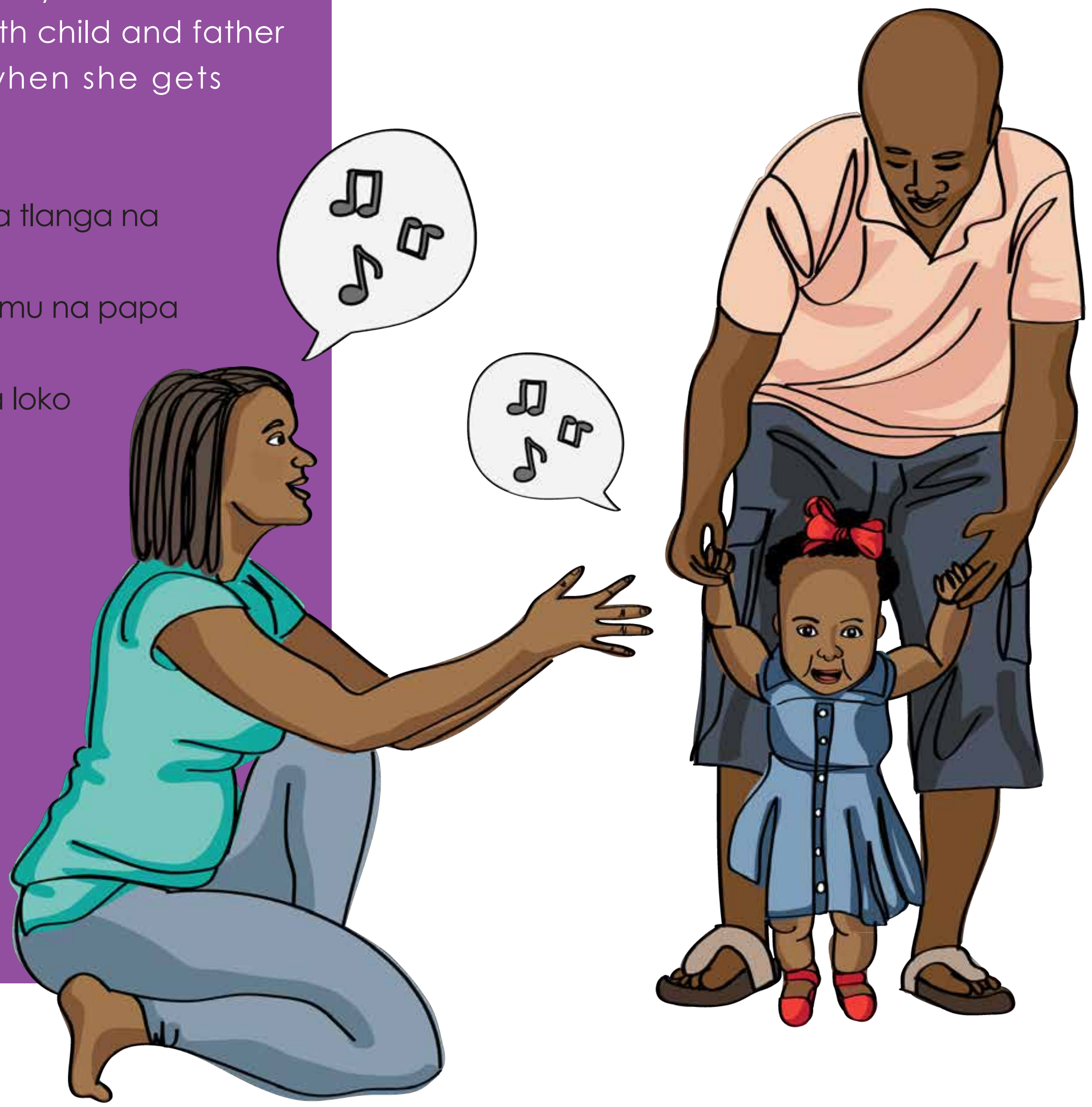

S
E
T

Start

Greet parent and explain purpose of visit.

Ask how parent and child are doing?

Ask caregiver if they have been trying any of the activities since last visit?

Ask if parent can show one of the activities?

Ask if mother/family has any difficulties/challenges in implementing the advice or activities?

Ask if mother/family observed any benefits in implementing the advice or activities?

Ask mother/family if she has any concerns (e.g. child is sick, appears slower to develop)?

Child Health

- As child starts to eat more and more new foods, the risks for diarrhea may increase. Encourage mom to pay close attention to whether child has diarrhea, particularly if baby is showing signs of dehydration or malnutrition due to diarrhea.
- Encourage mom to give child ORS when baby has diarrhea to avoid dehydration. Demonstrate for mom how to make ORS or discuss where she can buy ORS packets nearby.

Child Nutrition

- The child needs to consume a good amount of vitamin C through their diet in order for their body to develop correctly. In particular, vitamin C will help them fight infections.
- One good source of vitamin C is fruit. Encourage mom to incorporate fruit into the child's diet as much as possible.

Child Milestones

- Child might say more and more words each week. Demonstrate how mom can check for this.
- Child might drink from a cup. Demonstrate how mom can check for this.

Child Activity

- Having dad involved as the child grows is important. Encourage dad to play with the child.
- Encourage mom and dad to sing and dance together with child.
- Explain to mom that child cannot easily tell mom what she wants and may get frustrated often. Encourage mom to hold child when child gets frustrated.

Observe parent and child trying activity. If needed demonstrate again to help caregiver

Ask if the caregiver has any questions or concerns?
Arrange next visit date

Thank mother and family. Encourage mother to try implement the advice and activities.

Note any questions asked, action needed, referral required, or things to discuss with supervisor/team leader

End

S
E
T

Child Activity

- Tlhotlheletša ngwana go ra-koloka 'dira ke tshepe"
- Ruta ngwana go abelana le bangwe dilo
- Akara ngwana ge a gakaneg-ile le go thlaloša

- Encourage child to play 'make believe'
- Teach child to share things with others
- Hug child when frustrated and explain

- Hlohletela mhani ku tlanga endla kuni tshembha
- Dyondzisa n'wana ku avela van'wani swilo
- Vukarha n'wana loko a hela mbiu ivi u hlamusela

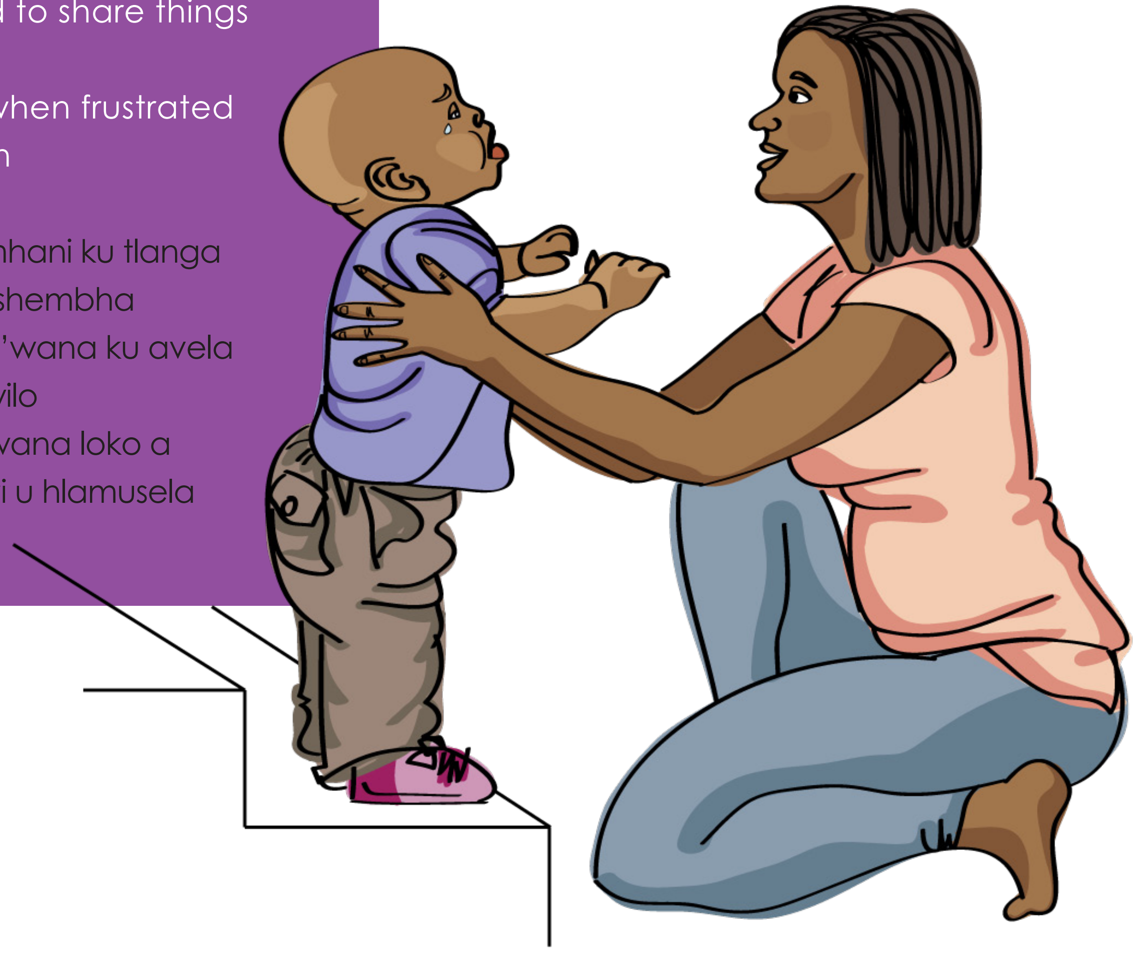

Child Milestones

- Ngwana a ka gopola dikanegelo le dikoša
- Ngwana a ka gakanega gabonolo

- Child might remember stories and songs
- Child might get frustrated easily

- Xihlangi xi nga tsundzuka switori na tinsimu
- Xihlangi xi nga nyangatseka hi ku olova

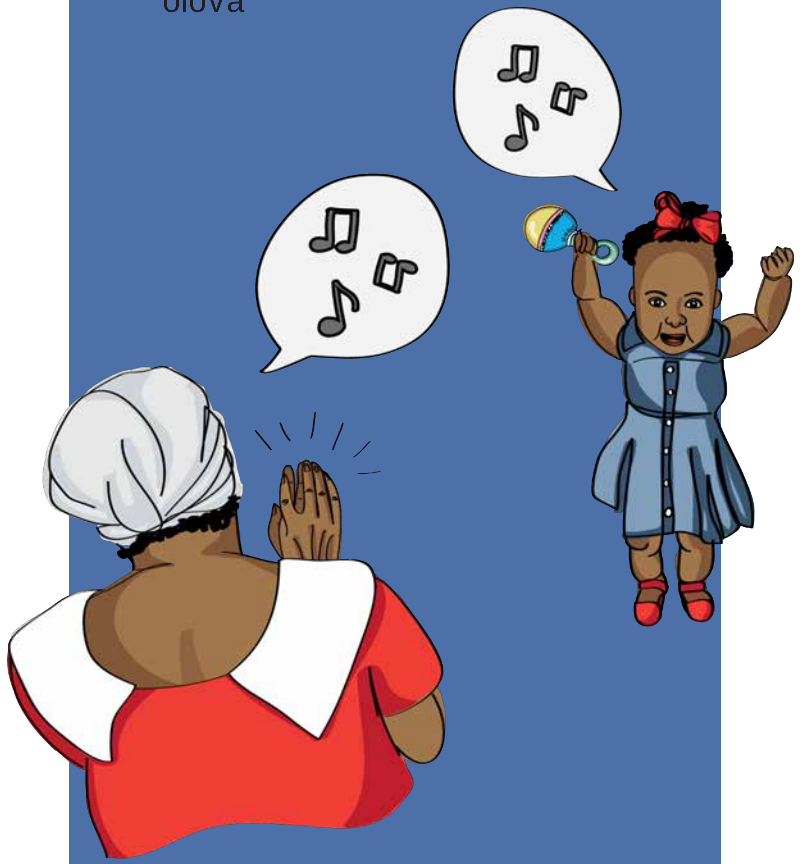

Child Health

- E ba le tsebo ka ga letadi
- Hwetša kalafo ka potlako ge ngwana a na le letadi
- Ngwana o swanetše go khupetšwa ka nete ge a robetše

- Look for fever
- Seek care immediately if child has fever
- Child should sleep under net

- Kamba ku hisa ka miri
- Lava vutshunguri hi xihatla loko n'wana a hisa miri
- N'wana u fanele ku etlela endzeni ka nete

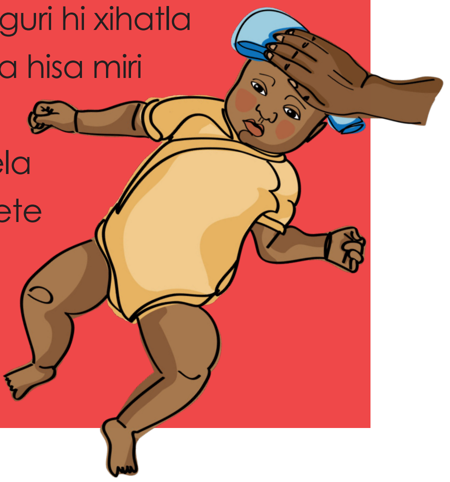

Child Nutrition

- Ngwana o hloka dijo tše ntši
- Leka motswako o moswa

- Child needs many foods
- Try a new recipe

- N'wana u lava swakudya swo tala
- Ringeta rhesipi yintswa

Start

Greet parent and explain purpose of visit.

Ask how parent and child are doing?

Ask caregiver if they have been trying any of the activities since last visit?

Ask if parent can show one of the activities?

Ask if mother/family has any difficulties/challenges in implementing the advice or activities?

Ask if mother/family observed any benefits in implementing the advice or activities?

Ask mother/family if she has any concerns (e.g. child is sick, appears slower to develop)?

Child Health

- Check child for signs of fever and demonstrate for mom how she can also check. Encourage mom to pay close attention to child for fever symptoms.
- Encourage mom to seek care immediately if child has signs of fever. Explain that fever might be a sign of malaria or other infection and that the clinic has medicines that can help.
- Encourage mom to place child under an insecticide treated bednet at night to protect child against mosquitos that might cause malaria.

Child Nutrition

- The child needs a diverse diet with protein, iron, vitamin A, vitamin C and other nutrients to grow and develop properly. Mom should give baby a variety of foods. Discuss adding at least one new food to increase diversity and add protein or nutrients.
- Discuss a new meal that mom can cook for child. Ask about child's preferences for food and make tailored suggestions.

Child Milestones

- Child might remember stories and songs and start to join in more as mom reads and sings. Demonstrate how mom can check for this.
- Child might get frustrated easily when things don't go their way. Explain to mom that this is natural.

Child Activity

- Playing 'make believe' is an important way for the child to develop their imagination. Demonstrate for mom how she can encourage child to make believe, for example have a tea party.
- Teach child to share things with others and praise child for sharing
- Explain to mom that child cannot easily tell mom what she wants and may get frustrated often. Encourage mom to hold child when child gets frustrated.

Observe parent and child trying activity. If needed demonstrate again to help caregiver

Ask if the caregiver has any questions or concerns?
Arrange next visit date

Thank mother and family. Encourage mother to try implement the advice and activities.

Note any questions asked, action needed, referral required, or things to discuss with supervisor/team leader

End

S
E
T

Child Milestones

- Ngwana a ka šupa go bontšha se a se nyakago
- Ngwana a ka kgorometša dilo

- Child might point to show what they want
- Child might push things

- Xihlangi xi nga kombetela leswi xi swi lavaka
- Xihlangi xi nga susumeta swilo

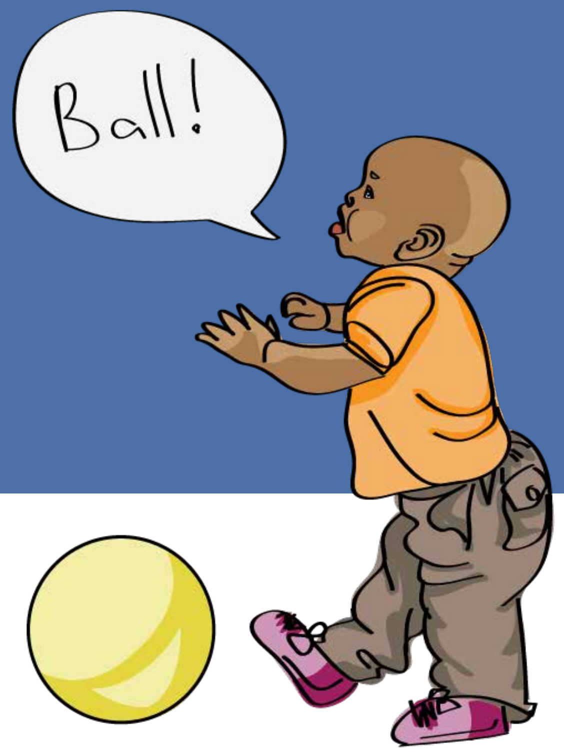

Child Health

- E ba le tsebo ka ga go gohlola le bothata bja go hema
- Hwetša kalafo ka potlako ge lesea le na le bothata bja go hema

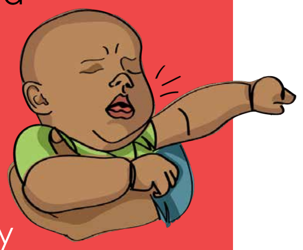

- Look for cough and difficulty breathing
- Seek care immediately if baby has difficulty breathing

- Kamba ku khohlola kumbe ku tik-eriwa ka ku hefemula
- Lava ku pfuniwa hi xihatla loko n'wana a tikeriwa ku hefemula

Child Nutrition

- Phepo e ama go gola ga ngwana
- Dino tša swikiri le malekere ga se tša lokela ngwana

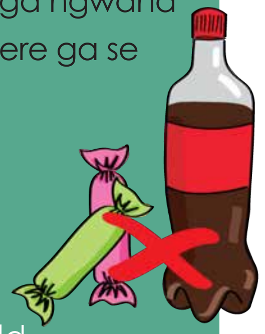

- Nutrition affects child development
- Sweet drinks and candy don't help child

- Tinyutiriyente ti khumba makulele ya n'wana
- Swinwiwa swo nyanganya na tikhendi a swi pfuni xihlangi

Child Activity

- Leka go hlalosetja ngwana maikutlo.
- O seka tlhabela ngwana mashata ge a kwatile
- Reta ngwana go ba a dirile selo se se botse

- Try to explain emotions to child
- Don't yell at child when child is upset
- Praise child for doing something good

- Ringeta ku hlamusela matitwelo eka n'wana
- Unga karhiheli nwana loko a kwatili
- Themendhela n'wana loko a endla swilo swa kahle

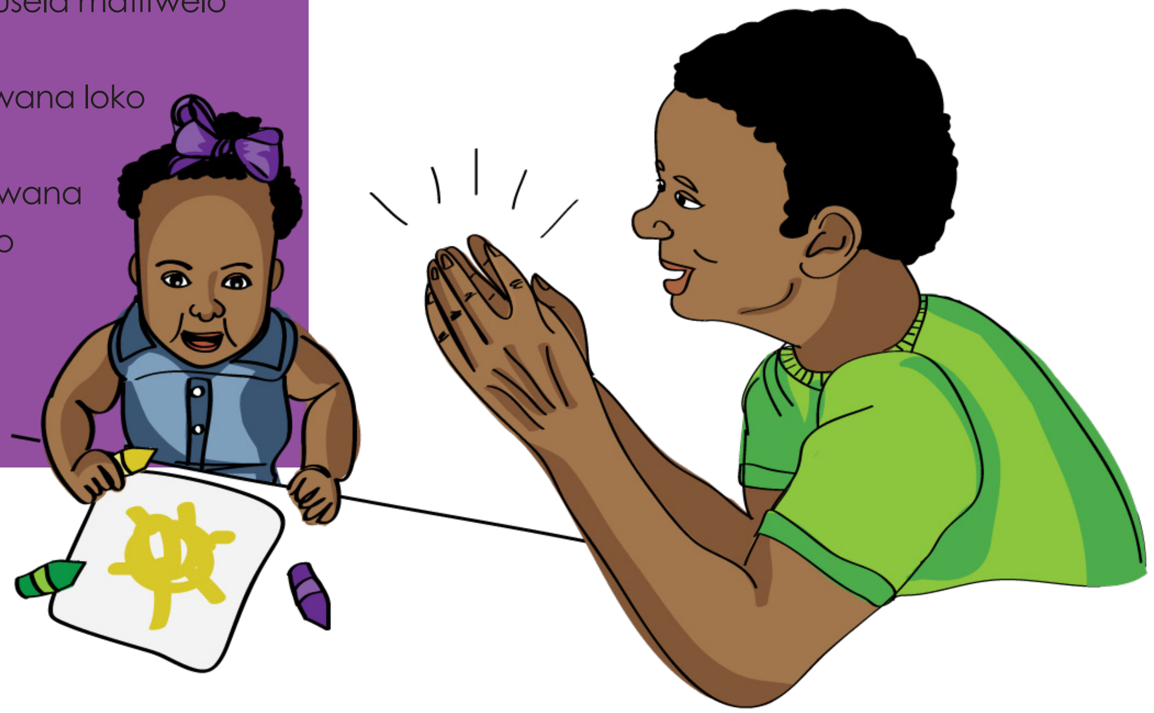

Start

Greet parent and explain purpose of visit.

Ask how parent and child are doing?

Ask caregiver if they have been trying any of the activities since last visit?

Ask if parent can show one of the activities?

Ask if mother/family has any difficulties/challenges in implementing the advice or activities?

Ask if mother/family observed any benefits in implementing the advice or activities?

Ask mother/family if she has any concerns (e.g. child is sick, appears slower to develop)?

Child Health

- Check baby for signs of cough and respiratory infection and demonstrate for mom how she can also check. Encourage mom to pay close attention to baby for respiratory symptoms.
- Encourage mom to seek care immediately if baby has danger signs of respiratory infection, including fast breathing and wheezing sounds. Explain that symptoms may be a sign of pneumonia or other infection and that the clinic has medicines that can help.

Child Nutrition

- Child's nutrition affects physical growth and development. It is important that child has enough protein and micronutrients in their diet. If they do not, their growth may be slowed.
- While sweet drinks and candy might sooth the child for a short time, they do not at all help the child develop over the long term. Discourage mom from giving child sweets and make it clear that sweets should not be a substitute for the nutritious food the child needs.

Child Milestones

- Child might point at the things that they want. Demonstrate how mom can check for this.
- Child might push things around the house. Demonstrate how mom can check for this.

Child Activity

- Try to explain emotions to your child, for example: "You are upset because… other children are upset because…"
- Yelling at the child when they are upset will not help them learn better behaviours. Encourage mom to calmly explain rules to child when child is upset.
- Showing the child love with positive verbal praise (e.g., "good job!") will help the child learn good behaviours. Encourage mom to give verbal praise when the child does something good.

Observe parent and child trying activity. If needed demonstrate again to help caregiver

Ask if the caregiver has any questions or concerns?
Arrange next visit date

Thank mother and family. Encourage mother to try implement the advice and activities.

Note any questions asked, action needed, referral required, or things to discuss with supervisor/team leader

End

S
E
T

Child Activity

- Dumelela ngwana go raloka le bana bangwe ba mengwaga ya go lekana le ya gwe
- Seka tlhabela ngwana mashata ge a nyamile
- Reta ngwana ga ntši ka mo o kgonago

- Let child play with other children of similar age
- Don't yell at child when child is upset
- Praise child as often as you can

- Endla n'wana a tlanga na vana vanwani vo ringana na yena
- Unga kariheli n'wana loko a kwatili
- Ndhundhuzela n'wana hi minkarhi hinkwayo

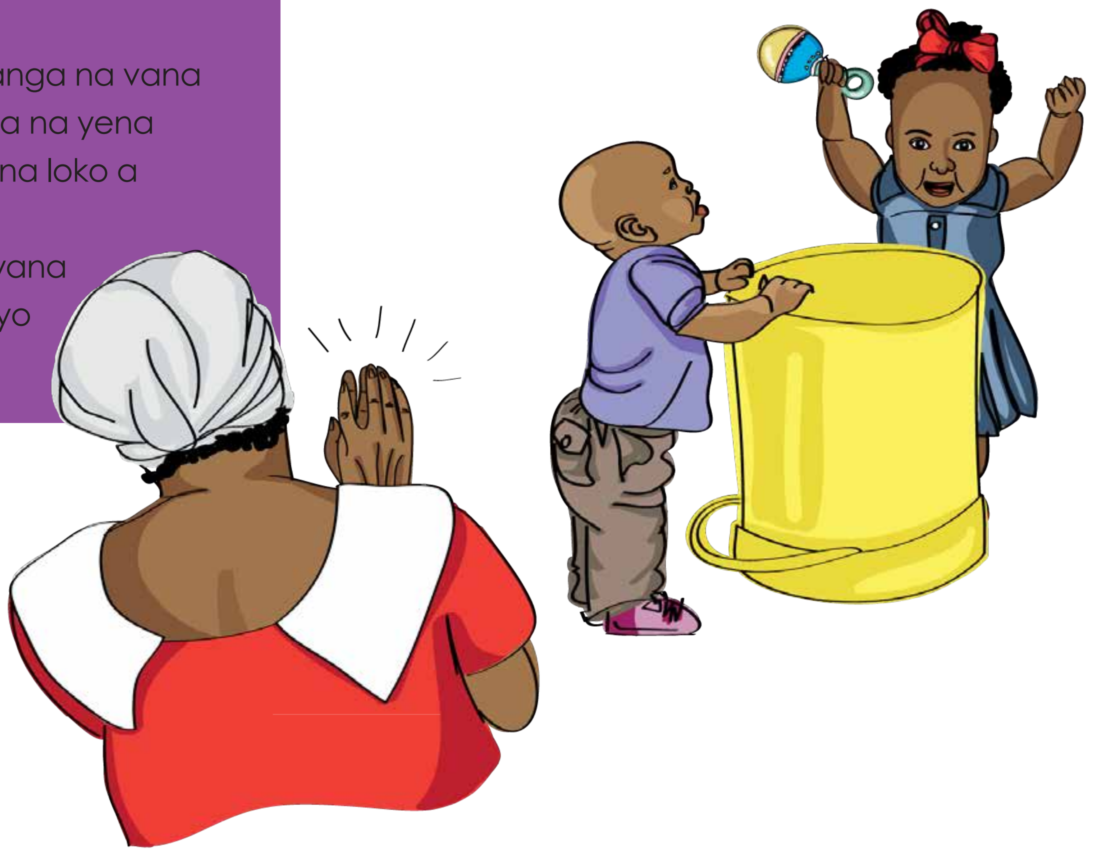

Child Milestones

- Ngwana a ka sepela gabotse ka noši
- Ngwana a ka ja ka noši ka diatla tša gagwe

- Child might walk well on own
- Child might eat with hands on own

- Xihlangi xi nga kota ku tifambela kahle
- Xihlangi xi nga kota ku tidyisa hi swandla

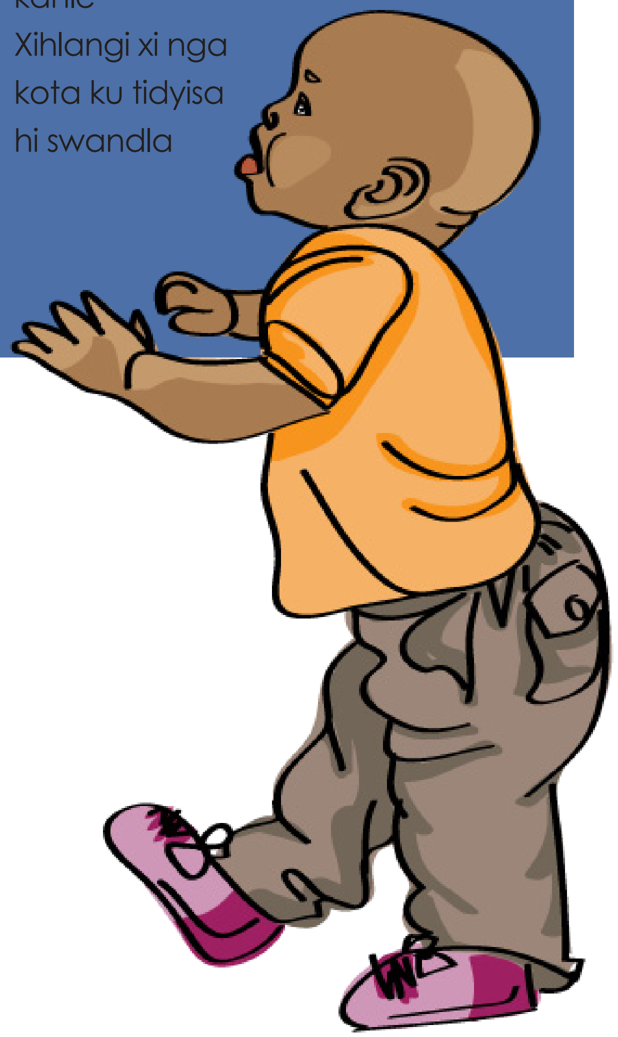

Child Health

- Ditšhwaana tša thibela malwetši di šireletša ngwana
- Maphelo a ama kgodišo ya ngwana

- Vaccines protect child
- Health affects child's development

- Misawutiso yi sirhelela xihlangi
- Rihanyu ri khumba makulele ya n'wana

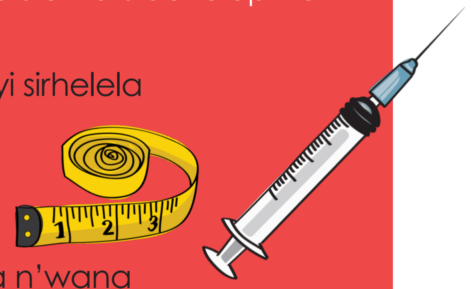

Child Nutrition

- Phepo e ama go gola ga ngwana
- Ngwana o hloka protheine
- Ngwana o swanetše go ja dinawa le mae

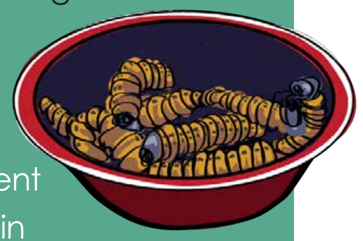

- Nutrition affects child's development
- Child needs protein
- Child should eat beans and eggs

- Tinyutiriyente ti khumba makulele ya n'wana
- N'wana u lava phurotheyini
- N'wana u fanele ku dya tinyawa na matandza

Start

Greet parent and explain purpose of visit.

Ask how parent and child are doing?

Ask caregiver if they have been trying any of the activities since last visit?

Ask if parent can show one of the activities?

Ask if mother/family has any difficulties/challenges in implementing the advice or activities?

Ask if mother/family observed any benefits in implementing the advice or activities?

Ask mother/family if she has any concerns (e.g. child is sick, appears slower to develop)?

Child Health

- At 18 months old, children should receive measles2 and DTaP-IPV/Hib4. Confirm that the child has received these vaccines. If not yet received, encourage mom to bring child to the clinic for these vaccines immediately.
- Child's health affects physical growth and development. It is important that mom seeks care for child when child has symptoms of illness. Even if symptoms do not seem too serious, untreated infections may slow the child's development over time. Mom should take child to the clinic for a general checkup every three months or so. Ask mom if child has been checked recently, and if not encourage mom to take child to clinic for a checkup.

Child Nutrition

- Child's nutrition affects physical growth and development. It is important that child has enough protein and micronutrients in their diet. If they do not, their growth may be slowed.
- The child needs to consume a good amount of protein through their diet in order for their body to develop properly. Protein will help them grow taller and stronger.
- A few good sources of protein are beans and eggs. Encourage mom to incorporate beans or eggs into the child's diet as much as possible.

Child Milestones

- Child might walk well on own. Demonstrate how mom can check for this.
- Child might eat with hands on own. Demonstrate how mom can check for this.

Child Activity

- Child is at the age now where they can start to play with other children in the community. Encourage mom to let child spend more time playing with other children of similar age.
- Yelling at the child when they are upset will not help them learn better behaviours. Encourage mom to calmly explain rules to child when child is upset.
- Showing the child love with positive verbal praise (e.g., "good job!") will help the child learn good behaviours. Encourage mom to give verbal praise as often as possible.

Observe parent and child trying activity. If needed demonstrate again to help caregiver

Ask if the caregiver has any questions or concerns?
Arrange next visit date

Thank mother and family. Encourage mother to try implement the advice and activities.

Note any questions asked, action needed, referral required, or things to discuss with supervisor/team leader

End

S
E
T

Child Milestones

- Ngwana a ka robala nako ye kopana
- Ngwana a ka leka go tshela mell-wane le go se obamele melao

- Child might sleep less
- Child might test limits and break rules

- Swi nga endleka xihlangi xi nga ha etleli ngopfu
- Xihlangi xi nga endla leswi u xi byelaka leswaku xi nga swi endli na ku tlula milawu

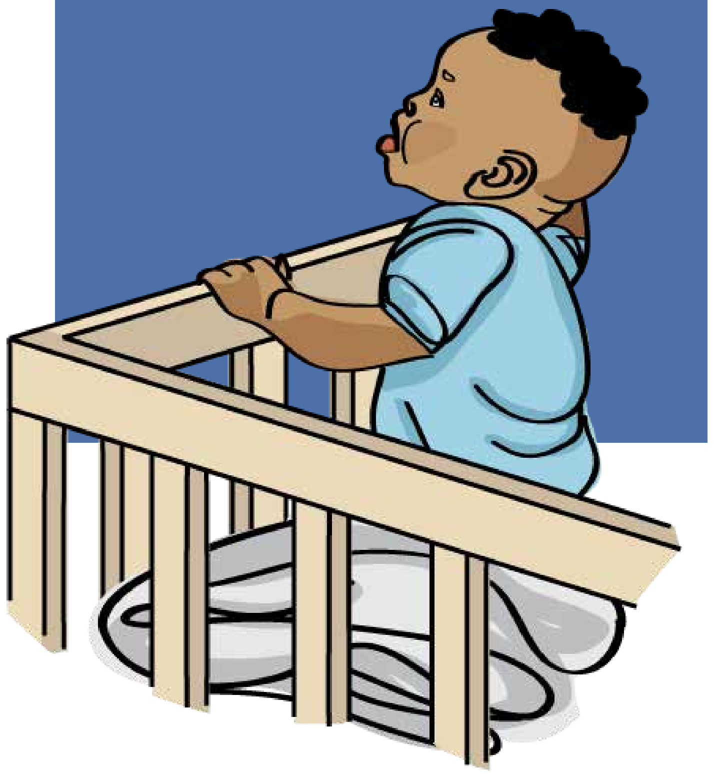

Child Health

- Ngwana o swanetše go hlapa meno ka poratšhe letšatši le lengwe le le lengwe
- E ba le tsebo ka dika
- Hwetša kalafo ka potlako ya bolwetši

- Child should brush teeth every day
- Look for symptoms
- Seek care immediately for illness

- Xihlangi xi fanele ku buracha meno masiku hinkwawo
- Languta swikombo
- Lava vutshunguri hi xihatla loko ku ri na mavabyi

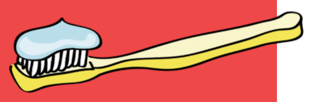

Child Nutrition

- Ngwana o hloka bitamine A
- Ngwana o swanetše go ja mero-go ye metala ya mahlare

- Child needs vitamin A
- Child should eat leafy green vegetables

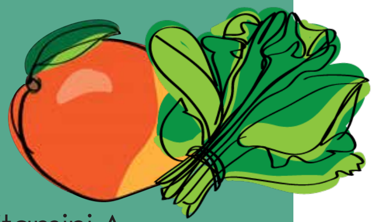

- N'wana u lava vhitamini A
- N'wana u fanele ku dya matsavu yo tsakama

Child Activity

- Nyakišiša thaloko yeo ngwana a e ratago
- Reta ngwana ge a latelela melao
- Se šomiši mokgwa wa go kweša ngwana bothloko

- Find out child's favourite activ-ity
- Praise child whenever child fol-lows rules
- Do not use physical punishment

- Kuma leswi n'wana aswi tsakelaku ngopfu loko ka mintlangu
- Ndhundhuzela n'wana loko a landelela milawu
- Unga tirhisi ku ba loko u tshinya n'wana

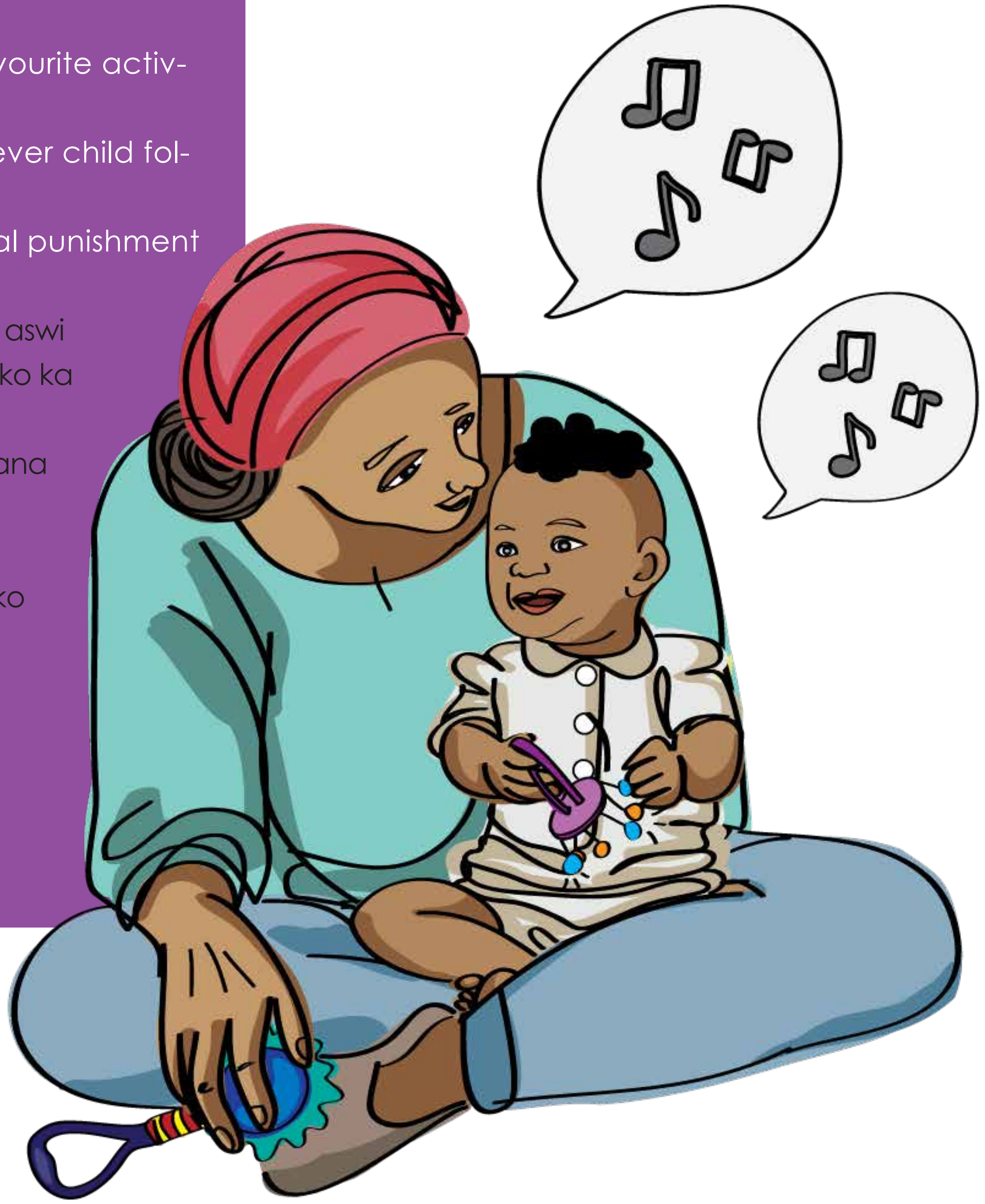

Start

Greet parent and explain purpose of visit.

Ask how parent and child are doing?

Ask caregiver if they have been trying any of the activities since last visit?

Ask if parent can show one of the activities?

Ask if mother/family has any difficulties/challenges in implementing the advice or activities?

Ask if mother/family observed any benefits in implementing the advice or activities?

Ask mother/family if she has any concerns (e.g. child is sick, appears slower to develop)?

Child Health

- Child should start to brush teeth every day if they haven't already. Teach child to brush teeth and explain that it is important to brush every day.
- Check child for symptoms and demonstrate for mom how she can also check. Encourage mom to pay close attention to child for symptoms.
- Encourage mom to seek care immediately if child has symptoms. Explain that even if symptoms seem non-severe, they may slow the child's development over time. Explain that the clinic has medicines that can help.

Child Nutrition

- The child needs to consume a good amount of vitamin A through their diet in order for their body to develop correctly. In particular, vitamin A will help them fight infections.
- One good source of vitamin A is leafy green vegetables. Encourage mom to incorporate leafy green vegetables into the child's diet as much as possible.

Child Milestones

- Child might sleep less and have a harder time taking naps and sleeping at night. Explain to mom that this is okay.
- Child might test the limits that mom sets and intentionally break rules. Explain to mom that this is okay and the child is just learning the boundaries of normal behaviour.

Child Activity

- Child is starting to develop individuality and learning what she likes and does not like. Encourage mom to learn what child's favourite activity is and to do this activity with child often.
- Encourage mom to give child positive verbal praise whenever child follows the rules.
- Explain to mom that it is normal for child to break rules. Explain that physical punishment will not help child learn good behaviours and instead will hurt child and damage relationship.

Observe parent and child trying activity. If needed demonstrate again to help caregiver

Ask if the caregiver has any questions or concerns?
Arrange next visit date

Thank mother and family. Encourage mother to try implement the advice and activities.

Note any questions asked, action needed, referral required, or things to discuss with supervisor/team leader

End

S
E
T

Child Activity

- Dumelela ngwana go raloka le bana ba mengwaga ya go lekana ya gwe
- Kgopela ngwana a go botse gore o nyamishitse ke eng
- Kgopela ngwana a go botse dilo tse a sa dirateng

- Let child play with other children of similar age
- Ask child to tell you why they are upset
- Ask child to tell you things they do not like

- Pfumelela n'wana a tlanga na vana vo ringana na yena
- Vutisa n'wana ku ri himhaka yini a kwatili
- Vutisa n'wana a ku byela swilo leswi anga swi tsakeleku

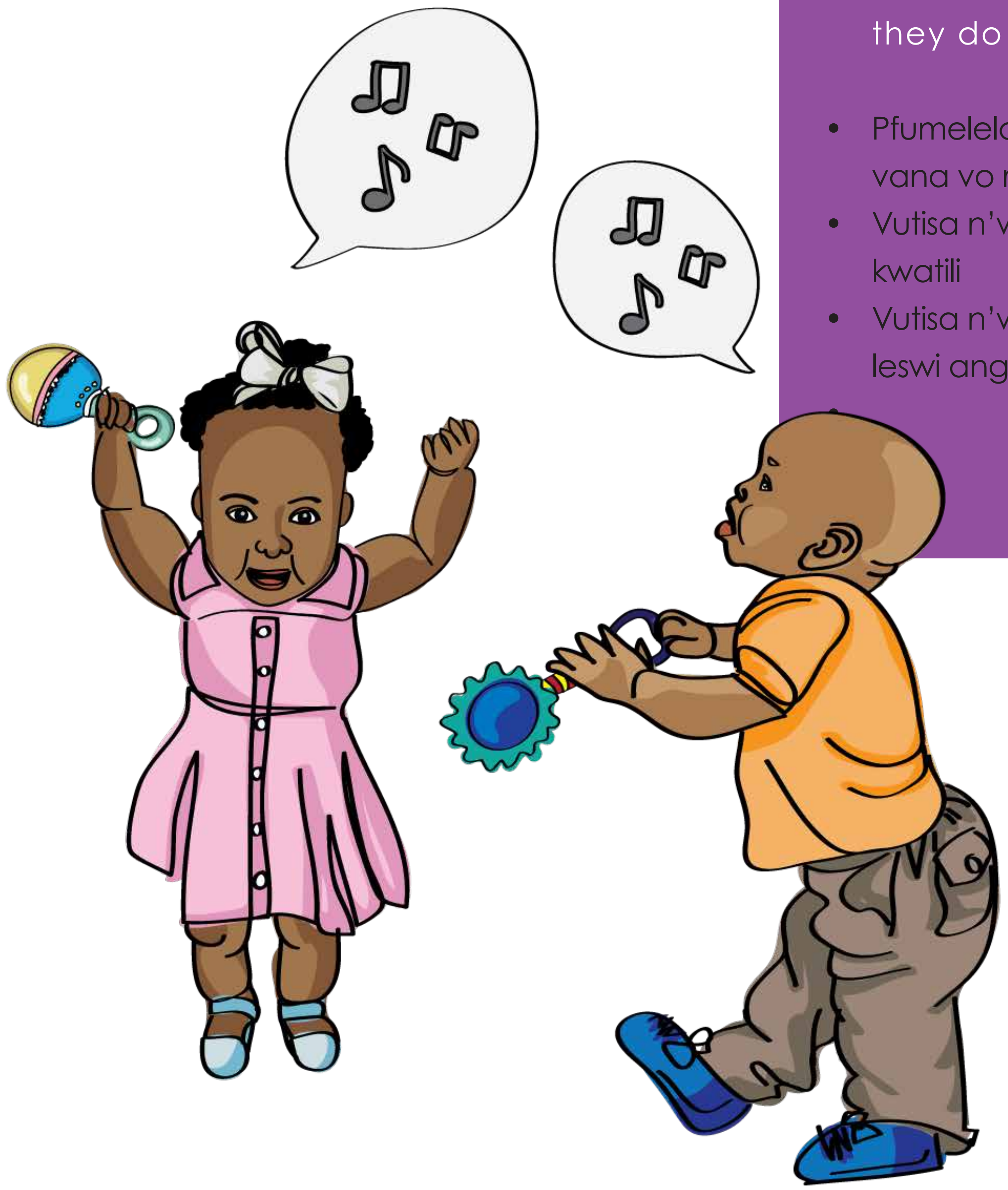

Child Milestones

- Ngwana a ka nyaka go ikema ka noši
- Ngwana a ka namela ditepisi le go tshela

- Child might become more independent
- Child might walk stairs and jump

- Xihlangi xi nga ka xi nga ha lavi ku pfuniwa eka swotala
- Xihlangi xi nga khandziya switepisi na ku tlula

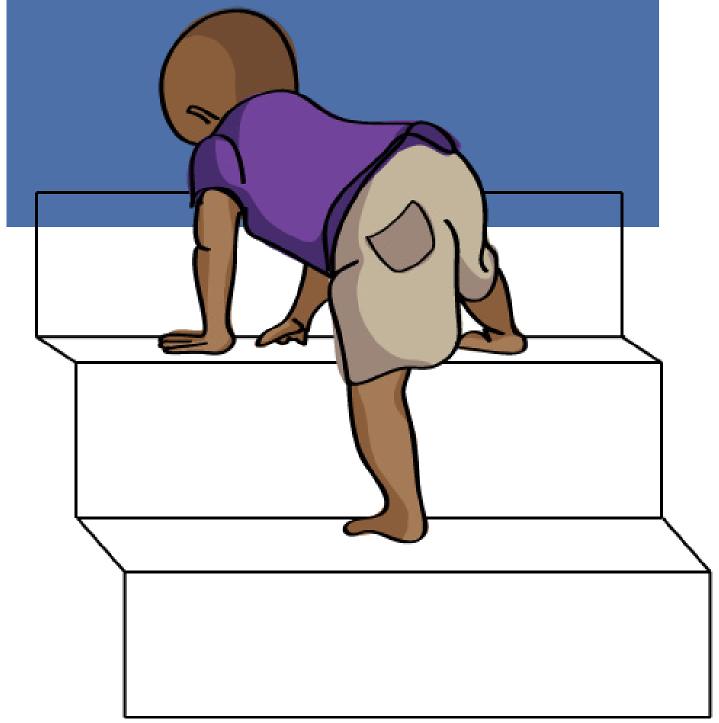

Child Health

- E ba le tsebo ka ga letšhollo
- Šomiša motswako wa go thibela letšhollo (ORS)

- Look for diarrhea
- Use oral rehydration salt (ORS) solution

- Languta nchuluko
- Tirhisa diripi (oral rehydration salt [ORS])

Child Nutrition

- Ngwana o hloka ayone
- Ngwana o swanetše go ja dinawa

- Child needs iron
- Child should eat beans

- N'wana u lava ayoni
- N'wana u fanele ku dya tinyawa

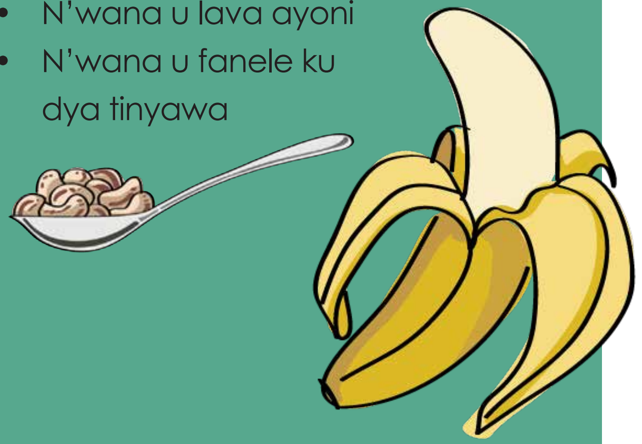

Start

Greet parent and explain purpose of visit.

Ask how parent and child are doing?

Ask caregiver if they have been trying any of the activities since last visit?

Ask if parent can show one of the activities?

Ask if mother/family has any difficulties/challenges in implementing the advice or activities?

Ask if mother/family observed any benefits in implementing the advice or activities?

Ask mother/family if she has any concerns (e.g. child is sick, appears slower to develop)?

Child Health

- As child starts to eat more and more new foods, the risks for diarrhea may increase. Encourage mom to pay close attention to whether child has diarrhea, particularly if baby is showing signs of dehydration or malnutrition due to diarrhea.
- Encourage mom to give child ORS when baby has diarrhea to avoid dehydration. Demonstrate for mom how to make ORS or discuss where she can buy ORS packets nearby.

Child Nutrition

- The child needs to consume a good amount of iron through their diet in order for their body to develop correctly. In particular, iron will help their brain develop.
- One good source of iron is beans. Encourage mom to incorporate beans into the child's diet as much as possible.

Child Milestones

- Child might start to do more activities by themselves and be more independent. Explain to mom that this is okay
- Child might learn to walk up and down stairs and to jump. Demonstrate how mom can check for this.

Child Activity

- Child is at the age now where they can start to play with other children in the community. Encourage mom to let child spend more time playing with other children of similar age.
- Child is at the age where they can start to talk about their emotions. Encourage mom to ask the child why they are upset and talk about their emotions.
- Being able to talk about the things they do not like will help the child feel supported. Encourage mom to ask the child to tell her about things they do not like.

Observe parent and child trying activity. If needed demonstrate again to help caregiver

Ask if the caregiver has any questions or concerns?
Arrange next visit date

Thank mother and family. Encourage mother to try implement the advice and activities.

Note any questions asked, action needed, referral required, or things to discuss with supervisor/team leader

End

S E T

Child Milestones

- Ngwana a ka namela le go fologa se sengwe le se sengwe
- Ngwana a ka raloka thwii le bana ba bangwe

- Child might climb up and down everything
- Child might interact directly with other children

- Xihlangi xi nga khandziya no chika eka nchumu wu'wana na wun'wana
- Xihlangi xi nga tlanga na vana van'wana

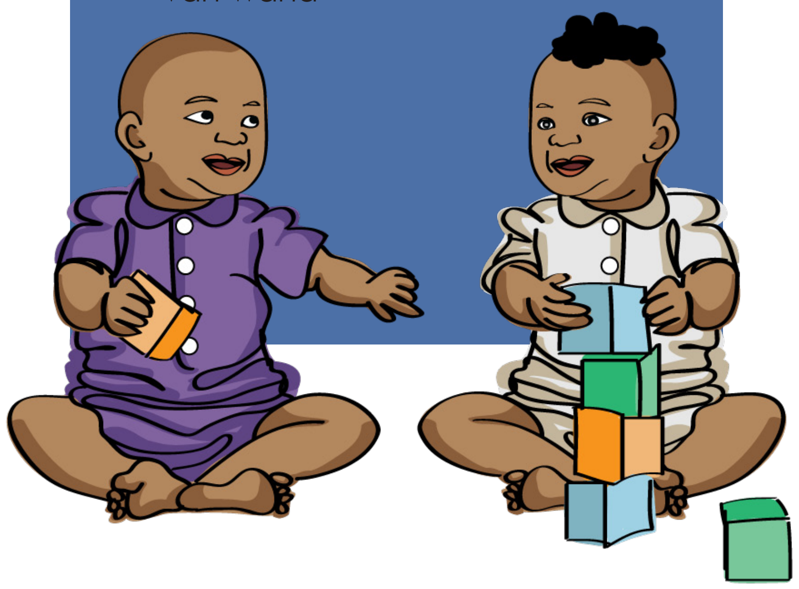

Child Health

- E ba le tsebo ka ga letadi
- Hwetša kalafo ka potlako ge ngwana a na le letadi
- Ngwana o swanetše go khupetšwa ka nete ge a robetše

- Look for fever
- Seek care immediately if child has fever
- Child should sleep under net

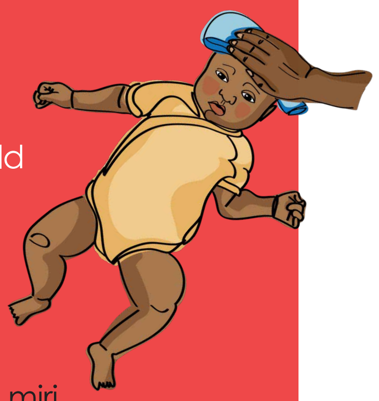

- Kamba ku hisa ka miri
- Lava vutshunguri hi xihatla loko n'wana a hisa miri
- N'wana u fanele ku etlela endzeni ka nete

Child Nutrition

- Ngwana o hloka bitamine C
- Ngwana o swanetše go ja dienywa

- Child needs vitamin C
- Child should eat fruits

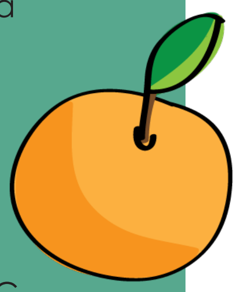

- N'wana u lava vhitamini C
- N'wana u fanele ku dya mihandzu

Child Activity

- Dumelela ngwana go dira thaloko yeo a e ratago
- Thuša ngwana go kwišiša melao
- Bolela ka batho bao ba ratang ngwana

- Let child do favourite activity
- Help child understand rules
- Talk about people who love the child

- N'wana a endla leswi aswi rhandzaka
- Pfuna n'wana kuri atwisisa milawu
- Valavula hi vanhu lava rhandzaku n'wana

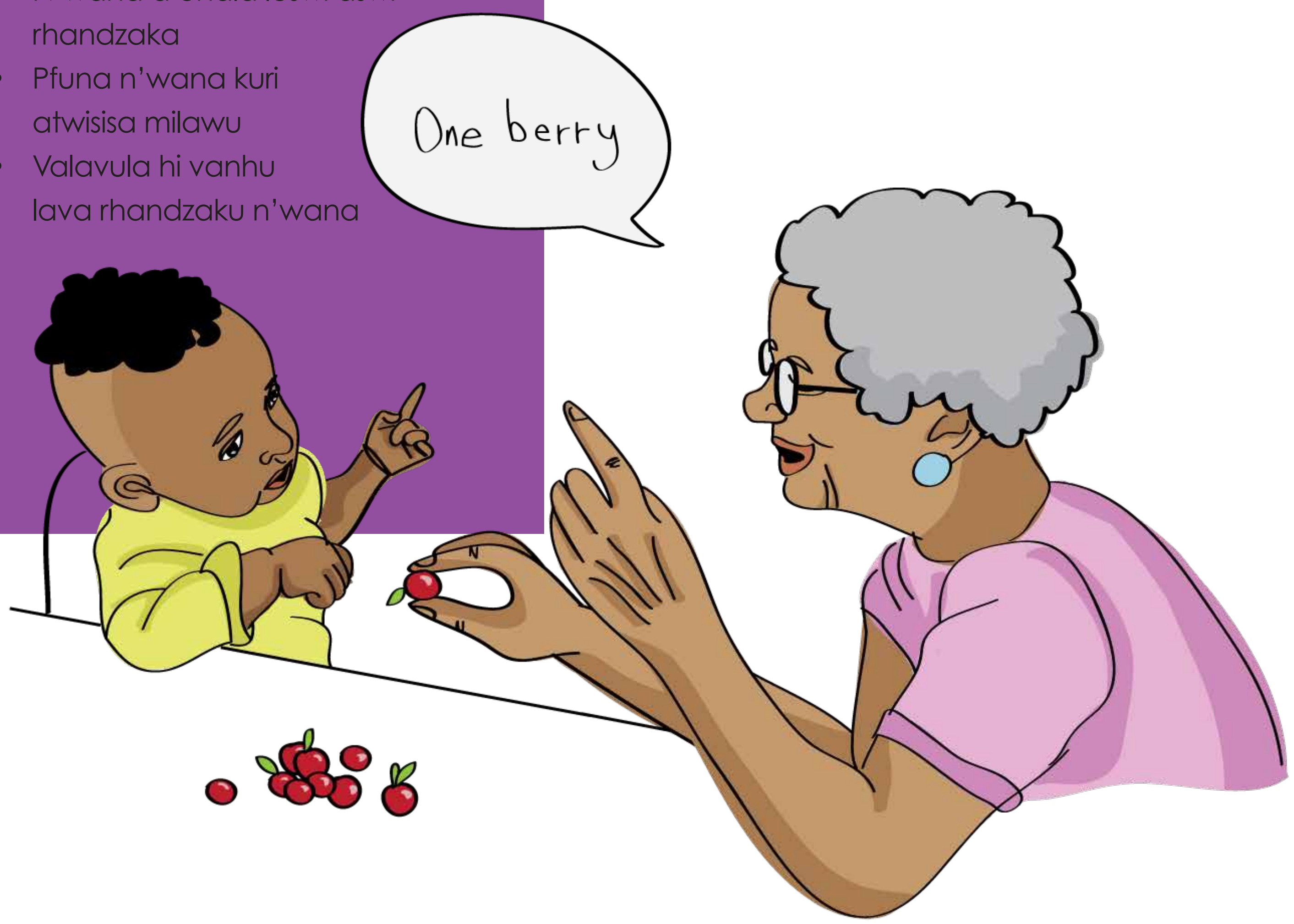

Start

Greet parent and explain purpose of visit.

Ask how parent and child are doing?

Ask caregiver if they have been trying any of the activities since last visit?

Ask if parent can show one of the activities?

Ask if mother/family has any difficulties/challenges in implementing the advice or activities?

Ask if mother/family observed any benefits in implementing the advice or activities?

Ask mother/family if she has any concerns (e.g. child is sick, appears slower to develop)?

Child Health

- Check child for signs of fever and demonstrate for mom how she can also check. Encourage mom to pay close attention to child for fever symptoms.
- Encourage mom to seek care immediately if child has signs of fever. Explain that fever might be a sign of malaria or other infection and that the clinic has medicines that can help.
- Encourage mom to place child under an insecticide treated bednet at night to protect child against mosquitos that might cause malaria.

Child Nutrition

- The child needs to consume a good amount of vitamin C through their diet in order for their body to develop correctly. In particular, vitamin C will help them fight infections.
- One good source of vitamin C is fruit. Encourage mom to incorporate fruit into the child's diet as much as possible.

Child Milestones

- Child might climb on everything, including furniture in the house. Check with mom that the home is safe for the child.
- Child might start to interact more with other children in the village. Child might fight with other children sometimes. Explain to mom that this is natural as the child learns how to interact.

Child Activity

- Child is starting to develop individuality and learning what she likes and does not like. Encourage mom to learn what child's favourite activity is and to do this activity with child often.
- Child needs to be told rules many times before they will understand them completely. Encourage mom to help child understand and follow rules in a positive way.
- Talking to the child about all of the people who love them will make the child feel secure and loved. Encourage mom to tell the child about the people in their life who love them.

Observe parent and child trying activity. If needed demonstrate again to help caregiver

Ask if the caregiver has any questions or concerns?
Arrange next visit date

Thank mother and family. Encourage mother to try implement the advice and activities.

Note any questions asked, action needed, referral required, or things to discuss with supervisor/team leader

End

S
E
T

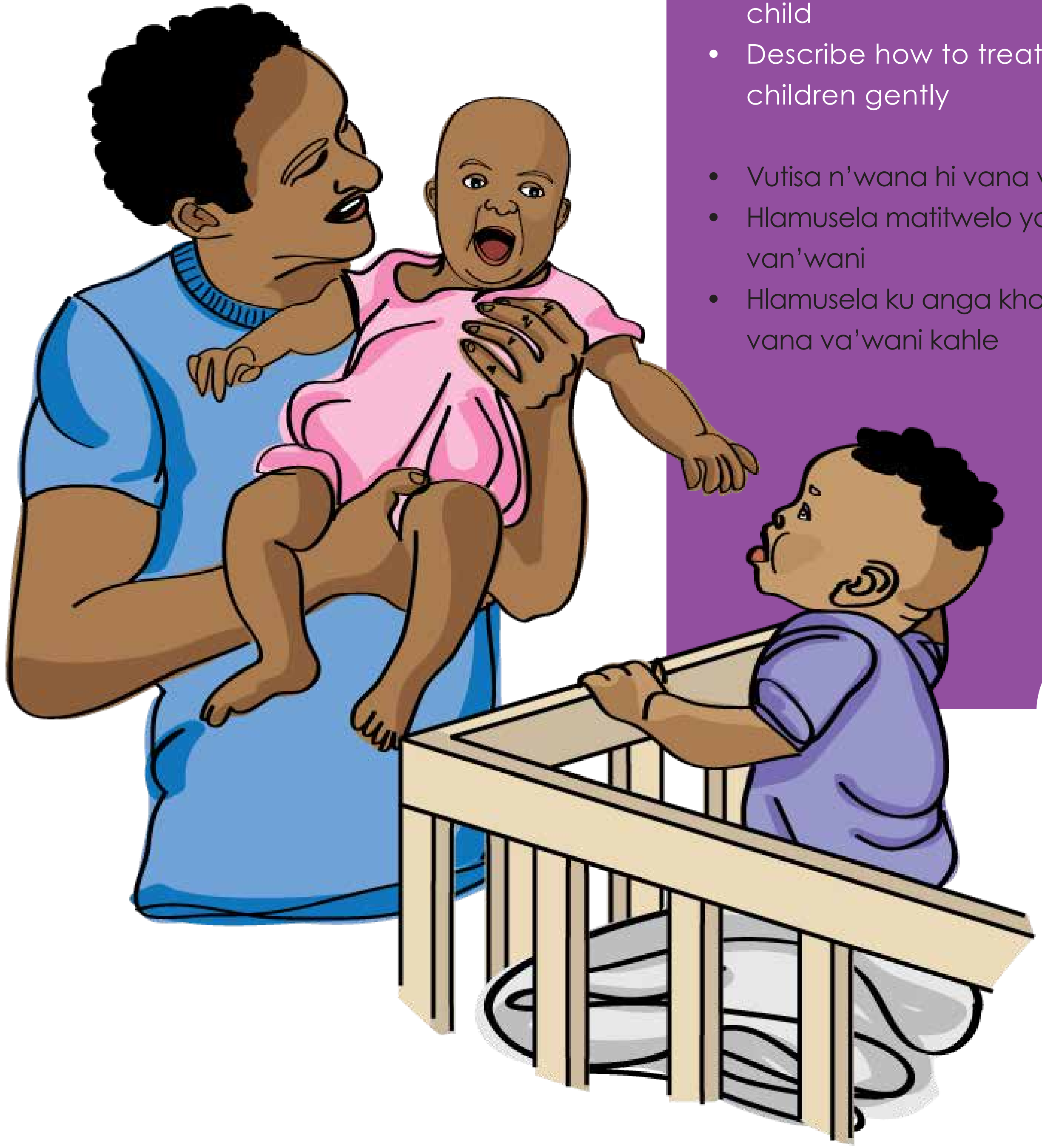

Child Activity

- Botjisha ngwana ka bana ba bangwe
- Hlalosetsa ngwana ka maikutlo a ban aba bangwe
- Laodishetsa ngwana gore a sware ban aba bangwe bjang

- Ask child about other children
- Explain emotions of others to child
- Describe how to treat other children gently

- Vutisa n'wana hi vana van'wani
- Hlamusela matitwelo ya vana van'wani
- Hlamusela ku anga khoma njhani vana va'wani kahle

Child Milestones

- Ngwana a ka bopa mafoko a bonolo
- Ngwana a ka ipshina ka go bina

- Child might make simple sen-tences
- Child might enjoy dancing

- Xihlangi xi nga vumba swivulwa swo olova
- Xihlangi xi nga tsakisiwa hi ku cina

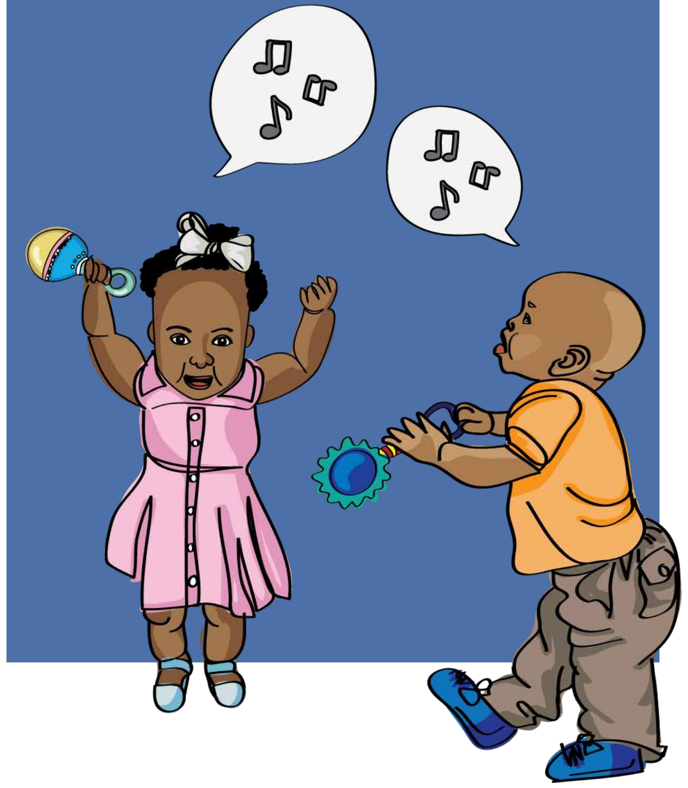

Child Health

- E ba le tsebo ka ga go gohlola le bothata bja go hema
- Hwetša kalafo ka potlako ge lesea le na le bothata bja go hema

- Look for cough and difficulty breathing
- Seek care immediately if baby has difficulty breathing

- Kamba ku khohlola kumbe ku tikeriwa ka ku hefemula
- Lava ku pfuniwa hi xihatla loko n'wana a tikeriwa ku hefemula

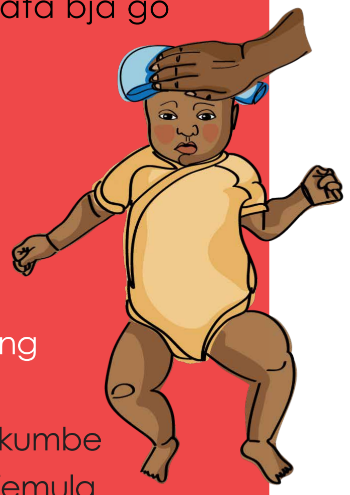

Child Nutrition

- Ngwana o hloka dijo tše ntši
- Leka motswako o moswa

- Child needs many foods
- Try a new recipe

- N'wana u lava swakudya swo tala
- Ringeta rhesipi yintswa

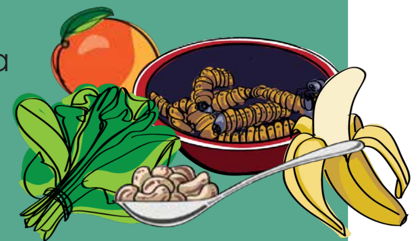

Start

Greet parent and explain purpose of visit.

Ask how parent and child are doing?

Ask caregiver if they have been trying any of the activities since last visit?

Ask if parent can show one of the activities?

Ask if mother/family has any difficulties/challenges in implementing the advice or activities?

Ask if mother/family observed any benefits in implementing the advice or activities?

Ask mother/family if she has any concerns (e.g. child is sick, appears slower to develop)?

Child Health

- Check baby for signs of cough and respiratory infection and demonstrate for mom how she can also check. Encourage mom to pay close attention to baby for respiratory symptoms.
- Encourage mom to seek care immediately if baby has danger signs of respiratory infection, including fast breathing and wheezing sounds. Explain that symptoms may be a sign of pneumonia or other infection and that the clinic has medicines that can help.

Child Nutrition

- The child needs a diverse diet with protein, iron, vitamin A, vitamin C and other nutrients to grow and develop properly. Mom should give baby a variety of foods. Discuss adding at least one new food to increase diversity and add protein or nutrients.
- Discuss a new meal that mom can cook for child. Ask about child's preferences for food and make tailored suggestions.

Child Milestones

- Child might start to make simple sentences. Demonstrate for mom how to check this.
- Child might begin to enjoy dancing with family or with other children. Demonstrate for mom how to check this.

Child Activity

- Child is learning to understand other children and by this learning about themselves. Encourage mom to ask the child about other children.
- Child is learning to understand the emotions of other people in their lives and by this learning about their own emotions. Encourage mom to explain emotions of others to the child.
- Child is still learning how to treat other children. Demonstrate how child can treat other children gently. Encourage mom to continue to teach child good behaviour with other children.

Observe parent and child trying activity. If needed demonstrate again to help caregiver

Ask if the caregiver has any questions or concerns?
Arrange next visit date

Thank mother and family. Encourage mother to try implement the advice and activities.

Note any questions asked, action needed, referral required, or things to discuss with supervisor/team leader

End

Child Milestones

- Ngwana a ka ithuta go bolela dilo tše mpsha letšatši le lengwe le le lengwe
- Ngwana a ka opela dikoša le ba bangwe

- Child might learn to say new things every day
- Child might sing along to songs

- Xihlangi xi nga dyondza ku vula swilo swintshwa masiku hinkwawo
- Xihlangi xi nga yimbelela na un'wana

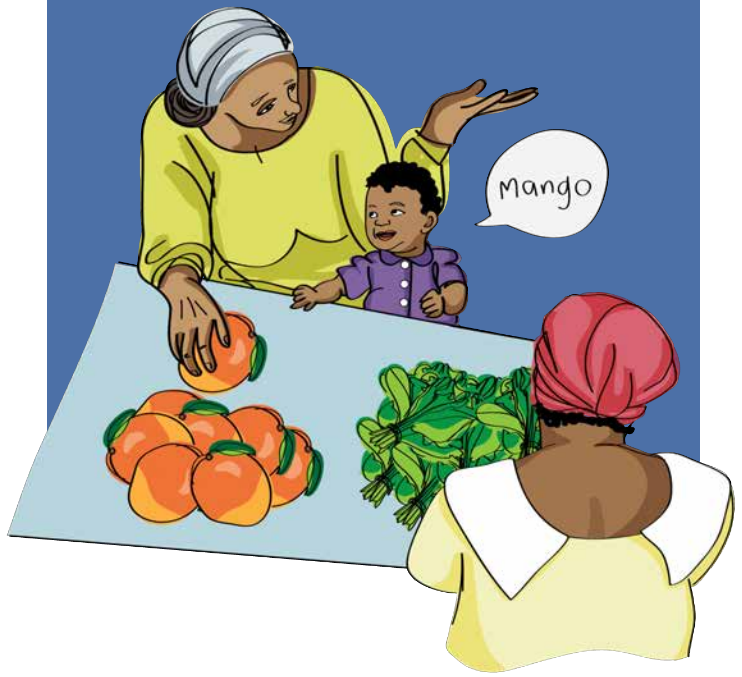

Child Nutrition

- Phepo e ama go gola ga ngwana

- Nutrition affects child's development

- Tinyutiriyente ti khumba makulele ya n'wana

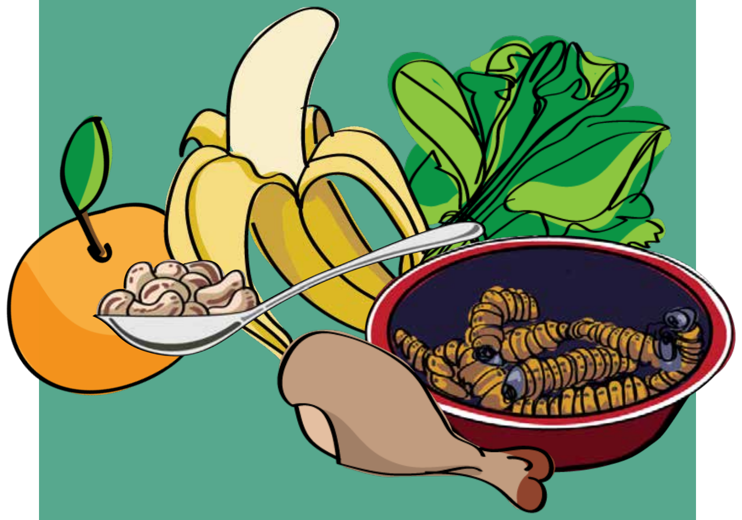

Child Health

- Maphelo a ama kgodišo ya ngwana

- Health affects child's development

- Rihanyu ri khumba makulele ya n'wana

Child Activity

- Opela dikoša le ngwana
- Opela dikosha le ngwana le papa
- Hlalosa maikutlo go ngwana

- Put child on your lap and sing favourite songs
- Sing songs with child and father
- Explain emotions to child

- Veka n'wana mathangeni u yimbelela risimu leri a ri rhandaka
- Yimbelela tinsimu na n'wana na papa.
- Hlamusela matitwelo ka n'wana

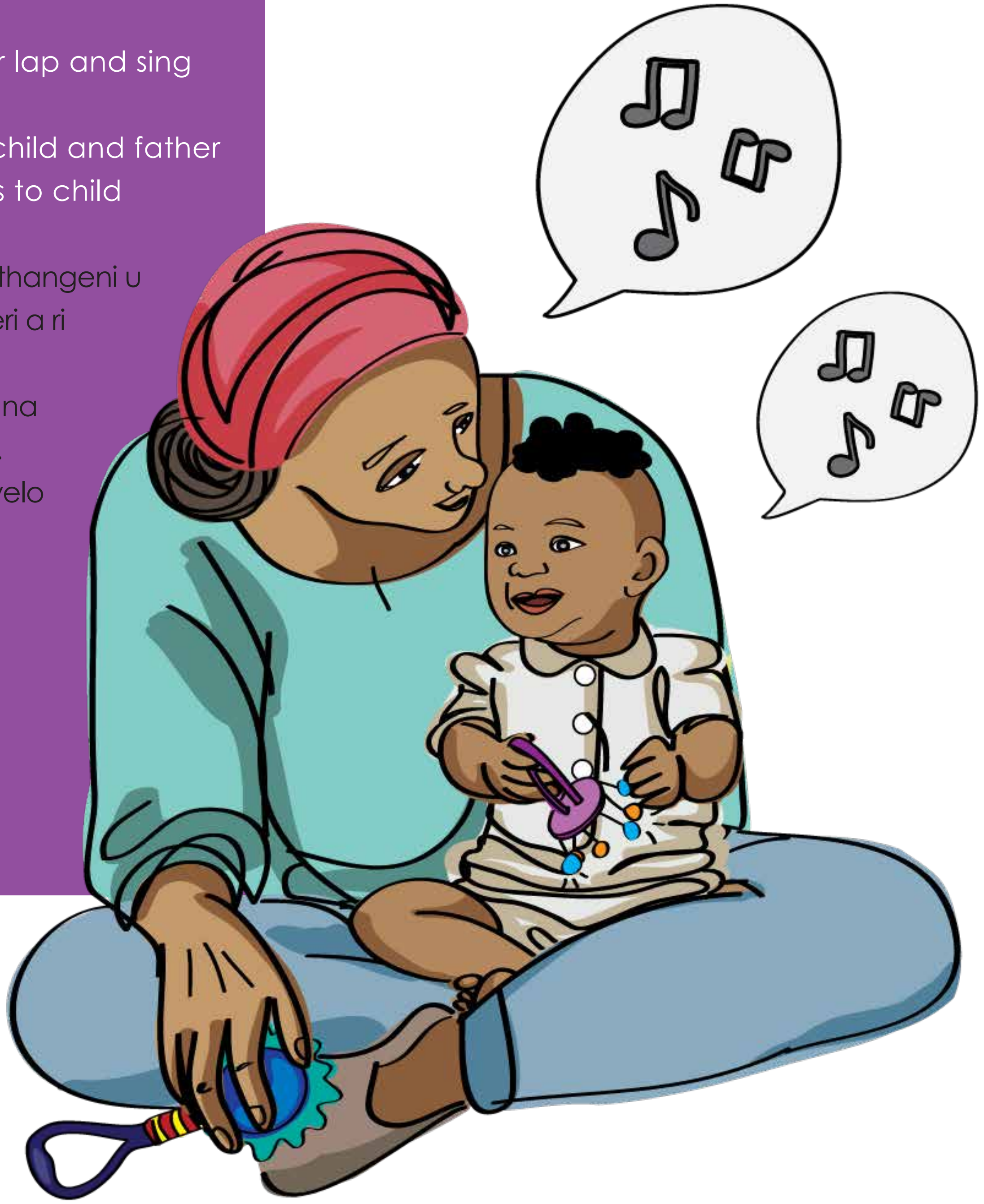

Start

Greet parent and explain purpose of visit.

Ask how parent and child are doing?

Ask caregiver if they have been trying any of the activities since last visit?

Ask if parent can show one of the activities?

Ask if mother/family has any difficulties/challenges in implementing the advice or activities?

Ask if mother/family observed any benefits in implementing the advice or activities?

Ask mother/family if she has any concerns (e.g. child is sick, appears slower to develop)?

Child Health

- Child's health affects physical growth and development. It is important that mom seeks care for child when child has symptoms of illness. Even if symptoms do not seem too serious, untreated infections may slow the child's development over time. Mom should take child to the clinic for a general checkup every three months or so. Ask mom if child has been checked recently, and if not encourage mom to take child to clinic for a checkup.

Child Nutrition

- Child's nutrition affects physical growth and development. It is important that child has enough protein and micronutrients in their diet. If they do not, their growth may be slowed.

Child Milestones

- The child is becoming increasingly talkative now. She might start to learn new words every day. Demonstrate how mom can check for this.
- Child might remember the words to songs and sing along when mom sings. Demonstrate how mom can check this.

Child Activity

- Encourage mom to continue to hold and sing with child even as the child gets older and more active outside.
- Encourage mom and dad to sing and dance together with child and family even as child gets older.
- Child does not fully understand own emotions. Encourage mom to explain emotions to child, e.g., 'you are upset because ...'; 'it is normal to be scared ...'; 'it is okay to be jealous ...'

Observe parent and child trying activity. If needed demonstrate again to help caregiver

Ask if the caregiver has any questions or concerns?
Arrange next visit date

Thank mother and family. Encourage mother to try implement the advice and activities.

Note any questions asked, action needed, referral required, or things to discuss with supervisor/team leader

End

S
E
T

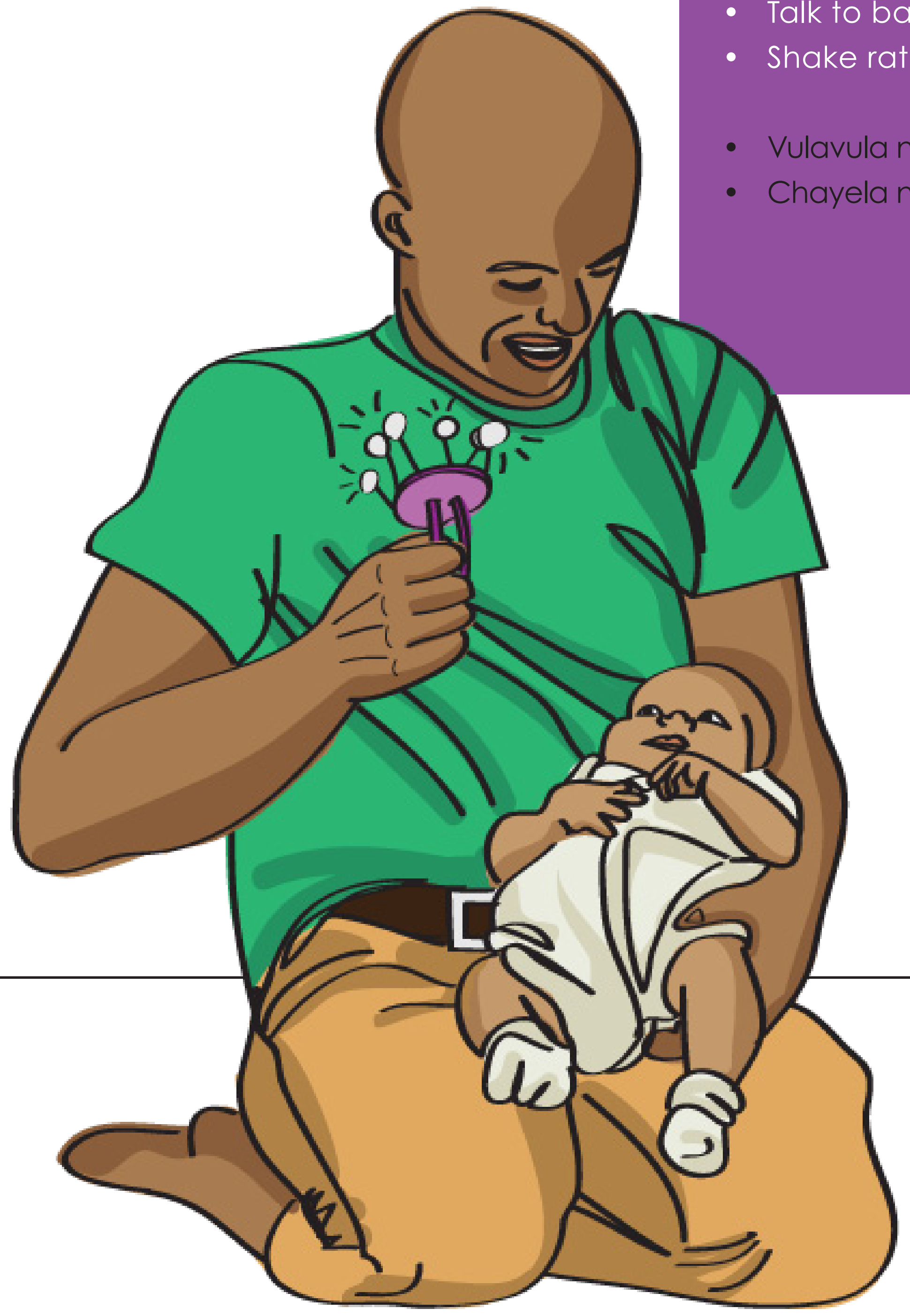

Child Activity

- Bolela le lesea
- Šišinya sebapadišwa sa lesea sa go dira modumongwana

- Talk to baby
- Shake rattle for baby

- Vulavula na n'wana
- Chayela n'wana xikocokoco

Child Milestones

- Lesea le swanetše go hwetša dijo go mma

- Baby should feed from mom

- N'wana u fanele ku hefemula kahle
- N'wana u fanele ku mama eka manana

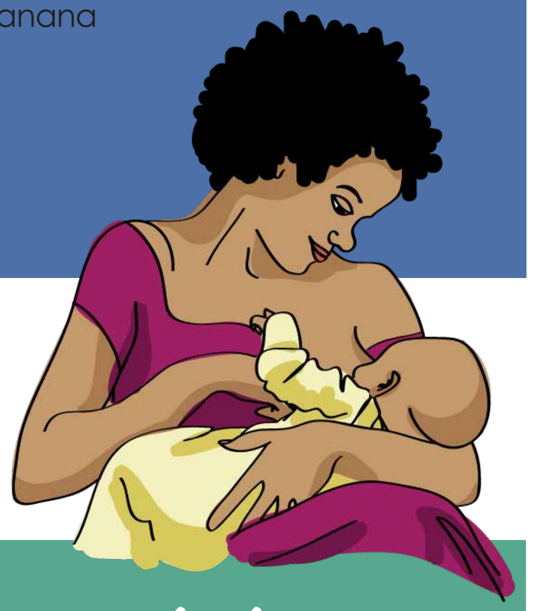

Child Nutrition

- Go amuša go šireletša lesea kgahl-anong le malwetši

- Breastfeeding protects baby against disease

- Ku mamisa vele swi sirhelela n'wana eka mavabyi

Child Health

- Ditšhwaana tša thibela malwetši di šireletša ngwana
- Nyaka thušo ka potlako ge lesea le lwala

- Vaccines protect baby
- Seek care immediately if baby is sick

- Misawutiso yi sirhelela n'wana
- Lava ku pfuniwa hi xihaltla loko n' wana a vabya

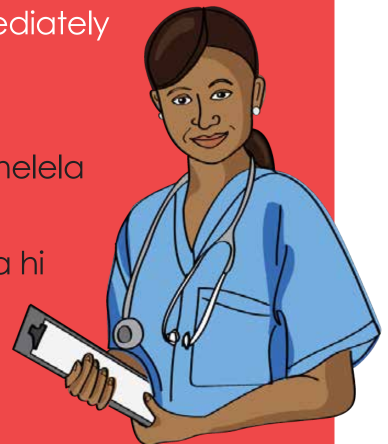

Maternal Wellbeing

- Gantši bomma ba bafsa ba na le go ikwa ba le fasenyana

- New moms often feel 'blue'

- Mikarhi yin'wana vamhani lavantshwa va titwa va 'tsanile'

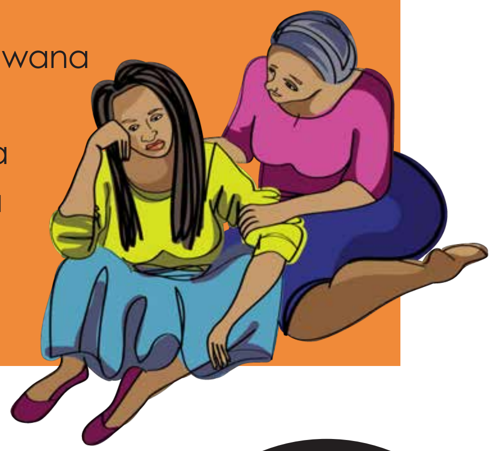

Start

Greet parent and explain purpose of visit.

Ask how parent and child are doing?

Ask mother/family if she has any concerns (e.g. child is sick, appears slower to develop)?

Maternal Wellbeing

- It is normal for new moms to feel 'blue' the first month or two after birth. This is partly due to changes with her body and will likely go away soon. If mom is feeling very 'blue' explain local resources that might help and encourage her to use them.

Child Health

- At birth, babies should receive BCG and OPV0. Confirm that the baby has received these vaccines. If not yet received, encourage mom to bring baby to the clinic for these vaccines immediately.
- Postnatal care in first days after birth is important to make sure baby is gaining weight and umbilical cord isn't infected.

Child Nutrition

- Assist mother with learning breastfeeding and encourage exclusive breastfeeding.

Child Milestones

- It is very normal for baby to sleep most of day, waking only to feed. Mom should observe normal breathing.

Child Activity

- Baby can hear mom talking from birth. Talking to baby from first day will help develop connection with mom, and stimulate development. Encourage mom to talk to baby every day.
- Any colourful object around the house that makes sound can be used as a rattle for baby. Shaking rattle will help baby learn about the world. Help mother find a rattle in house and show how to use with baby.

Observe parent and child trying activity. If needed demonstrate again to help caregiver

Ask if the caregiver has any questions or concerns?
Arrange next visit date

Thank mother and family. Encourage mother to try implement the advice and activities.

Note any questions asked, action needed, referral required, or things to discuss with supervisor/ team leader

End

Maternal Wellbeing

- Gantši bomma ba bafsa ba na le go ikwa ba le fasenyana
- New moms often feel 'blue'
- Mikarhi yin'wana vamhani lavant-shwa va titwa va 'tsanile'

Child Milestones

- Lesea le ka dula le phafogile leb-akanyana le letelele
- Lesea le ka myemyela
- Baby might be awake for longer periods
- Baby might smile
- N'wana u fanele ku hefemula kahle
- N'wana u fanele ku mama eka manna

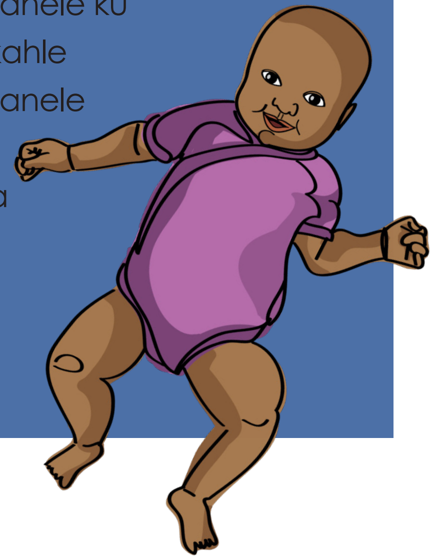

Child Health

- Hlapa diatla pele o swara lesea
- Nyaka thušo ka potlako ge lesea le lwala
- Wash hands before holding baby
- Seek care immediately if baby is sick
- Hlamba swandla u nga si khoma n'wana
- Lava ku pfuniwa hi xihatla loko n'wana a vabya

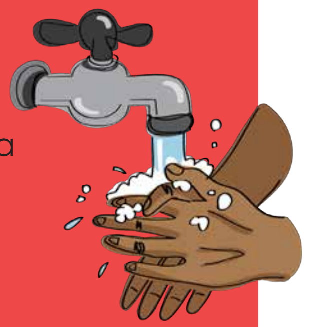

Child Nutrition

- Maswi a mma a na le tšohle tše di hlokwago ke lesea
- Meetse le dijo di ka ba kotsi go lesea
- Mom's milk has all baby needs
- Water and foods can be dangerous for baby
- Mafi ya manana ya na hinkwaswo leswi n'wana a swi lavaka
- Mati na swakudya swi nga va na khombo eka n'wana

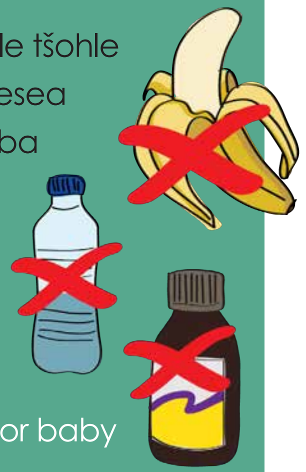

Child Activity

- Bolela le lesea
- Šišinya gape o sepediše sebapadišwa sa modumong-wana pele ga lesea
- Dira gore lesea le bone e bile le kgome dilo
- Talk to baby
- Shake and move rattle in front of baby
- Have baby see and touch objects
- Vulavula na n'wana
- Chaya na ku fambafambisa xiko-cokoco emahlweni ka n'wana
- Endla leswaku n'wana a vona na ku khoma swilo

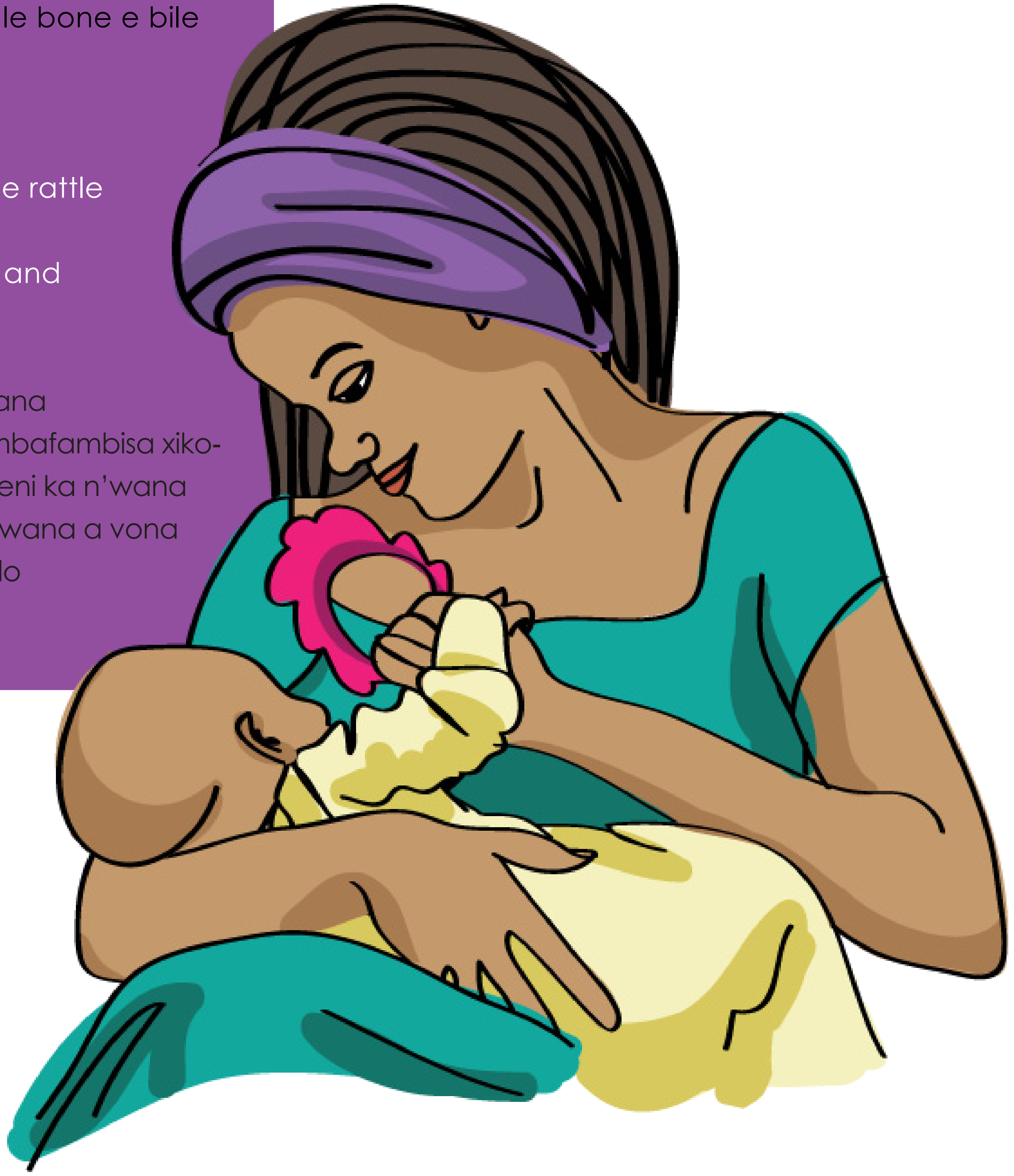

Start

Greet parent and explain purpose of visit.

Ask how parent and child are doing?

Ask caregiver if they have been trying any of the activities since last visit?

Ask if parent can show one of the activities?

Ask if mother/family has any difficulties/challenges in implementing the advice or activities?

Ask if mother/family observed any benefits in implementing the advice or activities?

Ask mother/family if she has any concerns (e.g. child is sick, appears slower to develop)?

Maternal Wellbeing

- It is normal for new moms to feel 'blue' the first month or two after birth. This is partly due to changes with her body and will likely go away soon. If mom is feeling very 'blue' explain local resources that might help and encourage her to use them.

Child Health

- Good hygiene by parents is important for baby. Encourage all caregivers in household to wash hands before touching baby, particularly after using toilet or changing baby.
- If baby has fever, cough, or is not feeding, they should be brought to the clinic immediately. Describe how to check for symptoms. Encourage immediate care seeking if symptoms found.

Child Nutrition

- Continue to encourage exclusive breastfeeding. Describe to mom how it has all of the nutrition that the baby needs at this age.
- Water and other foods can be dangerous for baby because they might have bacteria that cause illness and baby's digestive system is not developed.

Child Milestones

- As baby grows, she will start to be awake for longer periods of time.
- Baby might start smiling at this age.

Child Activity

- Mom should tell baby what she is doing throughout day. Demonstrate how mom can do this.
- Demonstrate how to shake and move rattle side to side in front of baby and see if baby follows with eyes.
- Help mom find colourful objects and different textures around the house for baby to see and touch.

Observe parent and child trying activity. If needed demonstrate again to help caregiver

Ask if the caregiver has any questions or concerns?
Arrange next visit date

Thank mother and family. Encourage mother to try implement the advice and activities.

Note any questions asked, action needed, referral required, or things to discuss with supervisor/team leader

End

S
E
T

Child Activity

- Botša lesea maina le mebala ya dilo
- Dira gore lesea le kgome dilo

- Tell baby object names and colors
- Have baby touch objects

- Byela n'wana mavito na mihlovo ya swilo
- Pfumelela n'wana ku khoma swilo

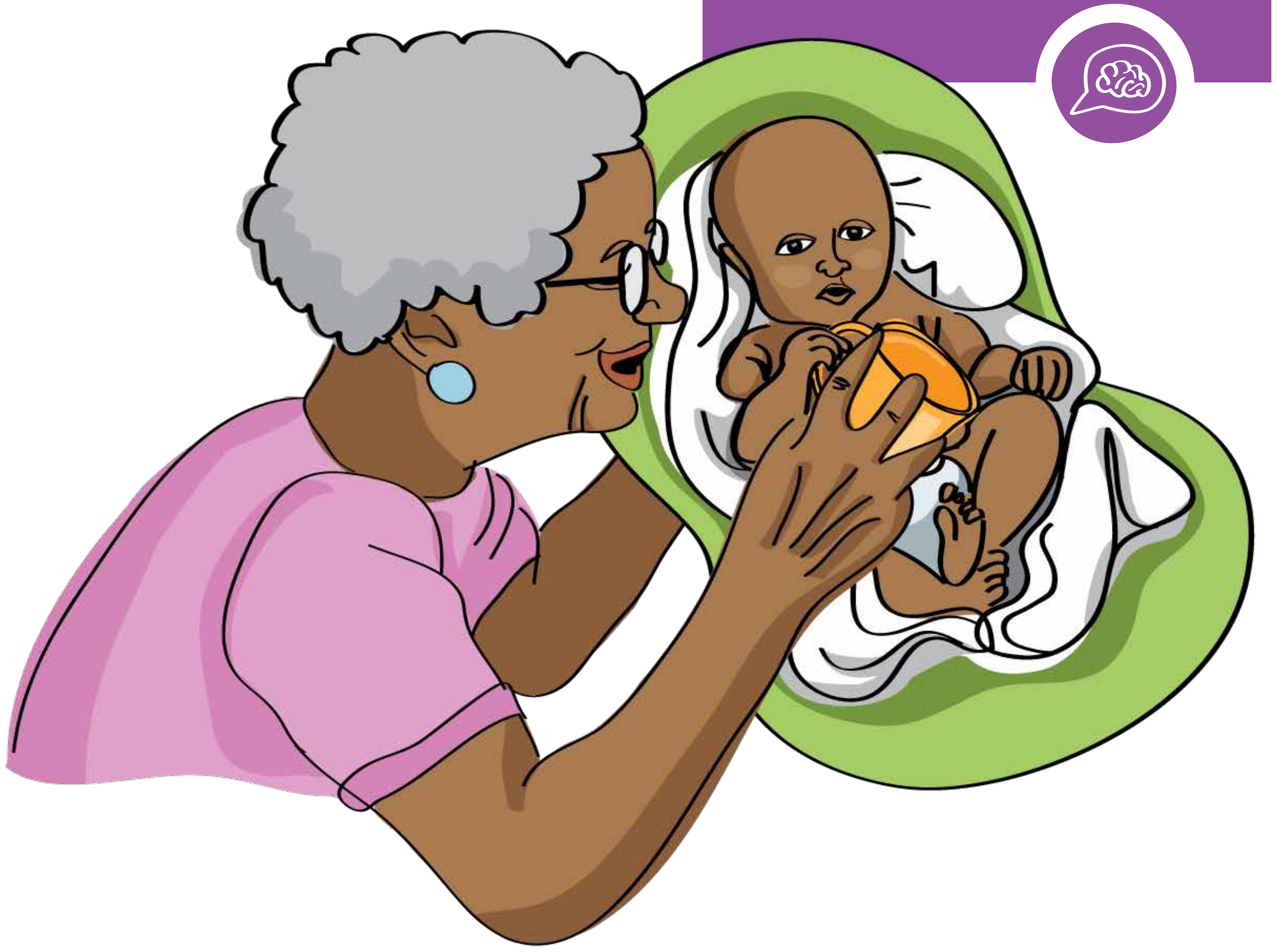

Child Milestones

- Lesea le ka dira medumo ya seb-jana
- Lesea le ka retola hlogo go ya ka modumo

- Baby might coo and make gur-gling sounds
- Baby might turn head toward sounds

- N'wana a nga endla mipfumawulo yo vulavula
- N'wana a nga hundzuluxa nhloko a languta laha mpfumawulo wu twakalaka kona

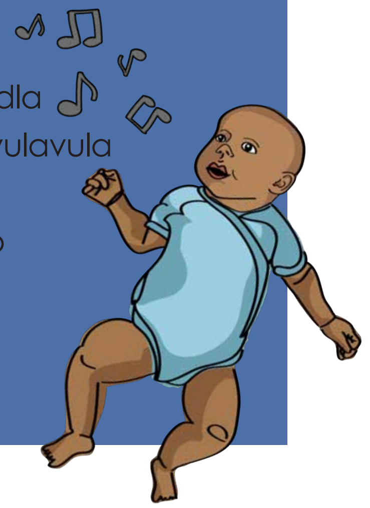

Maternal Wellbeing

- Thibela-pelegi e bohlokwa go tša maphelo a mma

- Family planning is important for mom's health

- Ku kunguhata mbeleko swi na nkoka eka rihanyu ra mhani

Child Health

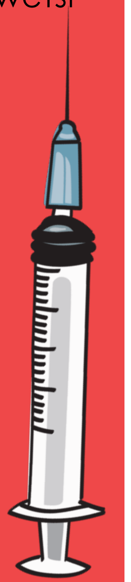

- Ditšhwaana tša thibela malwetši di šireletša ngwana
- Nyaka thušo ka potlako ge lesea le lwala

- Vaccines protect baby
- Seek care immediately if baby is sick

- Misawutiso yi sirhelela n'wana
- Lava ku pfuniwa hi xihaltla loko n'wana a vabya

Child Nutrition

- Maswi a mma a na le tšohle tše di hlokwago ke lesea
- Meetse le dijo di ka ba kotsi go lesea

- Mom's milk has all baby needs
- Water and foods can be dangerous for baby

- Mafi ya manana ya na hinkwaswo leswi n'wana a swi lavaka
- Mati na swakudya swi nga va na khombo eka n'wana

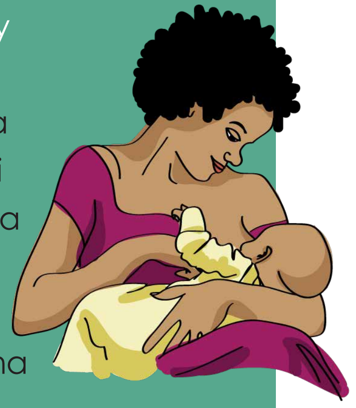

Start

Greet parent and explain purpose of visit.

Ask how parent and child are doing?

Ask caregiver if they have been trying any of the activities since last visit?

Ask if parent can show one of the activities?

Ask if mother/family has any difficulties/challenges in implementing the advice or activities?

Ask if mother/family observed any benefits in implementing the advice or activities?

Ask mother/family if she has any concerns (e.g. child is sick, appears slower to develop)?

Maternal Wellbeing

- Explain to mom the family planning options available and encourage her to visit the clinic soon for a consultation.

Child Health

- At 6 weeks old, babies should receive OPV1, RV1, DTaP-IPV/Hib1, HBV1, and PCV1. Confirm that the baby has received these vaccines. If not yet received, encourage mom to bring baby to the clinic for these vaccines immediately.

- If baby has fever, cough, or is not feeding, they should be brought to the clinic immediately. Describe how to check for symptoms. Encourage immediate care seeking if symptoms found.

Child Nutrition

- Continue to encourage exclusive breastfeeding. Describe to mom how it has all of the nutrition that the baby needs at this age.
- Water and other foods can be dangerous for baby because they might have bacteria that cause illness and baby's digestive system is not developed.

Child Milestones

- Baby might coo and make gurgling sounds. Demonstrate sounds mom can listen for.

- Baby might turn head toward sounds. Demonstrate how mom can look for this.

Child Activity

- Help mom find colourful objects around the house for baby to touch. Encourage mom to talk to the baby by telling her about the object names and colors.

- Demonstrate how mom can have baby touch objects.

Observe parent and child trying activity. If needed demonstrate again to help caregiver

Ask if the caregiver has any questions or concerns?
Arrange next visit date

Thank mother and family. Encourage mother to try implement the advice and activities.

Note any questions asked, action needed, referral required, or things to discuss with supervisor/team leader

End

Maternal Wellbeing

- Go amuša ga go thibele go ima
- Breastfeeding does not protect against pregnancy
- Ku mamisa vele a swi siveli vuyimani

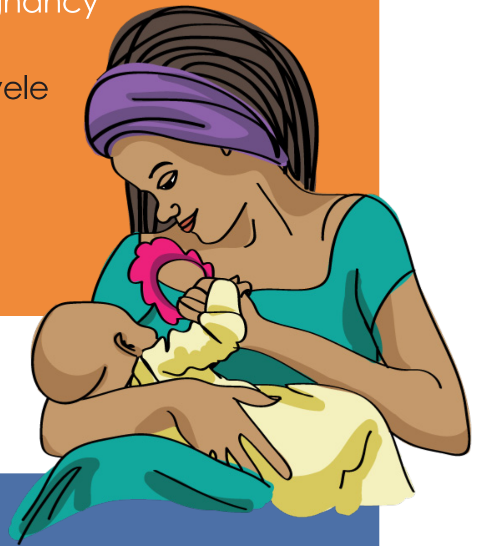

Child Health

- Ditšhwaana tša thibela malwetši di šireletša ngwana
- Nyaka thušo ka potlako ge lesea le lwala
- Vaccines protect baby
- Seek care immediately if baby is sick
- Misawutiso yi sirhelela n'wana
- Lava ku pfuniwa hi xihatla loko n'wana a vabya

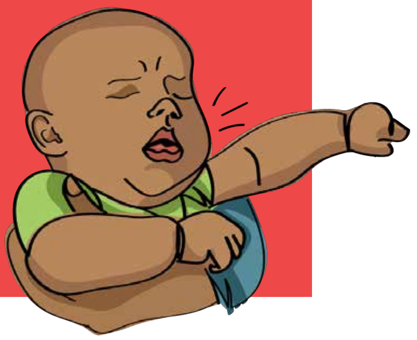

Child Activity

- Botša lesea maina le tirišo ya dilo
- Dira lesea le sware dilo
- Tell baby object names and uses
- Have baby hold objects
- Byela n'wana mavito na mitirho ya swilo
- Khomisa n'wana swilo

Child Milestones

- Lesea le ka latella dilo ka mahlo
- Lesea le ka emiša hlogo
- Baby might follow objects with eyes
- Baby might lift head
- N'wana a nga landzelela swilo hi mahlo
- N'wana a nga tlakusa nhloko

Child Nutrition

- Maswi a mma a na le tšohle tše di hlokwago ke lesea
- Meetse le dijo di ka ba kotsi go lesea
- Mom's milk has all baby needs
- Water and foods can be dangerous for baby
- Mafi ya manana ya na hinkwaswo leswi n'wana a swi lavaka
- Mati na swakudya swi nga va na khombo eka n'wana

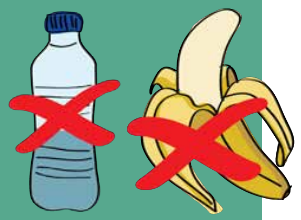

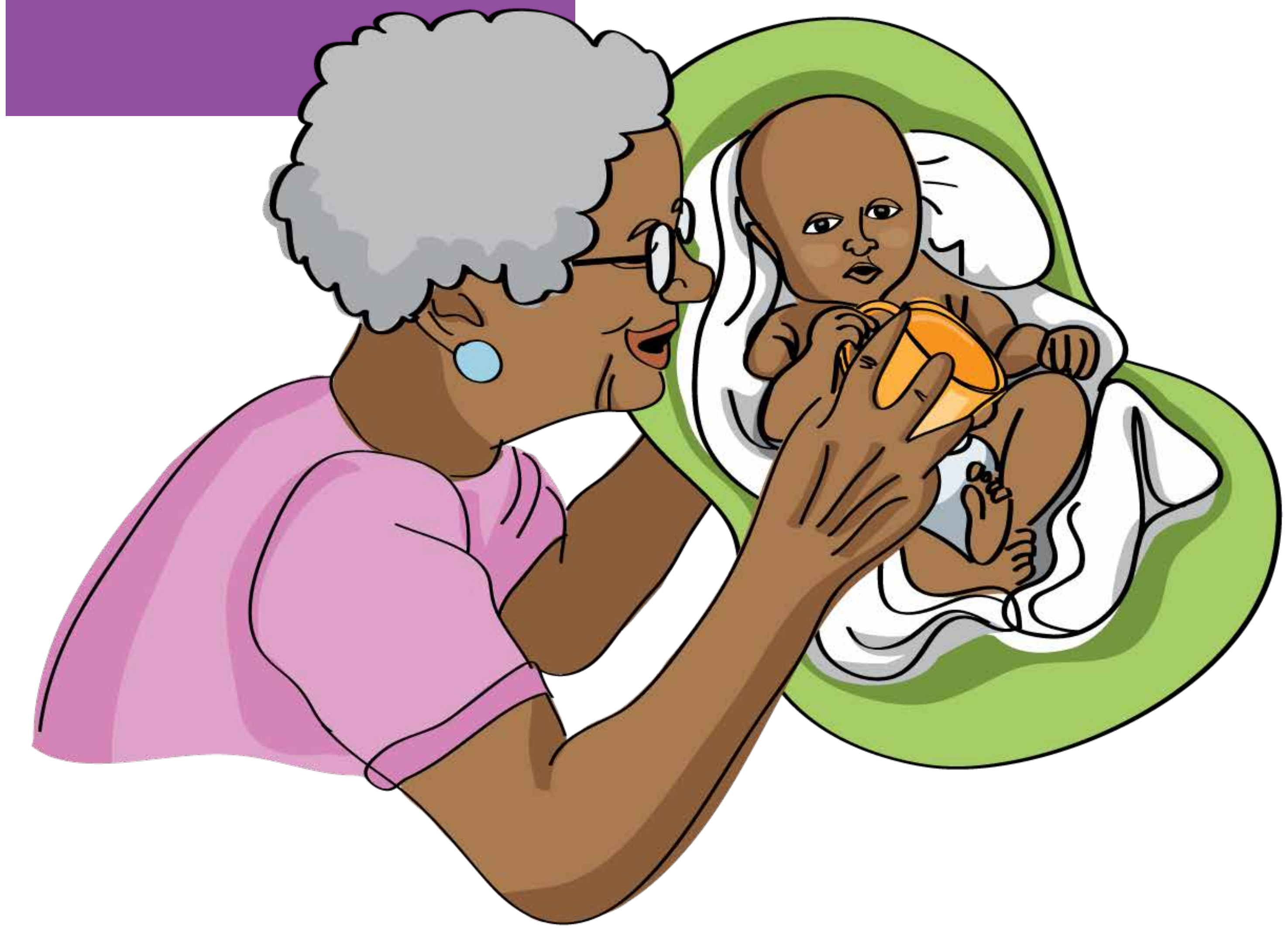

Start

Greet parent and explain purpose of visit.

Ask how parent and child are doing?

Ask caregiver if they have been trying any of the activities since last visit?

Ask if parent can show one of the activities?

Ask if mother/family has any difficulties/challenges in implementing the advice or activities?

Ask if mother/family observed any benefits in implementing the advice or activities?

Ask mother/family if she has any concerns (e.g. child is sick, appears slower to develop)?

Maternal Wellbeing

- Explain to mom the family planning options available and encourage her to visit the clinic soon for a consultation.

Child Health

- At 10 weeks old, babies should receive DTaP-IPV/Hib2, and HBV2. Confirm that the baby has received these vaccines. If not yet received, encourage mom to bring baby to the clinic for these vaccines immediately.
- If baby has fever, cough, or is not feeding, they should be brought to the clinic immediately. Describe how to check for symptoms. Encourage immediate care seeking if symptoms found.

Child Nutrition

- Continue to encourage exclusive breastfeeding. Describe to mom how it has all of the nutrition that the baby needs at this age.
- Water and other foods can be dangerous for baby because they might have bacteria that cause illness and baby's digestive system is not developed.

Child Milestones

- Baby might follow objects with eyes. Demonstrate how mom can look for this.
- Baby might lift head when being held against mom's shoulder. Demonstrate how mom can look for this.

Child Activity

- Help mom find objects around the house for baby to hold. Encourage mom to talk to the baby by telling her about the object names and uses.
- Demonstrate how mom can have baby hold objects.

Observe parent and child trying activity. If needed demonstrate again to help caregiver

Ask if the caregiver has any questions or concerns?
Arrange next visit date

Thank mother and family. Encourage mother to try implement the advice and activities.

Note any questions asked, action needed, referral required, or things to discuss with supervisor/team leader

End

S
E
T

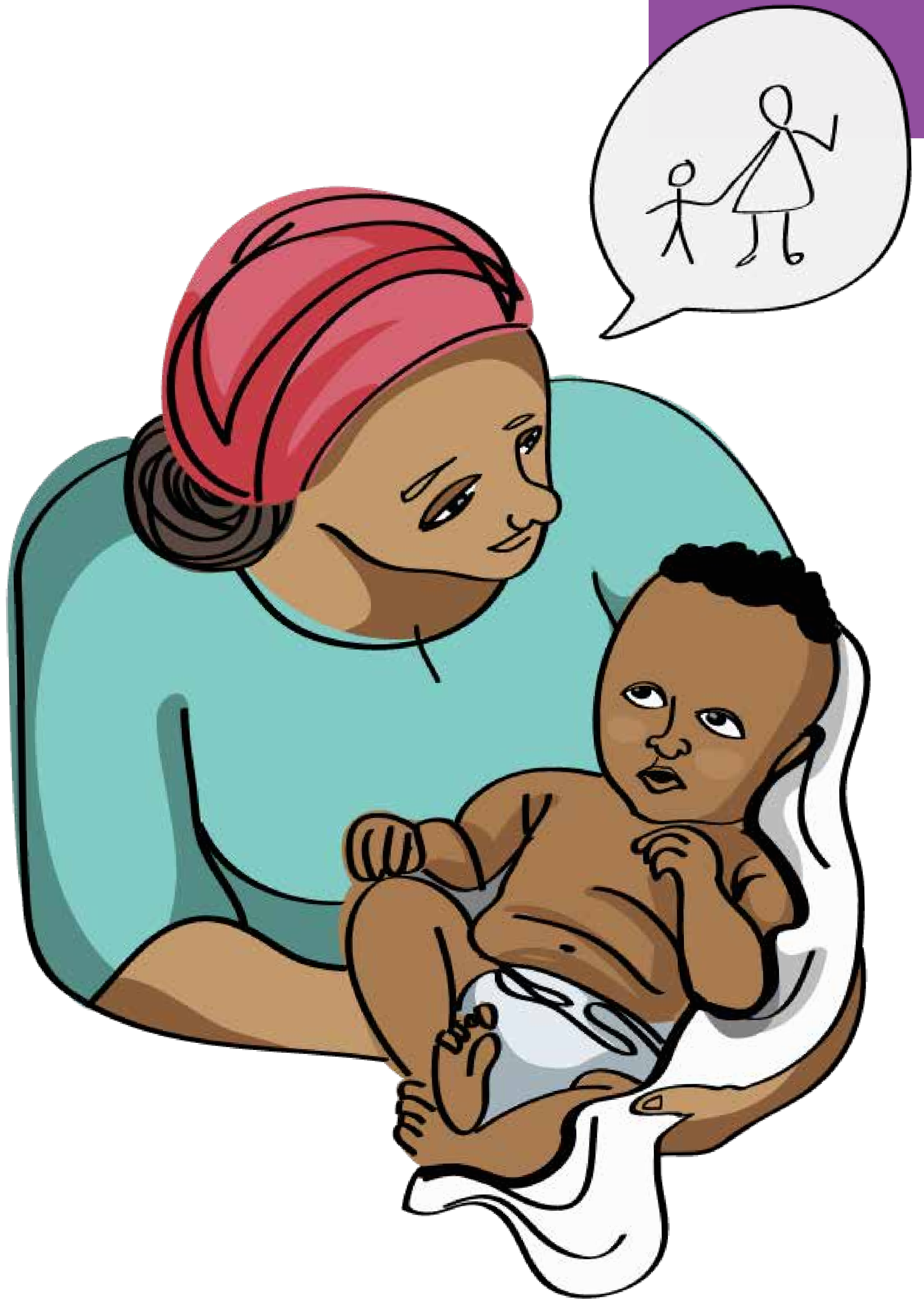

Child Activity

- Anegela lesea nonwane
- Dira gore lesea le fihlelle dilo

- Tell baby a story
- Have baby reach for objects

- Hlayela n'wana xitori
- Pfumelela n'wana ku fikelela swilo

Child Milestones

- Lesea le ka swara dilo ka diatla
- Lesea le ka iphetola

- Baby might hold objects in hands
- Baby might roll over

- N'wana a nga khoma swilo hi mavoko
- N'wana a nga vumbuluka

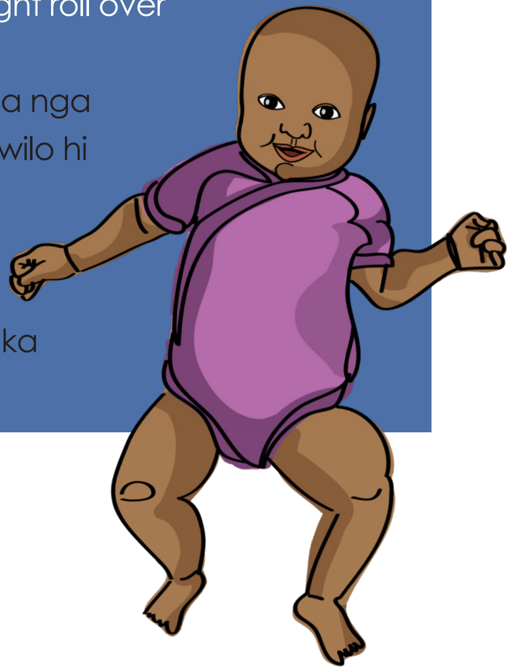

Maternal Wellbeing

- Thibela-pelegi e bohlokwa go tša maphelo a mma

- Family planning is important for mom's health

- Ku kunguhata mbeleko swi na nkoka eka rihanyu ra mhani

Child Health

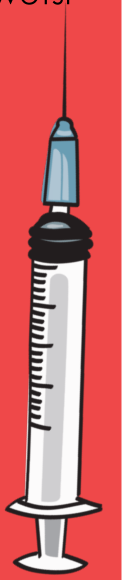

- Ditšhwaana tša thibela malwetši di šireletša ngwana
- Nyaka thušo ka potlako ge lesea le lwala

- Vaccines protect baby
- Seek care immediately if baby is sick

- Misawutiso yi sirhelela n'wana
- Lava ku pfuniwa hi xihaltla loko n'wana a vabya

Child Nutrition

- Maswi a mma a na le tšohle tše di hlokwago ke lesea
- Meetse le dijo di ka ba kotsi go lesea

- Mom's milk has all baby needs
- Water and foods can be dangerous for baby

- Mafi ya manana ya na hinkwaswo leswi n'wana a swi lavaka
- Mati na swakudya swi nga va na khombo eka n'wana

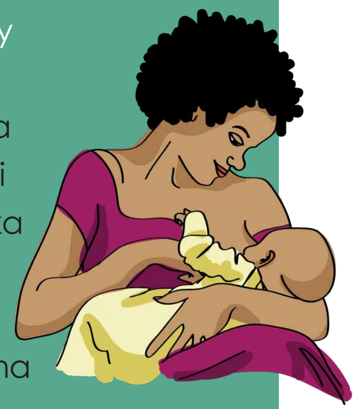

Start

Greet parent and explain purpose of visit.

Ask how parent and child are doing?

Ask caregiver if they have been trying any of the activities since last visit?

Ask if parent can show one of the activities?

Ask if mother/family has any difficulties/challenges in implementing the advice or activities?

Ask if mother/family observed any benefits in implementing the advice or activities?

Ask mother/family if she has any concerns (e.g. child is sick, appears slower to develop)?

Maternal Wellbeing

- Explain to mom the family planning options available and encourage her to visit the clinic soon for a consultation.

Child Health

- At 14 weeks old, babies should receive RV2, DTaP-IPV/Hib3, HBV3, and PCV2. Confirm that the baby has received these vaccines. If not yet received, encourage mom to bring baby to the clinic for these vaccines immediately.
- If baby has fever, cough, or is not feeding, they should be brought to the clinic immediately. Describe how to check for symptoms. Encourage immediate care seeking if symptoms found.

Child Nutrition

- Continue to encourage exclusive breastfeeding. Describe to mom how it has all of the nutrition that the baby needs at this age.
- Water and other foods can be dangerous for baby because they might have bacteria that cause illness and baby's digestive system is not developed.

Child Milestones

- Baby might hold objects in hands. Demonstrate how mom can look for this.
- Baby might roll over. Demonstrate how mom can look for this.

Child Activity

- Talk to mom about an example of a story she can tell to baby. Encourage mom to tell story to baby every day, maybe just before she goes to sleep at night.
- Demonstrate how mom can have baby reach for objects.

Observe parent and child trying activity. If needed demonstrate again to help caregiver

Ask if the caregiver has any questions or concerns?
Arrange next visit date

Thank mother and family. Encourage mother to try implement the advice and activities.

Note any questions asked, action needed, referral required, or things to discuss with supervisor/team leader

End

S
E
T

Maternal Wellbeing

- Go hlola ga maphelo a mma
- Check-up on mom's health
- Nkambelo wa rihanyu ra manana

Child Health

- Nyaka thušo ka potlako ge lesea le lwala
- Tšwelapele ka go fepa gareng ga bolwetši
- Hlola kgolo ya lesea

- Seek care immediately if baby is sick
- Continue feeding during sickness
- Check baby's growth

- Lava ku pfuniwa hi xihatla loko n'wana a vabya
- Yana emahlweni na ku mamisa n'wana hambiloko a vabya
- Kamba ku kula ka n'wana

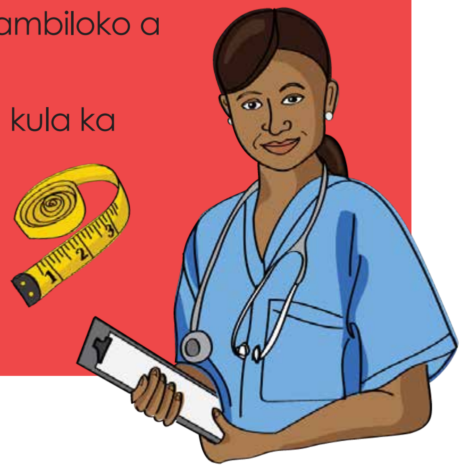

Child Milestones

- Lesea le ka fihlella dilo
- Lesea le ka lemoga ba leloko ba sa le kgojana

- Baby might reach for objects
- Baby might recognize family at a distance

- N'wana a nga fikelela swilo
- N'wana a nga tiva swirho swa ndyangu swi ri empfhukeni

Child Nutrition

- Ka go nanya thoma go fa lesea dijo tše nnyane tše bonolo
- Go tšwelela ka go amuša go loketše lesea

- Slowly introduce a few simple foods
- Continuing breastfeeding good for baby

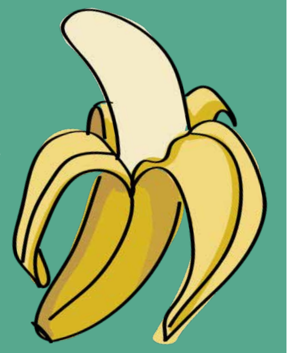

- Dyondzisa n'wana swakudya swintshwa hi switsongotsongo
- Yana emahlweni na ku mamisa hikuva swi kahle eka n'wana

Child Activity

- Bapala 'peek-a-boo' le lesea
- Dira gore lesea le sepediše dilo go tšwa seatleng se sengwe go ya go se sengwe

- Play peek-a-boo with baby
- Have baby pass objects be-tween hands

- Ntlanga peek-a-boo (ntlangu wo titumbeta xikandza hi swandla) na n'wana
- Pfumelela n'wana ku hundzisa swilo ku suka eka voko rin'wana ku ya eka lerin'wana

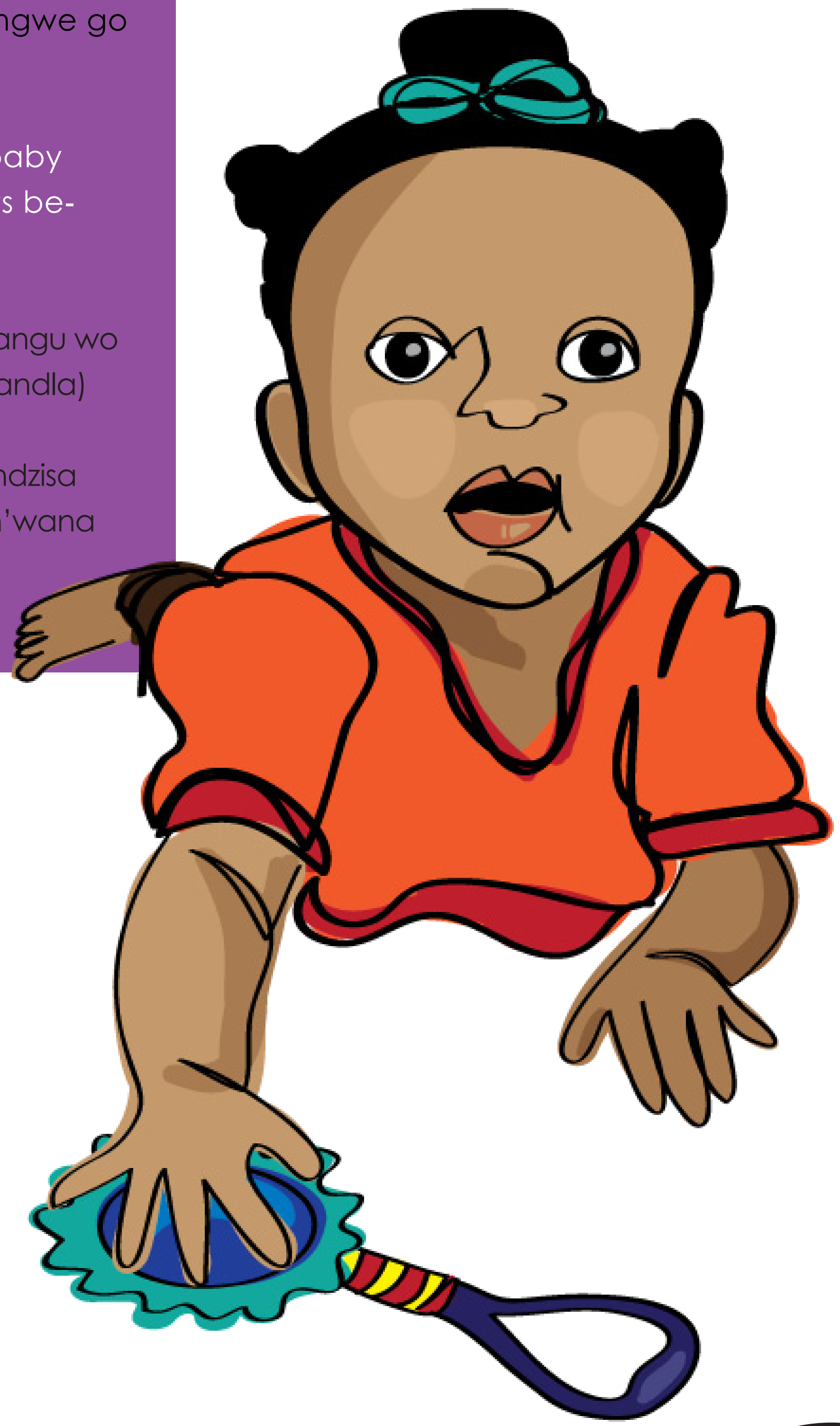

Start

Greet parent and explain purpose of visit.

Ask how parent and child are doing?

Ask caregiver if they have been trying any of the activities since last visit?

Ask if parent can show one of the activities?

Ask if mother/family has any difficulties/challenges in implementing the advice or activities?

Ask if mother/family observed any benefits in implementing the advice or activities?

Ask mother/family if she has any concerns (e.g. child is sick, appears slower to develop)?

Maternal Wellbeing

- Mom should get a general check-up at the clinic at least once in the six months after delivery. Check whether mom has been to the clinic for a check-up since delivery. If not, encourage her to visit clinic soon.

Child Health

- If baby has fever, cough, or is not feeding, they should be brought to the clinic immediately. Describe how to check for symptoms. Encourage immediate care seeking if symptoms found.
- Sometimes, sick babies might seem like they do not want to eat. However, it is very important for baby's health that she continue to be fed during sickness.
- Check whether baby has received weight or height measurement since birth. If not, encourage mom to take baby to clinic soon to be checked.

Child Nutrition

- At this age, mom can start to introduce simple new foods to baby. Encourage mom to give baby one new food each week, for one meal each day. Give examples of new foods.
- While new foods are introduced one meal each day, mom should continue to breastfeed for all other meals. Encourage mom to keep breastfeeding as long as possible, though her milk production may start to decline.

Child Milestones

- Baby might reach for objects. Demonstrate how mom can look for this.
- Baby might recognize family at a distance. Demonstrate how mom can check for this.

Child Activity

- Demonstrate how mom can play peek-a-boo with baby. Put face near baby's face, cover face with hands, and then move hands and smile and say 'peek-a-boo' to baby.
- Demonstrate how mom can have baby pass objects between hands.

Observe parent and child trying activity. If needed demonstrate again to help caregiver

Ask if the caregiver has any questions or concerns?
Arrange next visit date

Thank mother and family. Encourage mother to try implement the advice and activities.

Note any questions asked, action needed, referral required, or things to discuss with supervisor/team leader

End

S
E
T

Child Activity

- Botša lesea ka ga dilo tše le di lebeletšego
- Raloka papadi ya 'naa [selo] se kae?'

- Talk to baby about objects she looks at
- Play game 'where is the [object]?'

- Vulavula na xihlangi hi swilo leswi xi swi langutaka
- 2. Tlanga ntlangu wa '[xilo] xi le kwini?'

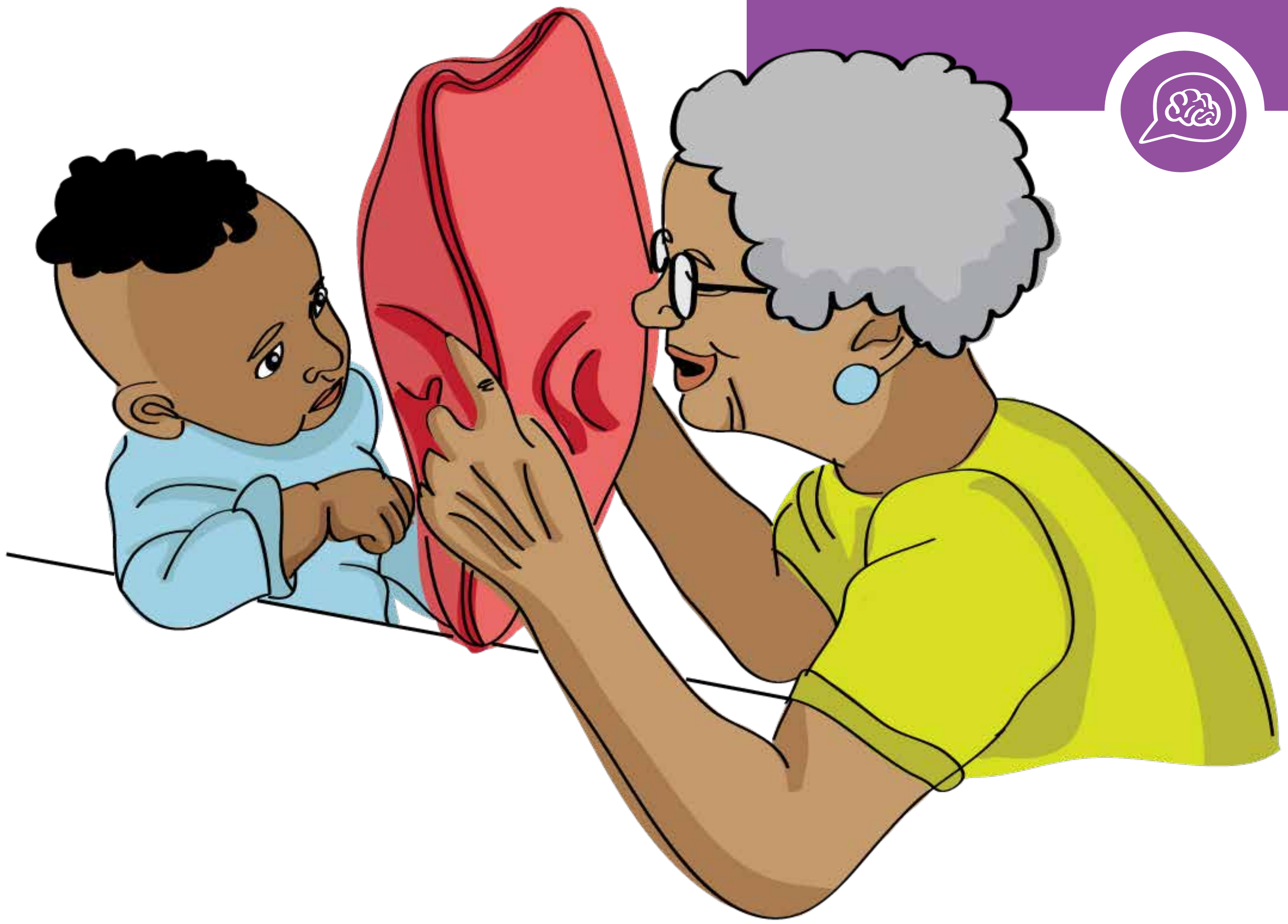

Child Milestones

- Lesea le ka dula ntle le go thekgwa
- Lesea le ka moma dilo ka molomong

- Baby might sit without support
- Baby might put things in mouth

- Xihlangi xi nga tshama handle ka ku seketeriwa
- Xihlangi xi nga hoxa swilo enon'weni

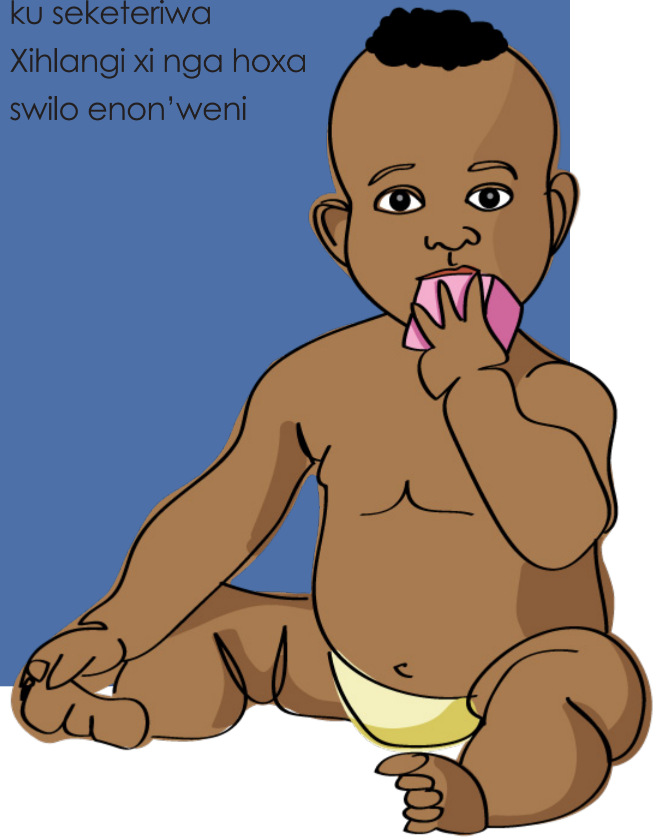

Child Health

- E ba le tsebo ka ga letšhollo
- Šomiša motswako wa go thibela letšhollo (ORS)
- Lesea le hloka maphelo a mabotse

- Look for diarrhea
- Use oral rehydration salt (ORS) solution
- Baby needs good hygiene

- Languta nchuluko
- Tirhisa diripi (oral rehydration salt [ORS])
- Swihlangi swi lava nsivelamavabyi wa kahle

Child Nutrition

- Go nyantšha letswele + go fa dijo makga a 2
- Mahwana a teye a 2-3 a protheine (dinawa goba mae)

- Breastfeeding + 2 meals
- 2-3 teaspoons protein (beans or eggs)

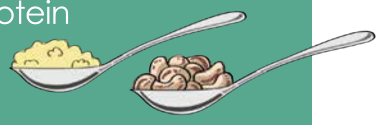

- Ku mamisa vele + miphamelo yi mbirhi.
- 2-3 wa swilepulana swa phurotheyini (tinyawa kumbe matandza)

Start

Greet parent and explain purpose of visit.

Ask how parent and child are doing?

Ask caregiver if they have been trying any of the activities since last visit?

Ask if parent can show one of the activities?

Ask if mother/family has any difficulties/challenges in implementing the advice or activities?

Ask if mother/family observed any benefits in implementing the advice or activities?

Ask mother/family if she has any concerns (e.g. child is sick, appears slower to develop)?

Child Health

- As baby starts to eat more and more new foods, the risks for diarrhea may increase. Encourage mom to pay close attention to whether baby has diarrhea, particularly if baby is showing signs of dehydration or malnutrition due to diarrhea.
- Encourage mom to give baby ORS when baby has diarrhea to avoid dehydration. Demonstrate for mom how to make ORS or discuss where she can buy ORS packets nearby.
- Baby is getting older and starting to move around and touch more things. This means baby is being exposed to more bacteria around the house. Encourage mom to clean baby regularly, every day if possible.

Child Nutrition

- At this age, baby's daily diet should include 2 meals of food and breastfeeding for all other meals. Discuss with mom a plan for the 2 food meals and continued breastfeeding.
- Baby needs protein to grow and develop properly. Breastmilk has lots of good protein. For the food meals, mom should give baby 2 – 3 teaspoons of protein. Discuss local options for protein for baby, for example beans or eggs.

Child Milestones

- Baby might sit without support. Demonstrate how mom can look for this.
- Baby might put things in mouth. Demonstrate how mom can look for this.

Child Activity

- At this age baby will be curious and look at many things around the house. Encourage mom to talk to baby about the objects that baby looks at.
- Demonstrate how mom can play game 'where is the [object]' with baby. For this game, mom should pick out an object that baby is familiar with and can see, and ask baby 'where is the [object]?'. For example, mom can put rattle near baby and ask 'where is the rattle?'

Observe parent and child trying activity. If needed demonstrate again to help caregiver

Ask if the caregiver has any questions or concerns?
Arrange next visit date

Thank mother and family. Encourage mother to try implement the advice and activities.

Note any questions asked, action needed, referral required, or things to discuss with supervisor/team leader

End

S
E
T

Child Health

- E ba le tsebo ka ga letadi
- Hwetša kalafo ka potlako ge lesea le na le letadi
- Lesea le swanetše go khupetšwa ka nete ge le robetše

- Look for fever
- Seek care immediately if baby has fever
- Baby should sleep under net

- Kamba ku hisa ka miri
- Lava vutshunguri hi xihatla loko xihlangi xi hisa miri
- Xihlangi xi fanele ku etlerisiwa endzeni ka nete

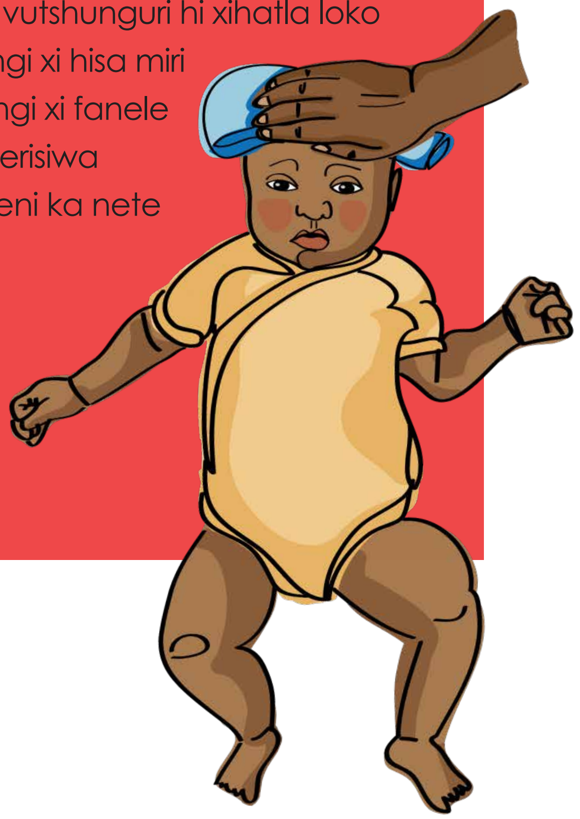

Child Milestones

- Lesea le ka araba ge leina le bitšwa
- Lesea le ka thoma go abula

- Baby might respond to name
- Baby might start to crawl

- Xihlangi xi nga tiva vito ra xona loko ri vitaniwa
- Xihlangi xi nga sungula ku kasa

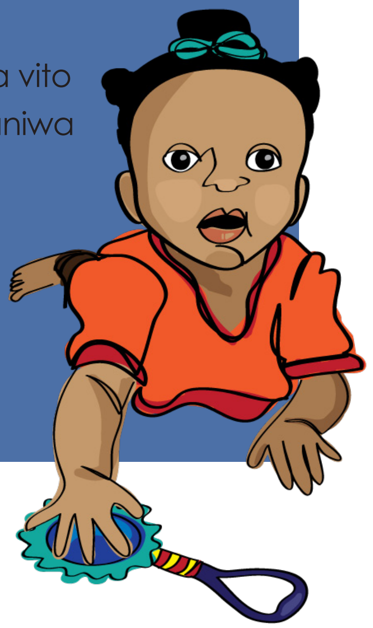

Child Nutrition

- Go nyantšha letswele + go fa dijo makga a 2
- Masea a rata dijo tše ntši

- Breastfeeding + 2 meals
- Baby likes many foods

- Ku mamisa vele + miphamelo yi mbirhi
- Xihlangi xi tsakela swakudya swo hambanahambana

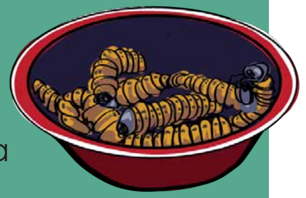

Child Activity

- Botša lesea ka ga dilo ge le le gare le šuthašutha
- E re lesea le bee dilo tša ka 'seatleng' ka khontheineng

- Talk to baby about objects as she moves
- Have baby put 'palm' objects in container

- Vulavula na xihlangi hi swilo leswi xi swi vonaka loko xi karhi xi rhendzelekarhendzeleka
- Byela xihlangi ku chela swilo leswi xi nga swi khomaka eka khontheyinara

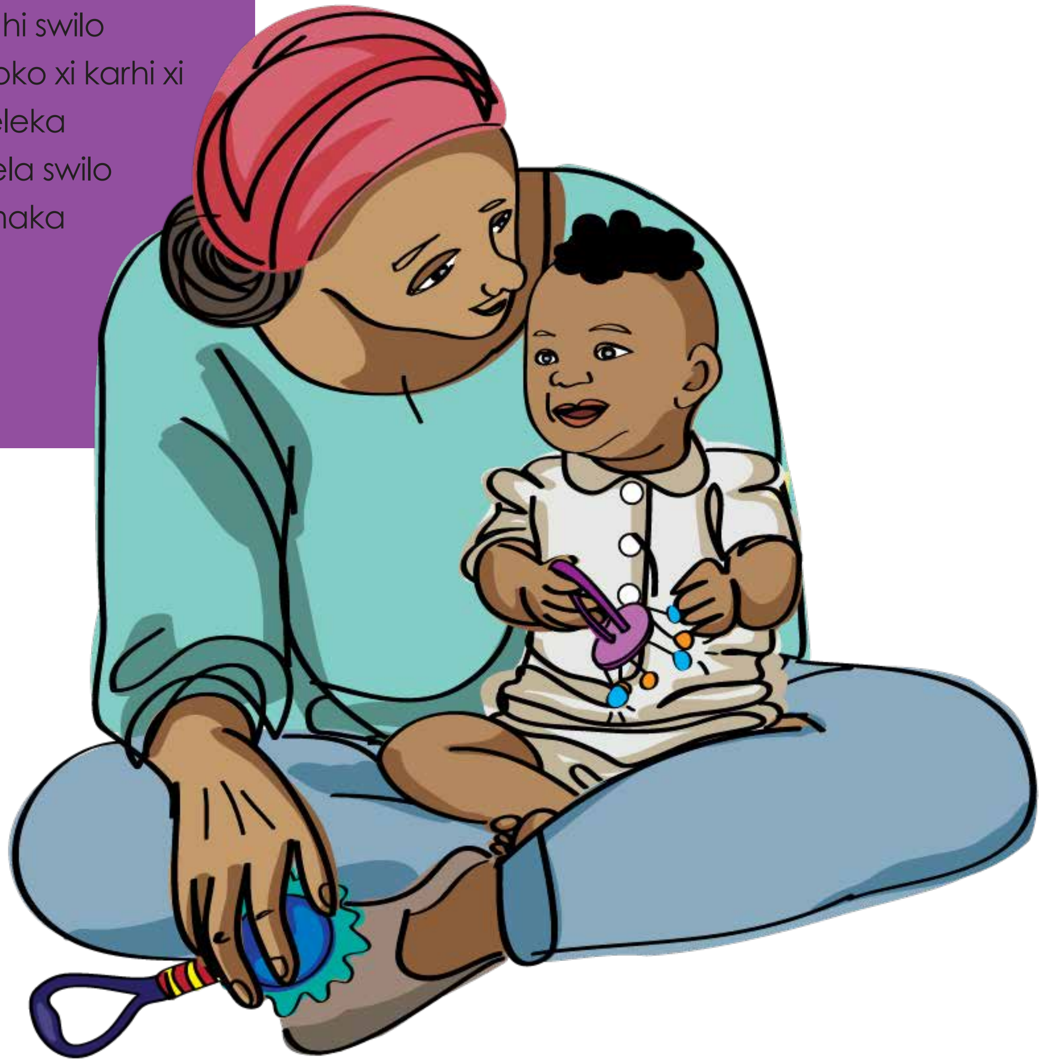

Start

Greet parent and explain purpose of visit.

Ask how parent and child are doing?

Ask caregiver if they have been trying any of the activities since last visit?

Ask if parent can show one of the activities?

Ask if mother/family has any difficulties/challenges in implementing the advice or activities?

Ask if mother/family observed any benefits in implementing the advice or activities?

Ask mother/family if she has any concerns (e.g. child is sick, appears slower to develop)?

Child Health

- Check baby for signs of fever and demonstrate for mom how she can also check. Encourage mom to pay close attention to baby for fever symptoms.
- Encourage mom to seek care immediately if baby has signs of fever. Explain that fever might be a sign of malaria or other infection and that the clinic has medicines that can help.
- Encourage mom to place baby under an insecticide treated bednet at night to protect baby against mosquitos that might cause malaria.

Child Nutrition

- At this age, baby's daily diet should include 2 meals of food and breastfeeding for all other meals. Discuss with mom a plan for the 2 food meals and continued breastfeeding.
- Baby needs a diverse diet with protein and other micronutrients to grow and develop properly. Breastmilk has lots of good protein and nutrients. For the food meals, mom should give baby a variety of other foods. Discuss adding at least one new food to increase diversity and add protein or micronutrients.

Child Milestones

- Baby might respond to name. Demonstrate how mom can look for this.
- Baby might start to crawl. Demonstrate how mom can look for this

Child Activity

- As baby starts to crawl, she will move around and explore objects around the house. Encourage mom to talk to baby about the objects that baby explores as she crawls.
- Baby should now be able to hold and move large objects on the palm of her hand. Demonstrate how mom can give baby larger 'palm' objects and have baby place the objects in a container.

Observe parent and child trying activity. If needed demonstrate again to help caregiver

Ask if the caregiver has any questions or concerns?
Arrange next visit date

Thank mother and family. Encourage mother to try implement the advice and activities.

Note any questions asked, action needed, referral required, or things to discuss with supervisor/team leader

End

S
E
T

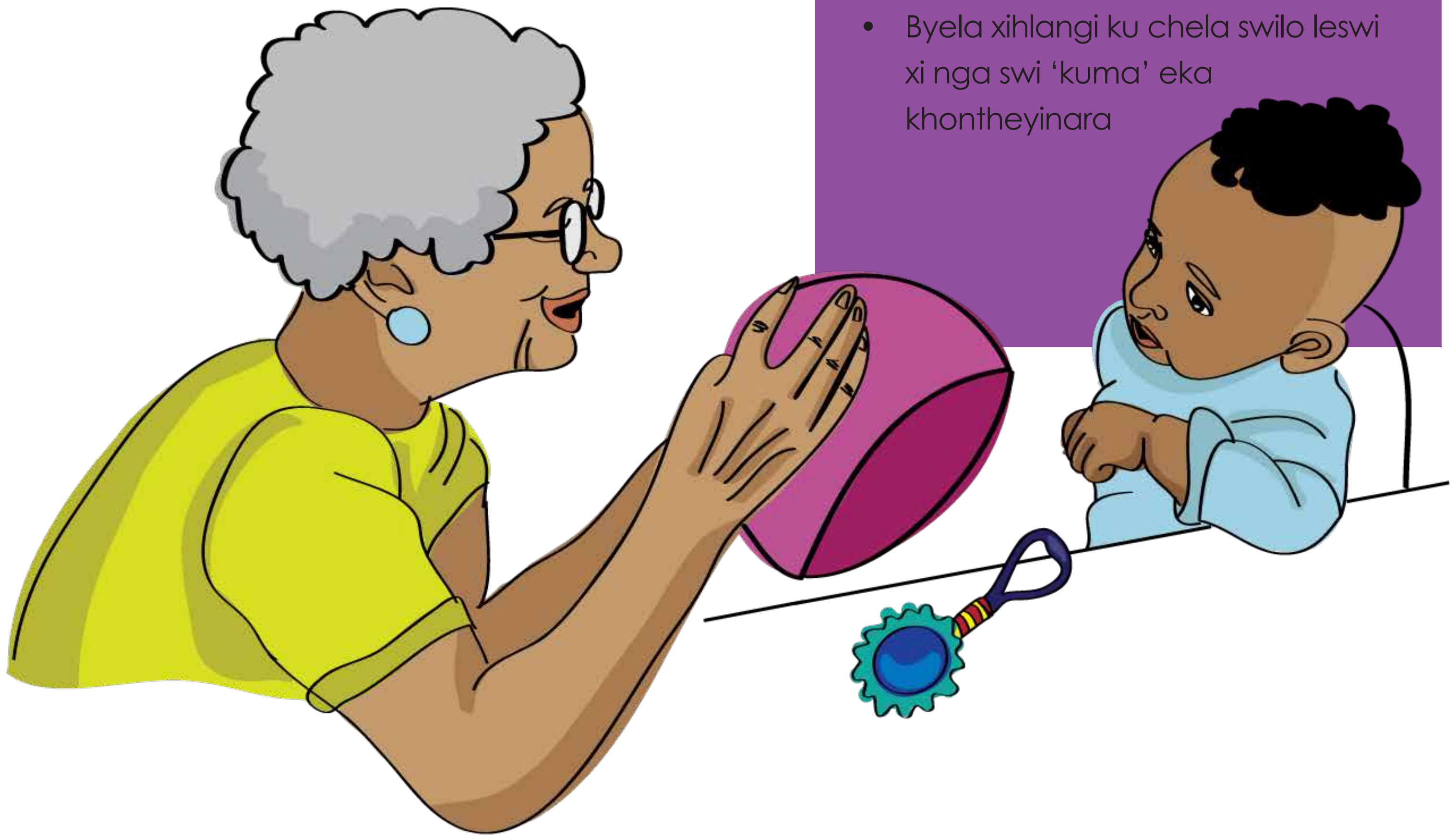

Child Activity

- Raloka papadi ya ‘fihla selo’ le lesea
- E re lesea le bee dilo tša ‘go pinyeletšwa’ ka khontheineng

- Play ‘hide object’ with baby
- Have child put ‘pinch’ objects in container

- Tlanga ntlangu wa ku ‘tumbeta xilo’ na xihlangi
- Byela xihlangi ku chela swilo leswi xi nga swi ‘kuma’ eka khontheyinara

Child Milestones

- Lesea le ka namela dilo goba go emelela
- Lesea le ka tseba selo le sa se bone

- Baby might pull up or stand
- Baby might know object without seeing

- Xihlangi xi nga ringeta ku yima
- Xihlangi xi nga tiva swilo handle ka ku swi vona

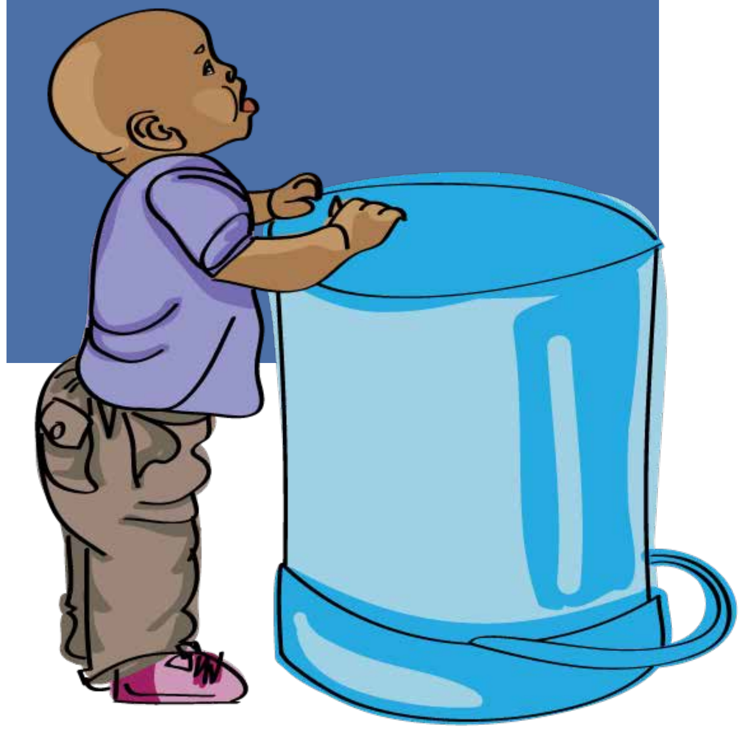

Child Health

- E ba le tsebo ka ga go gohlola le bothata bja go hema
- Hwetša kalafo ka potlako ge lesea le na le bothata bja go hema

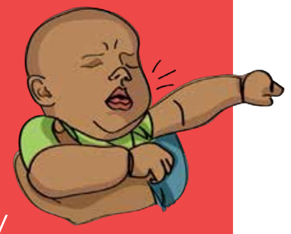

- Look for cough and difficulty breathing
- Seek care immediately if baby has difficulty breathing

- Kamba ku khohlola kumbe ku tik-eriwa ka ku hefemula
- Lava ku pfuniwa hi xihatla loko n’wana a tikeriwa ku hefemula

Child Nutrition

- Go nyantšha letswele + go fa dijo makga a 3
- Kgonthiša gore meetse a go nwa a hlwekile

- Breastfeeding + 3 meals
- Make sure drinking water is clean

- Ku mamisa vele + miphamelo yi 3
- Tiyisisa leswaku mati yo nwa ya basile

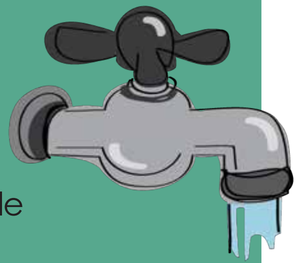

Start

Greet parent and explain purpose of visit.

Ask how parent and child are doing?

Ask caregiver if they have been trying any of the activities since last visit?

Ask if parent can show one of the activities?

Ask if mother/family has any difficulties/challenges in implementing the advice or activities?

Ask if mother/family observed any benefits in implementing the advice or activities?

Ask mother/family if she has any concerns (e.g. child is sick, appears slower to develop)?

Child Health

- Check baby for signs of cough and respiratory infection and demonstrate for mom how she can also check. Encourage mom to pay close attention to baby for respiratory symptoms.
- Encourage mom to seek care immediately if baby has danger signs of respiratory infection, including fast breathing and wheezing sounds. Explain that symptoms may be a sign of pneumonia or other infection and that the clinic has medicines that can help.

Child Nutrition

- At this age, baby's daily diet should include 3 meals of food and breastfeeding for all other meals. Discuss with mom a plan for the 3 food meals and continued breastfeeding.
- Explain to mom that dirty drinking water can make baby sick. Ask mom about current water source and discuss best ways to keep water clean.

Child Milestones

- Baby might pull herself up on household furniture or start to stand. Demonstrate how mom can look for this.
- Baby might know where objects are without directly looking at them. Demonstrate how mom can look for this.

Child Activity

- Baby might know objects without directly looking at them. The 'hide object' game is a way to build this skill. Demonstrate game to mom: put cover over toy, and show baby how to find toy under cover.
- Baby should now be able to hold and move small objects by pinching them between their thumb and index finger. Demonstrate how mom can give baby smaller 'pinch' objects and have baby place the objects in a container.

Observe parent and child trying activity. If needed demonstrate again to help caregiver

Ask if the caregiver has any questions or concerns?
Arrange next visit date

Thank mother and family. Encourage mother to try implement the advice and activities.

Note any questions asked, action needed, referral required, or things to discuss with supervisor/team leader

End

S

E

T

Child Health

- Ditšhwaana tša thibela malwetši di šireletša ngwana
- Maphelo a ama kgodišo ya lesea

- Vaccines protect baby
- Health affects baby's develop-ment
-
- Misawutiso yi sirhelela n'wana
- Rihanyu ri khumba makulele ya xihlangi

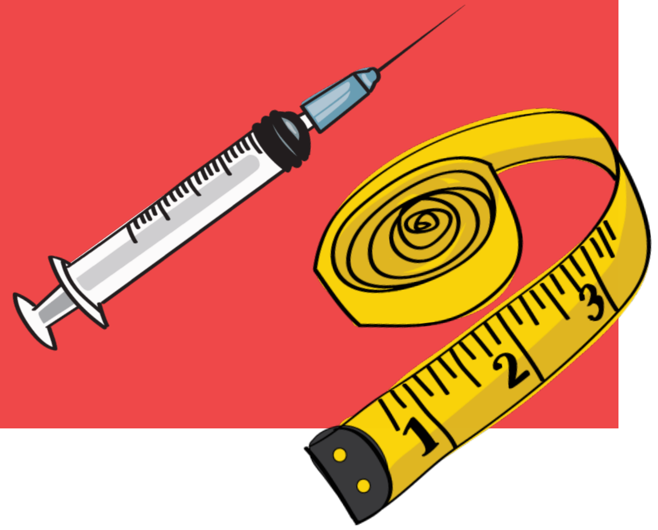

Child Milestones

- Lesea le ka sepela ka go phakiša
- Lesea le ka leka go bolela

- Baby might move faster
- Baby might to try to talk

- Xihlangi xi nga famba hi ku hatlisa
- Xihlangi xi nga ringeta ku vulavula

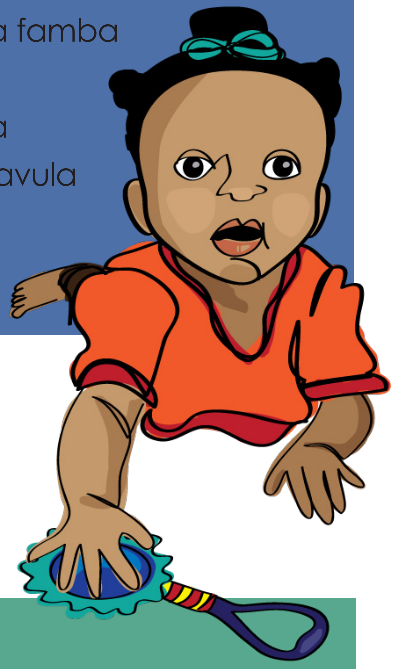

Child Nutrition

- Phepo e na le seabe go kgolo ya lesea
- Naa lesea le swanetše go ba lesea nako e kaakang?

- Nutrition affects baby's devel-opment
- How long should baby be?

- Tinyutiriyente ti khumba makulele ya xihlangi
- Xana xihlangi xi fanele ku va na vulehi byo tani hi kwihi?

Child Activity

- Ruta lesea maina a diphoofolo le medumo
- Raloka papadi ya 'naa [selo] se kae?'

- Teach baby animal names and sounds
- Play game 'where is the [object]?'

- Dyondzisa xihlangi mavito na mipfumawulo ya swifuwo
- Tlanga ntlangu wa '[xilo] xi le kwini?'

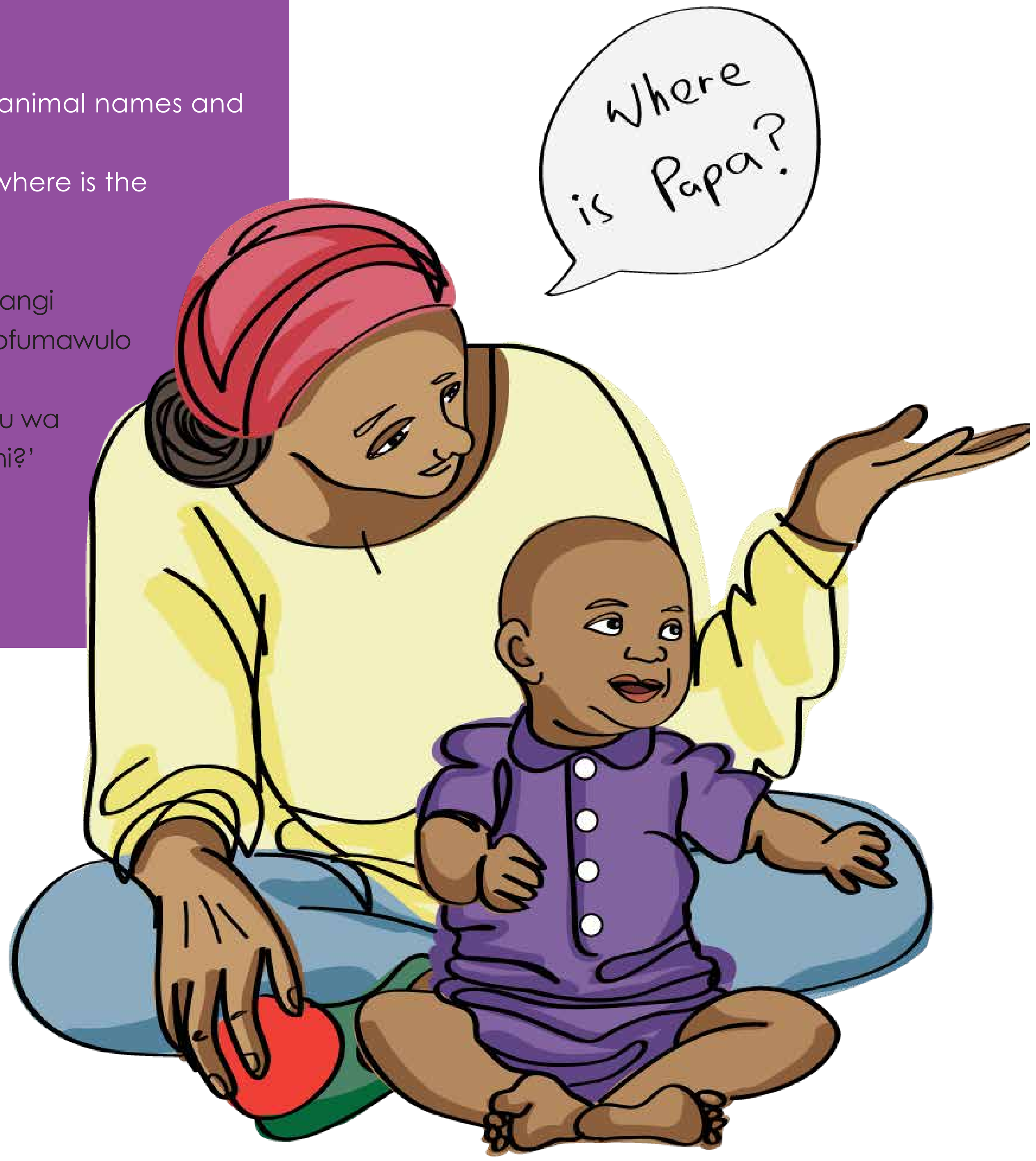

Start

Greet parent and explain purpose of visit.

Ask how parent and child are doing?

Ask caregiver if they have been trying any of the activities since last visit?

Ask if parent can show one of the activities?

Ask if mother/family has any difficulties/challenges in implementing the advice or activities?

Ask if mother/family observed any benefits in implementing the advice or activities?

Ask mother/family if she has any concerns (e.g. child is sick, appears slower to develop)?

Child Health

- At 9 months old, babies should receive measles1 and PCV3. Confirm that the baby has received these vaccines. If not yet received, encourage mom to bring baby to the clinic for these vaccines immediately.
- Baby's health affects physical growth and development. It is important that mom seeks care for baby when baby has symptoms of illness. Even if symptoms do not seem to serious, untreated infections may slow the baby's development over time. Mom should take baby to the clinic for a general checkup every three months or so. Ask mom if baby has been checked recently, and if not encourage mom to take baby to clinic for a checkup.

Child Nutrition

- Baby's nutrition affects physical growth and development. It is important that baby has enough protein and micronutrients in their diet. If they do not, their growth may be slowed.
- Baby's length is an important sign of their development, and is related to things like how the baby will perform in school later. Ask mom if baby's length has been measured recently (check Road to Health Booklet) and if baby is stunted. Talk with mom about how baby's nutrition might be improved to ensure better growth.

Child Milestones

- Baby might move faster and start to explore more. Demonstrate how mom can look for this.
- Baby might try to talk but will likely not be able to form clear words. Demonstrate how mom can look for this.

Child Activity

- Encourage mom to introduce baby to animals that can be found in the village, and to teach baby the names of the animals and the sounds that they make.
- Now that baby is more mobile, the 'where is the [object]?' game can expand. Mom can name objects that are not directly in front of baby, but that baby has to search for and find. Encourage mom to play this expanded form of the game.

Observe parent and child trying activity. If needed demonstrate again to help caregiver

Ask if the caregiver has any questions or concerns?
Arrange next visit date

Thank mother and family. Encourage mother to try implement the advice and activities.

Note any questions asked, action needed, referral required, or things to discuss with supervisor/team leader

End

S
E
T

Child Activity

- Boledišanang gore ngwana a ka ba a nyaka eng ge a šupa
- Raloka papadi ya 'naa [selo] se kae?' gomme o kgopele ngwana gore a šupe

- Talk about what child wants if pointing
- Play game 'where is the [object]?' and ask child to point

- Vulavulani hi leswi xihlangi xi swi lavaka loko xi swi kombetela
- Tlangani ntlawu wa '[xilo] xi le kwihi?' kutani u kombela xihlangi leswaku xi swi kombetela

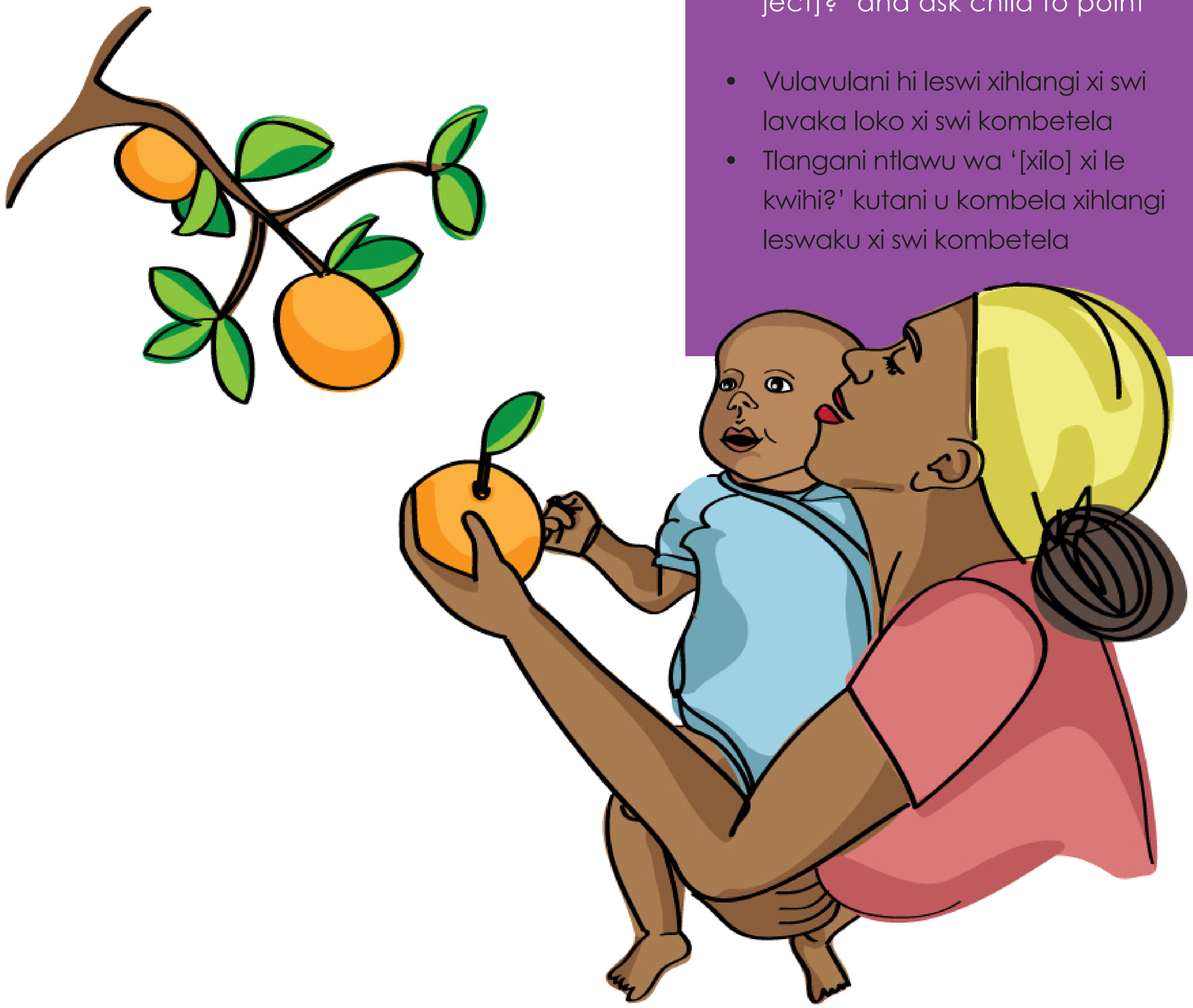

Child Nutrition

- Go nyantšha letswele + go fa dijo makga a 4
- Leka motswako o moswa

- Breastfeeding + 4 meals
- Try a new recipe

- Ku mamisa vele + miphamelo ya Mune.
- Ringeta rhesipi yintswa

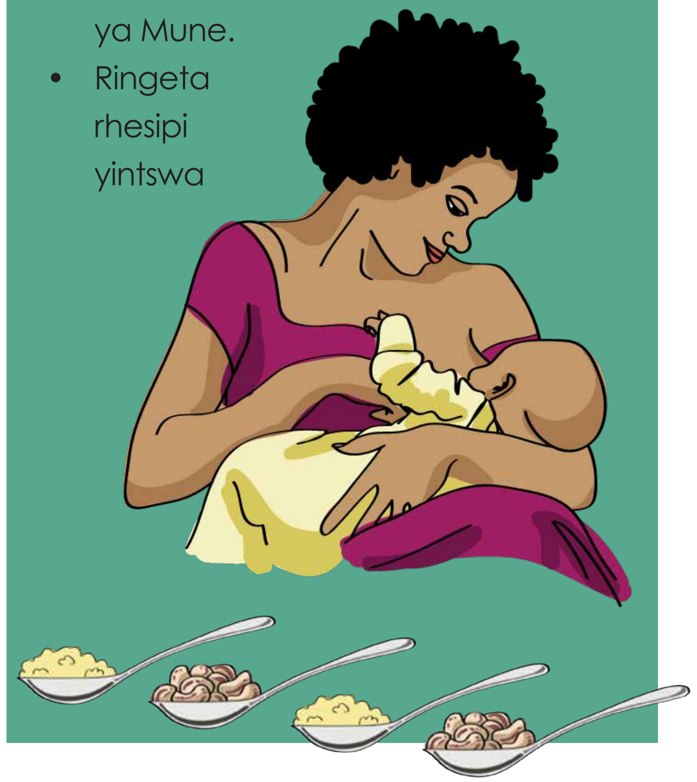

Child Health

- Kgonthiša gore ngwana o bolokegile ka ntlong

- Make house safe for child

- Endla leswaku yindlu yi va yo hlayiseka eka xihlangi

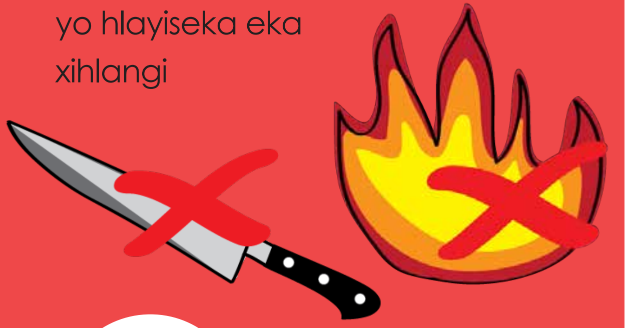

Child Milestones

- Ngwana a ka šupa dilo tše a di nyakago
- Ngwana a ka latela ditaelo tše bonolo go swana le 'go emelela' goba 'go ema'

- Child might point at objects they want
- Child might follow simple commands like 'stand up' or 'stop'

- Xihlangi xi nga kombetela swilo leswi xi swi lavaka
- Xihlangi xi nga landzelela swileriso swo olova swo tanihi 'tlakuka' kumbe 'yima'

Start

Greet parent and explain purpose of visit.

Ask how parent and child are doing?

Ask caregiver if they have been trying any of the activities since last visit?

Ask if parent can show one of the activities?

Ask if mother/family has any difficulties/challenges in implementing the advice or activities?

Ask if mother/family observed any benefits in implementing the advice or activities?

Ask mother/family if she has any concerns (e.g. child is sick, appears slower to develop)?

Child Health

- The child is becoming increasingly mobile and is exploring the house more and more. Walk around the house with mom and discuss ways in which the child might face danger or have an accident, and talk with mom about how she can make the house safer for the child.

Child Nutrition

- At this age, child's daily diet should include 4 meals of food and breastfeeding for all other meals. Discuss with mom a plan for the 4 food meals and continued breastfeeding.
- Discuss a new meal that mom can cook for child. Ask about child's preferences for food and make tailored suggestions.

Child Milestones

- Child might point at objects that they want. Demonstrate how mom can look for this.
- Child might follow simple commands like 'stand up' or 'stop'. Demonstrate how mom can check this.

Child Activity

- Encourage mom to talk to child when child points at objects they want. Mom can have a short conversation with child, saying 'do you want [object]?' going through a few objects until the child indicates the correct object is named.
- Encourage mom to play 'where is the [object]?' game but to have child point to the object that mom names.

Observe parent and child trying activity. If needed demonstrate again to help caregiver

Ask if the caregiver has any questions or concerns?
Arrange next visit date

Thank mother and family. Encourage mother to try implement the advice and activities.

Note any questions asked, action needed, referral required, or things to discuss with supervisor/team leader

End

Child Health

- Ngwana o hloka maphelo a mabotse
- Dira gore go tsene moya wo o hlwekilego ka gae

- Child needs good hygiene
- Ventilate home to keep air clean

- Xihlangi xi lava nsivelamavabyi wa kahle
- Vona leswaku yindlu yi nghena moya wo tenga na ku vona leswaku moya wu tshama wu tengile

Child Nutrition

- Go nyantšha letswele + go fa dijo makga a 5
- Leka motswako o moswa

- Breastfeeding + 5 meals
- Try a new recipe

- Ku mamisa vele + miphamelo ya nthlanu
- Ringeta rhesipi yintswa

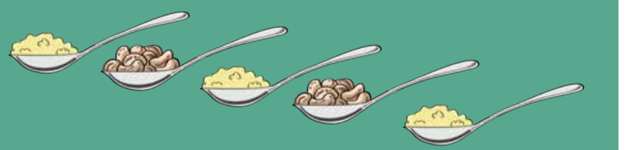

Child Milestones

- Ngwana a ka ganetša ka go šikinya hlogo
- Ngwana a ka sepela a thekgilwe

- Child might shake head 'no'
- Child might walk with support

- Xihlangi xi nga kombisa ku 'ala' hi ku dzungudza nhloko
- Xihlangi xi nga deyisiwa

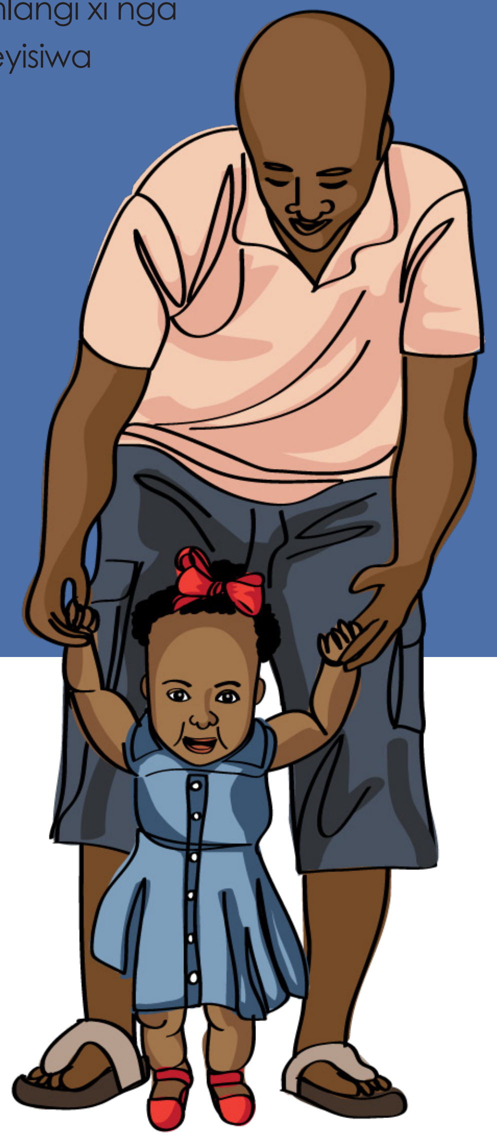

Child Activity

- E re ngwana a hlophe dilo
- Balela ngwana

- Have child stack objects
- Read to child

- Byela xihlangi ku paka swilo
- Hlayela xihlangi

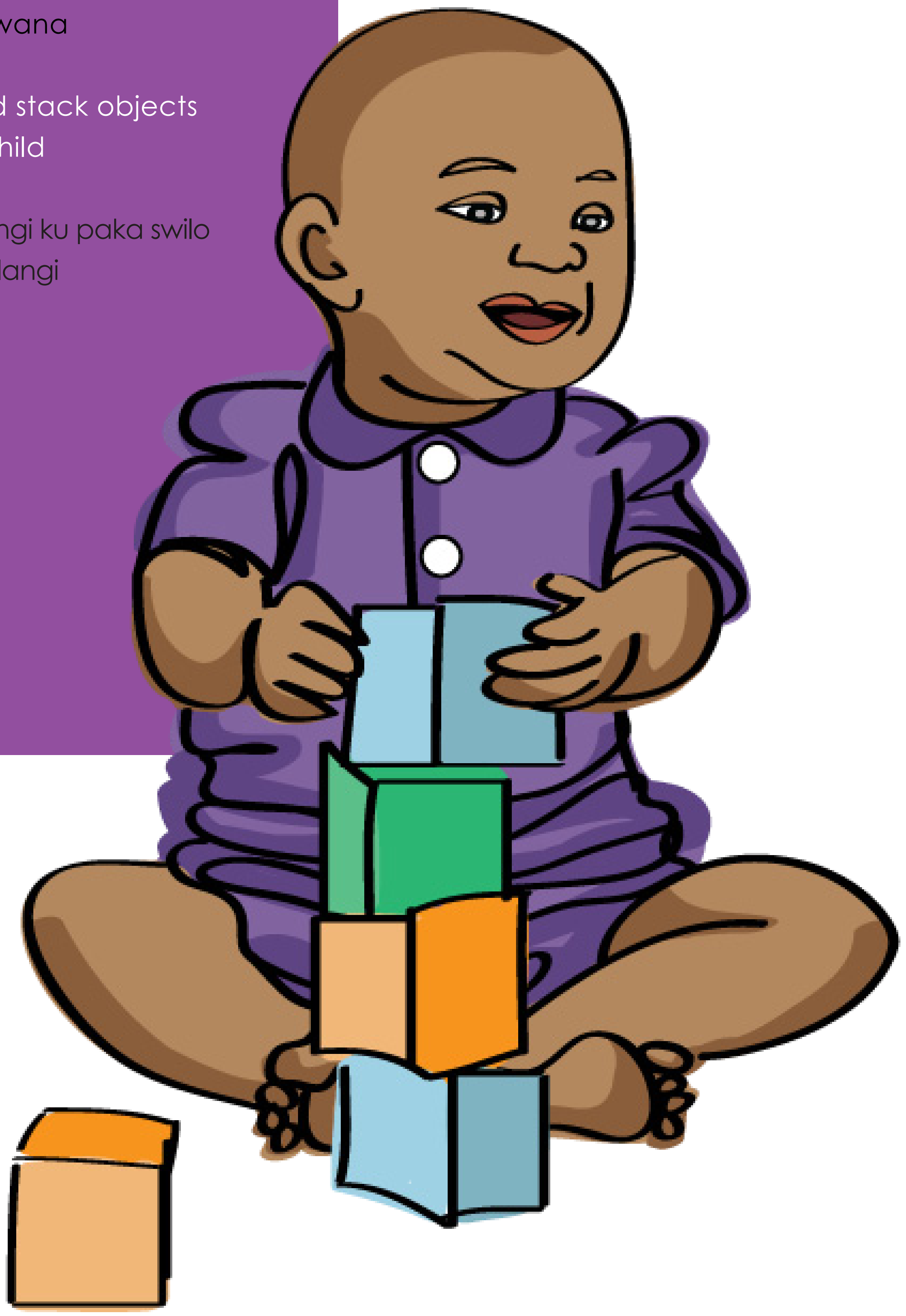

Start

Greet parent and explain purpose of visit.

Ask how parent and child are doing?

Ask caregiver if they have been trying any of the activities since last visit?

Ask if parent can show one of the activities?

Ask if mother/family has any difficulties/challenges in implementing the advice or activities?

Ask if mother/family observed any benefits in implementing the advice or activities?

Ask mother/family if she has any concerns (e.g. child is sick, appears slower to develop)?

Child Health

- Child may now handle their bathroom visits themselves. The child needs to practice good hygiene to avoid infections. Talk with mom and child about adopting good habits.
- Without proper ventilation, air in the home can contribute to illness. This is especially true if cooking is done in the home. Particles from cook stoves can make child sick over time. Ask mom to explain home ventilation and encourage better practices for ventilation if appropriate.

Child Nutrition

- At this age, child's daily diet should include 5 meals of food and breastfeeding for all other meals. Discuss with mom a plan for the 5 food meals and continued breastfeeding.
- Discuss a new meal that mom can cook for child. Ask about child's preferences for food and make tailored suggestions.

Child Milestones

- Child might shake head 'no'. Demonstrate how mom can check for this.
- Child might walk with or without support. Demonstrate how mom can check this.

Child Activity

- Help mom find a few objects in home that can be stacked. Demonstrate how mom can work with the child to stack objects and encourage mom to play this game with child each day.
- Mom should start to read things to the child. If home has a book, mom can read book to child. If no book, mom should read written materials in town (e.g., signs or posters) when possible.

Observe parent and child trying activity. If needed demonstrate again to help caregiver

Ask if the caregiver has any questions or concerns?
Arrange next visit date

Thank mother and family. Encourage mother to try implement the advice and activities.

Note any questions asked, action needed, referral required, or things to discuss with supervisor/team leader

End

S
E
T

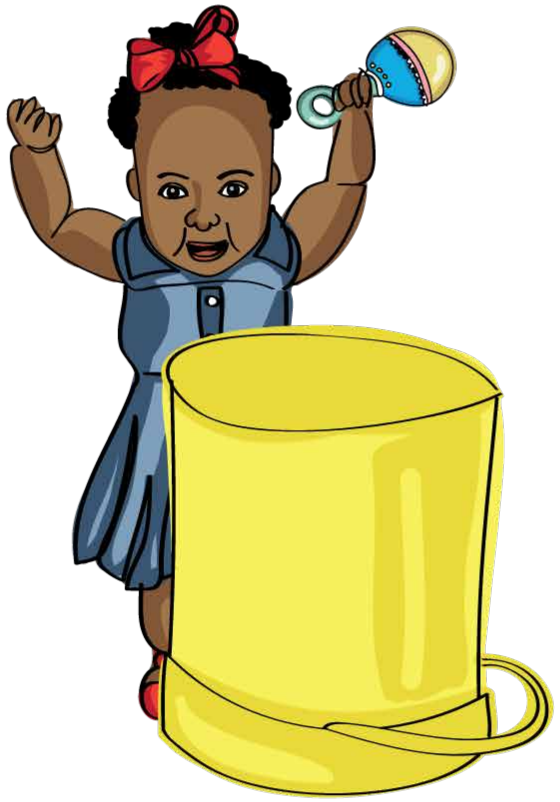

Child Activity

- E re ngwana a bethe dilo go swana le moropa
- Ikotlolle le ngwana ka maoto le ete le bolela ka go bona mafelo

- Have child bang on objects like a drum
- Take child for walk and talk about sights

- Tshika xihlangi xi chaya swilo ku fana na ngoma
- Huma na xihlangi mi fambanyana na ku vulavula hi swilo leswi mi swi vonaka

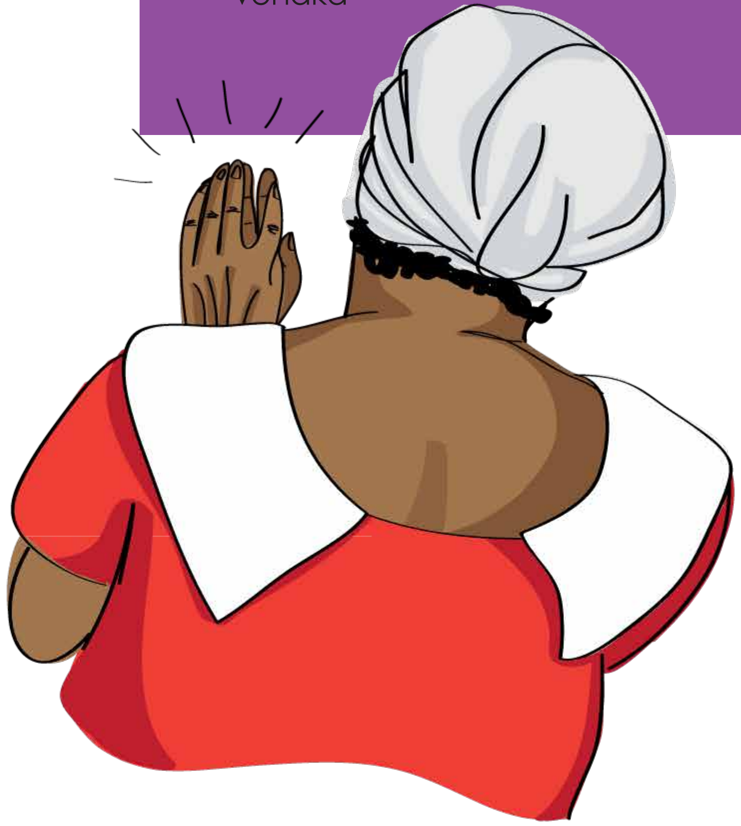

Child Nutrition

- Phepo e ama go gola ga ngwana
- Ngwana o hloka protheine
- Ngwana o swanetše go ja dinawa le mae

- Nutrition affects child's development
- Child needs protein
- Child should eat beans and eggs

- Tinyutiriyente ti khumba makulele ya n'wana
- N'wana u lava phurotheyini
- N'wana u fanele ku dya tinyawa na matandza

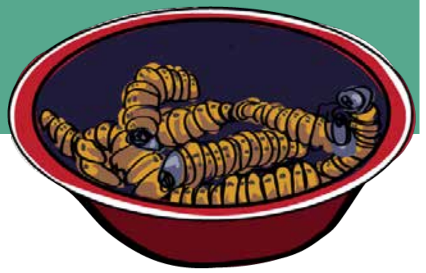

Child Health

- Maphelo a ama kgodišo ya ngwana

- Health affects child's development

- Rihanyu ri khumba makulele ya n'wana

Child Milestones

- Go lakaletša letšatši la matswalo!
- Ngwana a ka sepela a se a thekgwa

- Happy birthday!
- Child might walk without support

- Siku ra kahle ro velekiwa!
- Xihlangi xi nga kota ku famba handle ka nseketelo

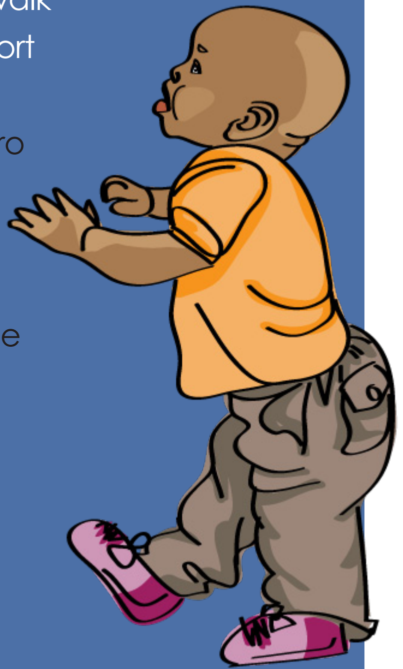

Start

Greet parent and explain purpose of visit.

Ask how parent and child are doing?

Ask caregiver if they have been trying any of the activities since last visit?

Ask if parent can show one of the activities?

Ask if mother/family has any difficulties/challenges in implementing the advice or activities?

Ask if mother/family observed any benefits in implementing the advice or activities?

Ask mother/family if she has any concerns (e.g. child is sick, appears slower to develop)?

Child Health

- Child's health affects physical growth and development. It is important that mom seeks care for child when child has symptoms of illness. Even if symptoms do not seem too serious, untreated infections may slow the child's development over time. Mom should take child to the clinic for a general checkup every three months or so. Ask mom if child has been checked recently, and if not encourage mom to take child to clinic for a checkup.

Child Nutrition

- Child's nutrition affects physical growth and development. It is important that child has enough protein and micronutrients in their diet. If they do not, their growth may be slowed.
- The child needs to consume a good amount of protein through their diet in order for their body to develop properly. Protein will help them grow taller and stronger.
- A few good sources of protein are beans and eggs. Encourage mom to incorporate beans or eggs into the child's diet as much as possible.

Child Milestones

- Child is now one-year-old and is likely twice as large as she was when born.
- Child might walk without support now. Demonstrate how mom can check this.

Child Activity

- Help mom find an object in the home that can be banged on like a drum. Demonstrate how mom can work with the child to play this game with child each day.
- Encourage mom to walk with child around village or town and talk to child about the things they see.

Observe parent and child trying activity. If needed demonstrate again to help caregiver

Ask if the caregiver has any questions or concerns?
Arrange next visit date

Thank mother and family. Encourage mother to try implement the advice and activities.

Note any questions asked, action needed, referral required, or things to discuss with supervisor/team leader

End

S
E
T

Child Health

- Hlapiša ngwana diatla pele ga ge a eja
- Hlapiša ngwana le go fetoša diaparo letšatši le lengwe le le lengwe

- Wash child's hands before they eat
- Bath child and change clothes every day

- Hlambisa xihlangi swandlla xi nga si dya
- Hlambisa xihlangi miri na ku xi cin-ca swiambalo masiku hinkwawo

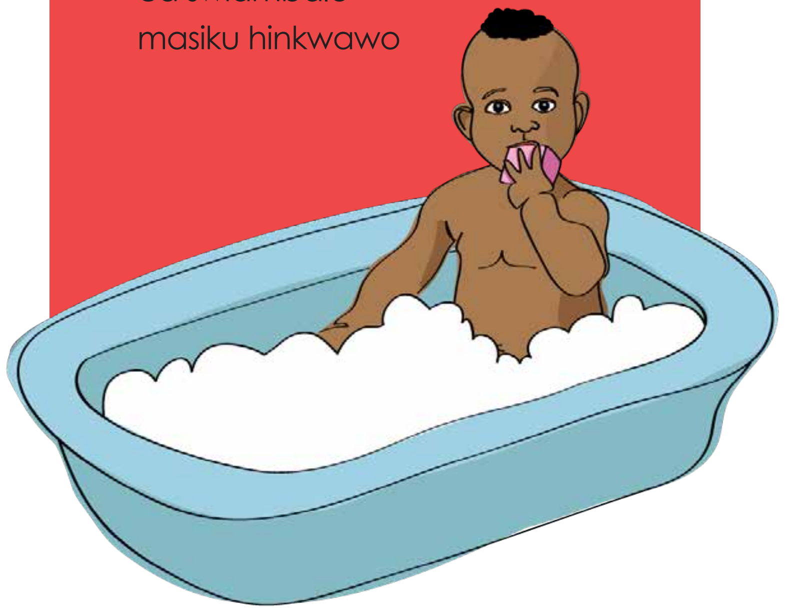

Child Milestones

- Ngwana a ka šomiša lehwana le komiki
- Ngwana a ka rata go thalathala

- Child might use spoon and cup
- Child might like to draw

- Xihlangi xi nga tirhisa lepula kumbe khapu
- Xihlangi xi nga tsakela ku dirowa

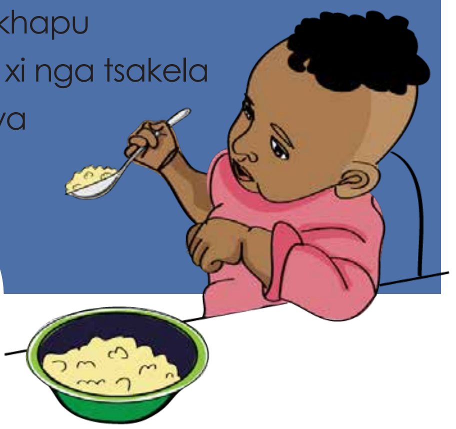

Child Nutrition

- Ngwana o hloka bitamine A
- Ngwana o swanetše go ja mero-go ye metala ya mahlare

- Child needs vitamin A
- Child should eat leafy green vegetables

- N'wana u lava vhitamini A
- N'wana u fanele ku dya matsavu yo tsakama

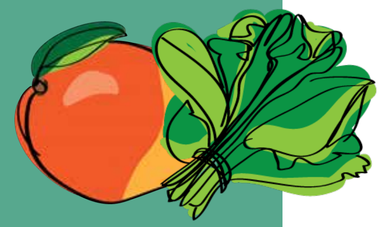

Child Activity

- Bitša leina la selo gore ngwana a se ntšhe ka khontheineng
- E re ngwana a thalathale ka phensele/kherayone

- Name object for child to pick from container
- Have child scribble with a pencil/crayon

- Vula mavito ya swilo leswaku xihlangi xi swi teka eka khontheyinara
- Byela xihlangi ku mpfampfarhuta hi penisele/khirayoni

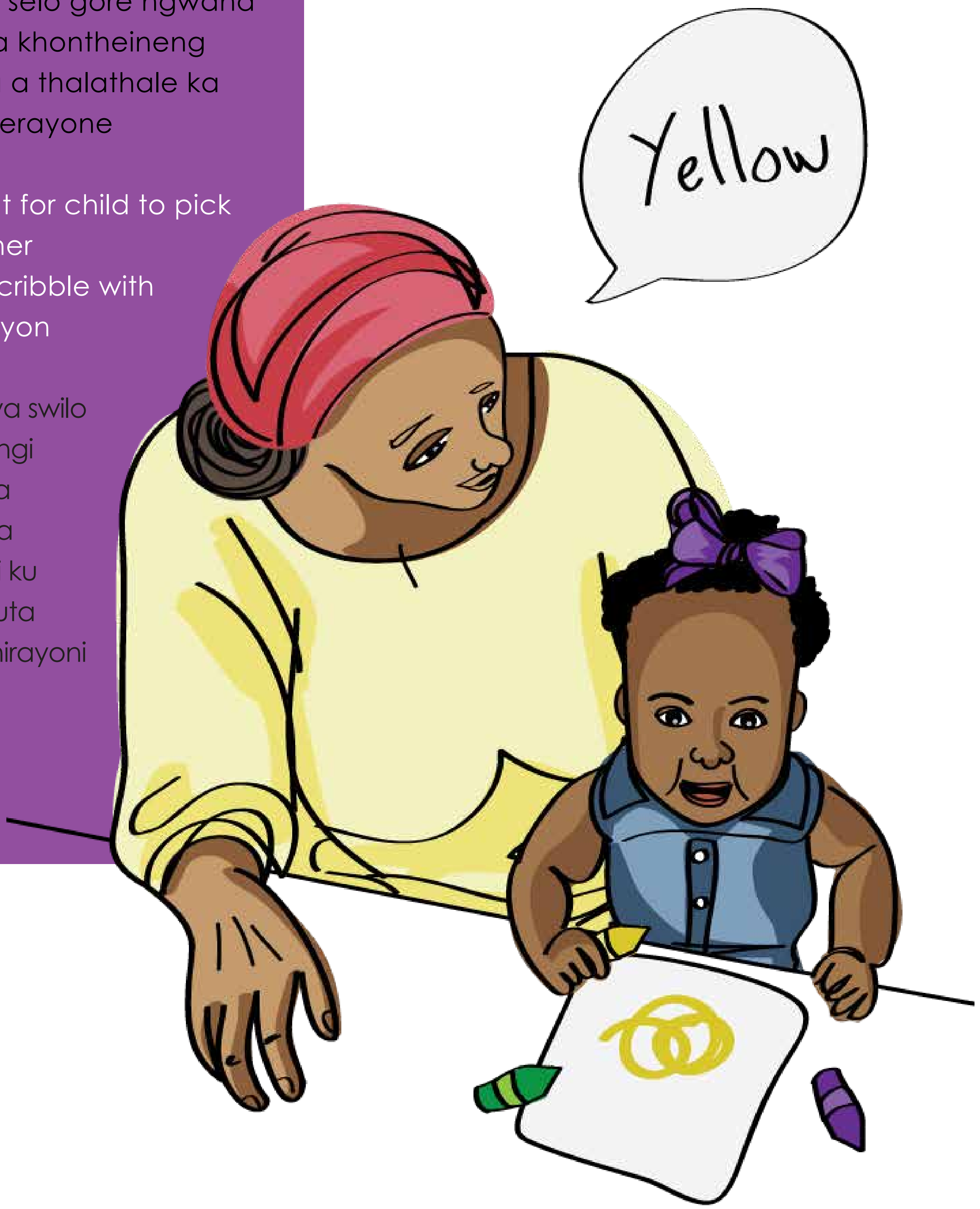

Start

Greet parent and explain purpose of visit.

Ask how parent and child are doing?

Ask caregiver if they have been trying any of the activities since last visit?

Ask if parent can show one of the activities?

Ask if mother/family has any difficulties/challenges in implementing the advice or activities?

Ask if mother/family observed any benefits in implementing the advice or activities?

Ask mother/family if she has any concerns (e.g. child is sick, appears slower to develop)?

Child Health

- Demonstrate for mom how they can wash the child's hands. Encourage mom to wash the child's hands before every meal. This will reduce the bacteria the child consumes that might make them sick.
- As the child moves and explores more, they will get dirty more quickly and will also be exposed to more bacteria. Explain to mom that the child needs to be cleaned more often to ensure good health.

Child Nutrition

- The child needs to consume a good amount of vitamin A through their diet in order for their body to develop correctly. In particular, vitamin A will help them fight infections.
- One good source of vitamin A is leafy green vegetables. Encourage mom to incorporate leafy green vegetables into the child's diet as much as possible.

Child Milestones

- Child might use a spoon and cup when eating and drinking. Demonstrate how mom can check for this.
- Child might like to draw with pencils or crayons. Demonstrate how mom can check this.

Child Activity

- Demonstrate for mom how to put a few objects in a container and then place the container in front of child and name an object for the child to pick from the container. Encourage mom to play this game every day.
- Ask mom if she has any pencils or crayons and paper that the child could use to draw. If yes, demonstrate how the child can draw on a paper.

Observe parent and child trying activity. If needed demonstrate again to help caregiver

Ask if the caregiver has any questions or concerns?
Arrange next visit date

Thank mother and family. Encourage mother to try implement the advice and activities.

Note any questions asked, action needed, referral required, or things to discuss with supervisor/team leader

End

S
E
T

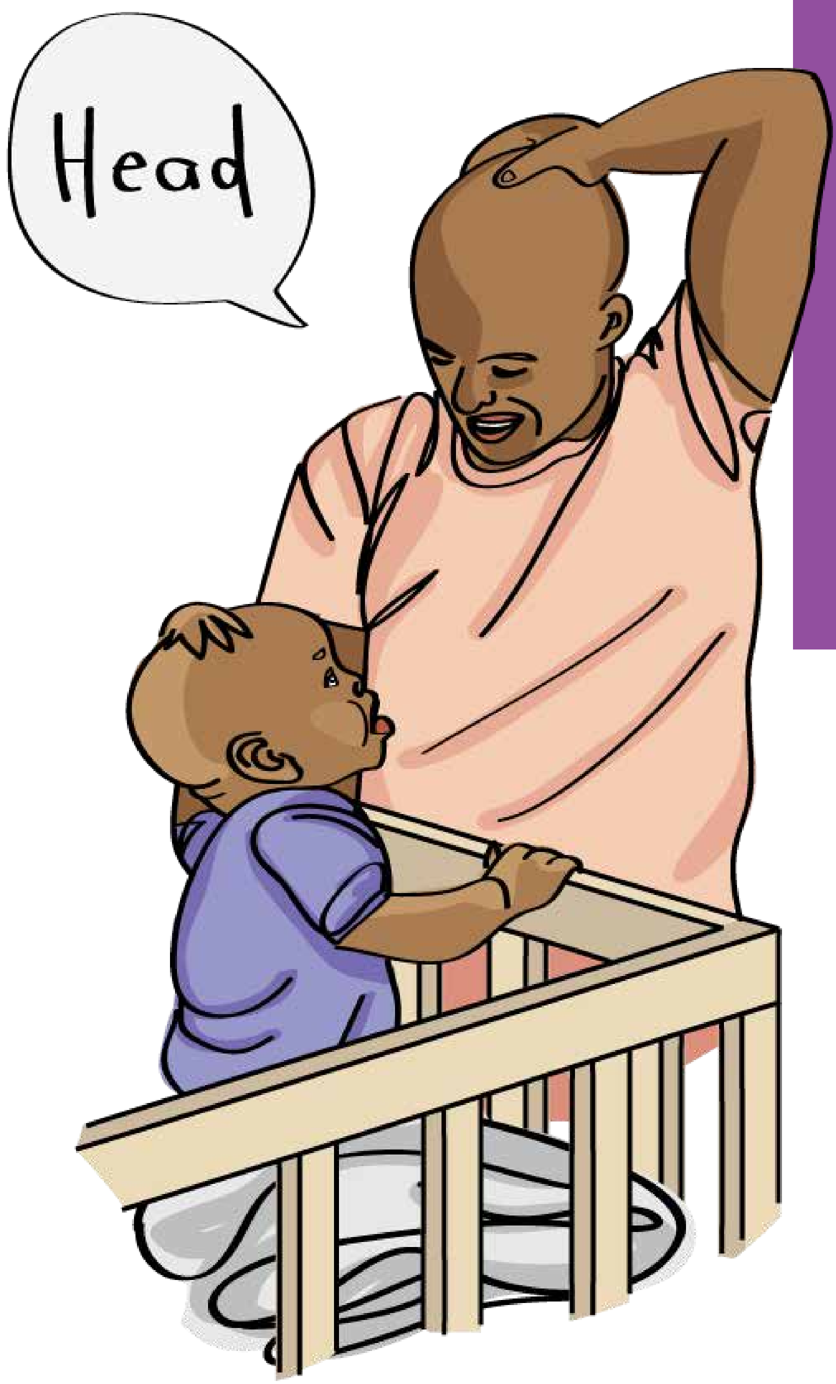

Child Activity

- Bitša maina a ditho tša mmele gomme o kgopele lesea gore le di šupe
- Bušeletša seo lesea le se bolelago gomme o tlaleletše mantšu

- Say body parts and ask baby to point to them
- Repeat what baby says and add words

- Vula mavito ya swirho swa miri kutani u kombela xihlangi ku swi komba
- Vuyelela leswi xihlangi xi swi vulaka kutani u engetela marito

Child Milestones

- Ngwana a ka bolela mantšu a mmalwa
- Ngwana a ka foša goba a raga dilo bjalo ka kgwele

- Child might say a few words
- Child might throw or kick objects like a ball

- Xihlangi xi nga vula marito mo ka ma nga talangi
- Xihlangi xi nga hoxa kumbe ku raha swilo swo tanihi bolo

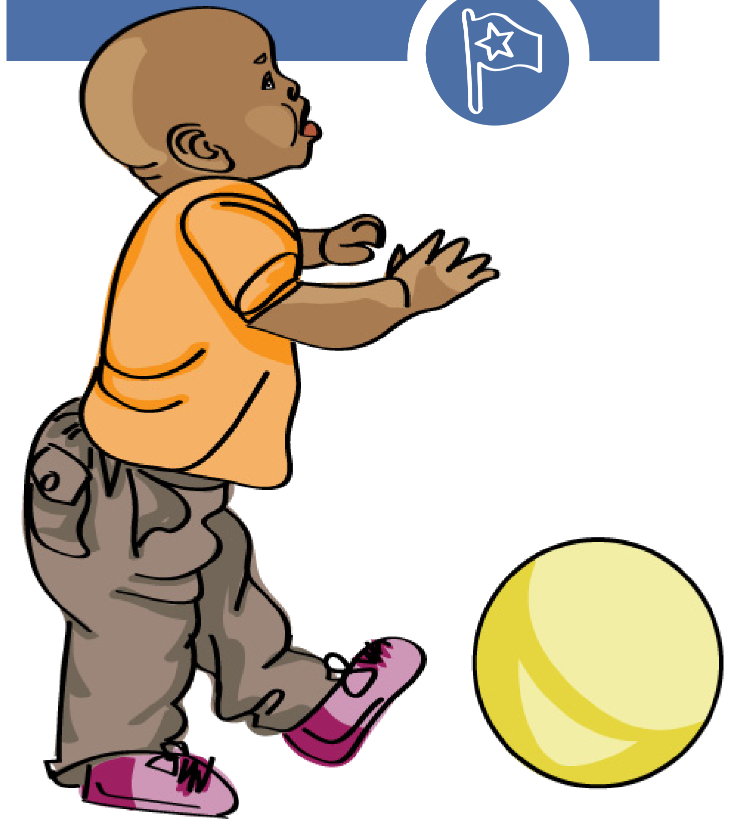

Child Health

- E ba le tsebo ka dika
- Hwetša kalafo ka potlako ya bolwetši

- Look for symptoms
- Seek care immediately for illness

- Languta swikombo
- Lava vutshunguri hi xihatla loko ku ri na mavabyi

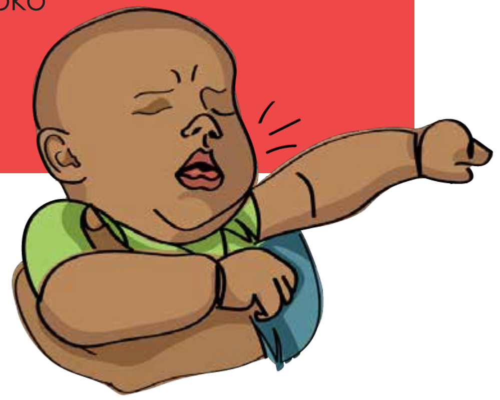

Child Nutrition

- Ngwana o hloka ayone
- Ngwana o swanetše go ja dinawa

- Child needs iron
- Child should eat beans

- N'wana u lava ayoni
- N'wana u fanele ku dya tinyawa

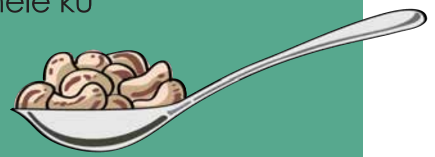

Start

Greet parent and explain purpose of visit.

Ask how parent and child are doing?

Ask caregiver if they have been trying any of the activities since last visit?

Ask if parent can show one of the activities?

Ask if mother/family has any difficulties/challenges in implementing the advice or activities?

Ask if mother/family observed any benefits in implementing the advice or activities?

Ask mother/family if she has any concerns (e.g. child is sick, appears slower to develop)?

Child Health

- Check child for symptoms and demonstrate for mom how she can also check. Encourage mom to pay close attention to child for symptoms.
- Encourage mom to seek care immediately if child has symptoms. Explain that even if symptoms seem non-severe, they may slow the child's development over time. Explain that the clinic has medicines that can help.

Child Nutrition

- The child needs to consume a good amount of iron through their diet in order for their body to develop correctly. In particular, iron will help their brain develop.
- One good source of iron is beans. Encourage mom to incorporate beans into the child's diet as much as possible.

Child Milestones

- Child might say a few words. Demonstrate how mom can check for this.
- Child might throw or kick objects like a ball. Demonstrate how mom can check for this.

Child Activity

- Demonstrate for mom how she can play with the child by naming parts of the body and asking the child to point to that part on their own body.
- Encourage mom to talk to the child when the child says a word by adding words. For example, if baby says 'ball' mom can say 'yes, great, this is a red ball, the ball is round'

Observe parent and child trying activity. If needed demonstrate again to help caregiver

Ask if the caregiver has any questions or concerns?
Arrange next visit date

Thank mother and family. Encourage mother to try implement the advice and activities.

Note any questions asked, action needed, referral required, or things to discuss with supervisor/team leader

End

Child Health

- E ba le tsebo ka ga letšhollo
- Šomiša motswako wa go thibela letšhollo (ORS)

- Look for diarrhea
- Use oral rehydration salt (ORS) solu-tion

- Languta nchuluko
- Tirhisa diripi (oral rehydration salt [ORS])

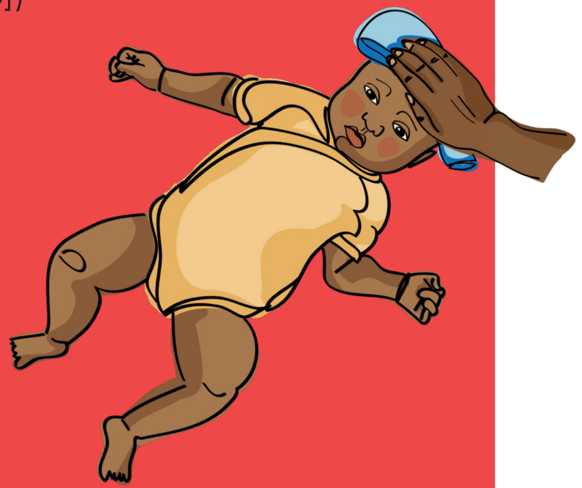

Child Milestones

- Ngwana a ka bolela mantšu a mantši
- Ngwana a ka nwa ka komiki

- Child might say more words
- Child might drink from a cup

- Xihlangi xi nga vula marito yo talanyana
- Xihlangi xi nga n'wa hi khapu

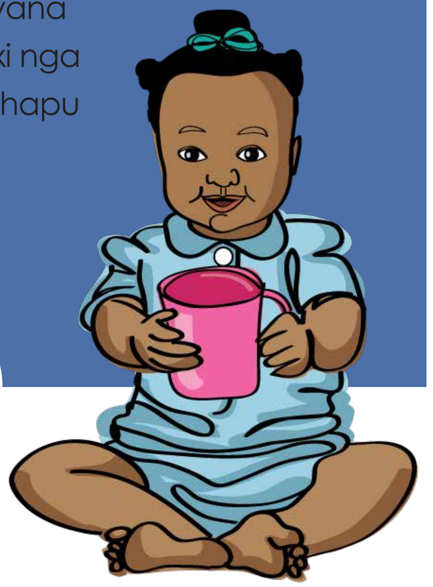

Child Nutrition

- Ngwana o hloka bitamine C
- Ngwana o swanetše go ja dienywa

- Child needs vitamin C
- Child should eat fruits

- N'wana u lava vhitamini C
- N'wana u fanele ku dya mihandzu

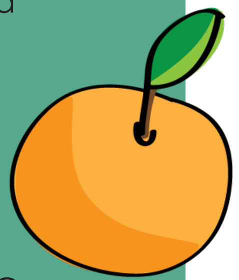

Child Activity

- E tla le ngwana mmarakeng gomme o šupe/bitše maina a dijo
- Kgopela ngwana go go tlela le dilo ge o di bitša
- Balela/biletša ngwana maina a dilo tša ka pukung

- Bring child to market and point/name foods
- Ask child to bring you objects by name
- Read/name objects in book to child

- Yana na xihlangi emakete kutani u kombetela/vula mavito ya swakudya
- Kombela xihlangi ku ku tisela swilo leswi u vulaka mavito ya swona
- Hlayela/byela xihlangi mavito ya swilo ku suka ebukwini

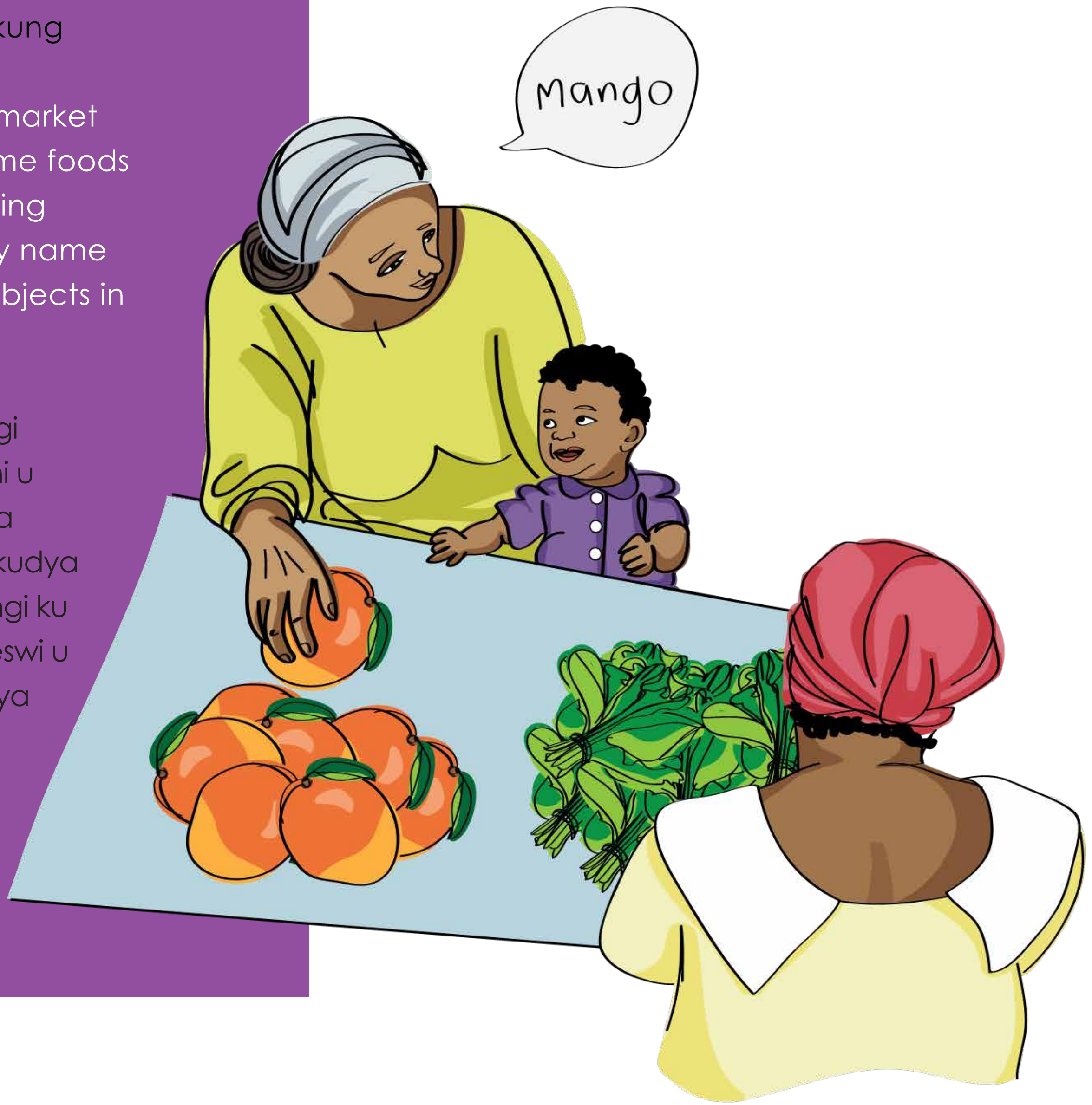

S
E
T

Start

Greet parent and explain purpose of visit.

Ask how parent and child are doing?

Ask caregiver if they have been trying any of the activities since last visit?

Ask if parent can show one of the activities?

Ask if mother/family has any difficulties/challenges in implementing the advice or activities?

Ask if mother/family observed any benefits in implementing the advice or activities?

Ask mother/family if she has any concerns (e.g. child is sick, appears slower to develop)?

Child Health

- As child starts to eat more and more new foods, the risks for diarrhea may increase. Encourage mom to pay close attention to whether child has diarrhea, particularly if baby is showing signs of dehydration or malnutrition due to diarrhea.
- Encourage mom to give child ORS when baby has diarrhea to avoid dehydration. Demonstrate for mom how to make ORS or discuss where she can buy ORS packets nearby.

Child Nutrition

- The child needs to consume a good amount of vitamin C through their diet in order for their body to develop correctly. In particular, vitamin C will help them fight infections.
- One good source of vitamin C is fruit. Encourage mom to incorporate fruit into the child's diet as much as possible.

Child Milestones

- Child might say more and more words each week. Demonstrate how mom can check for this.
- Child might drink from a cup. Demonstrate how mom can check for this.

Child Activity

- Encourage mom to bring child when she goes to the market and to walk around and point and name things for child.
- Demonstrate for mom how she can name objects around the house and ask the child to bring them to her.
- Mom should develop habit of sitting with child and looking at a book. In addition to reading the book, mom can also point at objects in the book and name them for the child. Demonstrate for mom how to do this. Encourage mom to sit with child and book every day.

Observe parent and child trying activity. If needed demonstrate again to help caregiver

Ask if the caregiver has any questions or concerns?
Arrange next visit date

Thank mother and family. Encourage mother to try implement the advice and activities.

Note any questions asked, action needed, referral required, or things to discuss with supervisor/team leader

End

S
E
T

Child Activity

- Opela le ngwana koša ye e bolelago ka ditho tša mmele
- E re ngwana a hlophe dilo gomme a bolele leina/mmala wa tšona
- Balela ngwana letšatši le leng-we le le lengwe

- Sing a song with child about body parts
- Have child stack objects and say name/color
- Read to the child every day

- Yimbelela na xihlangi risimu ra swirho swa miri
- Byela n'wana ku paka swilo na ku vula mavito/ mihlovo ya swona
- Hlayela n'wana masiku hinkwawo

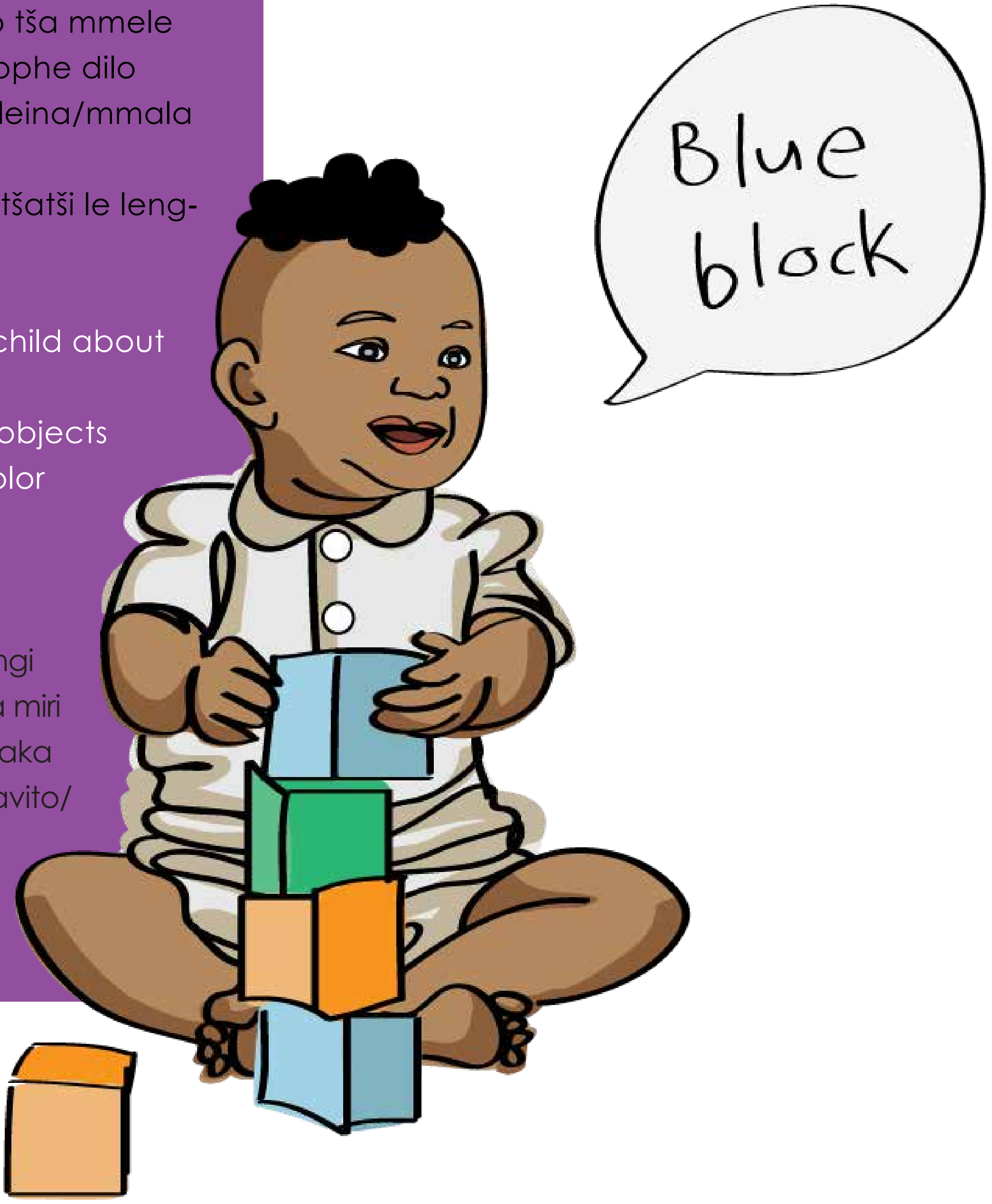

Child Milestones

- Ngwana a ka gopola dikanegelo le dikoša
- Ngwana a ka gakanega gabonolo

- Child might remember stories and songs
- Child might get frustrated easily

- Xihlangi xi nga tsundzuka switori na tinsimu
- Xihlangi xi nga nyangatseka hi ku olova

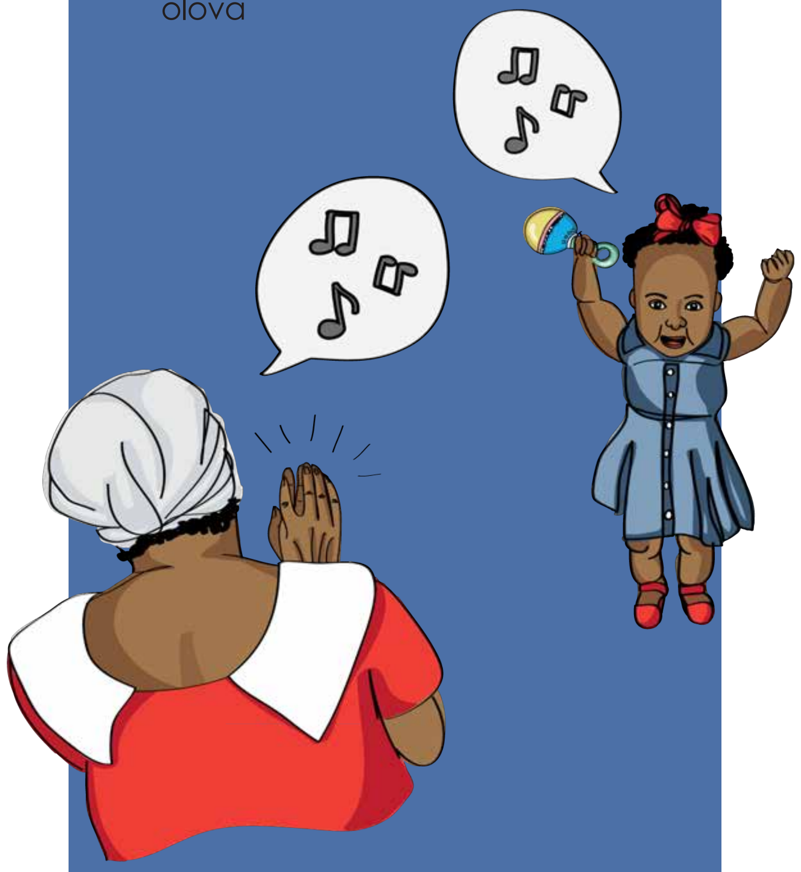

Child Health

- E ba le tsebo ka ga letadi
- Hwetša kalafo ka potlako ge ngwana a na le letadi
- Ngwana o swanetše go khupetšwa ka nete ge a robetše

- Look for fever
- Seek care immediately if child has fever
- Child should sleep under net

- Kamba ku hisa ka miri
- Lava vutshunguri hi xihatla loko n'wana a hisa miri
- N'wana u fanele ku etlela endzeni ka nete

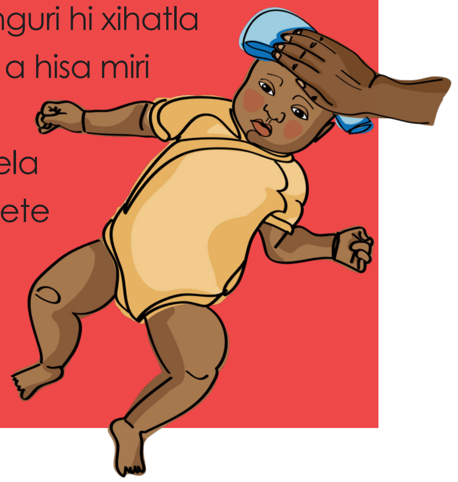

Child Nutrition

- Ngwana o hloka dijo tše ntši
- Leka motswako o moswa

- Child needs many foods
- Try a new recipe

- N'wana u lava swakudya swo tala
- Ringeta rhesipi yintswa

Start

Greet parent and explain purpose of visit.

Ask how parent and child are doing?

Ask caregiver if they have been trying any of the activities since last visit?

Ask if parent can show one of the activities?

Ask if mother/family has any difficulties/challenges in implementing the advice or activities?

Ask if mother/family observed any benefits in implementing the advice or activities?

Ask mother/family if she has any concerns (e.g. child is sick, appears slower to develop)?

Child Health

- Check child for signs of fever and demonstrate for mom how she can also check. Encourage mom to pay close attention to child for fever symptoms.
- Encourage mom to seek care immediately if child has signs of fever. Explain that fever might be a sign of malaria or other infection and that the clinic has medicines that can help.
- Encourage mom to place child under an insecticide treated bednet at night to protect child against mosquitos that might cause malaria.

Child Nutrition

- The child needs a diverse diet with protein, iron, vitamin A, vitamin C and other nutrients to grow and develop properly. Mom should give baby a variety of foods. Discuss adding at least one new food to increase diversity and add protein or nutrients.
- Discuss a new meal that mom can cook for child. Ask about child's preferences for food and make tailored suggestions.

Child Milestones

- Child might remember stories and songs and start to join in more as mom reads and sings. Demonstrate how mom can check for this.
- Child might get frustrated easily when things don't go their way. Explain to mom that this is natural.

Child Activity

- Teach mom a song about body parts that she can sing with the child. Over time, the child will learn the song and start to join mom in singing it.
- Help mom find a few objects in home that can be stacked. Demonstrate how mom can work with the child to stack objects. Have child say the name and color of the objects as they stack.
- Mom should develop habit of sitting with child and looking at a book. Encourage mom to sit with the child and a book every day.

Observe parent and child trying activity. If needed demonstrate again to help caregiver

Ask if the caregiver has any questions or concerns?
Arrange next visit date

Thank mother and family. Encourage mother to try implement the advice and activities.

Note any questions asked, action needed, referral required, or things to discuss with supervisor/team leader

End

Child Milestones

- Ngwana a ka šupa go bontšha se a se nyakago
- Ngwana a ka kgorometša dilo

- Child might point to show what they want
- Child might push things

- Xihlangi xi nga kombetela leswi xi swi lavaka
- Xihlangi xi nga susumeta swilo

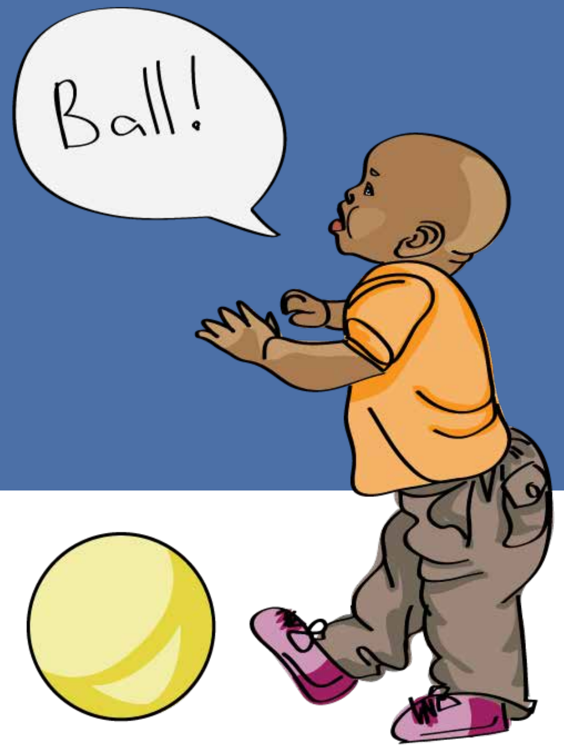

Child Health

- E ba le tsebo ka ga go gohlola le bothata bja go hema
- Hwetša kalafo ka potlako ge lesea le na le bothata bja go hema

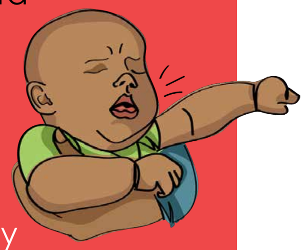

- Look for cough and difficulty breathing
- Seek care immediately if baby has difficulty breathing

- Kamba ku khohlola kumbe ku tik-eriwa ka ku hefemula
- Lava ku pfuniwa hi xihatla loko n'wana a tikeriwa ku hefemula

Child Nutrition

- Phepo e ama go gola ga ngwana
- Dino tša swikiri le malekere ga se tša lokela ngwana

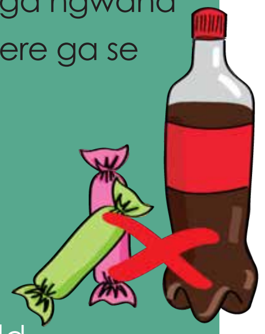

- Nutrition affects child development
- Sweet drinks and candy don't help child

- Tinyutiriyente ti khumba makulele ya n'wana
- Swinwiwa swo nyanganya na tikhendi a swi pfuni xihlangi

Child Activity

- Ikotlolle le ngwana ka maoto le go mmotšiša maina a dilo
- Eya le ngwana mmarakeng le go mmotšiša maina a dijo
- Balela ngwana letšatši le lengwe le le lengwe

- Take child for walk and ask object names
- Take child to market and ask food names
- Read to the child every day

- Teka xihlangi mi rhendzeleka kutani u xi vutisa mavito ya swakudya
- Famba na xihlangi emakete kutani u xi vutisa mavito ya swilo
- Hlayela n'wana masiku hinkwawo

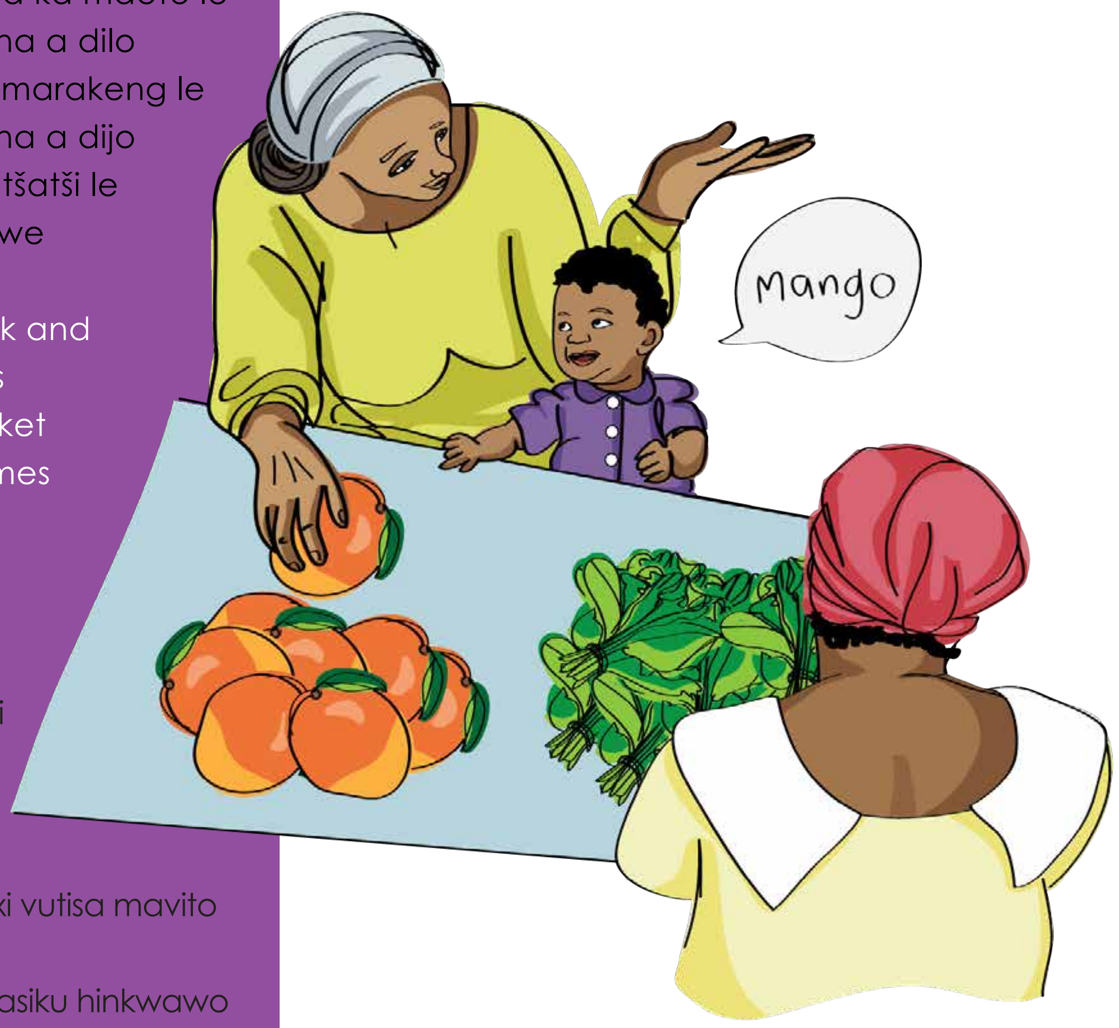

Start

Greet parent and explain purpose of visit.

Ask how parent and child are doing?

Ask caregiver if they have been trying any of the activities since last visit?

Ask if parent can show one of the activities?

Ask if mother/family has any difficulties/challenges in implementing the advice or activities?

Ask if mother/family observed any benefits in implementing the advice or activities?

Ask mother/family if she has any concerns (e.g. child is sick, appears slower to develop)?

Child Health

- Check baby for signs of cough and respiratory infection and demonstrate for mom how she can also check. Encourage mom to pay close attention to baby for respiratory symptoms.
- Encourage mom to seek care immediately if baby has danger signs of respiratory infection, including fast breathing and wheezing sounds. Explain that symptoms may be a sign of pneumonia or other infection and that the clinic has medicines that can help.

Child Nutrition

- Child's nutrition affects physical growth and development. It is important that child has enough protein and micronutrients in their diet. If they do not, their growth may be slowed.
- While sweet drinks and candy might sooth the child for a short time, they do not at all help the child develop over the long term. Discourage mom from giving child sweets and make it clear that sweets should not be a substitute for the nutritious food the child needs.

Child Milestones

- Child might point at the things that they want. Demonstrate how mom can check for this.
- Child might push things around the house. Demonstrate how mom can check for this.

Child Activity

- Encourage mom to go for a walk around the village or town and point at things they see and ask child to say the name of the food.
- Encourage mom to bring child when she goes to the market and to walk around and point at foods and ask child to say the name of the food.
- Mom should develop habit of sitting with child and looking at a book. Encourage mom to sit with the child and a book every day.

Observe parent and child trying activity. If needed demonstrate again to help caregiver

Ask if the caregiver has any questions or concerns?
Arrange next visit date

Thank mother and family. Encourage mother to try implement the advice and activities.

Note any questions asked, action needed, referral required, or things to discuss with supervisor/team leader

End

S
E
T

Child Activity

- E fa ngwana ditaelo tse di rarakenego
- Thala o be o rute ngwana dibopego
- Balelela ngwana tsatsi ka letsatsi

- Give the child complicated instructions
- Draw and teach child shapes
- Read to the child every day

- Nyika n'wana swileriso swo tika
- Tshala ni ku dyondzisa n'wana swivumbeko
- Hlayela n'wana masiku hinkwawo

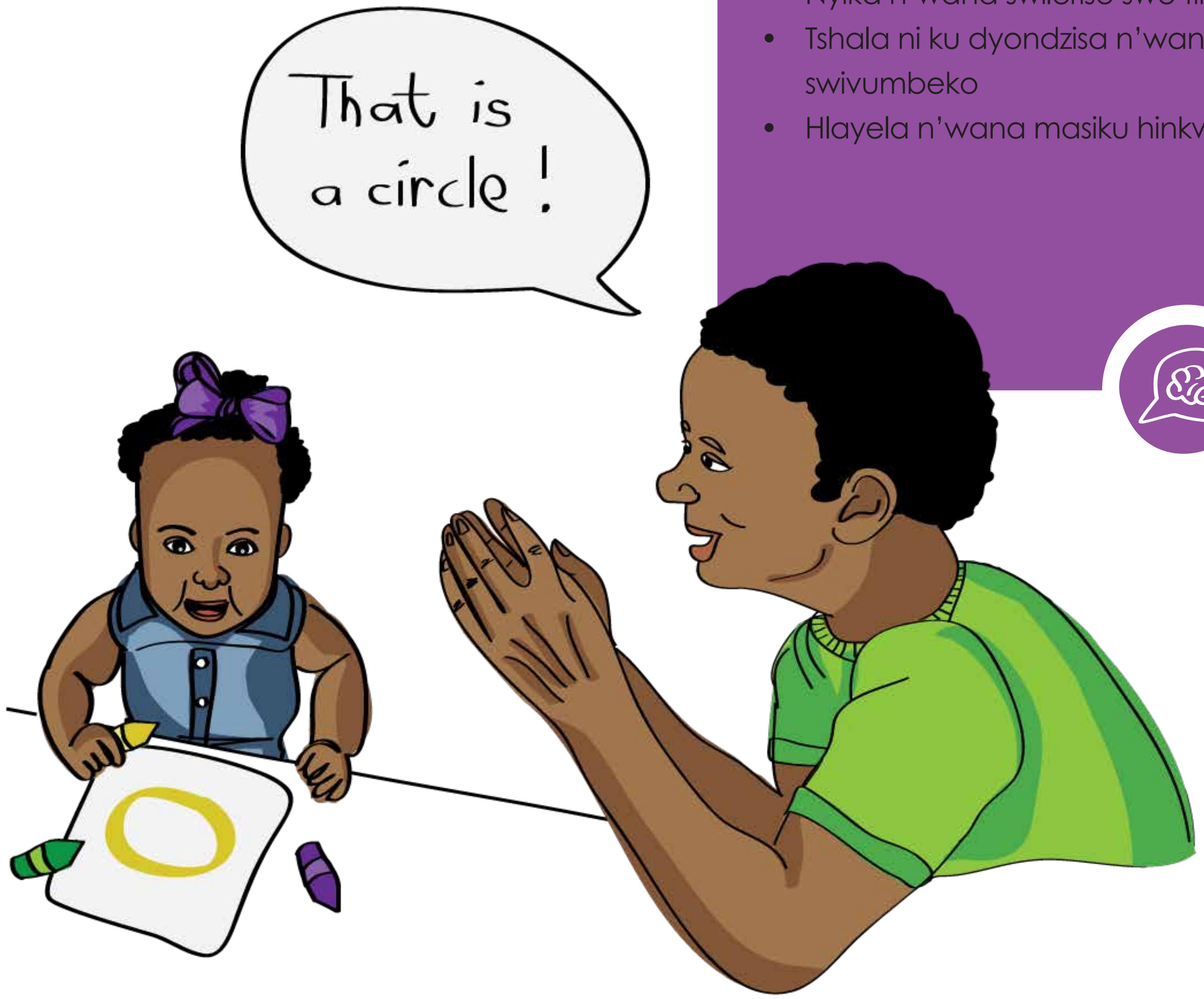

Child Milestones

- Ngwana a ka sepela gabotse ka noši
- Ngwana a ka ja ka noši ka diatla tša gagwe

- Child might walk well on own
- Child might eat with hands on own

- Xihlangi xi nga kota ku tifambela kahle
- Xihlangi xi nga kota ku tidyisa hi swandla

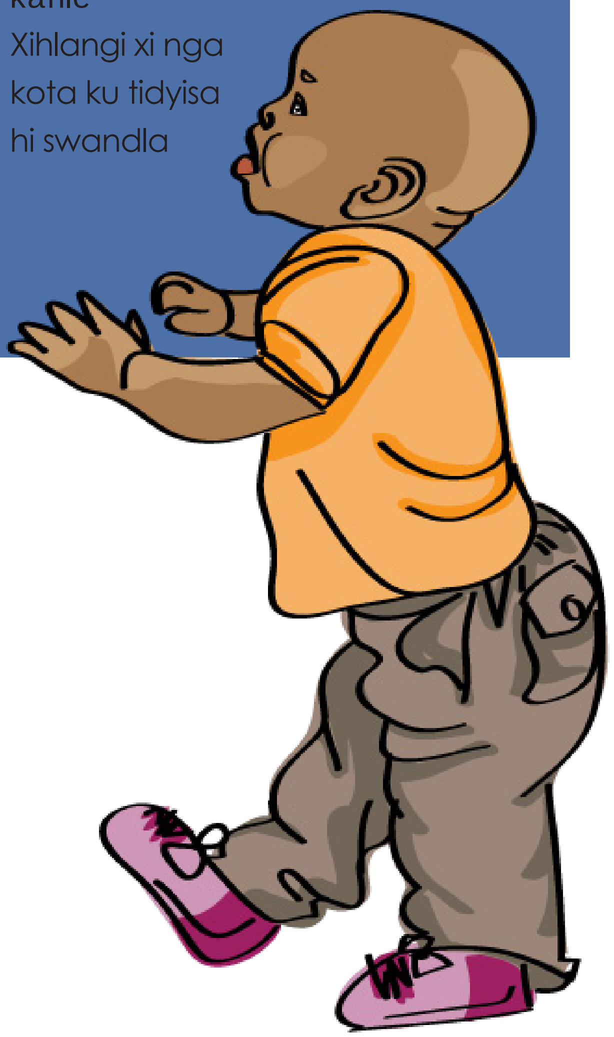

Child Health

- Ditšhwaana tša thibela malwetši di šireletša ngwana
- Maphelo a ama kgodišo ya ngwana

- Vaccines protect child
- Health affects child's development

- Misawutiso yi sirhelela xihlangi
- Rihanyu ri khumba makulele ya n'wana

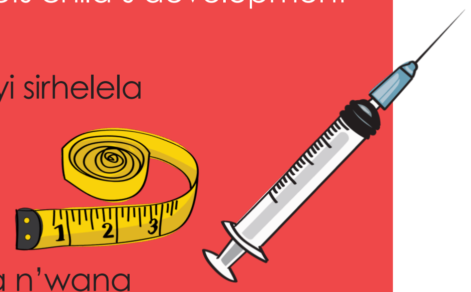

Child Nutrition

- Phepo e ama go gola ga ngwana
- Ngwana o hloka protheine
- Ngwana o swanetše go ja dinawa le mae

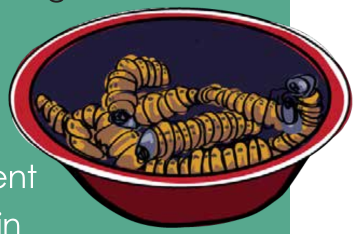

- Nutrition affects child's development
- Child needs protein
- Child should eat beans and eggs

- Tinyutiriyente ti khumba makulele ya n'wana
- N'wana u lava phurotheyini
- N'wana u fanele ku dya tinyawa na matandza

Start

Greet parent and explain purpose of visit.

Ask how parent and child are doing?

Ask caregiver if they have been trying any of the activities since last visit?

Ask if parent can show one of the activities?

Ask if mother/family has any difficulties/challenges in implementing the advice or activities?

Ask if mother/family observed any benefits in implementing the advice or activities?

Ask mother/family if she has any concerns (e.g. child is sick, appears slower to develop)?

Child Health

- At 18 months old, children should receive measles2 and DTaP-IPV/Hib4. Confirm that the child has received these vaccines. If not yet received, encourage mom to bring child to the clinic for these vaccines immediately.
- Child's health affects physical growth and development. It is important that mom seeks care for child when child has symptoms of illness. Even if symptoms do not seem too serious, untreated infections may slow the child's development over time. Mom should take child to the clinic for a general checkup every three months or so. Ask mom if child has been checked recently, and if not encourage mom to take child to clinic for a checkup.

Child Nutrition

- Child's nutrition affects physical growth and development. It is important that child has enough protein and micronutrients in their diet. If they do not, their growth may be slowed.
- The child needs to consume a good amount of protein through their diet in order for their body to develop properly. Protein will help them grow taller and stronger.
- A few good sources of protein are beans and eggs. Encourage mom to incorporate beans or eggs into the child's diet as much as possible.

Child Milestones

- Child might walk well on own. Demonstrate how mom can check for this.
- Child might eat with hands on own. Demonstrate how mom can check for this.

Child Activity

- Encourage mom to give child instructions that include at least two commands, for example 'go to the kitchen and get salt'.
- Show mom how to sit with child and drawn and teach basic shapes like circle, square, and triangle.
- Mom should develop habit of sitting with child and looking at a book. Encourage mom to sit with the child and a book every day.

Observe parent and child trying activity. If needed demonstrate again to help caregiver

Ask if the caregiver has any questions or concerns?
Arrange next visit date

Thank mother and family. Encourage mother to try implement the advice and activities.

Note any questions asked, action needed, referral required, or things to discuss with supervisor/team leader

End

Child Milestones

- Ngwana a ka robala nako ye kopana
- Ngwana a ka leka go tshela mellwane le go se obamele melao

- Child might sleep less
- Child might test limits and break rules

- Swi nga endleka xihlangi xi nga ha etleli ngopfu
- Xihlangi xi nga endla leswi u xi byelaka leswaku xi nga swi endli na ku tlula milawu

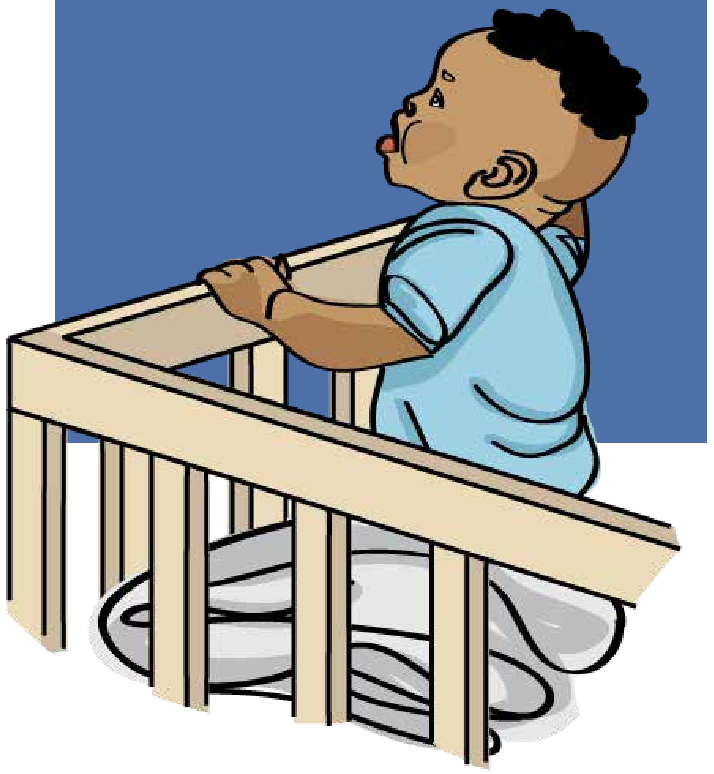

Child Health

- Ngwana o swanetše go hlapa meno ka poratšhe letšatši le lengwe le le lengwe
- E ba le tsebo ka dika
- Hwetša kalafo ka potlako ya bolwetši

- Child should brush teeth every day
- Look for symptoms
- Seek care immediately for illness

- Xihlangi xi fanele ku buracha meno masiku hinkwawo
- Languta swikombo
- Lava vutshunguri hi xihatla loko ku ri na mavabyi

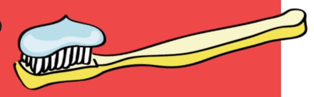

Child Nutrition

- Ngwana o hloka bitamine A
- Ngwana o swanetše go ja merogo ye metala ya mahlare

- Child needs vitamin A
- Child should eat leafy green vegetables

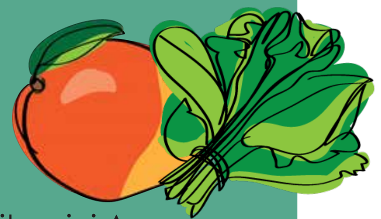

- N'wana u lava vhitamini A
- N'wana u fanele ku dya matsavu yo tsakama

Child Activity

- Botsisha ngwana maina a dilo ka mo ntlong
- Ngwala o rute ngwana dithlaka
- Balela ngwana tsatsi ka tsatsi

- Ask child name of objects in house
- Write and teach child letters
- Read to the child every day

- Vutisa n'wana mavito ya swilo a ndlwini
- Tsala ni dyondzisa n'wana marito
- Hlayela n'wana masiku hinkwawo

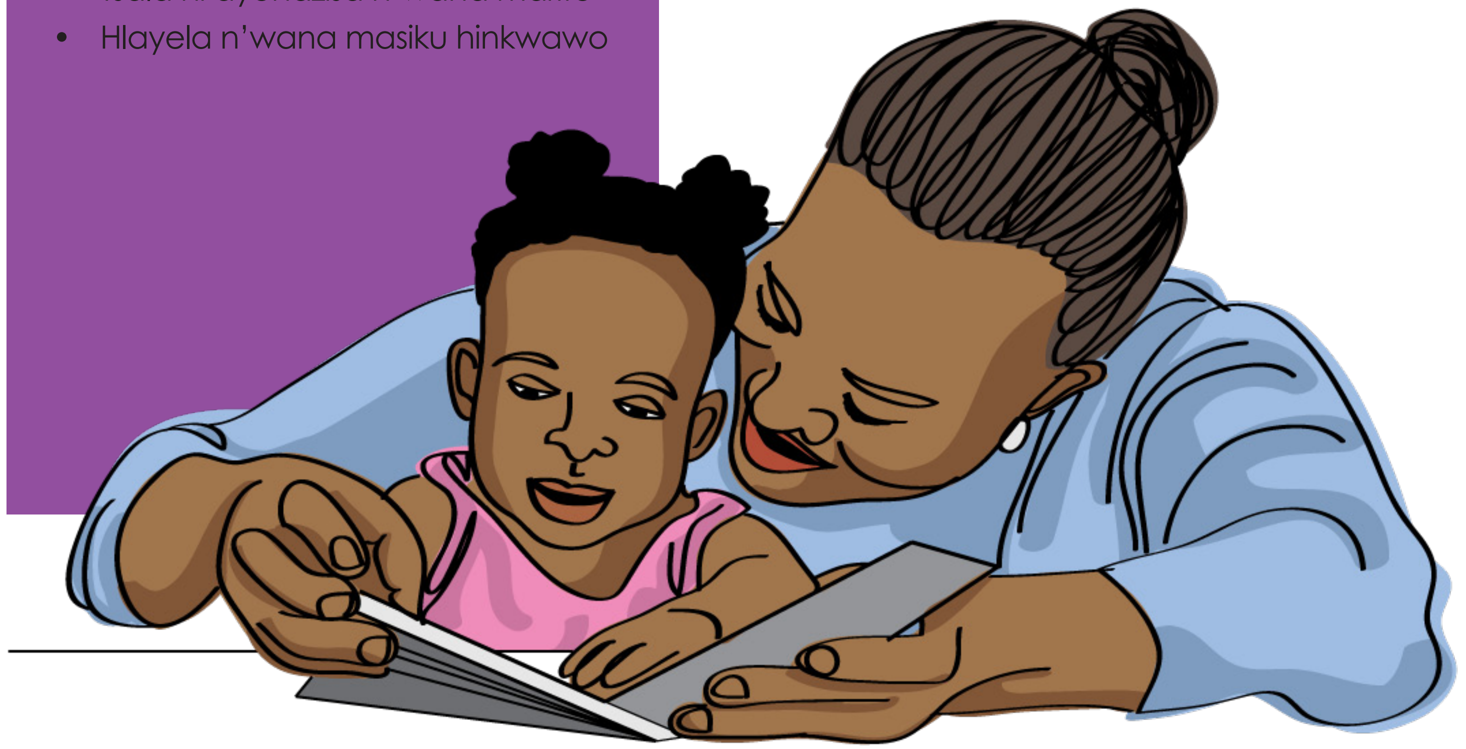

S
E
T

Start

Greet parent and explain purpose of visit.

Ask how parent and child are doing?

Ask caregiver if they have been trying any of the activities since last visit?

Ask if parent can show one of the activities?

Ask if mother/family has any difficulties/challenges in implementing the advice or activities?

Ask if mother/family observed any benefits in implementing the advice or activities?

Ask mother/family if she has any concerns (e.g. child is sick, appears slower to develop)?

Child Health

- Child should start to brush teeth every day if they haven't already. Teach child to brush teeth and explain that it is important to brush every day.
- Check child for symptoms and demonstrate for mom how she can also check. Encourage mom to pay close attention to child for symptoms.
- Encourage mom to seek care immediately if child has symptoms. Explain that even if symptoms seem non-severe, they may slow the child's development over time. Explain that the clinic has medicines that can help.

Child Nutrition

- The child needs to consume a good amount of vitamin A through their diet in order for their body to develop correctly. In particular, vitamin A will help them fight infections.
- One good source of vitamin A is leafy green vegetables. Encourage mom to incorporate leafy green vegetables into the child's diet as much as possible.

Child Milestones

- Child might sleep less and have a harder time taking naps and sleeping at night. Explain to mom that this is okay.
- Child might test the limits that mom sets and intentionally break rules. Explain to mom that this is okay and the child is just learning the boundaries of normal behaviour.

Child Activity

- Encourage mom to go around the house with the child and point at things they see and ask child to say the name of the object.
- Show mom how to sit with child and write and teach a 2-3 letters (e.g., A, B, C).
- Mom should develop habit of sitting with child and looking at a book. Encourage mom to sit with the child and a book every day.

Observe parent and child trying activity. If needed demonstrate again to help caregiver

Ask if the caregiver has any questions or concerns?
Arrange next visit date

Thank mother and family. Encourage mother to try implement the advice and activities.

Note any questions asked, action needed, referral required, or things to discuss with supervisor/team leader

End

S
E
T

Child Activity

- Botsisha ngwana mebala ya dilo ka mo ntlong
- Bala dilo le ngwana
- Balela ngwana tsatsi ka tsatsi

- Ask child color of objects in house
- Count objects with the child
- Read to the child every day

- Vutisa n'wana mihlovo ya swilo a ndlwini
- Hlayela swilo na n'wana
- Hlayela nwana masiku hinkwawo

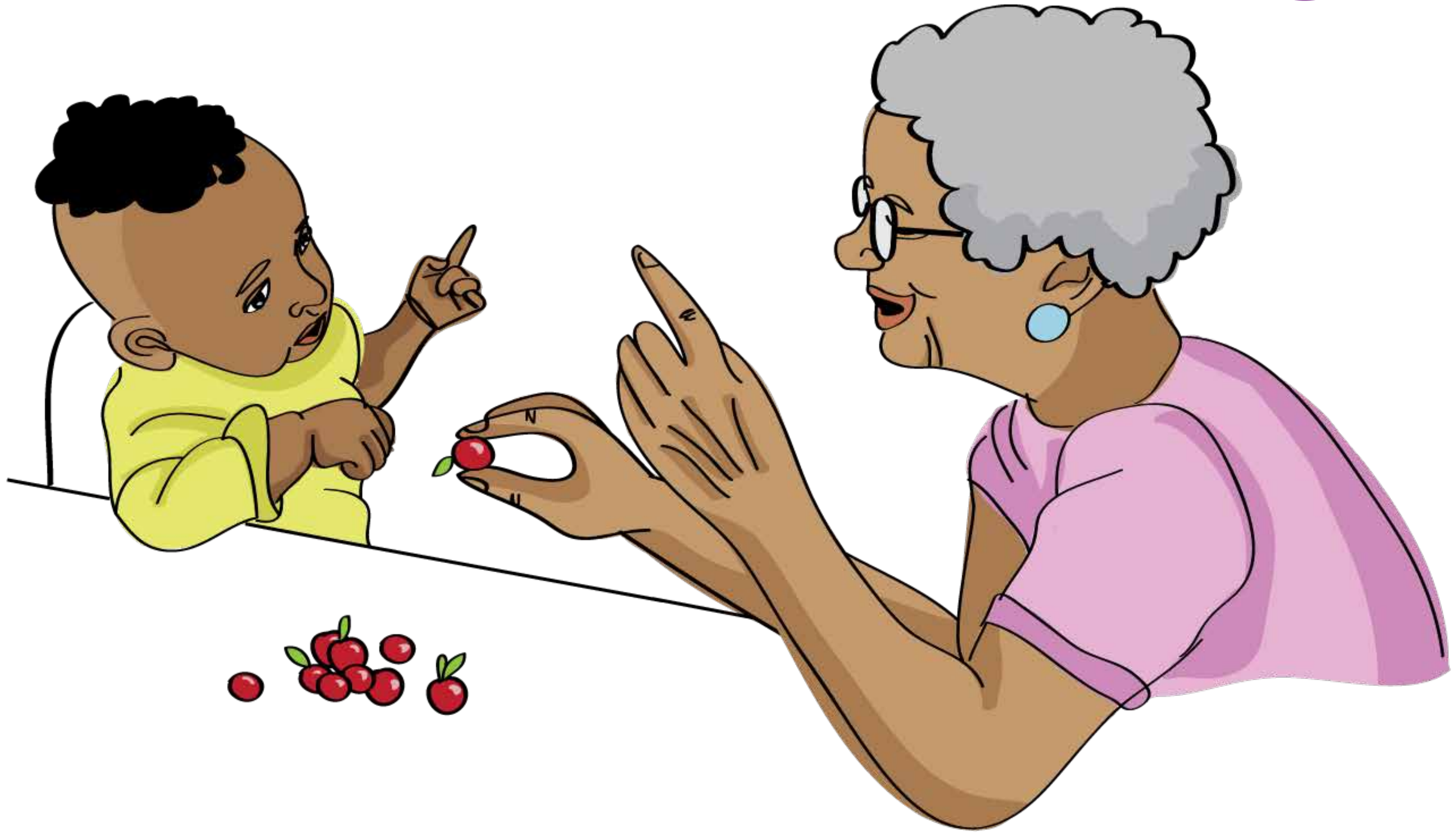

Child Milestones

- Ngwana a ka nyaka go ikema ka noši
- Ngwana a ka namela ditepisi le go tshela

- Child might become more independent
- Child might walk stairs and jump

- Xihlangi xi nga ka xi nga ha lavi ku pfuniwa eka swotala
- Xihlangi xi nga khandziya switepisi na ku tlula

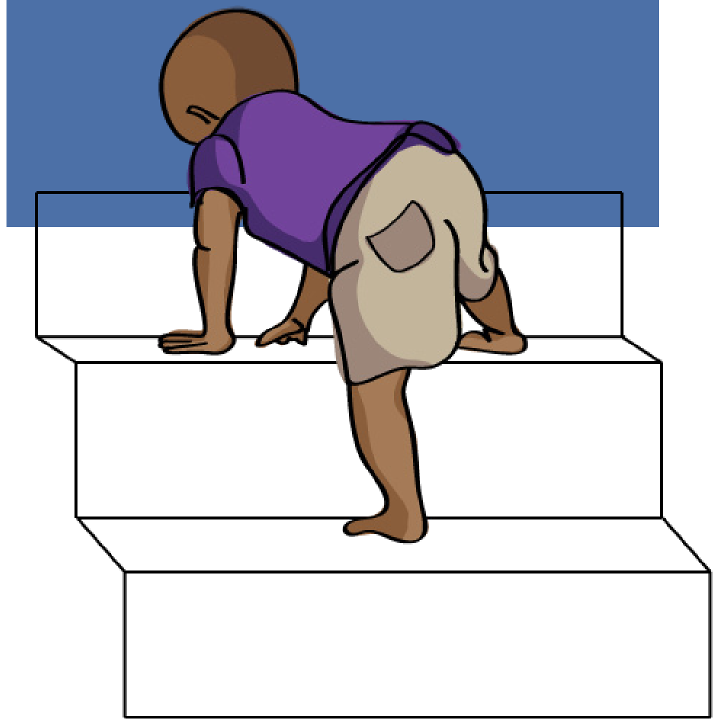

Child Health

- E ba le tsebo ka ga letšhollo
- Šomiša motswako wa go thibela letšhollo (ORS)

- Look for diarrhea
- Use oral rehydration salt (ORS) solution

- Languta nchuluko
- Tirhisa diripi (oral rehydration salt [ORS])

Child Nutrition

- Ngwana o hloka ayone
- Ngwana o swanetše go ja dinawa

- Child needs iron
- Child should eat beans

- N'wana u lava ayoni
- N'wana u fanele ku dya tinyawa

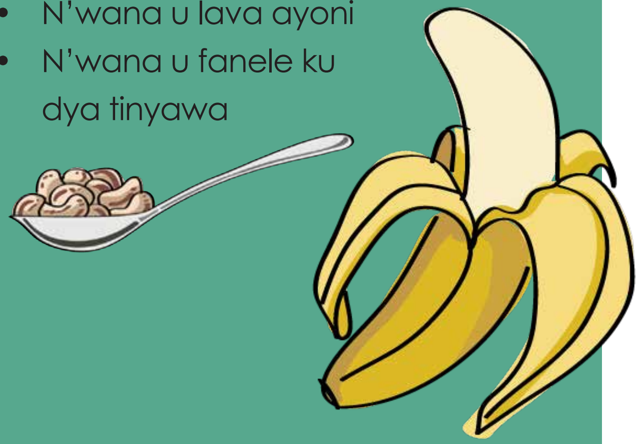

MONTH 20

Start

Greet parent and explain purpose of visit.

Ask how parent and child are doing?

Ask caregiver if they have been trying any of the activities since last visit?

Ask if parent can show one of the activities?

Ask if mother/family has any difficulties/challenges in implementing the advice or activities?

Ask if mother/family observed any benefits in implementing the advice or activities?

Ask mother/family if she has any concerns (e.g. child is sick, appears slower to develop)?

Child Health

- As child starts to eat more and more new foods, the risks for diarrhea may increase. Encourage mom to pay close attention to whether child has diarrhea, particularly if baby is showing signs of dehydration or malnutrition due to diarrhea.
- Encourage mom to give child ORS when baby has diarrhea to avoid dehydration. Demonstrate for mom how to make ORS or discuss where she can buy ORS packets nearby.

Child Nutrition

- The child needs to consume a good amount of iron through their diet in order for their body to develop correctly. In particular, iron will help their brain develop.
- One good source of iron is beans. Encourage mom to incorporate beans into the child's diet as much as possible.

Child Milestones

- Child might start to do more activities by themselves and be more independent. Explain to mom that this is okay
- Child might learn to walk up and down stairs and to jump. Demonstrate how mom can check for this.

Child Activity

- Encourage mom to go around the house with the child and point at things they see and ask child to say the color of the object.
- Help mom find a set of objects at least 10 in number (e.g., beans, small stones). Demonstrate how mom can count the objects with child.
- Mom should develop habit of sitting with child and looking at a book. Encourage mom to sit with the child and a book every day.

Observe parent and child trying activity. If needed demonstrate again to help caregiver

Ask if the caregiver has any questions or concerns?
Arrange next visit date

Thank mother and family. Encourage mother to try implement the advice and activities.

Note any questions asked, action needed, referral required, or things to discuss with supervisor/team leader

End

Child Milestones

- Ngwana a ka namela le go folo-ga se sengwe le se sengwe
- Ngwana a ka raloka thwii le bana ba bangwe

- Child might climb up and down everything
- Child might interact directly with other children

- Xihlangi xi nga khandziya no chika eka nchumu wu'wana na wun'wana
- Xihlangi xi nga tlanga na vana van'wana

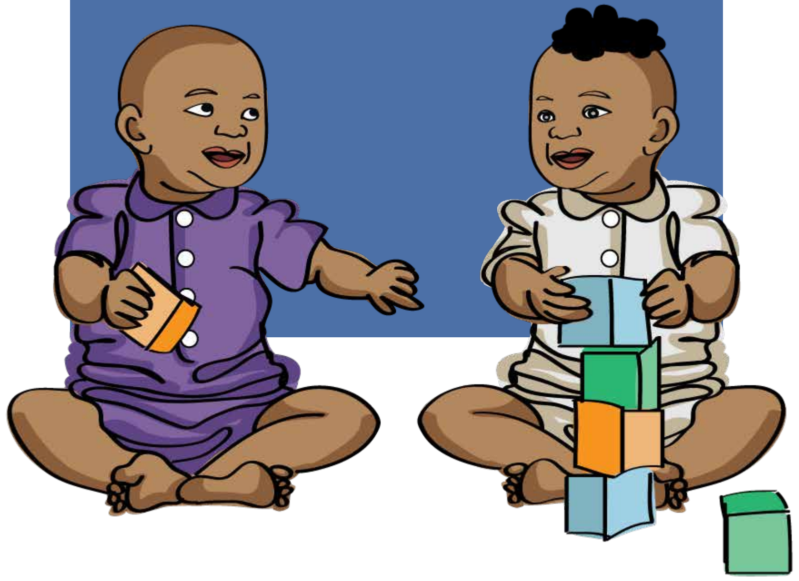

Child Health

- E ba le tsebo ka ga letadi
- Hwetša kalafo ka potlako ge ngwana a na le letadi
- Ngwana o swanetše go khupetšwa ka nete ge a robetše

- Look for fever
- Seek care immediately if child has fever
- Child should sleep under net

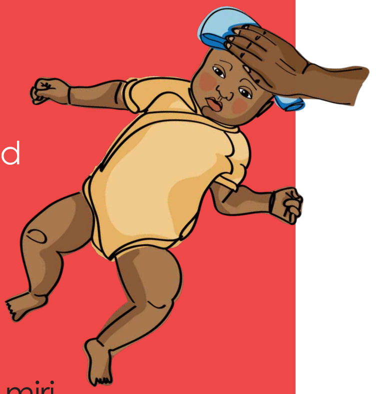

- Kamba ku hisa ka miri
- Lava vutshunguri hi xihatla loko n'wana a hisa miri
- N'wana u fanele ku etlela endzeni ka nete

Child Nutrition

- Ngwana o hloka bitamine C
- Ngwana o swanetše go ja dienywa

- Child needs vitamin C
- Child should eat fruits

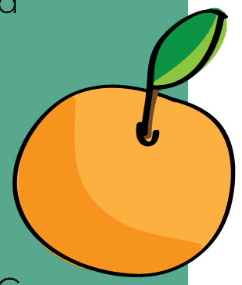

- N'wana u lava vhitamini C
- N'wana u fanele ku dya mihandzu

Child Activity

- Isha ngwana tulo ye e bolokegile-go gore a namele a be a hlotletse
- Raloka medumo ya diphoofolo le ngwana
- Balela ngwana tsatsi ka tsatsi

- Take child to safe place to climb and explore
- Play 'animal sounds' with the child
- Read to the child every day

- Teka n'wana ka ndhawu yo hlay-iseka ku ya khandiya khandiya
- Tlanga mimpfumawulo ya swiharhi na n'wana
- Hlayela n'wana masiku hinkwawo

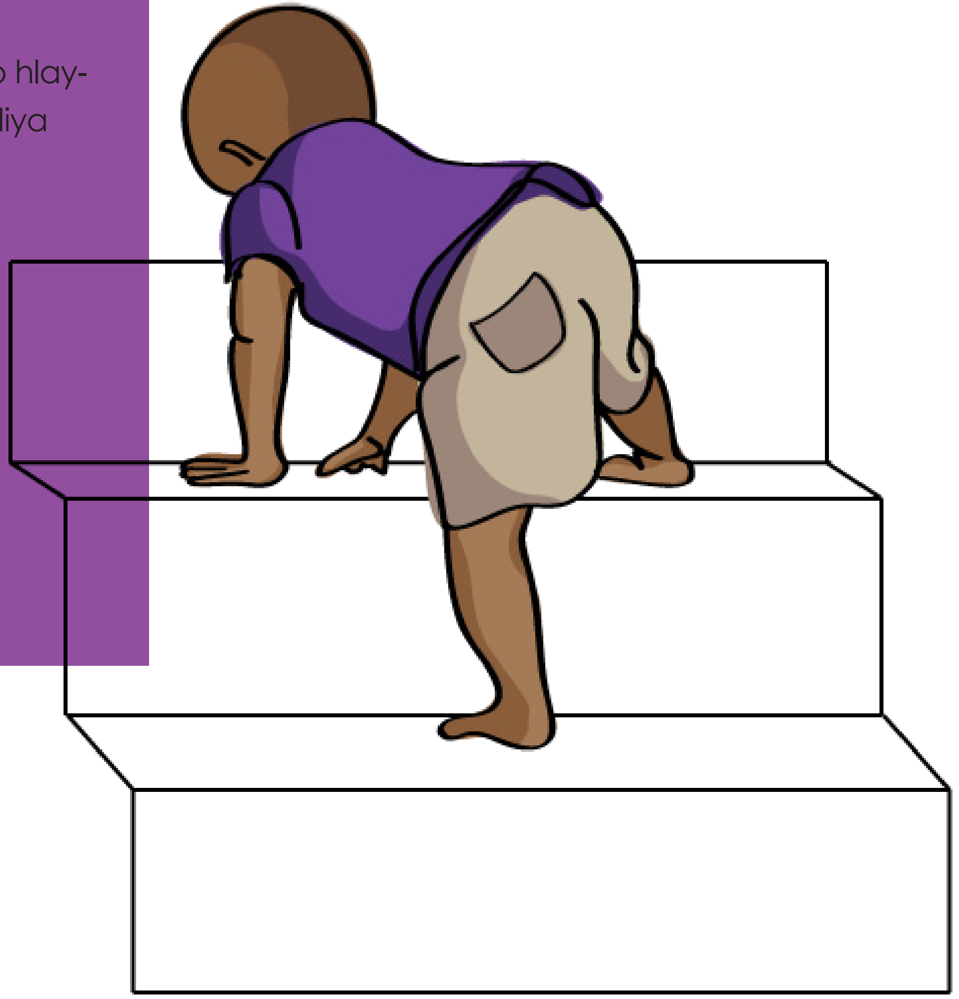

Start

Greet parent and explain purpose of visit.

Ask how parent and child are doing?

Ask caregiver if they have been trying any of the activities since last visit?

Ask if parent can show one of the activities?

Ask if mother/family has any difficulties/challenges in implementing the advice or activities?

Ask if mother/family observed any benefits in implementing the advice or activities?

Ask mother/family if she has any concerns (e.g. child is sick, appears slower to develop)?

Child Health

- Check child for signs of fever and demonstrate for mom how she can also check. Encourage mom to pay close attention to child for fever symptoms.
- Encourage mom to seek care immediately if child has signs of fever. Explain that fever might be a sign of malaria or other infection and that the clinic has medicines that can help.
- Encourage mom to place child under an insecticide treated bednet at night to protect child against mosquitos that might cause malaria.

Child Nutrition

- The child needs to consume a good amount of vitamin C through their diet in order for their body to develop correctly. In particular, vitamin C will help them fight infections.
- One good source of vitamin C is fruit. Encourage mom to incorporate fruit into the child's diet as much as possible.

Child Milestones

- Child might climb on everything, including furniture in the house. Check with mom that the home is safe for the child.
- Child might start to interact more with other children in the village. Child might fight with other children sometimes. Explain to mom that this is natural as the child learns how to interact.

Child Activity

- Talk with mom about a safe place that she can take the child where the child can climb and explore. Encourage mom to take child to this safe place often.
- Show mom how to play with the child by making animal sounds and asking the child to say the name of the animal for each sound.
- Mom should develop habit of sitting with child and looking at a book. Encourage mom to sit with the child and a book every day.

Observe parent and child trying activity. If needed demonstrate again to help caregiver

Ask if the caregiver has any questions or concerns?
Arrange next visit date

Thank mother and family. Encourage mother to try implement the advice and activities.

Note any questions asked, action needed, referral required, or things to discuss with supervisor/team leader

End

S
E
T

Child Activity

- E re ngwana a age dilo a bolela maina le mebala ya tsona
- E re ngwana a thale le gofa maila a dibopego
- Balela ngwana tsatsi ka tsatsi

- Have child stack objects and say name/color
- Have child draw and name shapes
- Read to the child every day

- Endla n'wana a aka swilo ni ku vula mavito/mihlovo
- Endla n'wana a tshala ni ku vula mavito ya swivumbeko
- Hlayela n'wana masiku hinkwawo

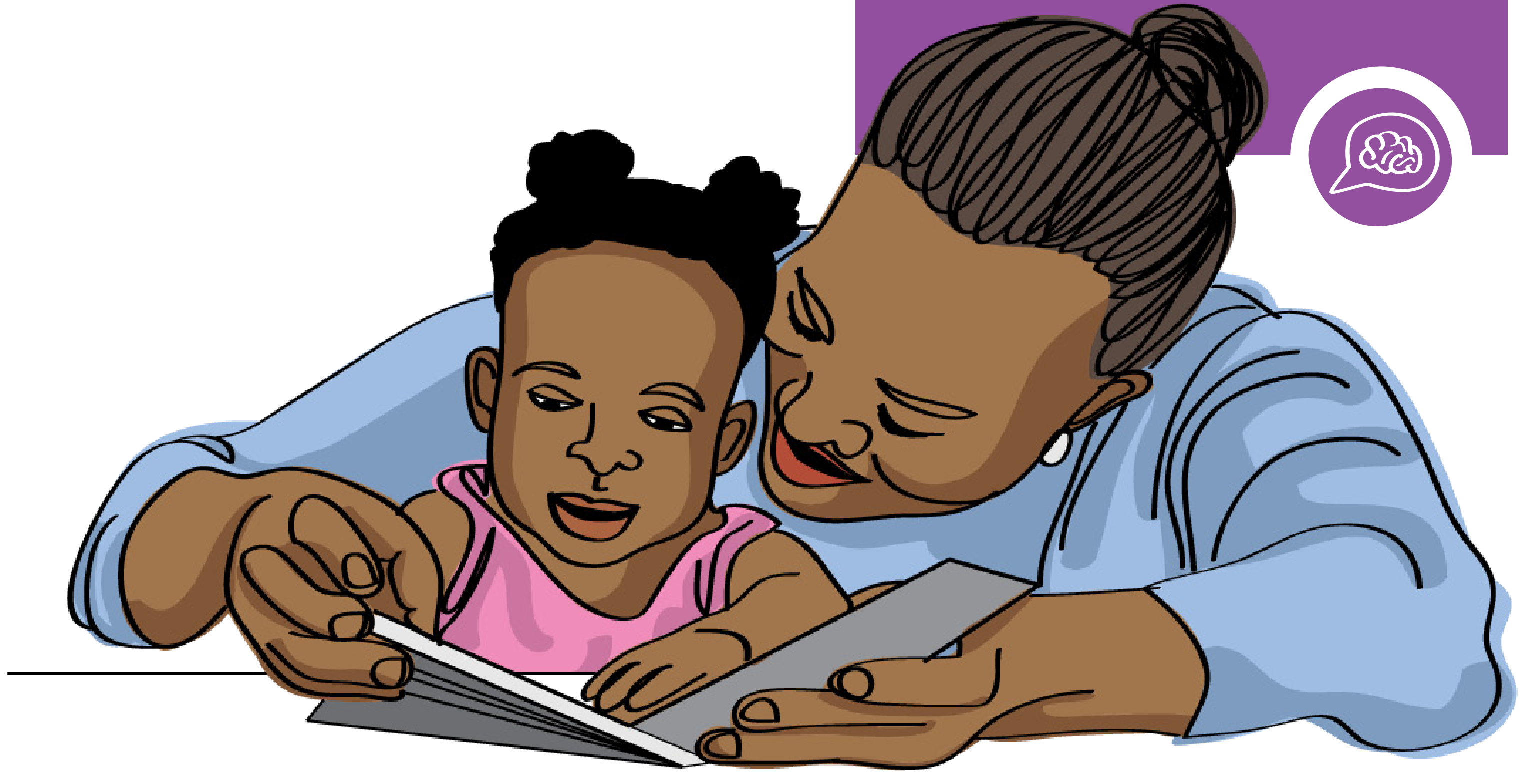

Child Milestones

- Ngwana a ka bopa mafoko a bonolo
- Ngwana a ka ipshina ka go bina

- Child might make simple sentences
- Child might enjoy dancing

- Xihlangi xi nga vumba swivulwa swo olova
- Xihlangi xi nga tsakisiwa hi ku cina

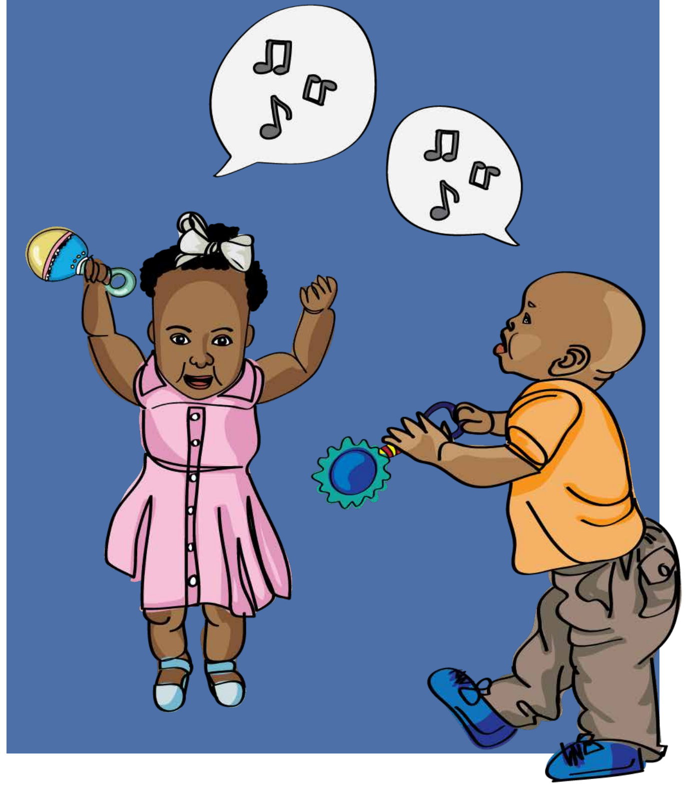

Child Health

- E ba le tsebo ka ga go gohlola le bothata bja go hema
- Hwetša kalafo ka potlako ge lesea le na le bothata bja go hema

- Look for cough and difficulty breathing
- Seek care immediately if baby has difficulty breathing

- Kamba ku khohlola kumbe ku tikeriwa ka ku hefemula
- Lava ku pfuniwa hi xihatla loko n'wana a tikeriwa ku hefemula

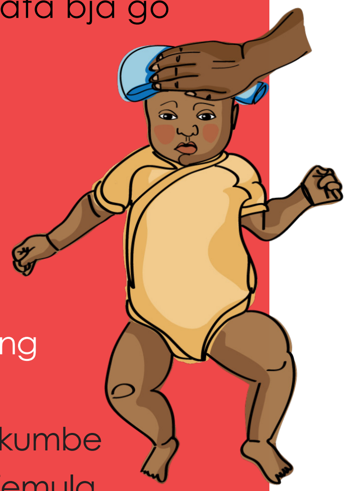

Child Nutrition

- Ngwana o hloka dijo tše ntši
- Leka motswako o moswa

- Child needs many foods
- Try a new recipe

- N'wana u lava swakudya swo tala
- Ringeta rhesipi yintswa

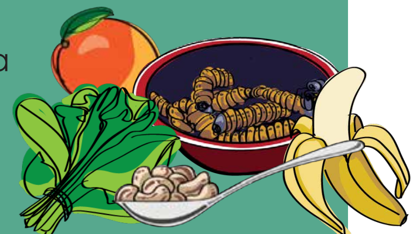

Start

Greet parent and explain purpose of visit.

Ask how parent and child are doing?

Ask caregiver if they have been trying any of the activities since last visit?

Ask if parent can show one of the activities?

Ask if mother/family has any difficulties/challenges in implementing the advice or activities?

Ask if mother/family observed any benefits in implementing the advice or activities?

Ask mother/family if she has any concerns (e.g. child is sick, appears slower to develop)?

Child Health

- Check baby for signs of cough and respiratory infection and demonstrate for mom how she can also check. Encourage mom to pay close attention to baby for respiratory symptoms.
- Encourage mom to seek care immediately if baby has danger signs of respiratory infection, including fast breathing and wheezing sounds. Explain that symptoms may be a sign of pneumonia or other infection and that the clinic has medicines that can help.

Child Nutrition

- The child needs a diverse diet with protein, iron, vitamin A, vitamin C and other nutrients to grow and develop properly. Mom should give baby a variety of foods. Discuss adding at least one new food to increase diversity and add protein or nutrients.
- Discuss a new meal that mom can cook for child. Ask about child's preferences for food and make tailored suggestions.

Child Milestones

- Child might start to make simple sentences. Demonstrate for mom how to check this.
- Child might begin to enjoy dancing with family or with other children. Demonstrate for mom how to check this.

Child Activity

- Help mom find a few objects in home that can be stacked. Demonstrate how mom can work with the child to stack objects. Have child say the name and color of the objects as they stack.
- Encourage mom to ask child to drawn and name basic shapes (e.g., circle, square, triangle)
- Mom should develop habit of sitting with child and looking at a book. Encourage mom to sit with the child and a book every day.

Observe parent and child trying activity. If needed demonstrate again to help caregiver

Ask if the caregiver has any questions or concerns?
Arrange next visit date

Thank mother and family. Encourage mother to try implement the advice and activities.

Note any questions asked, action needed, referral required, or things to discuss with supervisor/team leader

End

Child Milestones

- Ngwana a ka ithuta go bolela dilo tše mpsha letšatši le lengwe le le lengwe
- Ngwana a ka opela dikoša le ba bangwe

- Child might learn to say new things every day
- Child might sing along to songs

- Xihlangi xi nga dyondza ku vula swilo swintshwa masiku hinkwawo
- Xihlangi xi nga yimbelela na un'wana

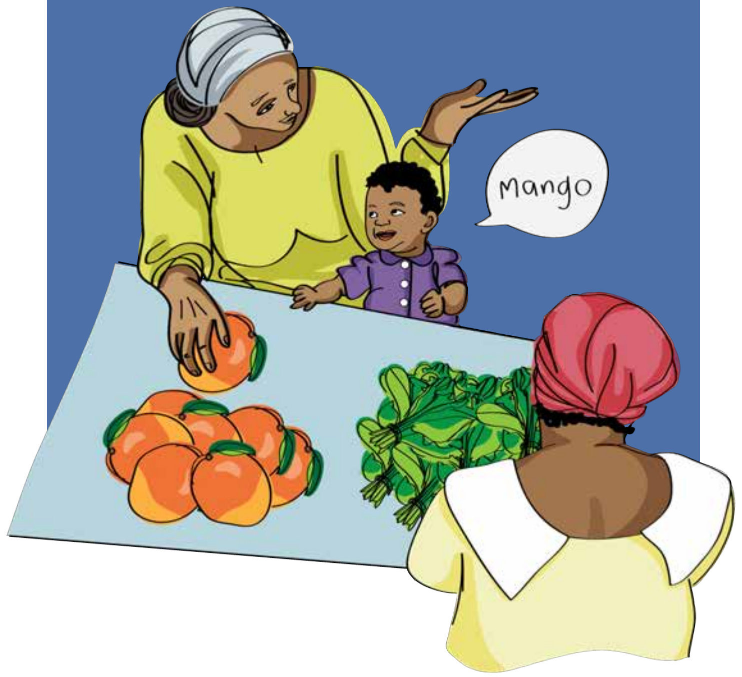

Child Nutrition

- Phepo e ama go gola ga ngwana

- Nutrition affects child's development

- Tinyutiriyente ti khumba makulele ya n'wana

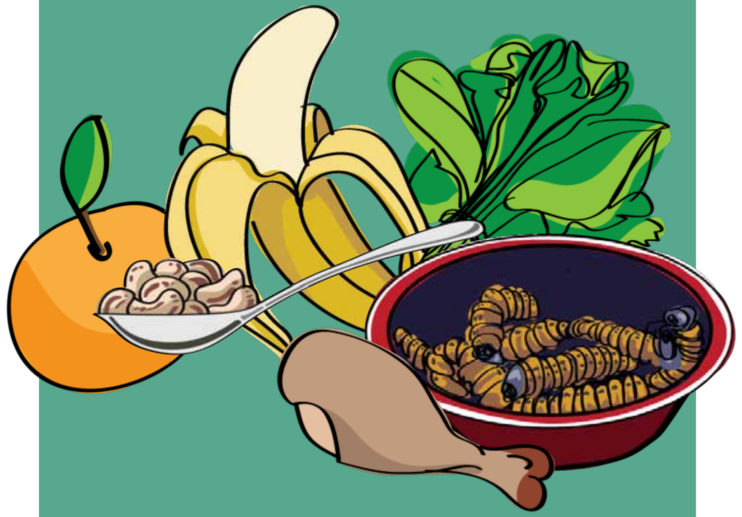

Child Health

- Maphelo a ama kgodišo ya ngwana

- Health affects child's development

- Rihanyu ri khumba makulele ya n'wana

Child Activity

- Opela kosha le ngwana
- Botja ngwana a go botje maina a dihlaka
- Balela ngwana tsatsi ka tsatsi

- Sing songs with child
- Have child name letters
- Read to the child every day

- Yimbelela tinsimu na n'wana
- Endla n'wana a thya marito
- Hlayela n'wana masiku hinkwawo

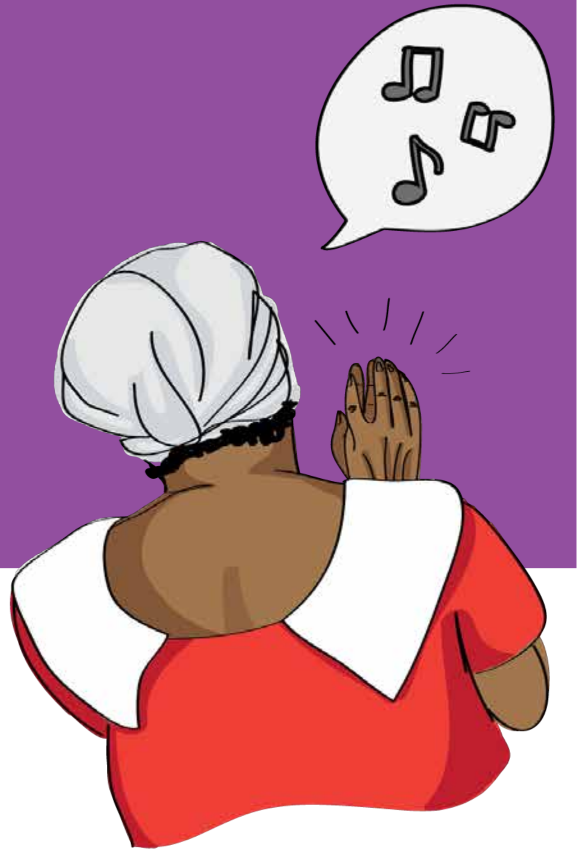

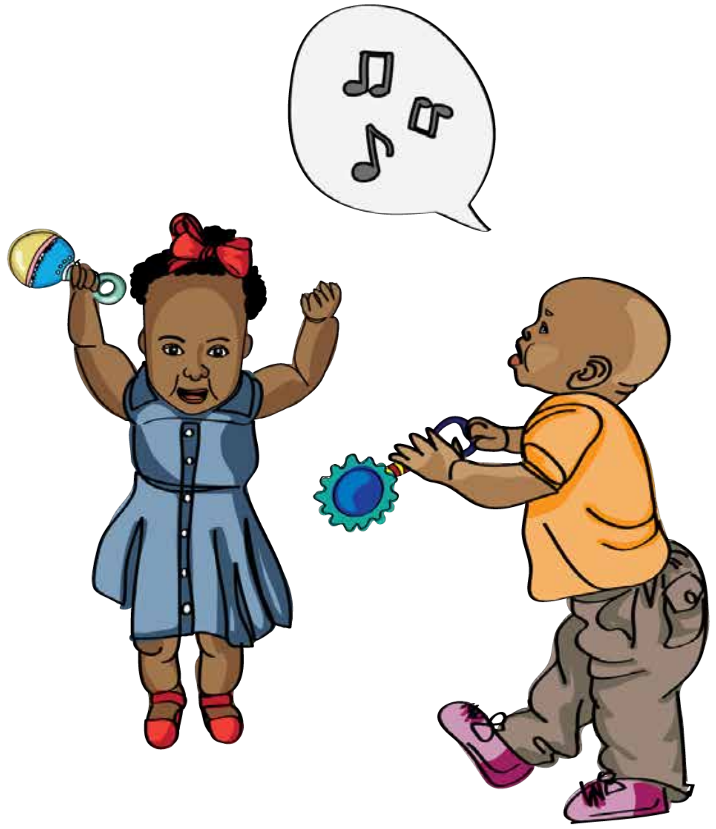

Start

Greet parent and explain purpose of visit.

Ask how parent and child are doing?

Ask caregiver if they have been trying any of the activities since last visit?

Ask if parent can show one of the activities?

Ask if mother/family has any difficulties/challenges in implementing the advice or activities?

Ask if mother/family observed any benefits in implementing the advice or activities?

Ask mother/family if she has any concerns (e.g. child is sick, appears slower to develop)?

Child Health

- Child's health affects physical growth and development. It is important that mom seeks care for child when child has symptoms of illness. Even if symptoms do not seem too serious, untreated infections may slow the child's development over time. Mom should take child to the clinic for a general checkup every three months or so. Ask mom if child has been checked recently, and if not encourage mom to take child to clinic for a checkup.

Child Nutrition

- Child's nutrition affects physical growth and development. It is important that child has enough protein and micronutrients in their diet. If they do not, their growth may be slowed.

Child Milestones

- The child is becoming increasingly talkative now. She might start to learn new words every day. Demonstrate how mom can check for this.
- Child might remember the words to songs and sing along when mom sings. Demonstrate how mom can check this.

Child Activity

- Encourage mom to sing songs with child each day and have child sing along.
- Encourage mom to help child learn letters by writing a few (e.g., A, B, C) and asking child to name them.
- Mom should develop habit of sitting with child and looking at a book. Encourage mom to sit with the child and a book every day.

Observe parent and child trying activity. If needed demonstrate again to help caregiver

Ask if the caregiver has any questions or concerns?
Arrange next visit date

Thank mother and family. Encourage mother to try implement the advice and activities.

Note any questions asked, action needed, referral required, or things to discuss with supervisor/team leader

End
