# Supplementary material for: Evaluation of a community health worker home visit intervention to improve child development in South Africa: A cluster-randomized controlled trial
Source: PLoS Med. 2023 Apr 14;20(4):e1004222. doi: 10.1371/journal.pmed.1004222 (PMC10146459; doi:10.1371/journal.pmed.1004222)
Supplement: Attachment — Submitted filename: Reviewer notes.docx [file pmed.1004222.s009.docx]

Reviewer notes -	Dr. Karl le Roux

For the editors – I am a medical doctor who is a practicing clinician and researcher in a deeply rural part of the Eastern Cape of South Africa, who has helped implement and investigate several home visiting Community Health Worker (CHW) programmes in our rural area. My experience and expertise is therefore in the practical aspects of implementing CHW programmes and also investigating the impact of these on children and mothers of home visiting through, amongst others, cluster randomized trials. I am interested in real-world lessons that have been learnt and can help clinicians working at the coal-face of healthcare delivery.

Though I understand basic statistics, I do not claim major expertise in this regard and hope that you and your other reviewers will be able to interrogate the statistical methods more thoroughly.

I think that this paper and approach of using neural markers is to measure the impact of an intervention is interesting and innovative, though there are several aspects of the study that need clarification and revision before I believe it ready to be published. There are a few significant clarifications that need to be made – for example, it isn’t at all clear what the differences in actual intervention was between the two groups. However, most of the questions I have asked in my review below should be fairly straightforward to answer and the revisions requested are pretty minor – I have therefore marked the paper as “Accepted with minor revisions” – but will need to see the responses of the authors before I can give my final verdict.

Review for authors:

Thank you for this interesting paper, which looks at the impact of a CHW home visiting intervention on several outcomes, including linear growth, development and neural function through electroencephalography (EEG) and saccadic reaction time (SRT) testing, using a cluster randomized control trial design.

I found the approach of using the assessment of children’s neural function as an outcome measure interesting, unusual and innovative, but feel that the detailed focus in the methods and outcomes sections of the paper on the EEG and SRT assessment has resulted in the omission of critical information for your readers about the intervention itself and its theoretical basis. There is also no information about what type of visits the mother-child dyads in the control group received – my understanding from the South African WBOT system is that CHW are expected to perform several postnatal home visits (in the methods section you mention that all children under 2 years are expected to be visited at home monthly). In the light of this, could you please answer the following:

1) How does the intervention compare to the standard care received in the control group? Describe the usual standard care in the control group and whether this was monitored in any way. There needs to a paragraph or a subsection in the methods clearly contrasting the intervention group with the control group and showing your reader the difference between the two groups.
2) I assume that randomization of WBOT clusters of CHW was performed before training with the “job aid” occurred and that only CHW that were on the intervention side received the training? If this is the case, it would be good to mention this under the methods section. It is not clear from the flow chart (Fig2) of the “Trial profile” how many CHW were in the control group and how many were in the intervention group. Please mention this in the text and add this into the figure.
3) Further – in figure 2 – for the lost to follow up boxes it would be better to use the term “dyads” rather than “participants” to remain internally consistent. Under deaths, it please differentiate between maternal and child/infant deaths.
4) Were all the CHW proficient in English? Did trainings occur in English or in Sepedi or Xitsonga? How long was the training period for the tool? You mention that didactic trainings occurred, and that live demonstrations of play activities were shown, but was there any in-the-field training and supervision of CWH who were using the job aid? For those CHW who were not proficient in English, were there translated versions of the job-aid available or was this not deemed necessary?
5) The job aid looks like an amazing resource which would be useful to CHW all over South Africa. Is it available for use by and distribution to government and NGO CHW in other provinces? Would it be possible to make it available for the patient-facing page to be translated into other South African languages?
6) As a rural clinician, I can see the practical utility of the job aid for CHW as a straightforward, simple and well-structured tool to guide CHW visits in homes. It would be good for you to motivate in the paper why you regard it as a useful tool that might make a difference to child outcomes. Was there any theoretical/evidence basis for its contents and structure?
7) It isn’t clear from the paper whether the main focus of the study was to test whether the job aid was a useful intervention tool that would help CHW and improve child outcomes, or whether your focus was on seeing whether the neurological assessments were practically possible to do and helpful from an outcome perspective. This needs to be clarified in the text.
8) It is a pity that only about half the clusters were assessed using EEG and SRT. Was this because of cost or because of distance or time? It would be good to explain why only half to a third of the children recruited into the trial were assessed in this way
9) How long did each full neurological assessment take? What is the level of expertise required by operators of the EEG and SRT equipment? Does this need to be done by a medical doctor or can it be done by a lay worker who has been trained? Again, I think this would be useful information for your readers.
10) I assume that you attempted to recruit all children born in the catchment area of each WBOT after 15 December 2017 to ensure that there was no selection bias – could you expand a bit more about exactly how mother-child dyads were recruited into the study? You mention “active monitoring by CHWs” and that health facility records were reviewed – were these hospital and clinic birth registers or other clinic or hospital records? Please include this in the manuscript.
11) You mention that once details of children born were found, dyads were recruited in their homes and that household survey data were also collected at baseline. What was the average age of the infants at baseline? And was any anthropometric data collected at this baseline visit?
12) Under the “Data collection” (line 142) of the Methods section there is no mention of when anthropometric measurements were performed, and whether these were once-off or serial measurements. The data collection for secondary outcomes, including caregiver engagement, dietary diversity score and household wealth index and should also be included here and not later, under “Outcomes”, (line 295 to 298) as it is currently.
13) At line 345 and 346 you mention that 266 intervention group dyads and 224 control group dyads were recruited for the lab portion of the study. Please put in the TOTAL number recruited to the lab section (490) in here too. There was a big drop off from recruitment into the lab portion at baseline to the first lab visit around 7 months (from 490 to 316) – but no drop out from the first to the second lab visit and only a small drop off to the endline assessment. Do you have any understanding why there was such a big, initial loss to follow up? Were participants nervous of the EEG testing etc. and therefore decide to withdraw or were there any other clear causes of this drop off?
14) As I have mentioned earlier, the neurological assessments you performed in the lab are an interesting and innovative, but as a public health clinician and researcher, I found the explanations very wordy, complicated and difficult to understand. It would be good to, in the introduction, write a little bit more about the utility and potential benefits of neural testing for early life interventions in simple, accessible language. And in the methods and results sections, it would also be useful to have a short, clear summary at the end of each technical section that cuts through the jargon and makes the EEG/SRT findings understandable by non-neurologists and non-specialists.
15) In the discussion, you mention that SRT was significantly reduced, indicating positive impact. However, while the EEG the impact on gamma power was significant, it was in the opposite direction to what you expected, and you reference an article indicating that this measure might not be the most appropriate one to use in this context because of the impacts of nutritional deprivation on scull conductivity. Why not just recommend SRT on its own? Your findings seem to indicate that EEG findings are confusing and counter to expectations. In the absence of good literature that suggests alternative, appropriate EEG measures in the rural African context, would a fairer conclusion of your study not be that SRT seemed helpful, yet EEG findings were counter to expectation and cannot, on the basis of this study, be recommended for use in the low-income African context util more appropriate measures are found?
16) It appears that only one height for age measure and Z score was calculated on each child at endline? Is there any reason why you decided not to perform serial height measurements over time – which may have given a better picture of the growth of the children investigated?
17) As one of your conclusions, you indicate that you believe that preforming EEG and SRT measurements are feasible in the rural African context. It would be useful if readers could get a sense of the time, expertise required and what the cost of running these neural tests would be.
18) Finally – SRT in particular appears to be a more sensitive marker of neurological and developmental impact than other traditional measurements such as growth or developmental markers, but it is a pity that these positive SRT effects seem to have faded a year after the intervention ended. Do you think that CHW should continue household interventions with beyond 2 years – and if so, how regularly and for how long? This may be worth raising in your discussion and conclusions.

Thanks again for this interesting paper. I am hopeful that your work will help researchers to investigate the impact of home visiting CHW interventions with more sensitive tools.
