# Supplementary material for: Evaluation of a community health worker home visit intervention to improve child development in South Africa: A cluster-randomized controlled trial
Source: PLoS Med. 2023 Apr 14;20(4):e1004222. doi: 10.1371/journal.pmed.1004222 (PMC10146459; doi:10.1371/journal.pmed.1004222)
Supplement: Attachment — Submitted filename: Response to reviewers R2.docx [file pmed.1004222.s010.docx]

Comments from the Academic Editor:

The authors need to address all the 4 reviewers’ comments in detail.

**Response: We have carefully addressed all the reviewers’ comments in detail, below.**

They should specifically address the issue of training CHWS and their supervisors.

**Response: We have provided additional detail on CHW trainings:**

***“******CHWs were trained on the job aid prior to the start of the intervention period and were provided with refresher trainings every six months thereafter, amounting to four trainings in total over the two-year intervention period. The first training lasted four days, while the subsequent refresher trainings lasted two days each. Trainings were conducted by members of the research team with logistical support from a local non-governmental organization that specialized in training CHWs for the Limpopo Department of Health.” (lines 171-176)***

***“******All CHWs were proficient in English, though many preferred to communicate in their home language (Xitsonga or Sepedi). Trainings were conducted in Sepedi and Xitsonga, which also prepared CHWs to use the job aid in the caregiver’s home language. In the six months that followed the first training, each CHW was accompanied on a home-visit by one of the trainers, who provided additional support and feedback to strengthen CHW knowledge around delivering the intervention.” (lines 180-186)***

**We have also provided additional detail on CHW participation in the study and trainings:**

***“Overall, 376 eligible CHWs were recruited for the study, of whom 356 (95%) participated. All participating CHWs in the intervention arm attended the first training, while 170 (83%) attended the second training, 183 (90%) attended the third training, and 186 (91%) attended the fourth training.” (lines 432-435)***

**With respect to supervision, we state:**

***“We integrated the intervention job aid and training into the existing CHW home visit infrastructure. We did not intervene in the local system of CHW supervision. As a result, important aspects of implementation were not under our control.” (lines 576-578)***

Additionally, the authors should:

1) give more information on program implementation, especially in the Methods section.

**Response: We have expanded our description of program implementation.**

**To clarify how the job aid was integrated into existing CHW operations, we have added a paragraph to the Methods describing the local standard of care:**

***“Participants in the control arm received the local standard of care throughout the study period. CHWs in the study area are responsible for making monthly home visits for all children under the age of two and for providing a broad range of services during those visits, including health screening, immunization monitoring, and nutrition counselling. The Road to Health Booklet serves as a guide for CHW visits as well as a record of the child’s growth and vaccination status. It also provides caregivers with important information on child health, nutrition, and common developmental milestones. The job aid used in the intervention arm expanded the content of home visits but was designed to be effort neutral by restructuring the time CHWs were already responsible for allotting.” (lines 162-170)***

**We have clarified that the structure of the job aid was informed by recent evidence on implementation factors for home visit interventions:**

***“The content and structure of the job aid were developed over the course of the year that preceded the start of the trial through a process that included several steps. Content development was informed by a robust body of evidence demonstrating that home visits covering a diversity of topics, including child health, nutrition, and play-based stimulation, can generate significant positive effects on child development [2]. The structure of the job aid built on recent research showing that implementation factors, including design aspects of curricula, training manuals, and job aids, are critical in determining the effectiveness of home visit interventions [14].” (lines 127-133)***

**New reference [14]: Yousafzai AK, Aboud F. Review of implementation processes for integrated nutrition and psychosocial stimulation interventions. Annals of the New York Academy of Sciences. 2014;1308: 33-45.**

**We have provided additional detail on the measurement of implementation factors:**

***“Finally, data on implementation of the intervention were collected during a set of phone-based interviews conducted with one randomly selected caregiver served by each intervention CHW after the intervention had been running for one year. Caregivers were asked to self-report the time since the CHW last visited their home and whether at the last visit the CHW counseled the caregiver on the child development content of the job aid. These interviews were not conducted in the control arm. Our initial plan was to measure CHW compliance based on caregiver self-report at endline, but because of the delay in fieldwork due to COVID-19, the recall period was too long to generate quality data.” (lines 209-216)***

**We have included an additional result related to implementation:**

***“Among intervention CHWs who made a home visit in the prior month, 93% counselled caregivers on the child development content of the job aid.” (lines 504-505)***

2) revisit and give details of sample size determination including the power. How were missing data handled?

**Response: We have clarified that our power calculation accounted for the cluster-randomized design:**

***“The target sample size of 1,092 dyads enrolled across 51 clusters . . .” (line 372)***

**We have added a description of the power calculation for the lab subsample:**

***“The target for the lab subsample was 900 assessments across the three waves, which provided 80% power to detect a 0.25 SD difference in absolute gamma power and SRT, assuming 85% of assessments would meet the preset validity criteria for inclusion in the analysis, a negligible ICC for geographic clustering, and an ICC for repeated measures within dyads of 0.20.” (lines 375-379)***

**We have clarified that we used a complete-case approach to missing data:**

***“For all analyses, data from participants not assessed at endline were assumed to be missing at random and a complete-case approach was used.” (lines 396-397)***

**We have added new columns in Table 1 summarizing the demographics of dyads not assessed at endline for each arm. We have added the following text:**

***“In the control group, there was a significant positive association between number of older siblings and probability of being assessed at endline. There were no other significant differences between those assessed and those not assessed at endline.” (lines 441-444)***

**We did not find evidence of systematic differences. We mention this in the Discussion:**

***“Based on Table 1, there does not appear to be a systematic relationship between attrition and treatment group or participant demographics, supporting the use of the complete-case approach to dealing with missing data in the analysis.” (lines 602-604)***

3) revise the conclusions section and make sure that they are based on the study findings.

**Response: We have clarified our findings related to the intervention’s impact on neural function. We now say that *“we found significant improvement in SRT”* (Abstract; lines 524-525). We have added the following interpretation of the EEG results:**

***“***

***The similar effect sizes on absolute gamma and total EEG power—and the corresponding null effect on relative gamma power—suggest an anatomical explanation rather than evidence of a negative effect on neural function.” (Abstract; lines 566-569)***

**We have removed mention of strengthening supervision as a conclusion from the abstract and main paper.**

**We have added the following to the first paragraph of the Discussion to better highlight our finding related to the use of EEG and eye-tracking technologies:**

***“Furthermore, we were able to collect high quality data on neural function using EEG and eye-tracking technologies, confirming that the use of these measures is feasible in low-resource settings.” (lines 525-527)***

4) clearly show the readers how to access the trial registration details on- PACTR201710002683810 and include the relevant link.

**Response: We have added the URL for the PACTR record to the text:**

***“https://pactr.samrc.ac.za/TrialDisplay.aspx?TrialID=2683” (line 44)***

5) give details on reliability of the MDAT.

**Response: We have expanded our description of the MDAT and added estimates of reliability:**

***“******The interobserver reliability of the tool is high, with 99% of items having a kappa above 0.75, based on assessments of the same child conducted independently on the same day by two observers [24]. The MDAT has been used in recent intervention trails conducted in several countries in sub-Saharan Africa [25-29].” (lines 308-311)***

24. **Gladstone M, Lancaster GA, Umar E, Nyirenda M, Kayira E, van den Broek NR, et al. The Malawi Developmental Assessment Tool (MDAT): the creation, validation, and reliability of a tool to assess child development in rural African settings. PLoS Medicine. 2010;7: e1000273.**
25. **Prado EL, Maleta K, Ashorn P, Ashorn U, Vosti SA, Sadalaki J, et al. Effects of maternal and child lipid-based nutrient supplements on infant development: a randomized trial in Malawi. American Journal of Clinical Nutrition. 2016;103:784-93.**
26. **Gladstone MJ, Chandna J, Kandawasvika G, Ntozini R, Majo FD, Tavengwa NV, et al. Independent and combined effects of improved water, sanitation, and hygiene (WASH) and improved complementary feeding on early neurodevelopment among children born to HIV-negative mothers in rural Zimbabwe: substudy of a cluster-randomized trial. PLoS Medicine. 2019;16:e1002766.**
27. **Prado EL, Adu-Afarwuah S, Lartey A, Ocansey M, Ashorn P, Vosti SA, et al. Effects of pre-and post-natal lipid-based nutrient supplements on infant development in a randomized trial in Ghana. Early Human Development. 2016;99:43-51.**
28. **Jensen SK, Placencio-Castro M, Murray SM, Brennan RT, Goshev S, Farrar J, et al. Effect of a home-visiting parenting program to promote early childhood development and prevent violence: a cluster-randomized trial in Rwanda. BMJ Global Health. 2021;6:e003508.**
29. **Olsen MF, Iuel-Brockdorff AS, Yaméogo CW, Cichon B, Fabiansen C, Filteau S, et al. Impact of food supplements on early child development in children with moderate acute malnutrition: A randomised 2 x 2 x 3 factorial trial in Burkina Faso. PLoS Medicine. 2020;17:e1003442.**

**Requests from the editors:**

Abstract Methods and Findings:

* Please ensure that all numbers presented in the abstract are present and identical to numbers presented in the main manuscript text.

**Response: We have confirmed this.**

* Please specify who was blinded to the intervention and control.

**Response: We have clarified:**

***“Group assignment was masked from all data collectors, but masking of participating CHWs and dyads was not possible” (Abstract)***

* Please define the intervention and control states.

**Response: We have done this:**

***“Intervention CHWs were trained on a job aid that included content on child health, nutrition, developmental milestones, and encouragement to engage in developmentally appropriate play-based activities. They were instructed to use the job aid to guide regular monthly home visits with caregivers of children under two years of age. Control CHWs were responsible for conducting regular monthly home visits without additional training or the job aid, in accordance with the standard of care in the study area.” (Abstract)***

* Please include the important covariates that are adjusted for in the analyses.

**Response: We have described the covariates included in the adjusted models:**

***“Adjusted models included a set of demographic covariates measured at baseline: child age, sex, and number of siblings; household wealth and receipt of Child Support Grant; caregiver age and education; and assessor fixed effects.” (Abstract)***

* Please provide the number of participants lost to follow up in each group.

**Response: We have added the number of dyads not assessed at endline in each group.**

***“At endline (last assessment June 11, 2021), 432 dyads (71%; 175 dyads not assessed at endline) in 26 clusters remained in the intervention group and 332 dyads (68%; 156 dyads not assessed at endline) in 25 clusters remained in the control group.” (Abstract)***

* In the last sentence of the Abstract Methods and Findings section, please describe the main limitation(s) of the study's methodology.

**Response: We have added the following main limitation of the study:**

***“Due to the COVID-19 pandemic, we were not able to assess outcomes until one year after the end of the intervention period.” (Abstract)***

**Additional note: The editors and reviewers have requested that substantial additional detail be included in the abstract. To adequately respond to these requests, it was necessary to exceed the limit of 500 words. We politely request an exception to the word count limit. We welcome your guidance on how to proceed.**

At this stage, we ask that you include a short, non-technical Author Summary of your research to make findings accessible to a wide audience that includes both scientists and non-scientists. The Author Summary should immediately follow the Abstract in your revised manuscript. This text is subject to editorial change and should be distinct from the scientific abstract. Please see our author guidelines for more information: <https://journals.plos.org/plosmedicine/s/revising-your-manuscript#loc-author-summary>

**Response: We have added an Author Summary after the Abstract.**

Please conclude the Introduction with a clear description of the study question or hypothesis.

**Response: We have added the following at the end of the Introduction section:**

***“******We tested a primary hypothesis that a CHW-delivered home visit intervention would improve child development in South Africa.” (lines 36-37)***

Please remove the ‘Role of the funding source’ section.

**Response: We have removed this section as requested.**

Please cite the items in your Supporting Information at the relevant points in the main text (guidance for how to do this can be found here: <https://journals.plos.org/plosmedicine/s/supporting-information>)

**Response: We have updated the Supporting Information citations to be consistent with the provided guidance.**

Please specify whether informed consent was written or oral.

**Response: We have clarified that informed consent was written (line 86).**

Please define "lost to follow-up" as used in this study. Other reasons for exclusion should be defined.

**Response: We have removed the term “lost to follow-up” from Fig 2 and the manuscript entirely and replaced it with “not assessed at endline” to be clearer. We have added a detailed breakdown in Fig 2 of the reasons why children were not assessed at endline, including child death, refusal, relocation, and inability to reach.**

Thank you for including the CONSORT checklist as Supporting Information. Please update the checklist to use section and paragraph numbers, rather than page numbers.

**Response: We have updated the CONSORT checklist (S1 Table) with section and paragraph numbers.**

The key exclusion criteria in the Methods differ from those described in the PACTR registry. Please explain, including in the manuscript.

**Response: We have updated the manuscript to be consistent with what is stated in the PACTR registry. Consistent with the PACTR, we have clarified that caregivers needing to be at least 18 years of age was an inclusion criterion rather than an exclusion criterion as previously stated. We have also expanded the description of inclusion criteria for participating CHWs to be consistent with the PACTR:**

***“All CHWs who were part of WBOTs operating in Greater Tzaneen and Greater Giyani and met the following eligibility criteria were invited to participate in the study: active as a CHW for more than three months at the time of enrollment; at least 18 years of age; completed the standard Phase I training for CHWs in South Africa; not using mHealth technologies as part of routine activities; not participating in another research project; and had an eligible caregiver-child dyad in their catchment area.” (lines 48-53)***

The secondary outcomes measures are not described in the study protocol. If the outcomes were not prespecified in the protocol, please indicate that they were post hoc and explain why they were added. Post hoc comparisons should be presented as hypothesis generating rather than conclusive.

**Response: We have added the following text to the description of the secondary outcomes:**

***“These outcomes were not prespecified in the study protocol and are meant to be hypothesis generating with respect to potential behaviour change pathways through which the intervention could influence child development.” (lines 364-367)***

The trial registration lists the outcomes of ‘autonomic assessments of each child’s heartbeat and breathing’ and ‘blood samples from children tested for biomarkers of systemic inflammation’. The trial registration also states that ‘an additional sample of 50 additional caregiver/child pairs of high socioeconomic status (SES) will be recruited from Greater Tzaneen and assessed with EEG and ET technologies to explore the relationship between household wealth and child neurodevelopment’. (a) Can you please present those results as part of this manuscript, or indicate why that is not possible? (b) Can you please indicate when you plan to publish those results?

**Response: Thank you for this comment. We collected autonomic measures of child heartbeat and breathing at the second and third waves of lab visits, and we collected child blood samples during the third wave. Due to challenges related to COVID-19, we were not able to collect EEG and eye-tracking data from additional high-SES dyads. Our intention was not to include the autonomic and blood-based assessments as outcomes in the trial, but rather to explore related relationships with descriptive analyses. We are not able to present those data in this paper because the autonomic data are still being processed and blood sample testing is not yet complete at the lab in South Africa. We expect to analyze and publish these data in 2023. We have added the following clarification to the manuscript:**

***“At the second and third wave of lab visits children were assessed for autonomic measures of heartbeat and breathing, and, at the third wave, blood samples were collected and tested for biomarkers of systemic inflammation. These data will be analyzed in future research.” (lines 295-297)***

Please define the abbreviation in Figure 3 (saccadic reaction time).

**Response: We have defined the abbreviation in the notes for Fig 3.**

Lines 459-460: As your trial had to undergo important modifications in response to extenuating circumstances, please complete the CONSERVE-CONSORT checklist and provide in your Supporting Information. When completing the checklist, please use section and paragraph numbers, rather than page numbers.

**Response: We have included a completed CONSERVE-CONSORT checklist (S5 Table) covering changes to the study that resulted from the delay in endline data collection due to COVID-19.**

Did your study have a prospective analysis plan? Please state this (either way) early in the Methods section.

a) If a prospective analysis plan was used in designing the study, please include the relevant prospectively written document with your revised manuscript as a Supporting Information file to be published alongside your study, and cite it in the Methods section. A legend for this file should be included at the end of your manuscript.

b) If no such document exists, please make sure that the Methods section transparently describes when analyses were planned, and when/why any data-driven changes to analyses took place.

c) In either case, changes in the analysis-- including those made in response to peer review comments-- should be identified as such in the Methods section of the paper, with rationale.

**Response: Thank you for this comment. We have added the following text to the Methods:**

***“******We did not publish a written prospective analysis plan. The specifications of the main models, including the use of linear mixed models with random effects and the selection of control variables to include in adjusted models, were planned prior to endline data collection. The measure of CHW compliance used in the per-protocol analysis was also determined prior to endline data collection.*** ***The decision to include absolute EEG total power and relative EEG gamma power as additional outcomes was made after analyzing the impact of the intervention on absolute EEG gamma power in order to better understand those results.” (lines 408-414)***

For reference 21, please include the date cited.

**Response: We have made the requested change.**

RE reference 44 listed as in preparation, papers cannot be listed i the reference list until they have been accepted for publication or are publicly available on a preprint archive. The information may be cited in the text as a personal communication with the author if the author provides written permission to be named. Alternatively, please provide a different appropriate reference.

**Response: This manuscript is now available on OSF Preprints (https://osf.io/pjhk4). We have updated the reference list with the correct citation.**

**Additional note: In a final check of the dataset, we identified a minor difference in a filtering parameter for the SRT variable at wave 3 as compared to waves 1 and 2. Correcting this did not affect the results of the analyses, which have been updated.**

**Reviewer #1: Statistical review**

This paper reports a cluster randomised trial assessing the effectiveness of a community health worker delivered intervention on child development.

Generally the trial used suitable statistical methods and was reported well, with changes from protocol were clearly explained. I had some minor comments which are provided below:

1.	Abstract "had no impact" could be "had no significant impact" (and similarly for subsequent results). I would recommend using alternative phrasing to 'a beta' - perhaps mean difference?

**Response: We have added the modifier “significant” in the abstract and throughout the paper, where appropriate. We have replaced “β” with “mean difference (MD)” in the abstract and main text.**

2.	Abstract/outcomes: It seemed that some secondary outcomes (e.g. child diet) were not reported in the abstract. Generally it is best practice to report all secondaries (or at least add 'no significant effect for other secondary outcomes) or none.

**Response: We do not report any secondary outcomes in the abstract. As per our response to the comment from the editors above, we have clarified in the Methods that the secondary outcomes (caregiver engagement and child diet) were meant to be hypothesis generating:**

***“These outcomes were not prespecified in the study protocol and are meant to be hypothesis generating with respect to potential behaviour change pathways through which the intervention could influence child development.” (lines 364-367)***

3.	Trial registration: I could not find PACTR201710002683810 on the PACTR website - can a link be provided? I would recommend all secondary outcomes mentioned in the registration page are reported in the paper (or a reason given why they cannot be).

**Response: We have added the URL for the PACTR record to the text:**

***“https://pactr.samrc.ac.za/TrialDisplay.aspx?TrialID=2683” (line 44)***

**As mentioned above, we have clarified in the Methods that the secondary outcomes (caregiver engagement and child diet) were no prespecified in the study protocol:**

***“These outcomes were not prespecified in the study protocol and are meant to be hypothesis generating with respect to potential behaviour change pathways through which the intervention could influence child development.” (lines 364-367)***

4.	Statistical analysis: I would recommend the text about the variables that were adjusted for is moved to earlier in the statistical analysis section (when read the early parts I had thought it had been missed out).

**Response: We have moved the description of the adjusted models to earlier in the statistical analysis section.**

5.	Statistical analysis: The primary analysis would account for missing at random data. Since the proportion of participants who dropped out was relatively high it would be good to mention how missing data was dealt with. The discussion might mention whether the missing at random assumption is plausible.

**Response: Thank you for this comment. We have clarified this point in the Methods:**

***“For all analyses, data from participants not assessed at endline were assumed to be missing at random and a complete-case approach was used.” (lines 396-397)***

**We have added new columns in Table 1 summarizing the demographics of dyads not assessed at endline for each arm. We have added the following text:**

***“In the control group, there was a significant positive association between number of older siblings and probability of being assessed at endline. There were no other significant differences between those assessed and those not assessed at endline.” (lines 441-443)***

**We did not find evidence of systematic differences. We mention this in the Discussion:**

***“Based on Table 1, there does not appear to be a systematic relationship between attrition and treatment group or participant demographics, supporting the use of the complete-case approach to dealing with missing data in the analysis.” (lines 602-604)***

**Reviewer #2**

Thank you very much for requesting a review of this article.

This paper presents a cluster randomised trial of a home visiting intervention integrated into community health worker practice in South Africa looking at an intervention that provided health workers with a job aid that included information on child health, nutrition, developmental milestones and encouragement to engage in developmentally appropriate play based activities on monthly visits for children less than 2 years of age. This is linked to the government's proposed National Integrated Early Childhood Development Policy programme.

The team measured household data as well as anthropometry, child development using MDAT, EEG and saccadic reaction time. It is an interesting study and demonstrates a few important results that should be published - in particular the early changes in neural testing on EEG and saccadic reaction time.

Generally, it is a well written and clear paper and I have few queries - although would recommend that a statistician review the cluster trial analysis. My points are outlined below and mainly relate to representativeness of the sample tested:

1. Recruitment occurred through "active monitoring and review of health facility records" - do we have an idea as to what "reach" this created and whether this identified a representative sample of the population?

**Response: We have strong reason to believe that our recruitment protocol reached a sample that is representative of the population in the study area. We have added the following to the Methods:**

***“According to the South Africa Demographic and Health Survey 2016 [13], 98% of births in Limpopo occur at health facilities and the vast majority are at public clinics and hospitals.” (lines 71-73)***

**New reference [13]: National Department of Health (NDoH), Statistics South Africa (Stats SA), South African Medical Research Council (SAMRC), and ICF. South Africa Demographic and Health Survey 2016. Pretoria, South Africa, and Rockville, Maryland, USA: NDoH, Stats SA, SAMRC, and ICF, 2016.**

Table 1 provides info on study participants including those enrolled and those who had endline assessments - is it clear if there were any significant differences between these groups?

**Response: We have added new columns in Table 1 summarizing the demographics of dyads not assessed at endline for each arm. We have added the following text:**

***“In the control group, there was a significant positive association between number of older siblings and probability of being assessed at endline. There were no other significant differences between those assessed and those not assessed at endline.” (lines 441-443)***

**We did not find evidence of systematic differences. We mention this in the Discussion:**

***“Based on Table 1, there does not appear to be a systematic relationship between attrition and treatment group or participant demographics, supporting the use of the complete-case approach to dealing with missing data in the analysis.” (lines 602-604)***

2. Is there any information about whether those who came for neural testing were representative of the sample as well as the population? Line 351 makes out that there were no clear differences in baseline demographics comparing observations included to those excluded - does this mean those included for neural testing vs excluded from neural testing had similar demographics. I think this might be the case but the way it was written was not entirely clear.

**Response: Thank you for this comment. The statement being referred to is a comparison of observations within the lab subsample, across groupings based on preset validity criteria for data inclusion in the analysis.**

**We have added new text describing a comparison between those assessed at the lab and those not assessed at the lab:**

***“The subsample of participants assessed at the lab differed slightly from the total enrolled sample. In particular, household wealth was higher among those assessed at the lab. However, this was driven by the fact that only households residing in Greater Tzaneen were recruited to attend the lab. The demographics of participants assessed at the lab were very similar to the demographics of the total enrolled sample in Greater Tzaneen.” (lines 444-448)***

3. Lines 127 - 131 - Are there any reasons why there was a change in emphasis to "responsiveness" in the activities promoted in the guides? Could this be explained better?

**Response: We have clarified our exploratory aim in developing two versions of the job aid:**

***“******Our aim in developing and using two versions of the job aid was to explore the relative importance of strengthening the caregiver-child relationship with a greater focus on responsiveness, versus strengthening child skills with a greater focus on developmental stimulation.” (lines 158-161)***

4. MDAT assessments - any information on the reliability of these assessments and any reliability done prior to starting these assessments? Was it translated and back translated into local language?

**Response: We have expanded our description of the MDAT and added estimates of reliability:**

***“The interobserver reliability of the tool is high, with 99% of items having a kappa above 0.75, based on assessments of the same child conducted independently on the same day by two observers [24]. The MDAT has been used in recent intervention trails conducted in several countries in sub-Saharan Africa [25-29].” (lines 308-311)***

24. **Gladstone M, Lancaster GA, Umar E, Nyirenda M, Kayira E, van den Broek NR, et al. The Malawi Developmental Assessment Tool (MDAT): the creation, validation, and reliability of a tool to assess child development in rural African settings. PLoS Medicine. 2010;7: e1000273.**
25. **Prado EL, Maleta K, Ashorn P, Ashorn U, Vosti SA, Sadalaki J, et al. Effects of maternal and child lipid-based nutrient supplements on infant development: a randomized trial in Malawi. American Journal of Clinical Nutrition. 2016;103:784-93.**
26. **Gladstone MJ, Chandna J, Kandawasvika G, Ntozini R, Majo FD, Tavengwa NV, et al. Independent and combined effects of improved water, sanitation, and hygiene (WASH) and improved complementary feeding on early neurodevelopment among children born to HIV-negative mothers in rural Zimbabwe: substudy of a cluster-randomized trial. PLoS Medicine. 2019;16:e1002766.**
27. **Prado EL, Adu-Afarwuah S, Lartey A, Ocansey M, Ashorn P, Vosti SA, et al. Effects of pre-and post-natal lipid-based nutrient supplements on infant development in a randomized trial in Ghana. Early Human Development. 2016;99:43-51.**
28. **Jensen SK, Placencio-Castro M, Murray SM, Brennan RT, Goshev S, Farrar J, et al. Effect of a home-visiting parenting program to promote early childhood development and prevent violence: a cluster-randomized trial in Rwanda. BMJ Global Health. 2021;6:e003508.**
29. **Olsen MF, Iuel-Brockdorff AS, Yaméogo CW, Cichon B, Fabiansen C, Filteau S, et al. Impact of food supplements on early child development in children with moderate acute malnutrition: A randomised 2 x 2 x 3 factorial trial in Burkina Faso. PLoS Medicine. 2020;17:e1003442.**

**We have clarified the point on translations in the Methods:**

***“Two nurses with expertise and experience in administering the MDAT traveled from Malawi to Tzaneen where they supported translation of the instrument and conducted an extensive training of a team of study assessors. The MDAT instrument was translated and back translated into Sepedi and Xitsonga prior to administration.” (lines 312-315)***

5. The abstract makes out that the significant improvements in EEG and saccades (neural testing) were throughout all time points (or at least it does not specify). I wonder if it might be important in the abstract to try and be clearer that the significant findings were only there in wave 1 and 2 and not in wave 3 and that similarly anthropometry and MDAT were only measured in wave 3.

**Response: Thank you for this suggestion. We have added the following:**

***“******While the effect on SRT was observed at the first two lab visits, it was no longer present at the third visit, which coincided with the overall endline assessment.” (Abstract)***

**And:**

***“Data on child anthropometry, development, and diet were collected from the full sample during endline surveys only.” (Abstract)***

**Reviewer #3**

Thank you for this interesting paper, which looks at the impact of a CHW home visiting intervention on several outcomes, including linear growth, development and neural function through electroencephalography (EEG) and saccadic reaction time (SRT) testing, using a cluster randomized control trial design.

I found the approach of using the assessment of children's neural function as an outcome measure interesting, unusual and innovative, but feel that the detailed focus in the methods and outcomes sections of the paper on the EEG and SRT assessment has resulted in the omission of critical information for your readers about the intervention itself and its theoretical basis. There is also no information about what type of visits the mother-child dyads in the control group received - my understanding from the South African WBOT system is that CHW are expected to perform several postnatal home visits (in the methods section you mention that all children under 2 years are expected to be visited at home monthly). In the light of this, could you please answer the following:

1)	How does the intervention compare to the standard care received in the control group? Describe the usual standard care in the control group and whether this was monitored in any way. There needs to a paragraph or a subsection in the methods clearly contrasting the intervention group with the control group and showing your reader the difference between the two groups.

**Response: We have added the following paragraph describing the local standard of care received by participants in the control arm:**

***“******Participants in the control arm received the local standard of care throughout the study period. CHWs in the study area are responsible for making monthly home visits for all children under the age of two and for providing a broad range of services during those visits, including health screening, immunization monitoring, and nutrition counselling. The Road to Health Booklet serves as a guide for CHW visits as well as a record of the child’s growth and vaccination status. It also provides caregivers with important information on child health, nutrition, and common developmental milestones. The job aid used in the intervention arm expanded the content of home visits but was designed to be effort neutral by restructuring the time CHWs were already responsible for allotting.” (lines 162-170)***

2)	I assume that randomization of WBOT clusters of CHW was performed before training with the "job aid" occurred and that only CHW that were on the intervention side received the training? If this is the case, it would be good to mention this under the methods section. It is not clear from the flow chart (Fig2) of the "Trial profile" how many CHW were in the control group and how many were in the intervention group. Please mention this in the text and add this into the figure.

**Response: We have clarified the point about the timing of the randomization and the training of the CHWs:**

***“The randomization was performed prior to the start of CHW training and only CHWs in the intervention arm were trained on the study job aid.” (lines 90-92)***

**We have also added the number of participating CHWs (control - 152; intervention - 204) to Fig 2 and the text (lines 420-421).**

3)	Further - in figure 2 - for the lost to follow up boxes it would be better to use the term "dyads" rather than "participants" to remain internally consistent. Under deaths, it please differentiate between maternal and child/infant deaths.

**Response: We have made the suggested changes.**

4)	Were all the CHW proficient in English? Did trainings occur in English or in Sepedi or Xitsonga?

**Response: We have clarified:**

***“******All CHWs were proficient in English, though many preferred to communicate in their home language (Xitsonga or Sepedi). Trainings were conducted in Sepedi and Xitsonga, which also prepared CHWs to use the job aid in the caregiver’s home language.” (lines 180-183)***

How long was the training period for the tool?

**Response: We have clarified:**

***“CHWs were trained on the job aid prior to the start of the intervention period and were provided with refresher trainings every six months thereafter, amounting to four trainings in total over the two-year intervention period. The first training lasted four days, while the subsequent refresher trainings lasted two days each.” (lines 171-174)***

You mention that didactic trainings occurred, and that live demonstrations of play activities were shown, but was there any in-the-field training and supervision of CWH who were using the job aid?

**Response: We have clarified:**

***“In the six months that followed the first training, each CHW was also accompanied on a home-visit by one of the trainers, who provided additional support and feedback to strengthen CHW knowledge around delivering the intervention.” (lines 183-186)***

For those CHW who were not proficient in English, were there translated versions of the job-aid available or was this not deemed necessary?

**Response: It was deemed unnecessary to have translated versions of the CHW-facing side of the job aid. On the caregiver-facing side, all content was presented in English, Xitsonga, and Sepedi.**

5)	The job aid looks like an amazing resource which would be useful to CHW all over South Africa. Is it available for use by and distribution to government and NGO CHW in other provinces? Would it be possible to make it available for the patient-facing page to be translated into other South African languages?

**Response: Thank you for this comment. All intervention resources are freely available to anyone interested. We have added this point:**

***“Full versions of both job aids are provided in S4 Figure;*** ***additional intervention resources are available from the corresponding author upon request.” (lines 156-158)***

6)	As a rural clinician, I can see the practical utility of the job aid for CHW as a straightforward, simple and well-structured tool to guide CHW visits in homes. It would be good for you to motivate in the paper why you regard it as a useful tool that might make a difference to child outcomes. Was there any theoretical/evidence basis for its contents and structure?

**Response: We have added the following:**

***“******The content and structure of the job aid were developed over the course of the year that preceded the start of the trial through a process that included several steps.*** ***Content development was informed by a robust body of evidence demonstrating that home visits covering a diversity of topics, including child health, nutrition, and play-based stimulation, can generate significant positive effects on child development [2].*** ***The structure of the job aid built on recent research showing that implementation factors, including design aspects of curricula, training manuals, and job aids, are critical in determining the effectiveness of home visit interventions [14].” (lines 127-133)***

**New reference [14]:** **Yousafzai AK, Aboud F. Review of implementation processes for integrated nutrition and psychosocial stimulation interventions. Annals of the New York Academy of Sciences. 2014;1308: 33-45.**

7)	It isn't clear from the paper whether the main focus of the study was to test whether the job aid was a useful intervention tool that would help CHW and improve child outcomes, or whether your focus was on seeing whether the neurological assessments were practically possible to do and helpful from an outcome perspective. This needs to be clarified in the text.

**Response: Apologies for the lack of clarity. We were primarily interested in the impact on child outcomes; the neurological assessments were an innovative aspect of the outcome measurement. We have added text at the end of the Introduction to clarify this point:**

***“We tested a primary hypothesis that a CHW-delivered home visit intervention would improve child development in South Africa.” (lines 36-37)***

8)	It is a pity that only about half the clusters were assessed using EEG and SRT. Was this because of cost or because of distance or time? It would be good to explain why only half to a third of the children recruited into the trial were assessed in this way.

**Response: We have clarified this point:**

***“Eligibility for the lab subsample was restricted to one subdistrict due to budget constraints.” (lines 81-82)***

9)	How long did each full neurological assessment take? What is the level of expertise required by operators of the EEG and SRT equipment? Does this need to be done by a medical doctor or can it be done by a lay worker who has been trained? Again, I think this would be useful information for your readers.

**Response: We note that “EEG data were recorded for 6 minutes” (line 254) And “Eye-tracking assessments lasted approximately 10 minutes” (line 292).**

**We have clarified:**

***“The average time to complete both assessments, including preparation activities and data collection, was around 45 minutes per child.” (lines 236-237)***

**And:**

***“Assessors did not have previous medical training nor prior experience with the technologies.” (lines 233-234)***

10)	I assume that you attempted to recruit all children born in the catchment area of each WBOT after 15 December 2017 to ensure that there was no selection bias - could you expand a bit more about exactly how mother-child dyads were recruited into the study? You mention "active monitoring by CHWs" and that health facility records were reviewed - were these hospital and clinic birth registers or other clinic or hospital records? Please include this in the manuscript.

**Response: We have expanded our description of the procedure for identifying eligible dyads:**

***“Eligible dyads were identified through active monitoring by CHWs, whose responsibilities include supporting pregnant women with accessing antenatal services provided at clinics, and review of birth registries at public hospitals and clinics in the study area.” (lines 68-71)***

11)	You mention that once details of children born were found, dyads were recruited in their homes and that household survey data were also collected at baseline. What was the average age of the infants at baseline? And was any anthropometric data collected at this baseline visit?

**Response: We have clarified:**

***“On average, children were 58 days old at the time of enrollment.” (line 439)***

**We did not collect anthropometric data at this visit.**

12)	Under the "Data collection" (line 142) of the Methods section there is no mention of when anthropometric measurements were performed, and whether these were once-off or serial measurements. The data collection for secondary outcomes, including caregiver engagement, dietary diversity score and household wealth index and should also be included here and not later, under "Outcomes", (line 295 to 298) as it is currently.

**Response: We have clarified the timing of the data collection:**

***“******Household surveys were administered at baseline (January – May 2018) and endline (September 2020 – June 2021). Demographic data and household asset information were collected during the baseline survey. Data on caregiver engagement were collected during baseline and endline surveys. Data on child anthropometry, development, and diet were collected during endline surveys only.” (lines 199-203)***

13)	At line 345 and 346 you mention that 266 intervention group dyads and 224 control group dyads were recruited for the lab portion of the study. Please put in the TOTAL number recruited to the lab section (490) in here too. There was a big drop off from recruitment into the lab portion at baseline to the first lab visit around 7 months (from 490 to 316) - but no drop out from the first to the second lab visit and only a small drop off to the endline assessment. Do you have any understanding why there was such a big, initial loss to follow up? Were participants nervous of the EEG testing etc. and therefore decide to withdraw or were there any other clear causes of this drop off?

**Response: We have added the total number of dyads (n=490) recruited to the lab subsample to the text (line 425). The lab participation rates were 64%, 64%, and 58% at the three waves, respectively. These rates are consistent with our expectations based on our previous experience in similar settings; we achieved our target sample size of 900 lab observations across the three waves (now described in the Methods, lines 375-379). We did not conduct a formal assessment of reasons for non-participation in the lab assessment but based on anecdotal evidence we believe some parents felt traveling with their child to the lab was too onerous while others were nervous about the assessments themselves.**

14)	As I have mentioned earlier, the neurological assessments you performed in the lab are an interesting and innovative, but as a public health clinician and researcher, I found the explanations very wordy, complicated and difficult to understand. It would be good to, in the introduction, write a little bit more about the utility and potential benefits of neural testing for early life interventions in simple, accessible language. And in the methods and results sections, it would also be useful to have a short, clear summary at the end of each technical section that cuts through the jargon and makes the EEG/SRT findings understandable by non-neurologists and non-specialists.

**Response: We appreciate this comment. We have tried to be thoughtful in how we describe these measures because this paper is one of the first to present them in the context of a public health trial in an LMIC. While we agree that it is important to minimize jargon and make the descriptions of the measures as accessible as possible, we also feel that it is important to explain them fully, which requires summarizing a literature established over several decades in other disciplines. We have done our best to balance these considerations.**

15)	In the discussion, you mention that SRT was significantly reduced, indicating positive impact. However, while the EEG the impact on gamma power was significant, it was in the opposite direction to what you expected, and you reference an article indicating that this measure might not be the most appropriate one to use in this context because of the impacts of nutritional deprivation on scull conductivity. Why not just recommend SRT on its own? Your findings seem to indicate that EEG findings are confusing and counter to expectations. In the absence of good literature that suggests alternative, appropriate EEG measures in the rural African context, would a fairer conclusion of your study not be that SRT seemed helpful, yet EEG findings were counter to expectation and cannot, on the basis of this study, be recommended for use in the low-income African context util more appropriate measures are found?

**Response: A vast literature confirms that EEG is a powerful tool for understanding brain development. Our study demonstrates that collecting EEG data in low-resource settings is feasible, which we believe is an important contribution. Our findings indicate that future work is needed to identify the best EEG-based outcome measures for intervention trials in such settings, and there are several commonly used alternative EEG measures that could be promising in this regard. We could not explore those measures in this paper because we did not prespecify them in our protocol.**

**We mention the need for future work on this in the Discussion:**

***“The use of EEG data in intervention trials in LMICs holds promise, but more work is needed to identify the most appropriate measures that can be constructed from these complex data for use in these contexts.” (lines 597-599)***

16)	It appears that only one height for age measure and Z score was calculated on each child at endline? Is there any reason why you decided not to perform serial height measurements over time - which may have given a better picture of the growth of the children investigated?

**Response: Most study participants were only visited at baseline and endline. We did not have the budget to conduct additional interim household visits.**

17)	As one of your conclusions, you indicate that you believe that preforming EEG and SRT measurements are feasible in the rural African context. It would be useful if readers could get a sense of the time, expertise required and what the cost of running these neural tests would be.

**As per our response to comment #9 above: we note that “EEG data were recorded for 6 minutes” (line 254) And “Eye-tracking assessments lasted approximately 10 minutes” (line 292).**

**We have clarified:**

***“The average time to complete both assessments, including preparation activities and data collection, was around 45 minutes per child.” (lines 236-237)***

**And:**

***“Assessors did not have previous medical training nor prior experience with the technologies.” (lines 233-234)***

**We did not conduct a formal costing of the assessments.**

18)	Finally - SRT in particular appears to be a more sensitive marker of neurological and developmental impact than other traditional measurements such as growth or developmental markers, but it is a pity that these positive SRT effects seem to have faded a year after the intervention ended. Do you think that CHW should continue household interventions with beyond 2 years - and if so, how regularly and for how long? This may be worth raising in your discussion and conclusions.

**Response: Thank you for this comment. We have added the following text to the Discussion:**

***“Extending the intervention period to age 3 and perhaps even beyond might have generated larger and longer-lasting effects [56].” (lines 558-559)***

**New reference [56]: Black MM, Pérez-Escamilla R, Fernandez Rao S. Integrating nutrition and child development interventions: scientific basis, evidence of impact, and implementation considerations. Advances in Nutrition. 2015;6: 852-9.**

**Reviewer #4**

Review of the Manuscript: PMEDICINE-D-22-03227R1

Impact of a community health worker home visit intervention on child development in South Africa: A cluster-randomized controlled trial

This manuscript addresses a very important topic, which is the effectiveness of an early childhood home visiting intervention implemented within an existing community health care system. While such programs have been found to improve developmental outcomes in research studies, further work is needed to understand how they can be scaled up with high fidelity and maintain effectiveness. Given the importance of this question, it is unfortunate that there is very little information on program implementation in the manuscript. Information on measures of program coverage are reported in unexpected places, such as the statistical analysis section and the Discussion, instead of the Methods where they would be expected to be reported. Other information on key factors such as implementation fidelity, workforce capacity, workforce coverage, and equity of access to the program are missing. Further comments on implementation information:

-	Page 10, line 135 "The job aid was designed to be effort neutral by restructuring the already allotted time." This sentence is not clear. Does it mean that adding the use of the job aid during the visit did not change the amount of time the CHW was intended to spend at the household? How much time was allotted? What were the other activities the CHW were meant to do at the visit before adding the job aid? How did the structure of the visit change with the addition of the job aid?

**Response: We mention in the Methods that *“preliminary work indicated that CHW home visits for young children generally lasted 30 minutes”* (lines 61-62). We have added an additional paragraph to the Methods describing the standard of care in the study area, with detail on the structure of CHW home visits prior to the introduction of the job aid, which continued in the control arm:**

***“Participants in the control arm received the local standard of care throughout the study period. CHWs in the study area are responsible for making monthly home visits for all children under the age of two and for providing a broad range of services during those visits, including health screening, immunization monitoring, and nutrition counselling. The Road to Health Booklet serves as a guide for CHW visits as well as a record of the child’s growth and vaccination status. It also provides caregivers with important information on child health, nutrition, and common developmental milestones. The job aid used in the intervention arm expanded the content of home visits but was designed to be effort neutral by restructuring the time CHWs were already responsible for allotting.” (lines 162-170)***

-	Page 10, lines 135-137 Who were the trainers? Were the trainers members of the research team or was the training also integrated into the existing system? If the trainers were research personnel, how would the program be implemented on a large scale within the existing system? What about supervisors? How were the supervisors trained and how did they monitor CHW performance? What about their level of education and CHW level of education? All of this is very important to consider for scaling up the intervention.

**Response: We have clarified:**

***“******Trainings were conducted by members of the research team with logistical support from a local non-governmental organization that specialized in training CHWs for the Limpopo Department of Health.” (lines 174-176)***

**With respect to supervision, we state:**

***“We integrated the intervention job aid and training into the existing CHW home visit infrastructure. We did not intervene in the local system of CHW supervision. As a result, important aspects of implementation were not under our control.” (lines 576-578)***

**We have added text stating that 81% of study CHWs had completed high school (line 589). We do not have data on the level of education of supervisors.**

-	Page 18-19, lines 320-323. The description of how CHW compliance was measured belongs earlier in the description of data collection methods, not in the Statistical Analysis section.

**Response: We have added a description of the collection of implementation and compliance data to the Methods:**

***“******Finally, data on implementation of the intervention were collected during a set of phone-based interviews conducted with one randomly selected caregiver served by each intervention CHW after the intervention had been running for one year. Caregivers were asked to self-report the time since the CHW last visited their home and whether at the last visit the CHW counseled the caregiver on the child development content of the job aid. These interviews were not conducted in the control arm. Our initial plan was to measure CHW compliance based on caregiver self-report at endline, but because of the delay in fieldwork due to COVID-19, the recall period was too long to generate quality data.” (lines 209-216)***

-	Were any other data collected on fidelity of program implementation? C.A.R.E. (consolidated advice for reporting ECD implementation research) guidelines recommend: "Describe the methods and tools used to assess fidelity of intervention delivery and receipt of intervention by recipients (e.g., self-report, observation, competency tests, checklists, and monitoring records on recipient participation), sources of data, and at which stage of the project implementation was fidelity assessed. If relevant, describe whether these data were collected in the comparison conditions." Other aspects of the CARE guidelines would also be beneficial to improve the manuscript.

**Response: Thank you for this suggestion. We have added detail on the measurement of program implementation factors:**

***“Finally, data on implementation of the intervention were collected during a set of phone-based interviews conducted with one randomly selected caregiver served by each intervention CHW after the intervention had been running for one year. Caregivers were asked to self-report the time since the CHW last visited their home and whether at the last visit the CHW counseled the caregiver on the child development content of the job aid. These interviews were not conducted in the control arm. Our initial plan was to measure CHW compliance based on caregiver self-report at endline, but because of the delay in fieldwork due to COVID-19, the recall period was too long to generate quality data.” (lines 209-216)***

**We have also included an additional result related to implementation:**

***“Among intervention CHWs who made a home visit in the prior month, 93% counselled caregivers on the child development content of the job aid.” (lines 504-505)***

Several issues regarding the sample selection and sample size need to be addressed:

-	It is not clear how many CHWs were employed in the catchment area, how many of those were invited to participate, and of those invited, how many did participate. Therefore, it is difficult to evaluate whether the sample enrolled in the trial was likely to be representative of the population.

**Response: We have provided additional detail on CHW participation in the study and trainings:**

***“Overall, 376 eligible CHWs were recruited for the study, of whom 356 (95%) participated. All participating CHWs in the intervention arm attended the first training, while 170 (83%) attended the second training, 183 (90%) attended the third training, and 186 (91%) attended the fourth training.” (lines 432-435)***

-	Were the authors able to conduct any assessment of whether the sample enrolled in the trial was likely to be representative of the population?

**Response: As per our response to Reviewer 2’s comment above, we have strong reason to believe that our recruitment protocol reached a sample that is representative of the population in the study area. We have added the following to the Methods:**

***“According to the South Africa Demographic and Health Survey 2016 [13], 98% of births in Limpopo occur at health facilities and the vast majority are at public clinics and hospitals.” (lines 71-73)***

**New reference [13]: National Department of Health (NDoH), Statistics South Africa (Stats SA), South African Medical Research Council (SAMRC), and ICF. South Africa Demographic and Health Survey 2016. Pretoria, South Africa, and Rockville, Maryland, USA: NDoH, Stats SA, SAMRC, and ICF, 2016.**

-	Page 18, lines 301-303. For a cluster-randomized trial, the power analysis must be based on the number of clusters, not the total number of individuals. The sample-size determination usually involves either the determination of the number of clusters given cluster size or the determination of cluster size given the number of clusters. Either way, it must be stated the number of clusters on which the power analysis was based.

**Response: Our power calculation accounted for the cluster-randomized design. We have clarified that our target sample size based on our power calculation was 1,092 dyads enrolled across 51 clusters:**

***“The target sample size of 1,092 dyads enrolled across 51 clusters . . .” (line 372)***

-	What was the power for the sub-sample?

**Response: We have added a description of the power calculation for the lab subsample:**

***“******The target for the lab subsample was 900 assessments across the three waves, which provided 80% power to detect a 0.25 SD difference in absolute gamma power and SRT, assuming 85% of assessments would meet the preset validity criteria for inclusion in the analysis, a negligible ICC for geographic clustering, and an ICC for repeated measures within dyads of 0.20.” (lines 375-379)***

Abstract:

-	First sentence of abstract "achieving scale" is not clear what this means.

**Response: We have replaced this text with “expanding access” (Abstract).**

-	Methods: Please state the target n of the EEG subsample. Clarify whether the 'lab subsample' is the same as the EEG subsample. Was EEG the only assessment conducted at the lab visit? Where were other outcome assessments conducted? It's not clear which outcomes were assessed on the full sample and which were assessed on the subsample.

**Response: We have expanded the description of data collection in the abstract:**

***“Household surveys were administered to the full study sample at baseline and endline. Demographic data and household asset information were collected during the baseline survey. Data on caregiver engagement were collected during baseline and endline surveys. Data on child anthropometry, development, and diet were collected from the full sample during endline surveys only. In a subsample of children, electroencephalography (EEG) and eye-tracking measures of neural function were assessed at a lab concurrent with endline and at two interim time points.” (Abstract)***

-	Findings: Please state the attrition of clusters as well as individuals (at endline, were there still 51 clusters)?

**Response: We have clarified this point:**

***“At endline (last assessment June 11, 2021), 432 dyads (71%; 175 dyads not assessed at endline) in 26 clusters remained in the intervention group and 332 dyads (68%; 156 dyads not assessed at endline) in 25 clusters remained in the control group.” (Abstract)***

-	Conclusions: The statement "we found evidence of an effect on neural function" is confusing and misleading because the effect on SRT indicated a positive effect of the intervention on neural maturity, while the effect on EEG power indicated a negative effect of the intervention on neural maturity, which is not clear in the abstract.

**Response: We have changed this statement throughout the paper to *“we found significant improvement in SRT.”* (Abstract; lines 524-525)**

**We have added the following interpretation of the EEG results to the abstract (the Discussion has similar text):**

***“The similar effect sizes on absolute gamma and total EEG power—and the corresponding null effect on relative gamma power—suggest an anatomical explanation rather than evidence of a negative effect on neural function.” (Abstract)***

-	The statement "Strengthening supervision of CHWs to improve compliance is necessary to maximizing impact" does not follow from any of the results presented in the Abstract.

**Response: We have removed this statement from the abstract.**

Other comments:

Page 4, line 10 Evidence on their effectiveness to do what?

**Response: We have updated this statement:**

***“Evidence of the impact of early childhood services delivered by CHWs on child health and development is mixed” (lines 10-11)***

Page 4, line 16 "requires careful planning" is not clear. Does this mean the program directors/supervisors of the CHWs need to plan carefully how to implement the program? Or does this mean the CHWs need to plan carefully to manage their daily schedules? Or something else?

**Response: We have clarified this point:**

***“******Effectively integrating child development services into their set of responsibilities requires careful planning, including establishing a manageable schedule and ensuring adequate support, to avoid additional overburdening.” (lines 16-18)***

Page 5, line 41-42: How many CHWs were invited to participate?

**Response: We have added the following to the Results:**

***“******Overall, 376 eligible CHWs were recruited for the study, of whom 356 (95%) participated.” (line 432-433)***

Page 6, line 61: How many? What was the target sample size for the subsample? Were all mother-child dyads in the target sub-district invited?

**Response: We have clarified that *“all study dyads residing in Greater Tzaneen subdistrict”* (line 77) were invited to visit the lab. We have also clarified:**

***“The target for the lab subsample was 900 assessments across the three waves, which provided 80% power to detect a 0.25 SD difference in absolute gamma power and SRT, assuming 85% of assessments would meet the preset validity criteria for inclusion in the analysis, a negligible ICC for geographic clustering, and an ICC for repeated measures within dyads of 0.20.” (lines 375-379)***

Page 20, line 351-352. In this sentence, it is not clear what "observations included" and "those excluded" is referring to. Is this to assess potential bias in loss to follow up for the main outcomes or potential bias in the subsample? I don't see any information on the former in supporting information. It is also difficult to assess this from Table 1 because there is no information on the group lost to follow-up specifically.

**Response: The statement being referred to is a comparison of observations within the lab subsample, across groupings based on preset validity criteria for data inclusion in the analysis.**

**We have added new columns in Table 1 summarizing the demographics of dyads not assessed at endline for each arm. We have added the following text:**

***“In the control group, there was a significant positive association between number of older siblings and probability of being assessed at endline. There were no other significant differences between those assessed and those not assessed at endline.” (lines 441-444)***

**We did not find evidence of systematic differences. We mention this in the Discussion:**

***“Based on Table 1, there does not appear to be a systematic relationship between attrition and treatment group or participant demographics, supporting the use of the complete-case approach to dealing with missing data in the analysis.” (lines 602-604)***

Table S2. It is not clear where the number included and excluded is coming from. These numbers in Table S2 do not match the numbers in Figure 2 Trial Profile.

**Response: We have updated Fig 2 to include the numbers of SRT and EEG observations included and excluded from the analysis at each wave based on preset validity criteria.**

Page 23, line 409-411. In the per protocol analysis, what proportion of the control group was used as the comparison group and how was this selected?

**Response:** **We have clarified:**

***“The full control group was used as the comparison group under the assumption that control CHWs did not have access to the intervention materials or training.” (line 406-407)***

Page 28, line 504-505. The conclusion that strengthening supervision of CHWs is necessary to maximizing impact does not follow from the evidence of the study because no information on supervision was presented or analyzed.

**Response: We have removed mention of strengthening supervision from the abstract and main paper.**

Page 28, line 505-506. It is not clear what evidence is the basis of the conclusion that this study demonstrates the feasibility of collecting neural markers like EEG and SRT. Is it based on the result that 91% of EEG observations and 78% of SRT observations were met the pre-set validity criteria for inclusion in the analysis? This result was not discussed or interpreted. Is this what would be expected to demonstrate the feasibility of the method? Or is this conclusion based on a different result?

**Response: We have added the following text to the first paragraph of the Discussion:**

***“******Furthermore, we were able to collect high quality data on neural function using EEG and eye-tracking technologies, confirming that the use of these measures is feasible in low-resource settings.” (lines 525-527)***
