# Supplementary material for: Evaluation of a community health worker home visit intervention to improve child development in South Africa: A cluster-randomized controlled trial
Source: PLoS Med. 2023 Apr 14;20(4):e1004222. doi: 10.1371/journal.pmed.1004222 (PMC10146459; doi:10.1371/journal.pmed.1004222)
Supplement: Attachment — Submitted filename: Response to reviewers R3.docx [file pmed.1004222.s011.docx]

**Requests from the editors:**

Please add line numbering to your manuscript.

**Response: We have extended the line numbering to the full manuscript.**

Please update your title to ‘Evaluation of a community health worker home visit intervention on child development in South Africa: A cluster-randomized controlled trial’

**Response: We have changed the title to “Evaluation of a community health worker home visit intervention to improve child development in South Africa: A cluster-randomized controlled trial.” If this is not acceptable, we are happy to change it to the suggested title.**

Please consider including additional intervention resources as Supporting Information or uploading them to an online repository and citing this in the manuscript.

**Response: We have included the key intervention resources in S4 Figure.**

Please provide Figure 1 as a separate file per our guidelines: [https://journals.plos.org/plosmedicine/s/figures](https://urldefense.proofpoint.com/v2/url?u=https-3A__journals.plos.org_plosmedicine_s_figures&d=DwMGaQ&c=WO-RGvefibhHBZq3fL85hQ&r=z27-kON3ecDsqmXs7Qc8ZVwMCVT4vcRpRBSXUKjMCnc&m=S2GUD94bRKTH4r3t3HUDIOMV-GOJCmS-IwjcQmpoga9EDQbHCdtKEc772eelyih-&s=IURNsKXOu_Z89cDQ25WGYxleLisVsCIUUsR5G9TUer0&e=)

**Response: We have provided all figures and confirmed that they comply with journal guidelines.**

Suggestions for shortening the Abstract:

* Delete ‘’but masking of participating CHWs and dyads was not possible’.

**Response: We have removed this text.**

* Shorten the description of the intervention and control states (the latter can be described as standard of care).

**Response: We have shortened the description of the intervention and control states.**

* The list of covariates can be removed and described simply as ‘a set of demographic covariates’.

**Response: We have removed the list of covariates.**

* Delete ‘175 dyads not assessed at endline’.

**Response: We have removed the text describing the number of dyads not assessed at endline for the intervention and control groups.**

* Details of data collection can be trimmed (current abstract text is duplicated in the Methods).

**Response: We have trimmed the description of the data collection.**

* The Abstract Conclusions can be shortened. Please interpret the study based on the results presented in the abstract, emphasizing what is new without overstating your conclusions.

**Response: We have shortened the Abstract Conclusions.**

Please avoid recycling text from the Abstract in your Author Summary, which is intended for a non-specialist audience.

**Response: We have revised the text in the Author Summary to be more appropriate for a non-specialist audience.**

Author Summary: Please include headline numbers, including sample size and key findings.

**Response: We have added numbers in the description of the sample size and key findings.**

Your Author Summary mentions '764 study children' but this is not mentioned elsewhere in the manuscript. Please ensure numbers are consistent throughout the text.

**Response: We have updated the numbers presented in the Author Summary to be consistent with the rest of the manuscript.**

Please consider moving some of the more technical aspects of the Methods to the Supporting Information (for instance, lab assessments).

**Response: We have removed some technical aspects of the description of the lab assessments from the main text and added a new Supporting Information file (S6 Text) with the full description.**

Please replace the term “compliance” with “adherence”.

**Response: We have made this change throughout the manuscript.**

Please ensure all Internet sources include a date of citation (e.g., #30).

**Response: We have added dates of citation to references #30 and #55.**

**Reviewer #1:**

Reviewer #1: Thank you to the authors for addressing my previous comments well. Just two minor clarifications/recommendations: 1) if the analysis used a complete cases approach then the assumption would be missing completely at random (MCAR) rather than missing at random (MAR);

**Response: We have revised the text to indicate that our assumption is “missing completely at random” (line 482).**

2) I would recommend that non pre-specified outcomes are referred to as exploratory outcomes rather than secondary.

**Response: We have changed “secondary outcomes” to “exploratory outcomes” throughout the text (line 449 and Table 3).**

**Reviewer #4**

The revised manuscript is much improved. A few additional comments:

Line 168-169. "The job aid used in the intervention arm expanded the content of home visits but was designed to be effort neutral by restructuring the time CHWs were already responsible for allotting." The difference between the intervention and control groups is still not clear. What activities or information were delivered in the control group that were not delivered in the intervention group? A table comparing the content and activities covered by the CHWs in each group would be useful.

**Response: We have added detail on how the activities and information delivered by control CHWs were integrated into the intervention:**

***“The Road to Health Booklet served as a guide for control CHW visits as well as a record of the child’s growth and vaccination status. It also provided caregivers with important information on child health, nutrition, and common developmental milestones. Intervention CHWs continued to use the Road to Health Booklet to record child growth and vaccination status, and the key health promotion messages in the Road to Health Booklet were integrated into the job aid.” (lines 282-287)***

Line 528-592. "To our knowledge, no previous trial in an LMIC has examined the impact of an early-life intervention on EEG power or SRT." Please see:

Larson, L. M., D. Feuerriegel, M. I. Hasan, S. Braat, J. Jin, S. M. M. U. Tipu, S. Shiraji, F. Tofail, B.-A. Biggs, J. Hamadani, K. Johnson, S.-R. Pasricha and S. Bode (2023). "Supplementation With Iron Syrup or Iron-Containing Multiple Micronutrient Powders Alters Resting Brain Activity in Bangladeshi Children." The Journal of Nutrition 153(1): 352-363.

Prado, E. L., K. Maleta, B. L. Caswell, M. George, L. M. Oakes, M. C. DeBolt, M. G. Bragg, C. D. Arnold, L. L. Iannotti, C. K. Lutter and C. P. Stewart (2020). "Early Child Development Outcomes of a Randomized Trial Providing 1 Egg Per Day to Children Age 6 to 15 Months in Malawi." J Nutr 150(7): 1933-1942.

Response time on the IOWA task is a type of saccadic reaction time.

**Response: Thank you for bringing these citations to our attention. We have added them to the manuscript (#42 & #43) along with a third citation for another recent nutrition trial in Malawi that used the IOWA task (#44) and revised the related text:**

***“This study is one of the first in an LMIC to examine the impact of an early-life intervention on EEG power and SRT. These technologies generate robust markers of neural function and are portable and well suited for use in low resource field settings. One recent trial conducted in the United States found that providing households with unconditional cash transfers had a positive impact on infant EEG high frequency power [41]. Another recent trial in Bangladesh found evidence of a positive short-term effect of iron supplementation on EEG alpha power over sensorimotor cortical regions [42]. No significant effects were found for other EEG frequency bands and the effect on alpha power was not found at a later follow-up assessment. A small pilot study recently conducted in Sierra Leone demonstrated the feasibility of assessing SRT in a low-resource setting and found a relationship between the measure and supplementary feeding in moderately malnourished children [21]. In addition, two recent trials in Malawi that tested nutrition interventions found no significant effects on response time for the Infant Orienting with Attention task [43,44], an eye-tracking measure that is similar to our measure of SRT.” (lines 613-625)***
